# Synthesis of 2*H*-azirine-2,2-dicarboxylic acids and their derivatives

**DOI:** 10.3762/bjoc.20.264

**Published:** 2024-12-05

**Authors:** Anastasiya V Agafonova, Mikhail S Novikov, Alexander F Khlebnikov

**Affiliations:** 1 Saint Petersburg State University, Institute of Chemistry, 7/9 Universitetskaya Naberezhnaya, St. Petersburg 199034, Russia

**Keywords:** azirine-2,2-dicarboxamides, azirine-2,2-dicarboxylic acids, isomerization, isoxazoles

## Abstract

Methods for the preparation of 3-aryl-2*H*-azirine-2,2-dicarboxylic acids and their amides, esters, and azides by FeCl_2_-catalyzed isomerization of 3-aryl-5-chloroisoxazole-4-carbonyl chlorides into 3-aryl-2*H*-azirine-2,2-dicarbonyl dichlorides followed by their reaction with nucleophiles are reported. Two approaches to the preparation of 3-aryl-5-chloroisoxazole-4-carbonyl chlorides have been developed.

## Introduction

**Scheme 1:**
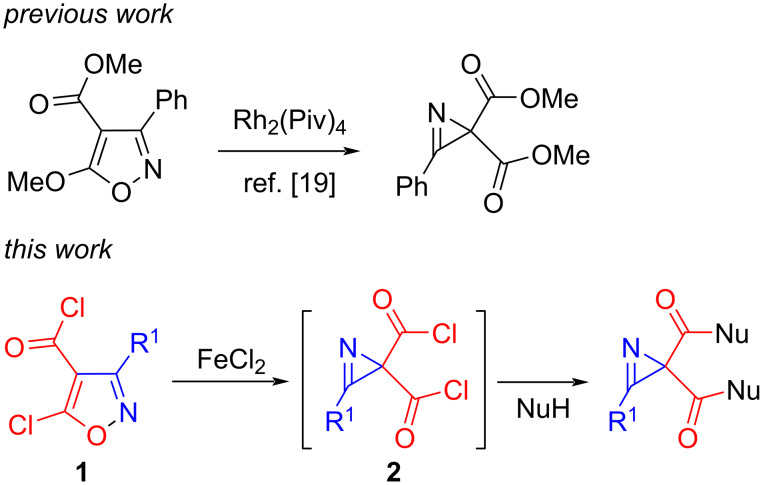
Approaches to 2*H*-azirine-2,2-dicarboxylic acid derivatives.

The isomerization of isoxazoles, containing a heteroatomic substituent at C5, to 2*H*-azirines is a powerful method for the preparation of 2*H*-azirine-2-carboxylic acid derivatives [1]. In particular, the catalytic isomerization of 5-chloroisoxazoles allows the generation of azirine-2-carbonyl chlorides, which can be easily converted into a variety of azirine-2-carboxylic acid derivatives by reactions with nucleophilic reagents. Using this approach, numerous 2-(1*H*-pyrazol-1-ylcarbonyl)-2*H*-azirines, 1-(2*H*-azirine-2-carbonyl)benzotriazoles, 2*H*-azirine-2-carbonyl azides, anhydrides, amides, esters, and thioesters of azirine carboxylic acids, as well as azirine carboxylic acids themselves, have been prepared over the last decade (see [2] and references therein). Azirine-2-carboxylic acid derivatives are not only valuable synthetic building blocks [3–11] but also show useful biological activities [12–18]. Although many 2,2-bifunctionalized azirines have been synthesized [3–11], the synthesis of only one 2*H*-azirine-2,2-dicarboxylic acid derivative, dimethyl 3-phenyl-2*H*-azirine-2,2-dicarboxylate, has been reported to date. This compound was prepared by a Rh_2_(Piv)_4_-catalyzed isomerization of methyl 5-methoxy-3-phenylisoxazole-4-carboxylate [19]. The described linear synthesis, unfortunately, allows obtaining only one azirine-2,2-dicarboxylic acid derivative from a certain isoxazole precursor. Herein, we would like to report a method for the synthesis of 2*H*-azirine-2,2-dicarboxylic acids and their various derivatives from a single starting material, 3-substituted 2*H*-azirine-2,2-dicarbonyl dichloride **2**, via the reaction with nucleophiles (Scheme 1). Two approaches to the preparation of diacyl chlorides **2** without using noble metals have also been developed.

## Results and Discussion

5-Сhloroisoxazole-4-carbonyl chlorides **1**, required for the preparation of 2*H*-azirine-2,2-dicarboxylic acids and their derivatives, were synthesized using two reaction sequences (Table 1). The first sequence involved the chloroformylation of isoxazolones **3** to 5-chloroisoxazole-4-carbaldehydes **4** by POCl_3_/DMF [20–23], followed by radical chlorination of **4** with SO_2_Cl_2_/AIBN [24]. The alternative route to acid chlorides **1** included oxidation of aldehydes **4** with Oxone to acids **5** and the conversion of the latter into acid chlorides with thionyl chloride.

**Table 1:** Synthesis of 5-chloroisoxazole-4-carbonyl chlorides.

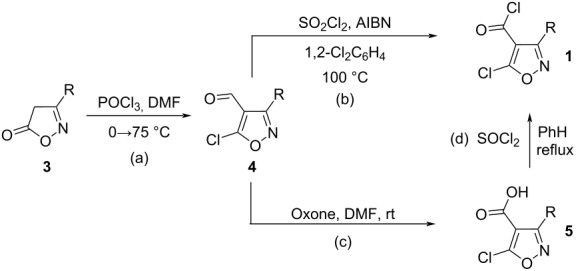					

entry	**1**, **3**, **4**, **5**	R	yield of **4** (%)	yield of **5** (%)	yield of **1** (%)

1	**a**	Ph	53	–	77^a^ (b)
2	**b**	4-MeC_6_H_4_	20	98	25 (b)/84 (d)
3	**c**	4-CF_3_C_6_H_4_	64	–	83^a^ (b)
4	**d**	3-MeOC_6_H_4_	35	97	– (b)/84 (d)
5	**e**	4-FC_6_H_4_	47	–	84^a^ (b)
6	**f**	4-ClC_6_H_4_	63	–	94^a^ (b)
7	**g**	4-BrC_6_H_4_	50	97	– (b)/77 (d)
8	**h**	2-BrC_6_H_4_	14	99	– (b)/84(d)
9	**i**	4-NO_2_C_6_H_4_	64	99	0 (b)/92 (d)
10	**j**	*t*-Bu	69	92	–/99 (d)

^a^Isolation without chromatography.

The first reaction sequence was suitable for obtaining compounds **1a**–**c**,**e**,**f** with substituents tolerant to radical reaction conditions. A significant advantage of the method is that chromatography was not required to isolate the products. At the same time, compound **4i** proved to be inactive under the used chlorination conditions, compounds **4g**,**h** underwent partial hydrodebromination in the aryl substituent in the same step, while compound **4j** yielded a product with difficult to separate impurities. In these cases, as well as in reactions giving low aldehyde yields in the first step (**4b**,**d**), the second developed reaction sequence turned out to be more effective. In the second approach, the oxidation of aldehydes **4** with Oxone to acids **5** occurs with yields close to quantitative, and the conversion of the latter to the acid chlorides **1** with thionyl chloride proceeded with yields of 77–92%. This made it possible to synthesize the target isoxazoles **1b**,**d**,**g**–**j** with fairly high yields.

**Scheme 2:**
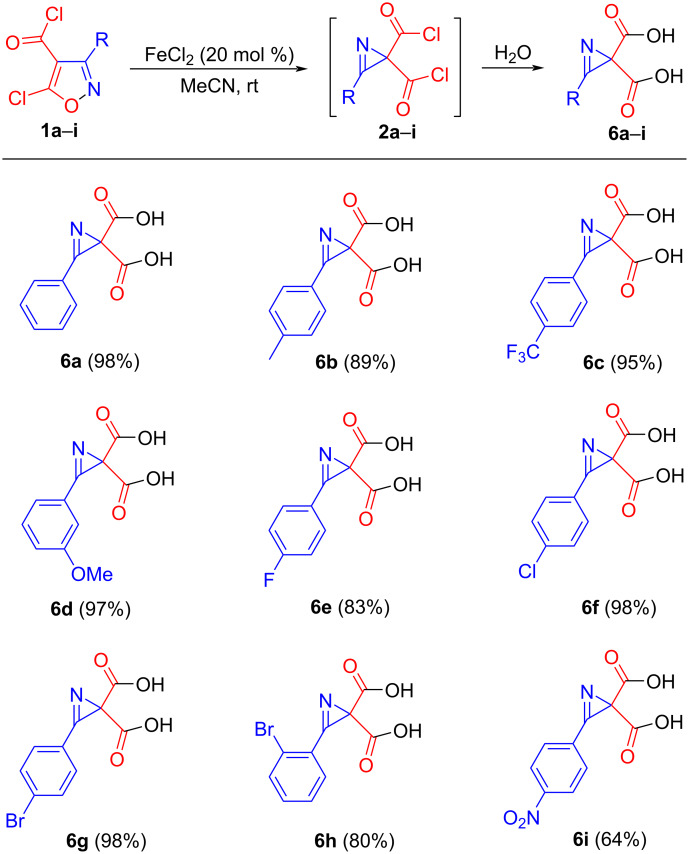
Synthesis of 2*H*-azirine-2,2-dicarboxylic acids **6**.

Having in hand a set of isoxazoles **1a**–**i** containing aryl substituents at the 3-position of the isoxazole ring, both with electron-donating and electron-withdrawing groups, and the *tert*-butyl-substituted isoxazole **1j**, we proceeded to obtain dicarboxylic acids **6** (Scheme 2). The isomerization of isoxazoles **1** into diacyl chlorides **2** was achieved by applying the conditions for the isomerization of 3-aryl-5-chloroisoxazoles [25–27] using anhydrous FeCl_2_ as a catalyst and carrying out the reaction in acetonitrile at rt for 2 h. After TLC showed the disappearance of the starting isoxazoles **1**, the reaction mixture was treated with water and acids **6a**–**i** were isolated in 64–98% yield. Isoxazole **1j** did not isomerize at room temperature, which is typical for highly sterically congested isoxazoles containing a 3-*tert-*butyl substituent [26]. The mechanism of such isomerizations of isoxazoles has been previously discussed using DFT calculations [25–26], which revealed the formation of an isoxazole–Fe complex, which facilitates the cleavage of the N–O bond and subsequent 1,3-cyclization, ultimately leading to the formation of 2*H*-azirine.

**Scheme 3:**
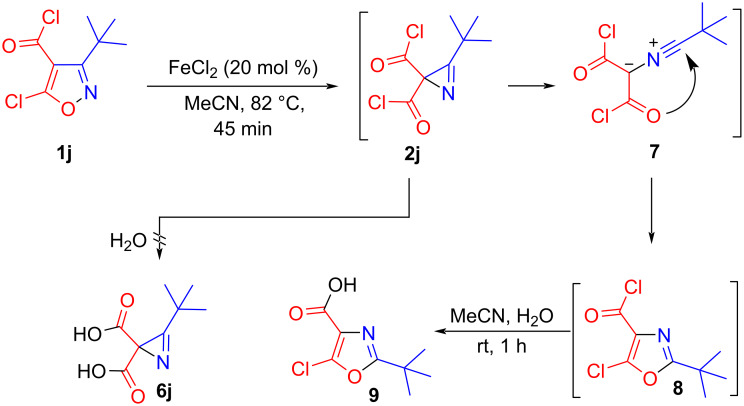
Transformations of 3-(*tert*-butyl)-5-chloroisoxazole-4-carbonyl chloride (**1j**).

Therefore, the isomerization of isoxazole **1j** was carried out at a higher temperature, 82 °C, but after hydrolysis of the reaction mixture, instead of the expected azirine dicarboxylic acid **6j**, oxazole-4-carboxylic acid **9** was isolated. Apparently, azirine **2j** underwent ring opening at higher temperature to nitrile ylide **7**, which after cyclization and hydrolysis gave acid **9** (Scheme 3) (cf., e.g. [23]).

Next, given that the preparation of 2*H*-azirine-2-carboxamides from 2*H*-azirine-2-carbonyl chlorides is challenging [27], we proceeded to carefully optimize the conversion of 2*H*-azirine-2,2-dicarbonyl dichlorides **2** to 2*H*-azirine-2,2-dicarboxamides **10** using isoxazole **1a** and benzylamine as starting materials (Table 2). It turned out that the previously found optimal reaction conditions for the preparation of amides from azirine-2-carbonyl chlorides [27] are not suitable for obtaining bis-amides from azirine-2,2-dicarbonyl dichlorides. In order to obtain a maximum yield, it is better in this case, to carry out the reaction with 2 equiv of the amine in the presence of 4 equiv of Cs_2_CO_3_ to trap hydrogen chloride. Additionally, the workup procedure, in which the product is isolated by filtration through celite after reaction with the amine, often allows one to obtain higher yields than an aqueous treatment of the reaction mixture.

**Table 2:** Optimization of amide preparation.

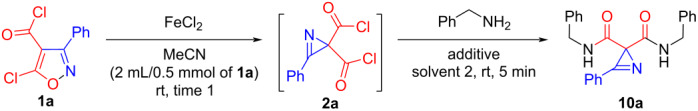						

Entry	FeCl_2_ (mol %)	time 1 (h)	additive (equiv)	BnNH_2_ (equiv)	solvent 2	yield of **10a** (%)

1^a^	20	2	2-MePy (2)	2	PhMe	14
2	20	2	2-MePy (2)	2	PhMe	39
3	20	2	2-MePy (2)	2	–	38
4	20	2	2-MePy (2)	3	–	30
5	20	2	DMAP (2)	3	–	9
6	20	2	ClC(O)OEt (1) + 2-TMSPy (1)	6	–	19
7	20	2	–	4	–	14
8	20	2	K_2_CO_3_ (4)	2	–	45
9	20	2	Cs_2_CO_3_ (4)	2	–	72
10^b^	20	2	Cs_2_CO_3_ (4)	2	–	73
11	5	4	Cs_2_CO_3_ (4)	2	–	10

^a^The residue obtained from the isomerization of **1a → 2a** was diluted with dry Et_2_O (50 mL), the precipitated FeCl_2_ was filtered off and after evaporation of Et_2_O, **2a** was dissolved in anhydrous toluene. ^b^Filtration through celite after reaction with the amine (without aqueous work-up).

**Scheme 4:**
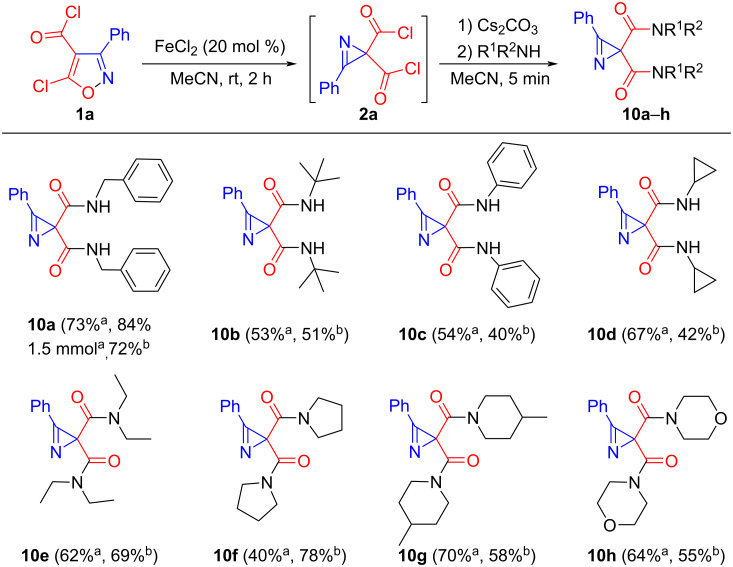
Synthesis of amides **10**. ^a^Filtration through celite after reaction with amine (without aqueous workup). ^b^Work up with H_2_O.

A number of amides **10a**–**h** were obtained from isoxazole **1a** and primary and secondary amines according to the conditions described in entries 9 and 10 in Table 2, with yields of up to 78% (Scheme 4). An experiment with benzylamine and isoxazole **1a** on a 1.5 mmol scale gave diamide **10a** in 84% yield. The structure of compound **10h** was confirmed by single-crystal X-ray diffraction analysis. The reaction of azetidine with diacyl chloride **2a** gave a complex mixture of products, and *O*-methyl hydroxylamine did not react.

**Scheme 5:**
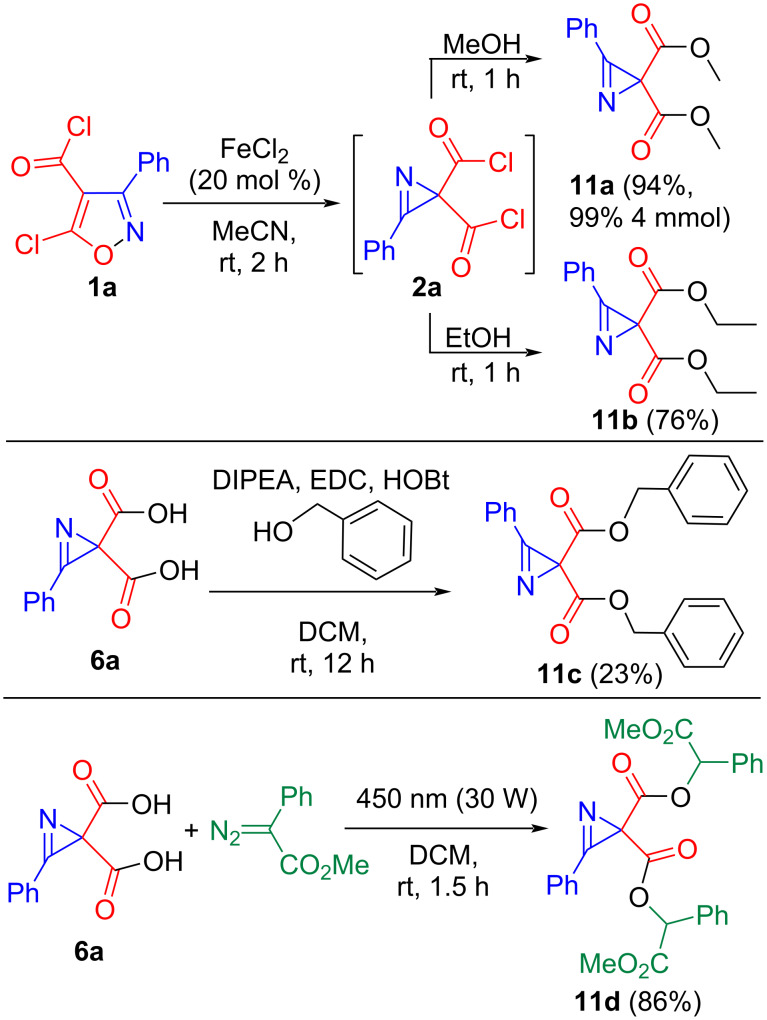
Synthesis of esters **11**.

Diacyl chloride **2a** reacts with methanol and ethanol to give diesters **11a**,**b** (Scheme 5). An experiment with isoxazole **1a** and methanol on a 4 mmol scale gave dimethyl ester **11a** in 99% yield. Unexpectedly, the reaction of branched alcohols with diacyl chloride **2a** failed. For example, the reaction with benzyl alcohol resulted in the formation of an overly complex mixture of products. Adding bases to trap HCl did not improve the situation. Dibenzyl ester **11c** was prepared using traditional activation of carboxylic acid **6a**, although the yield was only 23%. A higher yield of the branched ester **11d** (86%, as a mixture of diastereomers) was obtained by carbene insertion, generated by blue LED irradiation of methyl 2-diazo-2-phenylacetate, into the O–H bonds of diacid **6a** (Scheme 5). Apparently, in this case, the reaction proceeds through a less sterically congested transition state.

**Scheme 6:**
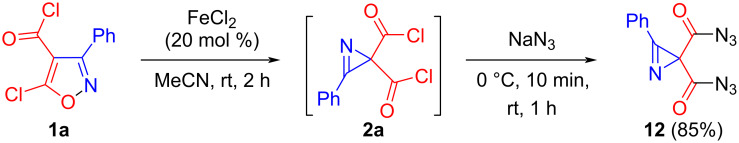
Synthesis of dicarbonyl azide **12**.

Diacyl chloride **2a** was also reacted with sodium azide as nucleophile at room temperature giving dicarbonyl azide **12** in 85% yield (Scheme 6).

## Conclusion

Two reaction sequences for the synthesis of 3-aryl-5-chloroisoxazole-4-carbonyl chlorides have been developed. These compounds are convenient precursors for the preparation of 2*H*-azirine-2,2-dicarboxylic acids and their derivatives such as amides, esters and azides, via an Fe(II)-catalyzed room temperature isomerization to 3-aryl-2*H*-azirine-2,2-dicarbonyl dichlorides followed by their fast reaction at the same temperature with O- and N-nucleophiles. 3-Aryl-2*H*-azirine-2,2-dicarboxylic acids were prepared in 64–98% yield, whereas 3-(*tert*-butyl)-2*H*-azirine-2,2-dicarboxylic acid could not be obtained by this method because the isomerization of 3-(*tert*-butyl)-5-chloroisoxazole-4-carbonyl chloride did not occur at room temperature, but at elevated temperature (82 °C) the reaction proceeded via the formation of the nitrile ylide, which cyclized to 2-(*tert*-butyl)-5-chlorooxazole-4-carbonyl chloride. 3-Phenyl-2*H*-azirine-2,2-dicarboxamides were prepared using primary and secondary amines in 53–84% yield, and the reaction is scalable. Methyl and ethyl esters of 3-phenyl-2*H*-azirine-2,2-dicarboxylic acid were prepared in 76–99% yield from 3-phenyl-2*H*-azirine-2,2-dicarbonyl dichloride and methanol or ethanol, but the reaction of more branched alcohols failed. Such esters could be prepared from the dicarboxylic acids using traditional activation or OH-insertion reaction of carbenes formed by irradiation of the appropriate diazo compound.

## Supporting Information

File 1 Experimental procedures and characterization data of new compounds.

## Data Availability

All data that supports the findings of this study is available in the published article and/or the supporting information of this article.
