# Supplementary material for: Synthesis of 2H-azirine-2,2-dicarboxylic acids and their derivatives
Source: Beilstein J Org Chem. 2024 Dec 5;20:3191–7. doi: 10.3762/bjoc.20.264 (PMC11635289; doi:10.3762/bjoc.20.264)
Supplement: File 1 — Experimental procedures and characterization data of new compounds. [file Beilstein_J_Org_Chem-20-3191-s001.pdf]

BEILSTEIN JOURNAL OF ORGANIC CHEMISTRY

Supporting Information

for

Synthesis of 2*H*-azirine-2,2-dicarboxylic acids and their derivatives

Anastasiya V. Agafonova, Mikhail S. Novikov and Alexander F. Khlebnikov

Beilstein J. Org. Chem. **2024,** *20,* 3191–3197. doi:10.3762/bjoc.20.264

Experimental procedures and characterization data of new compounds

License and Terms: This is a supporting information file under the terms of the Creative Commons Attribution License (https://creativecommons.org/licenses/by/4.0). Please note that the reuse, redistribution and reproduction in particular requires that the author(s) and source are credited and that individual graphics may be subject to special legal provisions.
The license is subject to the *Beilstein Journal of Organic Chemistry* terms and conditions: (https://www.beilstein-journals.org/bjoc/terms)

Table of contents

1. Experimental procedures and characterization data of new compounds S2

2. NMR spectra S23

3. X-ray data S78

4. References S80

1. Experimental procedures and characterization data of new compounds

General information and methods. Melting points were determined on a melting point apparatus. ^{1}H (400 MHz), ^{13}C (100 MHz), ^{19}F (376 MHz) spectra were recorded on Bruker AVANCE NMR spectrometer in $CDCl_3$, DMSO-d_6 or C_6D_6. Chemical shifts (δ) are reported in parts per million downfield from tetramethylsilane (TMS, δ = 0.00). ^{1}H NMR spectra were calibrated according to the residual peak of $CDCl_3$ (7.26 ppm), DMSO-d_6 (2.50 ppm), and C_6D_6 (7.16 ppm); ^{13}C{1H} were calibrated according to the peak of $CDCl_3$ (77.00 ppm), DMSO-d_6 (39.51 ppm), and C_6D_6 (128.00 ppm). Electrospray ionization (ESI) mass spectra were recorded on a Bruker MaXis mass spectrometer, HRMS-ESI-QTOF. Single-crystal X-ray data were collected by means of a SuperNova, Dual, Cu at home/near, Atlas diffractometer. The crystals of **10h** were measured at a temperature of 100.00(10) K, using monochromated Cu Kα radiation. Crystallographic data for the structures **10h** (CCDC 2368949) have been deposited with the Cambridge Crystallographic Data Centre. Thin-layer chromatography (TLC) was conducted on aluminum sheets with 0.2 mm silica gel with a fluorescent indicator. Physical and spectral data of isoxazol-5(4*H*)-ones **3a**,**b**,**f**,**g**,**h** [1], **3c**,**e** [2], **3d** [3], **3i** [4], **3j** [5] prepared according to the published procedures, were in agreement with previously reported values.

General procedure A for the preparation of 5-chloro-3-isoxazole-4-carbaldehydes 4.

N,*N*-Dimethylformamide (6.2 mL, 80 mmol, 8 equiv) was added dropwise over 5 min to phosphorus oxychloride (15.0 mL, 160 mmol, 16 equiv) at 0 °C (water–ice bath) and the reaction mixture was stirred for an additional 30 min at the same temperature. Then, isoxazol-5(4*H*)-one (**3**, 10 mmol) was added and the reaction mixture was heated at 75 °C for 0.5–2 h. After reaction completion (according to TLC), the pre-cooled mixture was poured very slowly into an ice–water mixture (300 mL) under vigorous stirring. The formed mixture was extracted with EtOAc (3 × 75 mL). The combined organic layers were washed with brine (300 mL) and dried over Na_2SO_4. After evaporation of the solvent under reduced pressure, the residue was purified by column chromatography on silica gel (eluent light petroleum/EtOAc) to give 5-chloro-1-formylisoxazoles **4**.

Caution! *$POCl_3$ is corrosive and toxic by ingestion, inhalation or contact with skin and eyes and should be used with protective measures to avoid direct contact.*

*5-Chloro-3-phenylisoxazole-4-carbaldehyde (**4a**)* [6]. Compound **4a** was prepared following the general procedure A from DMF (6.2 mL, 80 mmol, 8 equiv), $POCl_3$ (15.0 mL, 160 mmol, 16 equiv), isoxazolone **3a** (1.61 g, 10 mmol) for 1.5 h, in 1.1 g (53% yield), after column chromatography on silica (light petroleum/ethyl acetate, (from 75:1 to 10:1, (v/v)) as a colorless solid: mp 43–44 °C (Et_2O–hexane). ^1H NMR ($CDCl_3$, 400 MHz): δ 9.95 (s, 1H), 7.79–7.75 (m, 2H), 7.55–7.48 (m, 3H); $^{13}C\{^1H\}$ NMR ($CDCl_3$, 100 MHz): δ 181.7, 163.2, 162.4, 131.1, 129.0, 128.8, 126.2, 113.2. HRMS–ESI $[M + MeOH + Ag]^+$ calcd for $C_{11}H_{10}Ag^{37}ClNO_3^+$ 347.9392; found 347.9389.

*5-Chloro-3-(p-tolyl)isoxazole-4-carbaldehyde (**4b**)* [6]. Compound **4b** was prepared following the general procedure A from DMF (6.2 mL, 80 mmol, 8 equiv), $POCl_3$ (15.0 mL, 160 mmol, 16 equiv), isoxazolone **3b** (1.75 g, 10 mmol) for 1.5 h, in 443 mg (20% yield), after column chromatography on silica (light petroleum/ethyl acetate, (from 20:1 to 10:1, (v/v)) as a beige solid: mp 86–87 °C (Et_2O–hexane). ^1H NMR ($CDCl_3$, 400 MHz): δ 9.95 (s, 1H), 7.76–7.61 (m, 2H), 7.38–7.27 (m, 2H), 2.43 (s, 3H); $^{13}C\{^1H\}$ NMR ($CDCl_3$, 100 MHz): δ 181.9, 163.2, 162.3, 141.6, 129.5, 128.9, 123.3, 113.2, 21.5. HRMS–ESI $[M + H]^+$ calcd for $C_{11}H_9{}^{35}ClNO_2^+$ 222.0317; found 222.0313.

*5-Chloro-3-(4-(trifluoromethyl)phenyl)isoxazole-4-carbaldehyde (**4c**).* Compound **4c** was prepared following the general procedure A from DMF (6.2 mL, 80 mmol, 8 equiv), $POCl_3$ (15.0 mL, 160 mmol, 16 equiv), isoxazolone **3c** (2.29 g, 10 mmol) for 0.5 h, in 1.76 g (64% yield), after column chromatography on silica (light petroleum/ethyl acetate, (from 10:1 to 5:1, (v/v)) as a beige solid: m.p. 46–47 °C (Et_2O–hexane); ^1H NMR ($CDCl_3$, 400 MHz): δ 9.97 (s, 1H), 7.96 (d, J = 8.1 Hz, 2H), 7.77 (d, J = 8.1 Hz, 2H); $^{13}C\{^1H\}$ NMR ($CDCl_3$, 100 MHz): δ 181.3, 163.6, 161.8, 133.0 (q, J = 32.8 Hz), 129.8, 129.5, 125.7 (q, J = 3.8 Hz), 123.7 (q, J = 272.5 Hz), 113.4. $^{19}F\{^1H\}$

NMR ($CDCl_3$, 376 MHz): δ -63.1. HRMS–ESI $[M + MeOH + H]^+$ calcd for $C_{12}H_{10}{}^{35}ClF_3NO_3^+$ 308.0296; found 308.0287.

5-Chloro-3-(3-methoxyphenyl)isoxazole-4-carbaldehyde (4d). Compound **4d** was prepared following the general procedure A from DMF (6.2 mL, 80 mmol, 8 equiv), $POCl_3$ (15.0 mL, 160 mmol, 16 equiv), isoxazolone **3d** (1.91 g, 10 mmol) for 0.5 h, in 832 mg (35% yield), after column chromatography on silica (light petroleum/ethyl acetate, (from 20:1 to 4:1, (v/v)) as a pale yellow solid: m.p. 36–37 °C (Et_2O–hexane); ^1H NMR ($CDCl_3$, 400 MHz): δ 9.96 (s, 1H), 7.45–7.39 (m, 1H), 7.38–7.32 (m, 2H), 7.11–7.07 (m, 1H), 3.87 (s, 3H); $^{13}C\{^1H\}$ NMR ($CDCl_3$, 100 MHz): δ 181.8, 163.1, 162.4, 159.8, 129.9, 127.3, 121.3, 117.3, 114.0, 113.3, 55.4. HRMS–ESI $[M + H]^+$ calcd for $C_{11}H_9{}^{35}ClNO_3^+$ 238.0266; found 238.0256.

5-Chloro-3-(4-fluorophenyl)isoxazole-4-carbaldehyde (4e) [7]. Compound **4e** was prepared following the general procedure A from DMF (6.2 mL, 80 mmol, 8 equiv), $POCl_3$ (15.0 mL, 160 mmol, 16 equiv), isoxazolone **3e** (1.79 g, 10 mmol) for 1.5 h, in 1.06 g (47% yield), after column chromatography on silica (light petroleum/ethyl acetate, (from 20:1 to 10:1, (v/v)) as a colorless solid: m.p. 55–56 °C (Et_2O–hexane); ^1H NMR ($CDCl_3$, 400 MHz): δ 9.95 (s, 1H), 7.88–7.78 (m, 2H), 7.23–7.13 (m, 2H); $^{13}C\{^1H\}$ NMR ($CDCl_3$, 100 MHz): δ 181.6, 165.8, 162.7 (d, J = 121.0 Hz), 131.2 (d, J = 8.5 Hz), 122.4 (d, J = 3.7 Hz), 116.1, 115.9, 113.2. $^{19}F\{^1H\}$ NMR ($CDCl_3$, 376 MHz): δ -108.3. HRMS–ESI $[M + Ag]^+$ calcd for $C_{10}H_5Ag^{37}ClFNO_2^+$ 333.9035; found 333.0938.

5-Chloro-3-(4-chlorophenyl)isoxazole-4-carbaldehyde (4f) [6]. Compound **4f** was prepared following the general procedure A from DMF (6.2 mL, 80 mmol, 8 equiv), $POCl_3$ (15.0 mL, 160 mmol, 16 equiv), isoxazolone **3f** (1.96 g, 10 mmol) for 2 h, in 1.52 g (63% yield), after column chromatography on silica (light petroleum/ethyl acetate, (from 20:1 to 10:1, (v/v)) as a pale yellow solid: m.p. 55–56 °C (Et_2O–hexane); ^1H NMR ($CDCl_3$, 400 MHz): δ 9.94 (s, 1H), 7.83–7.71 (m, 2H), 7.53–7.41 (m, 2H); $^{13}C\{^1H\}$ NMR ($CDCl_3$, 100 MHz): δ 181.5, 163.4, 162.0, 137.6, 130.4, 129.1, 124.8, 113.3. HRMS–ESI $[M + H]^+$ calcd for $C_{10}H_6{}^{35}Cl_2NO_2^+$ 241.9771; found 241.9769.

3-(4-Bromophenyl)-5-chloroisoxazole-4-carbaldehyde ***(4g)***. Compound **4g** was prepared following the general procedure A from DMF (6.2 mL, 80 mmol, 8 equiv), $POCl_3$ (15.0 mL, 160 mmol, 16 equiv), isoxazolone **3g** (2.40 g, 10 mmol) for 0.5 h, in 1.43 g (50% yield), after column chromatography on silica (light petroleum/ethyl acetate, (from 20:1 to 4:1, (v/v)) as a beige solid: m.p. 55–56 °C (Et_2O–hexane); ^{1}H NMR ($CDCl_3$, 400 MHz): δ 9.95 (s, 1H), 7.74–7.67 (m, 2H), 7.67–7.59 (m, 2H); ^{13}C{^{1}H} NMR ($CDCl_3$, 100 MHz): δ 181.5, 163.4, 162.1, 132.0, 130.5, 125.9, 125.2, 113.2. HRMS–ESI [M + H]$^+$ calcd for $C_{10}H_6{}^{79}Br^{35}ClNO_2{}^+$ 285.9265; found 285.9261.

3-(2-Bromophenyl)-5-chloroisoxazole-4-carbaldehyde ***(4h)***. Compound **4h** was prepared following the general procedure A from DMF (6.2 mL, 80 mmol, 8 equiv), $POCl_3$ (15.0 mL, 160 mmol, 16 equiv), isoxazolone **3h** (2.40 g, 10 mmol) for 1 h, in 401 mg (14% yield), after column chromatography on silica (light petroleum/ethyl acetate, (from 10:1 to 4:1, (v/v)) as a brown solid: m.p. 58–59 °C (Et_2O–hexane); ^{1}H NMR ($CDCl_3$, 400 MHz): δ 9.77 (s, 1H), 7.78–7.67 (m, 1H), 7.48–7.39 (m, 3H); ^{13}C{^{1}H} NMR ($CDCl_3$, 100 MHz): δ 181.2, 163.0, 160.5, 133.3, 132.2, 131.4, 128.0, 127.7, 123.0, 114.1. HRMS–ESI [M + H]$^+$ calcd for $C_{10}H_6{}^{79}Br^{35}ClNO_2{}^+$ 285.9265; found 285.9263.

5-Chloro-3-(4-nitrophenyl)isoxazole-4-carbaldehyde ***(4i)*** [6]. Compound **4i** was prepared following the general procedure A from DMF (6.2 mL, 80 mmol, 8 equiv), $POCl_3$ (15.0 mL, 160 mmol, 16 equiv), isoxazolone **3i** (2.06 g, 10 mmol) for 1.5 h, in 1.62 g (64% yield), after column chromatography on silica (light petroleum/ethyl acetate, (from 20:1 to 3:1, (v/v)) as a pale yellow solid: m.p. 121–122 °C (Et_2O– hexane); ^{1}H NMR ($CDCl_3$, 400 MHz): δ 9.98 (s, 1H), 8.41–8.29 (m, 2H), 8.10–8.00 (m, 2H); ^{13}C{^{1}H} NMR ($CDCl_3$, 100 MHz): δ 181.2, 164.2, 161.1, 149.4, 132.4, 130.3, 123.7, 113.4. HRMS–ESI [M + Ag]$^+$ calcd for $C_{10}H_5Ag^{35}ClN_2O_4{}^+$ 358.8984; found 358.8973.

3-(tert-Butyl)-5-chloroisoxazole-4-carbaldehyde ***(4j)*** [8]. Compound **4j** was prepared following the general procedure A from DMF (6.2 mL, 80 mmol, 8 equiv), $POCl_3$ (15.0 mL, 160 mmol,

16 equiv), isoxazolone **3i** (1.41 g, 10 mmol) for 1.5 h, in 1.29 g (69% yield), after column chromatography on silica (light petroleum/ethyl acetate, (from 20:1 to 10:1, (v/v)) as a yellow oil: ^{1}H NMR ($CDCl_3$, 400 MHz): δ 9.91 (s, 1H),1.40 (s, 9H); ^{13}C{^{1}H} NMR ($CDCl_3$, 100 MHz): δ 181.8, 170.7, 164.5, 113.7, 34.0, 27.6. HRMS–ESI [M + H]$^+$ calcd for $C_8H_{11}{}^{35}ClNO_2^+$ 188.0473; found 188.0479.

General procedure B for the synthesis of 5-chloroisoxazole-4-carboxylic acid 5.

5-Chloroisoxazole-4-carbaldehyde **4** (5 mmol) was dissolved in DMF (10 mL). Then, Oxone (1.3 equiv) was added in one portion and the reaction mixture was stirred at ambient temperature for 12 h (for compound **5j**: heating at 100 °C for 7 h). The reaction mixture was treated with 1 M HCl (150 mL) to dissolve the salts and extracted with EtOAc (3 × 50 mL). The combined organic layers were washed with brine (100 mL) and dried over Na_2SO_4. After evaporation of the solvent under reduced pressure, the residue was recrystallized from EtOAc/hexane mixture.

*5-Chloro-3-(p-tolyl)isoxazole-4-carboxylic acid (**5b**).* Compound **5b** was prepared following the general procedure B from carbaldehyde **4b** (1.11 g, 5 mmol), Oxone (2.0 g, 6.5 mmol, 1.3 equiv) in 1.16 g (98% yield) as a yellow solid: m.p. 137–138 °C (EtOAc–hexane); ^{1}H NMR ($CDCl_3$, 400 MHz): δ 11.84 (bs, 1H), 7.65–7.45 (m, 2H), 7.37–7.17 (m, 2H), 2.43 (s, 3H); ^{13}C{^{1}H} NMR ($CDCl_3$, 100 MHz): δ 165.5, 164.3, 161.6, 140.9, 129.2, 129.1, 124.0, 106.7, 21.5. HRMS–ESI [M + H]$^+$ calcd for $C_{11}H_9{}^{35}ClNO_3^+$ 238.0266; found 238.0267.

*5-Chloro-3-(3-methoxyphenyl)isoxazole-4-carboxylic acid (**5d**).* Compound **5d** was prepared following the general procedure B from carbaldehyde **4d** (1.19 g, 5 mmol), Oxone (2.0 g, 6.5 mmol, 1.3 equiv) in 1.23 g (97% yield) as a colorless solid: m.p. 116–117 °C (EtOAc–hexane); ^{1}H NMR (DMSO-d_6, 100 MHz) δ 13.62 (bs, 1H), 7.45–7.38 (m, 1H), 7.23–7.18 (m, 2H), 7.14–7.09 (m, 1H), 3.79 (s, 3H); ^{13}C{^{1}H} NMR (DMSO-d_6, 100 MHz): δ 163.7, 160.5, 158.8, 158.7, 129.4, 128.5, 121.3, 116.0, 114.7, 108.4, 55.2. HRMS–ESI [M + H]$^+$ calcd for $C_{11}H_9{}^{35}ClNO_4^+$ 254.0215; found 254.0216.

*3-(4-Bromophenyl)-5-chloroisoxazole-4-carboxylic acid (**5g**).* Compound **5g** was prepared following the general procedure B from carbaldehyde **4g** (1.43 g, 5 mmol), Oxone (2.0 g, 6.5 mmol, 1.3 equiv) in 1.47 g (97% yield) as a colorless solid: m.p. 168–169 °C (EtOAc–hexane); ^{1}H NMR (DMSO-d_6, 100 MHz) δ 13.61 (bs, 1H), 7.79–7.66 (m, 2H), 7.66–7.51 (m, 2H); ^{13}C{^{1}H} NMR (DMSO-d_6, 100 MHz): δ 163.2, 160.4, 159.1, 131.3 (2C), 126.6, 124.1, 108.3. HRMS–ESI [M - H]$^{-}$ calcd for $C_{10}H_4{}^{81}Br^{35}ClNO_3{}^{-}$ 301.9048; found 301.9045.

*3-(2-Bromophenyl)-5-chloroisoxazole-4-carboxylic acid (**5h**).* Compound **5h** was prepared following the general procedure B from carbaldehyde **4h** (1.43 g, 5 mmol), Oxone (2.0 g, 6.5 mmol, 1.3 equiv) in 1.50 g (99% yield) as a pale yellow solid: m.p. 152–152 °C (EtOAc–hexane); ^{1}H NMR (DMSO-d_6, 100 MHz) δ 13.54 (bs, 1H), 7.83–7.71 (m, 1H), 7.60–7.42 (m, 3H); ^{13}C{^{1}H} NMR (DMSO-d_6, 100 MHz): δ 163.9, 160.0, 158.5, 132.4, 132.0, 131.3, 129.3, 127.6, 122.7, 109.3. HRMS–ESI [M - H]$^{-}$ calcd for $C_{10}H_4{}^{81}Br^{35}ClNO_3{}^{-}$ 301.9048; found 301.9048.

*5-Chloro-3-(4-nitrophenyl)isoxazole-4-carboxylic acid (**5i**).* Compound **5i** was prepared following the general procedure B from carbaldehyde **4i** (1.26 g, 5 mmol), Oxone (2.0 g, 6.5 mmol, 1.3 equiv) in 1.33 g (99% yield) as a colorless solid: m.p. 194–196 °C (EtOAc–hexane); ^{1}H NMR (DMSO-d_6, 100 MHz) δ 13.77 (bs, 1H), 8.44–8.22 (m, 2H), 8.05–7.81 (m, 2H); ^{13}C{^{1}H} NMR (DMSO-d_6, 100 MHz): δ 162.8, 160.3, 159.5, 148.6, 133.7, 130.9, 123.3, 108.6. HRMS–ESI [M - H]$^{-}$ calcd for $C_{10}H_4{}^{35}ClN_2O_5{}^{-}$ 266.9814; found 266.9807.

*3-(tert-Butyl)-5-chloroisoxazole-4-carboxylic acid (**5j**).* Compound **5j** was prepared following the general procedure B from carbaldehyde **4j** (938 mg, 5 mmol), Oxone (2.0 g, 6.5 mmol, 1.3 equiv) in 937 mg (92% yield) as a colorless solid: m.p. 118–119 °C (EtOAc–hexane); ^{1}H NMR ($CDCl_3$, 400 MHz): δ 11.44 (bs, 1H), 1.46 (s, 9H); ^{13}C{^{1}H} NMR ($CDCl_3$, 100 MHz): δ 171.5, 166.3, 162.1, 106.8, 34.2, 27.9. HRMS–ESI [M - H]$^{-}$ calcd for $C_8H_8{}^{35}ClNO_3{}^{-}$ 202.0276; found 202.0284.

General procedure C for the synthesis of 5-chloroisoxazole-4-carbonyl chlorides 1.

Sulfuryl chloride (3.9 mmol, 1.3 equiv) was added to a solution of 5-chloroisoxazole-4-carbaldehyde **4** (3 mmol) and AIBN (2 mol %) in 1,2-dichlorobenzene (6 mL). The reaction mixture was heated at 100 °C for 0.5–3 h. After evaporation of the solvent under reduced pressure, the residue was recrystallized from an Et_2O/hexane mixture.

General procedure D for the synthesis of 5-chloroisoxazole-4-carbonyl chlorides 1.

Thionyl chloride (9 mmol, 3 equiv) was added to a solution of 5-chloroisoxazole-4-carboxylic acid **5** (3 mmol) in benzene (15 mL). The resulting reaction mixture was refluxed for 0.5–3 h. After evaporation of the solvent under reduced pressure, the residue was purified by column chromatography on silica gel (eluent EtOAc/light petroleum) to give 5-chloroisoxazole-4-carbonyl chlorides **1**.

*5-Chloro-3-phenylisoxazole-4-carbonyl chloride (**1a**).* Compound **1a** was prepared following the general procedure C from 5-chloroisoxazole-4-carbaldehyde **4a** (623 mg, 3 mmol), AIBN (10 mg, 0.06 mmol, 2 mol %) in 559 mg (77% yield) for 1 h as a colorless solid: m.p. 46–47 °C (Et_2O–hexane); ^{1}H NMR ($CDCl_3$, 400 MHz): δ 7.69–7.38 (m, 5H); ^{13}C{^{1}H} NMR ($CDCl_3$, 100 MHz): δ 163.7, 161.6, 158.2, 131.0, 129.2, 128.6, 126.2, 112.4. HRMS–ESI [M – H]$^-$ calcd for $C_{10}H_4{}^{35}Cl_2NO_2^-$ 239.9624; found 239.9606.

*5-Chloro-3-(p-tolyl)isoxazole-4-carbonyl chloride (**1b**).* Compound **1b** was prepared following the general procedure C from 5-chloroisoxazole-4-carbaldehyde **4b** (665 mg, 3 mmol), AIBN (10 mg, 0.06 mmol, 2 mol %) in 192 mg (25% yield) for 1.5 h. Compound **1b** was also prepared following the general procedure D from 5-chloroisoxazole-4-carboxylic acid **5b** (713 mg, 3 mmol), thionyl chloride (1.07 g, 9 mmol, 3 equiv) in 645 mg (84% yield) for 0.5 h, after column chromatography on silica (light petroleum/ethyl acetate, (20:1 (v/v)).

A colorless solid: m.p. 67–67 °C (Et_2O–hexane); ^{1}H NMR ($CDCl_3$, 400 MHz): δ 7.49–7.42 (m, 2H), 7.33–7.27 (m, 2H), 2.43 (s, 3H); ^{13}C{^{1}H} NMR ($CDCl_3$, 100 MHz): δ 163.6, 161.4, 158.2,

141.4, 129.3, 129.0, 123.3, 112.4, 21.5. HRMS–ESI [M – Cl]$^-$ calcd for $C_{11}H_7{}^{35}ClNO_2{}^-$ 220.0170; found 220.0159.

5-Chloro-3-(4-(trifluoromethyl)phenyl)isoxazole-4-carbonyl chloride (***1c***). Compound **1c** was prepared following the general procedure C from 5-chloroisoxazole-4-carbaldehyde **4c** (827 mg, 3 mmol), AIBN (10 mg, 0.06 mmol, 2 mol %) in 772 mg (83% yield) for 0.5 h as a colorless solid: m.p. 86–87 °C (Et_2O–hexane); ^{1}H NMR ($CDCl_3$, 400 MHz): δ 7.82–7.74 (m, 2H), 7.74–7.66 (m, 2H); ^{13}C{^{1}H} NMR ($CDCl_3$, 100 MHz): δ 162.6, 162.2, 158.0, 133.1 (q, *J* = 32.8 Hz), 129.8, 129.8, 125.6 (q, *J* = 3.8 Hz), 123.6 (q, *J* = 272.6 Hz), 112.4. ^{19}F{^{1}H} NMR ($CDCl_3$, 376 MHz): δ -63.1. HRMS–ESI [M + MeOH - Cl]$^-$ calcd for $C_{12}H_8{}^{35}ClF_3NO_3{}^-$ 306.0150; found 306.0142.

*5-Chloro-3-(3-methoxyphenyl)isoxazole-4-carbonyl chloride (**1d**).* Compound **1d** was prepared following the general procedure D from 5-chloroisoxazole-4-carboxylic acid **5d** (761 mg, 3 mmol), thionyl chloride (1.07 g, 9 mmol, 3 equiv) in 686 mg (84% yield) for 0.5 h, after column chromatography on silica (light petroleum/ethyl acetate, (20:1 (v/v)) as a colorless oil: ^{1}H NMR ($CDCl_3$, 400 MHz): δ 7.41–7.31 (m, 1H), 7.13–7.01 (m, 3H), 3.82 (s, 3H); ^{13}C{^{1}H} NMR ($CDCl_3$, 100 MHz): δ 163.5, 161.5, 159.5, 158.1, 129.7, 127.3, 121.5, 116.9, 114.4, 112.5, 55.4. HRMS–ESI [M + Na + Ag]$^{2+}$ calcd for $C_{11}H_7Ag^{35}Cl_2NNaO_3{}^{2+}$ 400.8741; found 400.8755.

*5-Chloro-3-(4-fluorophenyl)isoxazole-4-carbonyl chloride (**1e**).* Compound **1e** was prepared following the general procedure C from 5-chloroisoxazole-4-carbaldehyde **4e** (677 mg, 3 mmol), AIBN (10 mg, 0.06 mmol, 2 mol %) in 772 mg (84% yield) for 2 h as a colorless solid: m.p. 80–81 °C (Et_2O–hexane); ^{1}H NMR ($CDCl_3$, 400 MHz): δ 7.61–7.53 (m, 2H), 7.22–7.14 (m, 2H); ^{13}C{^{1}H} NMR ($CDCl_3$, 100 MHz): δ 165.7, 162.8, 162.5 (d, *J* = 127.8 Hz), 158.1, 131.4 (d, *J* = 8.6 Hz), 122.3 (d, *J* = 3.7 Hz), 115.9 (d, *J* = 22.3 Hz), 112.3. ^{19}F{^{1}H} NMR ($CDCl_3$, 376 MHz): δ -108.5. HRMS–ESI [M - Cl]$^-$ calcd for $C_{10}H_4{}^{35}ClFNO_2{}^-$ 223.9920; found 223.9906.

*5-Chloro-3-(4-chlorophenyl)isoxazole-4-carbonyl chloride (**1f**).* Compound **1f** was prepared following the general procedure C from 5-chloroisoxazole-4-carbaldehyde **4f** (726 mg, 3 mmol), AIBN (10 mg, 0.06 mmol, 2 mol %) in 780 mg (94% yield) for 3 h as a colorless solid: m.p. 75–

76 °C (Et_2O–hexane); ^1H NMR ($CDCl_3$, 400 MHz): δ 7.58–7.42 (m, 4H); $^{13}C\{^1H\}$ NMR ($CDCl_3$, 100 MHz): δ 162.8, 162.0, 158.1, 137.5, 130.5, 129.0, 124.7, 112.3. HRMS–ESI [M - Cl]$^-$ calcd for $C_{10}H_4{}^{35}Cl_2NO_2{}^-$ 239.9624; found 239.9606.

3-(4-Bromophenyl)-5-chloroisoxazole-4-carbonyl chloride (1g). Compound **1g** was prepared following the general procedure D from 5-chloroisoxazole-4-carboxylic acid **5g** (908 mg, 3 mmol), thionyl chloride (1.07 g, 9 mmol, 3 equiv) in 741 mg (77% yield) for 1 h, after column chromatography on silica (light petroleum/ethyl acetate, (from 20:1 to 10:1 (v/v)) as a colorless solid: m.p. 92–94 °C (EtOAc–hexane); ^1H NMR ($CDCl_3$, 400 MHz): δ 7.69–7.58 (m, 2H), 7.50–7.36 (m, 2H); $^{13}C\{^1H\}$ NMR ($CDCl_3$, 100 MHz): δ 162.8, 162.0, 158.1, 131.9, 130.7, 125.8, 125.1, 112.3. HRMS–ESI [M - Cl]$^-$ calcd for $C_{10}H_4{}^{79}Br^{35}ClNO_2{}^-$ 283.9119; found 283.9123.

3-(2-Bromophenyl)-5-chloroisoxazole-4-carbonyl chloride (1h). Compound **1h** was prepared following the general procedure D from 5-chloroisoxazole-4-carboxylic acid **5h** (908 mg, 3 mmol), thionyl chloride (1.07 g, 9 mmol, 3 equiv) in 741 mg (84% yield) for 0.5 h, after column chromatography on silica (light petroleum/ethyl acetate, (20:1 (v/v)) as a colorless oil: ^1H NMR ($CDCl_3$, 400 MHz): δ 7.75–7.65 (m, 2H), 7.51–7.35 (m, 2H); $^{13}C\{^1H\}$ NMR ($CDCl_3$, 100 MHz): δ 163.2, 161.1, 157.6, 133.0, 132.1, 131.1, 128.4, 127.6, 123.5, 113.2. HRMS–ESI [M - Cl]$^-$ calcd for $C_{10}H_4{}^{79}Br^{35}ClNO_2{}^-$ 283.9119; found 283.9119.

5-Chloro-3-(4-nitrophenyl)isoxazole-4-carbonyl chloride (1i). Compound **1i** was prepared following the general procedure D from 5-chloroisoxazole-4-carboxylic acid **5i** (806 mg, 3 mmol), thionyl chloride (1.07 g, 9 mmol, 3 equiv) in 792 mg (92% yield) for 3 h, after column chromatography on silica (light petroleum/ethyl acetate, (from 20:1 to 10:1 (v/v)) as a colorless solid: m.p. 94–95 °C (Et_2O–hexane); ^1H NMR ($CDCl_3$, 400 MHz): δ 8.42–8.33 (m, 2H), 7.83–7.75 (m, 2H); $^{13}C\{^1H\}$ NMR ($CDCl_3$, 100 MHz): δ 162.6, 162.1, 158.0, 149.4, 132.4, 130.5, 123.7, 112.4. HRMS–ESI [M - Cl]$^-$ calcd for $C_{10}H_4{}^{35}ClN_2O_4{}^-$ 250.9865; found 250.9852.

3-(tert-Butyl)-5-chloroisoxazole-4-carbonyl chloride (1j). Compound **1j** was prepared following the general procedure D from 5-chloroisoxazole-4-carboxylic acid **5j** (611 mg, 3 mmol), thionyl

chloride (1.07 g, 9 mmol, 3 equiv) in 660 mg (99% yield) for 0.5 h, after column chromatography on silica (light petroleum/ethyl acetate, (from 20:1 to 10:1 (v/v)) as a colorless oil: ^{1}H NMR ($CDCl_3$, 400 MHz): δ 1.39 (s, 9H); ^{13}C{^{1}H} NMR ($CDCl_3$, 100 MHz): δ 170.6, 162.0, 159.3, 112.6, 34.2, 27.8. HRMS–ESI [M + H]$^+$ calcd for $C_8H_{10}{}^{35}Cl_2NO_2^+$ 222.0084; found 222.0087.

General procedure E for the synthesis of 2*H*-azirine-2,2-dicarboxylic acids 6.

Anhydrous $FeCl_2$ (26 mg, 0.2 mmol, 0.2 equiv) was added to a solution of 5-chloroisoxazole-4-carbonyl chloride **1** (1 mmol) in acetonitrile (10 mL) under Ar atmosphere. The mixture was stirred at room temperature for 2 h. Then, water (20 mL) was added and the mixture was stirred at room temperature for 1 h. The 2*H*-azirine-2,2-dicarboxylic acid was extracted with EtOAc (3 × 50 mL), washed with brine (50 mL), and dried over Na_2SO_4. After evaporation of the solvent under reduced pressure, the residue was recrystallized from EtOAc/hexanes mixture.

*3-Phenyl-2H-azirine-2,2-dicarboxylic acid (**6a**).* Compound **6a** was prepared following the general procedure E from 5-chloroisoxazole-4-carbonyl chloride **1a** (242 mg, 1 mmol), $FeCl_2$ (26 mg, 0.2 mmol, 0.2 equiv) in 193 mg (98% yield) as a beige solid: m.p. 143–144 °C (EtOAc–hexane); ^{1}H NMR (DMSO-d_6, 400 MHz): δ 7.98–7.90 (m, 2H), 7.83–7.75 (m, 1H), 7.74–7.67 (m, 2H), 6.03 (bs, 2H); ^{13}C{^{1}H} NMR (DMSO-d_6, 100 MHz): δ 169.1, 155.4, 134.7, 130.5, 129.9, 120.0, 38.2. HRMS–ESI [M - H]$^-$ calcd for $C_{10}H_6NO_4^-$ 204.0302; found 204.0304.

Scale up experiment. Anhydrous $FeCl_2$ (52 mg, 0.4 mmol, 0.2 equiv) was added to a solution of 5-chloroisoxazole-4-carbonyl chloride **1a** (484 mg, 2 mmol) in acetonitrile (20 mL) under Ar atmosphere. The mixture was stirred at room temperature for 2 h. Then, water (40 mL) was added and the mixture was stirred at room temperature for 1 h. The 2*H*-azirine-2,2-dicarboxylic acid was extracted with EtOAc (3 × 100 mL), washed with brine (100 mL) and dried over Na_2SO_4. After evaporation of the solvent under reduced pressure, the residue was recrystallized from EtOAc/hexane mixture to give dicarboxylic acid **6a** (332 mg, 81%).

*3-(p-Tolyl)-2H-azirine-2,2-dicarboxylic acid (**6b**).* Compound **6b** was prepared following the general procedure E from 5-chloroisoxazole-4-carbonyl chloride **1b** (256 mg, 1 mmol), $FeCl_2$

(26 mg, 0.2 mmol, 0.2 equiv) in 195 mg (89% yield) as a yellow solid: m.p. 148–149 °C (EtOAc–hexane); ^{1}H NMR (DMSO-d_6, 400 MHz): δ 12.54 (bs, 2H), 7.89–7.77 (m, 2H), 7.57–7.45 (m, 2H), 2.43 (s, 3H); ^{13}C{^{1}H} NMR (DMSO-d_6, 100 MHz): δ 169.3, 154.9, 145.6, 130.6, 130.5, 117.3, 38.1, 21.5. HRMS–ESI [M + Na]$^+$ calcd for $C_{11}H_9NNaO_4^+$ 242.0424; found 242.0427.

*3-(4-(Trifluoromethyl)phenyl)-2H-azirine-2,2-dicarboxylic acid (**6c**).* Compound **6c** was prepared following the general procedure E from 5-chloroisoxazole-4-carbonyl chloride **1c** (310 mg, 1 mmol), $FeCl_2$ (26 mg, 0.2 mmol, 0.2 equiv) in 260 mg (95% yield) as a beige solid: m.p. 147–148 °C (EtOAc–hexane); ^{1}H NMR (DMSO-d_6, 400 MHz): δ 10.71 (bs, 2H), 8.26–8.15 (m, 2H), 8.11–8.01 (m, 2H); ^{13}C{^{1}H} NMR (DMSO-d_6, 100 MHz): δ 168.9, 155.7, 133.9 (q, J = 32.4 Hz), 131.4, 126.9 (q, J = 3.8 Hz),124.1, 123.4 (q, J = 273.0 Hz), 38.7. ^{19}F{^{1}H} NMR (DMSO-d_6, 376 MHz): δ -61.9. HRMS–ESI [M + Na]$^+$ calcd for $C_{11}H_6F_3NNaO_4^+$ 296.0142; found 296.0144.

*3-(3-Methoxyphenyl)-2H-azirine-2,2-dicarboxylic acid (**6d**).* Compound **6d** was prepared following the general procedure E from 5-chloroisoxazole-4-carbonyl chloride **1d** (272 mg, 1 mmol), $FeCl_2$ (26 mg, 0.2 mmol, 0.2 equiv) in 228 mg (97% yield) as a yellow solid: m.p. 153–154 °C (EtOAc–hexane); ^{1}H NMR (DMSO-d_6, 400 MHz): δ 9.99 (bs, 2H), 7.65–7.57 (m, 1H), 7.53–7.45 (m, 2H), 7.39–7.32 (m, 1H), 3.86 (s, 3H); ^{13}C{^{1}H} NMR (DMSO-d_6, 100 MHz): δ 169.1, 160.0, 155.5, 131.2, 123.1, 121.3, 121.2, 114.4, 55.7, 38.5. HRMS–ESI [M + H]$^+$ calcd for $C_{11}H_{10}NO_5^+$ 236.0554; found 236.0556.

*3-(4-Fluorophenyl)-2H-azirine-2,2-dicarboxylic acid (**6e**).* Compound **6e** was prepared following the general procedure E from 5-chloroisoxazole-4-carbonyl chloride **1e** (260 mg, 1 mmol), $FeCl_2$ (26 mg, 0.2 mmol, 0.2 equiv) in 185 mg (83% yield) as a beige solid: m.p. 145–146 °C (EtOAc–hexane); ^{1}H NMR (DMSO-d_6, 400 MHz): δ 8.12–7.99 (m, 2H), 7.61–7.51 (m, 2H), 5.22 (bs, 2H); ^{13}C{^{1}H} NMR (DMSO-d_6, 100 MHz): δ 169.1, 165.6 (d, J = 254.9 Hz), 154.6, 133.6 (d, J = 9.9 Hz), 117.5 (d, J = 22.9 Hz), 116.9 (d, J = 3.2 Hz), 38.4. ^{19}F{^{1}H} NMR (DMSO-d_6, 376 MHz): δ -102.4. HRMS–ESI [M - H]$^-$ calcd for $C_{10}H_5FNO_4^-$ 222.0208; found 222.0207.

*3-(4-Chlorophenyl)-2H-azirine-2,2-dicarboxylic acid (**6f**).* Compound **6f** was prepared following the general procedure E from 5-chloroisoxazole-4-carbonyl chloride **1f** (276 mg, 1 mmol), $FeCl_2$ (26 mg, 0.2 mmol, 0.2 equiv) in 235 mg (98% yield) as a beige solid: m.p. 150–152 °C (EtOAc–hexane); ^{1}H NMR (DMSO-d_6, 400 MHz): δ 8.05–7.89 (m, 2H), 7.85–7.70 (m, 2H), 5.23 (bs, 2H); ^{13}C{^{1}H} NMR (DMSO-d_6, 100 MHz): δ 169.0, 155.0, 139.6, 132.3, 130.2, 119.1, 38.4. HRMS–ESI [M + Na]$^+$ calcd for $C_{10}H_6{}^{35}ClNNaO_4^+$ 261.9878; found 261.9870.

*3-(4-Bromophenyl)-2H-azirine-2,2-dicarboxylic acid (**6g**).* Compound **6g** was prepared following the general procedure E from 5-chloroisoxazole-4-carbonyl chloride **1g** (321 mg, 1 mmol), $FeCl_2$ (26 mg, 0.2 mmol, 0.2 equiv) in 278 mg (98% yield) as a beige solid: m.p. 160–161 °C (EtOAc–hexane); ^{1}H NMR (DMSO-d_6, 400 MHz): δ 7.98–7.84 (m, 4H); ^{13}C{^{1}H} NMR (DMSO-d_6, 100 MHz): δ 169.1, 155.2, 133.2, 132.3, 128.8, 119.4, 38.40. HRMS–ESI [M - H]$^-$ calcd for $C_{10}H_5{}^{79}BrNO_4^-$ 281.9407; found 291.9401.

*3-(2-Bromophenyl)-2H-azirine-2,2-dicarboxylic acid (**6h**).* Compound **6h** was prepared following the general procedure E from 5-chloroisoxazole-4-carbonyl chloride **1h** (321 mg, 1 mmol), $FeCl_2$ (26 mg, 0.2 mmol, 0.2 equiv) in 227 mg (80% yield) as a yellow solid: m.p. 153–154 °C (EtOAc–hexane); ^{1}H NMR (DMSO-d_6, 400 MHz): δ 8.03–7.84 (m, 2H), 7.77–7.64 (m, 2H); ^{13}C{^{1}H} NMR (DMSO-d_6, 100 MHz): δ 169.0, 155.2, 136.1, 134.4, 134.3, 128.9, 124.7, 120.7, 38.3. HRMS–ESI [M + H]$^+$ calcd for $C_{10}H_7{}^{79}BrNO_4^+$ 283.9553; found 283.9558.

*3-(4-Nitrophenyl)-2H-azirine-2,2-dicarboxylic acid (**6i**).* Compound **6i** was prepared following the general procedure E from 5-chloroisoxazole-4-carbonyl chloride **1i** (287 mg, 1 mmol), $FeCl_2$ (26 mg, 0.2 mmol, 0.2 equiv) in 166 mg (64% yield) as a yellow solid: m.p. 145–146 °C (EtOAc–hexane); ^{1}H NMR (DMSO-d_6, 400 MHz): δ 8.55–8.42 (m, 2H), 8.29–8.18 (m, 2H); ^{13}C{^{1}H} NMR (DMSO-d_6, 100 MHz): δ 169.0, 155.5, 150.8, 131.9, 125.8, 124.9, 38.9. HRMS–ESI [M - H]$^-$ calcd for $C_{10}H_5N_2O_6^-$ 249.0153; found 249.0151.

*Synthesis of 2-(tert-butyl)-5-chlorooxazole-4-carboxylic acid (**9**).* Anhydrous $FeCl_2$ (38 mg, 0.3 mmol, 0.3 equiv) was added to a solution of 3-(*tert*-butyl)-5-chloroisoxazole-4-carbonyl

chloride (**1j**, 222 mg, 1 mmol) in acetonitrile (10 mL) under Ar atmosphere. The mixture was refluxed for 45 min. Then, water (20 mL) was added and the mixture was stirred at room temperature for 1 h. The reaction mixture was extracted with EtOAc (3 × 50 mL), washed with brine (50 mL), and dried over Na_2SO_4. After evaporation of the solvent under reduced pressure, the residue was purified by column chromatography on silica gel (light petroleum/ethyl acetate, (from 1:4 to 1:1 (v/v)) to give compound **9** in 67 mg (33% yield) as colorless oil: ^{1}H NMR ($CDCl_3$, 400 MHz): δ 9.62 (bs, 1H), 1.40 (s, 9H); $^{13}C\{^1H\}$ NMR ($CDCl_3$, 100 MHz): δ 170.2, 164.8, 142.6, 125.4, 34.2, 28.1. HRMS–ESI $[M + H]^+$ calcd for $C_8H_{11}{}^{35}ClNO_3{}^+$ 204.0422; found 204.0425.

General procedure F for the synthesis of 2*H*-azirine-2,2-dicarboxamides 10.

Anhydrous $FeCl_2$ (13 mg, 0.1 mmol, 0.2 equiv) was added to a solution of 5-chloroisoxazole-4-carbonyl chloride **1** (0.5 mmol) in acetonitrile (2 mL) under Ar atmosphere. The mixture was stirred at room temperature for 2 h. Then, Cs_2CO_3 (652 mg, 2 mmol, 4 equiv) and amine (1 mmol, 2 equiv) were added consecutively and the mixture was stirred at room temperature for 5 min. The reaction mixture was filtered through a pad of Celite. After evaporation of the solvent under reduced pressure, the residue was purified by column chromatography on silica gel (eluent EtOAc/light petroleum) to give 2*H*-azirine-2,2-dicarboxamide **10**.

General procedure G for the synthesis of 2*H*-azirine-2,2-dicarboxamides 10.

Anhydrous $FeCl_2$ (13 mg, 0.1 mmol, 0.2 equiv) was added to a solution of 5-chloroisoxazole-4-carbonyl chloride **1** (0.5 mmol) in acetonitrile (2 mL) under Ar atmosphere. The mixture was stirred at room temperature for 2 h. Then, Cs_2CO_3 (652 mg, 2 mmol, 4 equiv) and amine (1 mmol, 2 equiv) were added consecutively and the mixture was stirred at room temperature for 5 min. The reaction mixture was treated with water (50 mL) and extracted with EtOAc (3 × 25 mL). The combined organic layers were washed with brine (50 mL) and dried over Na_2SO_4. After evaporation of the solvent under reduced pressure, the residue was purified by column chromatography on silica gel (eluent EtOAc/light petroleum) to give 2*H*-azirine-2,2-dicarboxamide **10**.

*N,N'-Dibenzyl-3-phenyl-2H-azirine-2,2-dicarboxamide (**10a**).* Compound **10a** was prepared following the general procedure F from 5-chloroisoxazole-4-carbonyl chloride **1a** (121 mg, 0.5 mmol), $FeCl_2$ (13 mg, 0.1 mmol, 0.2 equiv), Cs_2CO_3 (652 mg, 2 mmol, 4 equiv) and benzylamine (107 mg, 1 mmol, 2 equiv) in 140 mg (73% yield), after column chromatography on silica (light petroleum/ethyl acetate, (from 3:1 to 1:1 (v/v)). Compound **10a** was also prepared following the general procedure G from 5-chloroisoxazole-4-carbonyl chloride **1a** (121 mg, 0.5 mmol), $FeCl_2$ (13 mg, 0.1 mmol, 0.2 equiv), Cs_2CO_3 (652 mg, 2 mmol, 4 equiv) and benzylamine (107 mg, 1 mmol, 2 equiv) in 138 mg (72% yield), after column chromatography on silica (light petroleum/ethyl acetate, (from 3:1 to 1:1 (v/v)).

A beige solid: m.p. 115–116 °C (Et_2O–hexane); ^1H NMR ($CDCl_3$, 400 MHz): δ 7.97–7.86 (m, 2H), 7.83–7.74 (m, 2H), 7.72–7.67 (m, 1H), 7.64–7.56 (m, 2H), 7.34–7.23 (m, 9H), 4.56–4.42 (m, 4H); $^{13}C\{^1H\}$ NMR ($CDCl_3$, 100 MHz): δ 168.1, 156.4, 137.6, 134.8, 131.2, 129.5, 128.7, 127.6, 127.5, 120.1, 43.7, 38.7. HRMS–ESI $[M + H]^+$ calcd for $C_{24}H_{22}N_3O_2^+$ 384.1707; found 384.1706.

Scale up experiment. Anhydrous $FeCl_2$ (38 mg, 0.3 mmol, 0.2 equiv) was added to a solution of 5-chloroisoxazole-4-carbonyl chloride **1a** (363 mg, 1.5 mmol) in acetonitrile (6 mL) under Ar atmosphere. The mixture was stirred at room temperature for 2 h. Then, Cs_2CO_3 (1.96 g, 6 mmol, 4 equiv) and benzylamine (324 mg, 3 mmol, 2 equiv) were added consecutively and the mixture was stirred at room temperature for 5 min. The reaction mixture was filtered through a pad of Celite. After evaporation of the solvent under reduced pressure, the residue was purified by column chromatography on silica gel (eluent EtOAc/light petroleum from 1:3 to 1:1 (v/v)) to give 2*H*-azirine-2,2-dicarboxamide **10a** (480 mg, 84%).

*N,N'-Di-tert-butyl-3-phenyl-2H-azirine-2,2-dicarboxamide (**10b**).* Compound **10b** was prepared following the general procedure F from 5-chloroisoxazole-4-carbonyl chloride **1a** (121 mg, 0.5 mmol), $FeCl_2$ (13 mg, 0.1 mmol, 0.2 equiv), Cs_2CO_3 (652 mg, 2 mmol, 4 equiv) and *tert*-butylamine (73 mg, 1 mmol, 2 equiv) in 84 mg (53% yield), after column chromatography on silica (light petroleum/ethyl acetate, (from 10:1 to 4:1 (v/v)). Compound **10b** was also prepared

following the general procedure G from 5-chloroisoxazole-4-carbonyl chloride **1a** (121 mg, 0.5 mmol), $FeCl_2$ (13 mg, 0.1 mmol, 0.2 equiv), Cs_2CO_3 (652 mg, 2 mmol, 4 equiv) and *tert*-butylamine (73 mg, 1 mmol, 2 equiv) in 80 mg (51% yield), after column chromatography on silica (light petroleum/ethyl acetate, (from 10:1 to 4:1 (v/v)).

A colorless solid: m.p. 134–135 °C (Et_2O–hexane); ^1H NMR ($CDCl_3$, 400 MHz): δ 7.89–7.83 (m, 2H), 7.69–7.61 (m, 1H), 7.59–7.53 (m, 2H), 7.23 (bs, 2H), 1.33 (s, 18H); $^{13}C\{^1H\}$ NMR ($CDCl_3$, 100 MHz): δ 167.4, 156.9, 134.4, 130.9, 129.5, 120.6, 51.5, 39.2, 28.6. HRMS–ESI $[M + H]^+$ calcd for $C_{18}H_{26}N_3O_2^+$ 316.2020; found 316.2017.

*N,N',3-Triphenyl-2H-azirine-2,2-dicarboxamide (**10c**).* Compound **10c** was prepared following the general procedure F from 5-chloroisoxazole-4-carbonyl chloride **1a** (121 mg, 0.5 mmol), $FeCl_2$ (13 mg, 0.1 mmol, 0.2 equiv), Cs_2CO_3 (652 mg, 2 mmol, 4 equiv) and aniline (93 mg, 1 mmol, 2 equiv) in 96 mg (54% yield), after column chromatography on silica (light petroleum/ethyl acetate, (from 4:1 to 2:1 (v/v)). Compound **10c** was also prepared following the general procedure G from 5-chloroisoxazole-4-carbonyl chloride **1a** (121 mg, 0.5 mmol), $FeCl_2$ (13 mg, 0.1 mmol, 0.2 equiv), Cs_2CO_3 (652 mg, 2 mmol, 4 equiv) and aniline (93 mg, 1 mmol, 2 equiv) in 71 mg (40% yield), after column chromatography on silica (light petroleum/ethyl acetate, (from 4:1 to 2:1 (v/v)).

A pale yellow solid: m.p. 193–194 °C (Et_2O–hexane); ^1H NMR (DMSO-d_6, 400 MHz): δ 10.34 (s, 2H), 8.12–8.02 (m, 2H), 7.83–7.77 (m, 1H), 7.76–7.69 (m, 2H), 7.65–7.58 (m, 4H), 7.36–7.29 (m, 4H), 7.14–7.07 (m, 2H); $^{13}C\{^1H\}$ NMR (DMSO-d_6, 100 MHz): δ 166.6, 155.7, 138.0, 134.7, 131.1, 129.7, 128.6, 124.1, 120.6, 120.5, 40.5. HRMS–ESI $[M + Na]^+$ calcd for $C_{22}H_{17}N_3NaO_2^+$ 378.1213; found 378.1209.

*N,N'-Dicyclopropyl-3-phenyl-2H-azirine-2,2-dicarboxamide (**10d**).* Compound **10d** was prepared following the general procedure F from 5-chloroisoxazole-4-carbonyl chloride **1a** (121 mg, 0.5 mmol), $FeCl_2$ (13 mg, 0.1 mmol, 0.2 equiv), Cs_2CO_3 (652 mg, 2 mmol, 4 equiv) and cyclopropylamine (57 mg, 1 mmol, 2 equiv) in 95 mg (67% yield), after column chromatography

on silica (light petroleum/ethyl acetate, (from 3:1 to 1:1 (v/v)). Compound **10d** was also prepared following the general procedure G from 5-chloroisoxazole-4-carbonyl chloride **1a** (121 mg, 0.5 mmol), $FeCl_2$ (13 mg, 0.1 mmol, 0.2 equiv), Cs_2CO_3 (652 mg, 2 mmol, 4 equiv) and cyclopropylamine (57 mg, 1 mmol, 2 equiv) in 59 mg (42% yield), after column chromatography on silica (light petroleum/ethyl acetate, (from 3:1 to 1:1 (v/v)).

A pale yellow solid: m.p. 133–134 °C (Et_2O–hexane); ^{1}H NMR ($CDCl_3$, 400 MHz): δ 7.88–7.80 (m, 2H), 7.68–7.61 (m, 1H), 7.59–7.51 (m, 2H), 7.43 (s, 2H), 2.77–2.65 (m, 2H), 0.77–0.69 (m, 4H), 0.55–0.45 (m, 4H); ^{13}C{^{1}H} NMR ($CDCl_3$, 100 MHz): δ 169.4, 156.1, 134.6, 131.1, 129.5, 120.0, 38.4, 22.7, 6.3. HRMS–ESI [M + H]$^+$ calcd for $C_{16}H_{18}N_3O_2^+$ 284.1394; found 284.1391.

*N,N,N',N'-Tetraethyl-3-phenyl-2H-azirine-2,2-dicarboxamide (**10e**).* Compound **10e** was prepared following the general procedure F from 5-chloroisoxazole-4-carbonyl chloride **1a** (121 mg, 0.5 mmol), $FeCl_2$ (13 mg, 0.1 mmol, 0.2 equiv), Cs_2CO_3 (652 mg, 2 mmol, 4 equiv) and diethylamine (73 mg, 1 mmol, 2 equiv) in 95 mg (62% yield), after column chromatography on silica (light petroleum/ethyl acetate, (from 3:1 to 1:1 (v/v)). Compound **10e** was also prepared following the general procedure G from 5-chloroisoxazole-4-carbonyl chloride **1a** (121 mg, 0.5 mmol), $FeCl_2$ (13 mg, 0.1 mmol, 0.2 equiv), Cs_2CO_3 (652 mg, 2 mmol, 4 equiv) and diethylamine (73 mg, 1 mmol, 2 equiv) in 109 mg (69% yield), after column chromatography on silica (light petroleum/ethyl acetate, (from 3:1 to 1:1 (v/v)).

A colorless oil: ^{1}H NMR ($CDCl_3$, 400 MHz): δ 8.07–7.96 (m, 2H), 7.64–7.56 (m, 1H), 7.56–7.48 (m, 2H), 4.00–3.72 (m, 4H), 3.49–3.24 (m, 4H), 1.20 (t, J = 7.1 Hz, 6H), 1.11 (t, J = 7.1 Hz, 6H); ^{13}C{^{1}H} NMR ($CDCl_3$, 100 MHz): δ 167.6, 161.3, 133.5, 130.3, 129.1, 123.0, 43.8, 41.9, 39.9, 13.8, 12.4. HRMS–ESI [M + H]$^+$ calcd for $C_{18}H_{26}N_3O_2^+$ 316.2020; found 316.2028.

*(3-Phenyl-2H-azirine-2,2-diyl)bis(pyrrolidin-1-ylmethanone) (**10f**).* Compound **10f** was prepared following the general procedure F from 5-chloroisoxazole-4-carbonyl chloride **1a** (121 mg, 0.5 mmol), $FeCl_2$ (13 mg, 0.1 mmol, 0.2 equiv), Cs_2CO_3 (652 mg, 2 mmol, 4 equiv) and pyrrolidine (71 mg, 1 mmol, 2 equiv) in 62 mg (40% yield), after column chromatography on silica (light

petroleum/ethyl acetate, (from 1:1 to 0:1 (v/v)). Compound **10f** was also prepared following the general procedure G from 5-chloroisoxazole-4-carbonyl chloride **1a** (121 mg, 0.5 mmol), $FeCl_2$ (13 mg, 0.1 mmol, 0.2 equiv), Cs_2CO_3 (652 mg, 2 mmol, 4 equiv) and pyrrolidine (71 mg, 1 mmol, 2 equiv) in 121 mg (78% yield), after column chromatography on silica (light petroleum/ethyl acetate, (from 1:1 to 0:1 (v/v)).

A colorless solid: m.p. 136–137 °C (Et_2O–hexane); ^1H NMR ($CDCl_3$, 400 MHz): δ 8.14–7.88 (m, 2H), 7.72–7.40 (m, 3H), 3.90–3.61 (m, 4H), 3.61–3.31 (m, 4H), 2.08–1.77 (m, 8H); $^{13}C\{^1H\}$ NMR ($CDCl_3$, 100 MHz): δ 166.4, 160.5, 133.6, 130.6, 129.1, 122.6, 46.7, 46.5, 43.7, 26.3, 23.8. HRMS–ESI $[M + H]^+$ calcd for $C_{18}H_{22}N_3O_2^+$ 312.1707; found 312.1710.

*(3-Phenyl-2H-azirine-2,2-diyl)bis((4-methylpiperidin-1-yl)methanone) (**10g**).* Compound **10g** was prepared following the general procedure F from 5-chloroisoxazole-4-carbonyl chloride **1a** (121 mg, 0.5 mmol), $FeCl_2$ (13 mg, 0.1 mmol, 0.2 equiv), Cs_2CO_3 (652 mg, 2 mmol, 4 equiv) and 4-methylpiperidine (99 mg, 1 mmol, 2 equiv) in 129 mg (70% yield), after column chromatography on silica (light petroleum/ethyl acetate, (from 3:1 to 2:1 (v/v)). Compound **10g** was also prepared following the general procedure G from 5-chloroisoxazole-4-carbonyl chloride **1a** (121 mg, 0.5 mmol), $FeCl_2$ (13 mg, 0.1 mmol, 0.2 equiv), Cs_2CO_3 (652 mg, 2 mmol, 4 equiv) and 4-methylpiperidine (99 mg, 1 mmol, 2 equiv) in 107 mg (58% yield), after column chromatography on silica (light petroleum/ethyl acetate, (from 3:1 to 2:1 (v/v)).

A colorless solid: m.p. 137–138 °C (Et_2O–hexane); ^1H NMR ($CDCl_3$, 400 MHz): δ 8.07–7.91 (m, 2H), 7.66–7.44 (m, 3H), 4.87–4.39 (m, 4H), 3.25–2.96 (m, 2H), 2.77–2.54 (m, 2H), 1.82–1.59 (m, 6H), 1.26–0.99 (m, 4H), 0.94 (s, 3H), 0.93 (s, 3H); $^{13}C\{^1H\}$ NMR ($CDCl_3$, 100 MHz): δ 166.8, 133.6, 130.3, 129.1, 122.8, 46.5, 43.3, 43.1, 42.9, 34.5, 33.7, 31.0, 21.7. HRMS–ESI $[M + H]^+$ calcd for $C_{22}H_{30}N_3O_2^+$ 368.2333; found 368.2330.

*(3-Phenyl-2H-azirine-2,2-diyl)bis(morpholinomethanone) (**10h**).* Compound **10h** was prepared following the general procedure F from 5-chloroisoxazole-4-carbonyl chloride **1a** (121 mg, 0.5 mmol), $FeCl_2$ (13 mg, 0.1 mmol, 0.2 equiv), Cs_2CO_3 (652 mg, 2 mmol, 4 equiv) and

morpholine (87 mg, 1 mmol, 2 equiv) in 110 mg (64% yield), after column chromatography on silica (light petroleum/ethyl acetate, (from 1:1 to 0:1 (v/v)). Compound **10h** was also prepared following the general procedure G from 5-chloroisoxazole-4-carbonyl chloride **1a** (121 mg, 0.5 mmol), $FeCl_2$ (13 mg, 0.1 mmol, 0.2 equiv), Cs_2CO_3 (652 mg, 2 mmol, 4 equiv) and morpholine (87 mg, 1 mmol, 2 equiv) in 94 mg (55% yield), after column chromatography on silica (light petroleum/ethyl acetate, (from 1:1 to 0:1 (v/v)).

A colorless solid: m.p. 194–195 °C (Et_2O–hexane); ^1H NMR ($CDCl_3$, 400 MHz): δ 8.03–7.92 (m, 2H), 7.69–7.60 (m, 1H), 7.60–7.52 (m, 2H), 4.17–3.50 (m, 16H); $^{13}C\{^1H\}$ NMR ($CDCl_3$, 100 MHz): δ 166.8, 160.6, 134.1, 130.3, 129.4, 122.3, 66.8 (2C), 46.7, 42.9, 42.4. HRMS–ESI [M + Na]$^+$ calcd for $C_{18}H_{21}N_3NaO_4^+$ 366.1425; found 366.1421.

General procedure H for the synthesis of dialkyl 2*H*-azirine-2,2-dicarboxylates 11.

Anhydrous $FeCl_2$ (26 mg, 0.2 mmol, 0.2 equiv) was added to a solution of 5-chloroisoxazole-4-carbonyl chloride **1** (1 mmol) in acetonitrile (10 mL) under Ar atmosphere. The mixture was stirred at room temperature for 2 h. Then, alcohol (20 mL) was added and the mixture was stirred at room temperature for 1 h. The reaction mixture was treated with water (50 mL) and extracted with EtOAc (3 × 25 mL). The combined organic layers were washed with brine (50 mL) and dried over Na_2SO_4. After evaporation of the solvent under reduced pressure, the residue was purified by column chromatography on silica gel (eluent EtOAc/light petroleum) to give 2*H*-azirine-2,2-dicarboxylate **11**.

*Dimethyl 3-phenyl-2H-azirine-2,2-dicarboxylate **(11a)*** [9]. Compound **11a** was prepared following the general procedure H from 5-chloroisoxazole-4-carbonyl chloride **1a** (242 mg, 1 mmol), $FeCl_2$ (26 mg, 0.2 mmol, 0.2 equiv) and MeOH (20 mL) in 219 mg (94% yield), after column chromatography on silica (light petroleum/ethyl acetate, (from 10:1 to 2:1 (v/v)) as a pale yellow solid: m.p. 90–91 °C (Et_2O/hexane); ^1H NMR ($CDCl_3$, 400 MHz): δ 8.01–7.84 (m, 2H), 7.72–7.64 (m, 1H), 7.64–7.54 (m, 2H), 3.77 (s, 6H); $^{13}C\{^1H\}$ NMR ($CDCl_3$, 100 MHz): δ 167.5,

155.2, 134.6, 131.0, 129.5, 120.2, 52.9, 38.6. HRMS–ESI $[M + Na]^+$ calcd for $C_{12}H_{11}NNaO_4^+$ 256.0581; found 256.0586.

Scale up experiment. Anhydrous $FeCl_2$ (102 mg, 0.8 mmol, 0.2 equiv) was added to a solution of 5-chloroisoxazole-4-carbonyl chloride **1a** (968 mg, 4 mmol) in acetonitrile (40 mL) under Ar atmosphere. The mixture was stirred at room temperature for 2 h. Then, MeOH (80 mL) was added and the mixture was stirred at room temperature for 1 h. The reaction mixture was treated with water (500 mL) and extracted with EtOAc (3 × 100 mL). The combined organic layers were washed with brine (300 mL) and dried over Na_2SO_4. After evaporation of the solvent under reduced pressure, the residue was purified by column chromatography on silica gel (eluent EtOAc/light petroleum from 10:1 to 2:1 (v/v)) to give 2*H*-azirine-2,2-dicarboxylate **11a** (924 mg, 99%).

*Diethyl 3-phenyl-2H-azirine-2,2-dicarboxylate (**11b**).* Compound **11b** was prepared following the general procedure H from 5-chloroisoxazole-4-carbonyl chloride **1a** (242 mg, 1 mmol), $FeCl_2$ (26 mg, 0.2 mmol, 0.2 equiv) and EtOH (20 mL) in 199 mg (76% yield), after column chromatography on silica (light petroleum/ethyl acetate, (from 10:1 to 5:1 (v/v)) as a pale yellow oil: ^{1}H NMR ($CDCl_3$, 400 MHz): δ 7.97–7.84 (m, 2H), 7.71–7.63 (m, 1H), 7.63–7.54 (m, 2H), 4.23 (q, J = 7.1 Hz, 4H), 1.25 (t, J = 7.1 Hz, 6H); $^{13}C\{^1H\}$ NMR ($CDCl_3$, 100 MHz): δ 167.2, 155.4, 134.4, 130.9, 129.4, 120.4, 61.9, 38.9, 14.0. HRMS–ESI $[M + H]^+$ calcd for $C_{14}H_{16}NO_4^+$ 262.1074; found 262.1070.

*Dibenzyl 3-phenyl-2H-azirine-2,2-dicarboxylate (**11c**).* To a solution of the 2*H*-azirine-2,2-dicarboxylic acid **6a** (62 mg, 0.3 mmol) in DCM (2 mL) was added DIPEA (77 mg, 0.6 mmol, 2 equiv). The mixture was cooled to 0 °C and treated with EDC (93 mg, 0.6 mmol, 2 equiv), HOBt (81 mg, 0.6 mmol, 2 equiv), and phenylmethanol (162 mg, 1.5 mmol, 5 equiv). The reaction was stirred at room temperature for 12 h. The reaction mixture was treated with water (20 mL) and extracted with EtOAc (3 × 10 mL). The combined organic layers were washed with brine (20 mL) and dried over Na_2SO_4. After evaporation of the solvent under reduced pressure, the residue was

purified by column chromatography on silica gel (eluent EtOAc/light petroleum, from 1:10 to 1:5 (v/v)) to give 2*H*-azirine-2,2-dicarboxylate **11c** in 27 mg (23% yield) as a colorless oil; ^{1}H NMR ($CDCl_3$, 400 MHz): δ 7.94–7.85 (m, 2H), 7.72–7.63 (m, 1H), 7.61–7.52 (m, 2H), 7.35–7.22 (m, 10H), 5.26–5.17 (m, 4H); $^{13}C\{^{1}H\}$ NMR ($CDCl_3$, 100 MHz): δ 167.0, 155.1, 135.2, 134.5, 131.0, 129.4, 128.5, 128.2, 127.9, 120.1, 67.5, 39.0. HRMS–ESI $[M + H]^+$ calcd for $C_{24}H_{20}NO_4^+$ 386.1387; found 386.1379.

*Bis(2-methoxy-2-oxo-1-phenylethyl) 3-phenyl-2H-azirine-2,2-dicarboxylate (**11d**).* A solution of 2*H*-azirine-2,2-dicarboxylic acid **6a** (51 mg, 0.25 mmol) and methyl 2-diazo-2-phenylacetate (106 mg, 0.6 mmol, 2.4 equiv) in DCM (5 mL) in a screw cap tube was placed at a distance of 5 cm from the irradiation source (EvoluChem PhotoRedOx Box™, 450 nm, 30 W). The reaction mixture was irradiated for 1.5 h. After evaporation of the solvent under reduced pressure, the residue was purified by column chromatography on silica gel (eluent EtOAc/light petroleum, from 1:10 to 1:3 (v/v)) to give 2*H*-azirine-2,2-dicarboxylate **11d** in 108 mg (86% yield) as a colorless oil (mixture of diastereomers): ^{1}H NMR ($CDCl_3$, 400 MHz): δ 8.15–7.92 (m, 2H), 7.75–7.57 (m, 3H), 7.49–7.21 (m, 10H), 6.15–5.80 (m, 2H), 3.76–3.60 (m, 6H); $^{13}C\{^{1}H\}$ NMR ($CDCl_3$, 100 MHz): δ 168.4, 168.38, 168.3, 166.5, 166.3, 166.1, 166.0, 154.5, 154.4, 154.0, 134.6, 133.04, 133.02, 132.99, 132.9, 131.3, 131.23, 131.21, 129.5, 129.2, 129.1, 128.63, 128.61, 127.5, 127.3 (2C), 127.27, 119.72, 119.67, 119.6, 75.6, 75.5, 75.3, 52.6, 52.59, 52.55, 38.7, 38.4, 38.1. HRMS–ESI $[M + Na]^+$ calcd for $C_{28}H_{23}NNaO_8^+$ 524.1316; found 524.1324.

*3-Phenyl-2H-azirine-2,2-dicarbonyl diazide (**12**).* Anhydrous $FeCl_2$ (26 mg, 0.2 mmol, 0.2 equiv) was added to a solution of 5-chloroisoxazole-4-carbonyl chloride **1a** (242 mg, 1 mmol) in acetonitrile (4 mL) under Ar atmosphere. The mixture was stirred at room temperature for 2 h. Then, reaction mixture was cooled to 0 °C, sodium azide (208 mg, 3.2 mmol, 3.2 equiv) was added, and the resulting mixture was stirred for additional 10 min at 0 °C and at room temperature for 1 h. The reaction mixture was filtered through a pad of Celite. After evaporation of the solvent under reduced pressure, the residue was purified by column chromatography on silica gel (eluent

EtOAc/light petroleum, from 1:5 (v/v)) to give 2*H*-azirine-2,2-dicarbonyl diazide **12** in 217 mg (85% yield) as a colorless solid: m.p. 53–54 °C (Et_2O/hexane); ^{1}H NMR ($CDCl_3$, 400 MHz): δ 7.99–7.82 (m, 2H), 7.79–7.69 (m, 1H), 7.69–7.57 (m, 2H); $^{13}C\{^{1}H\}$ NMR (C_6D_6, 100 MHz): δ 176.8, 161.5, 134.5, 130.4, 129.5, 120.4, 50.7. HRMS–ESI $[M + Na]^+$ calcd for $C_{10}H_5N_7NaO_2^+$ 278.0397; found 278.0394.

Caution! *Although diazide* ***12*** *was found to be safe in our hands, it is potentially explosive and should be handled with care.*

2. NMR spectra

^{1}H NMR spectrum (400 MHz, $CDCl_3$) of compound **4a**

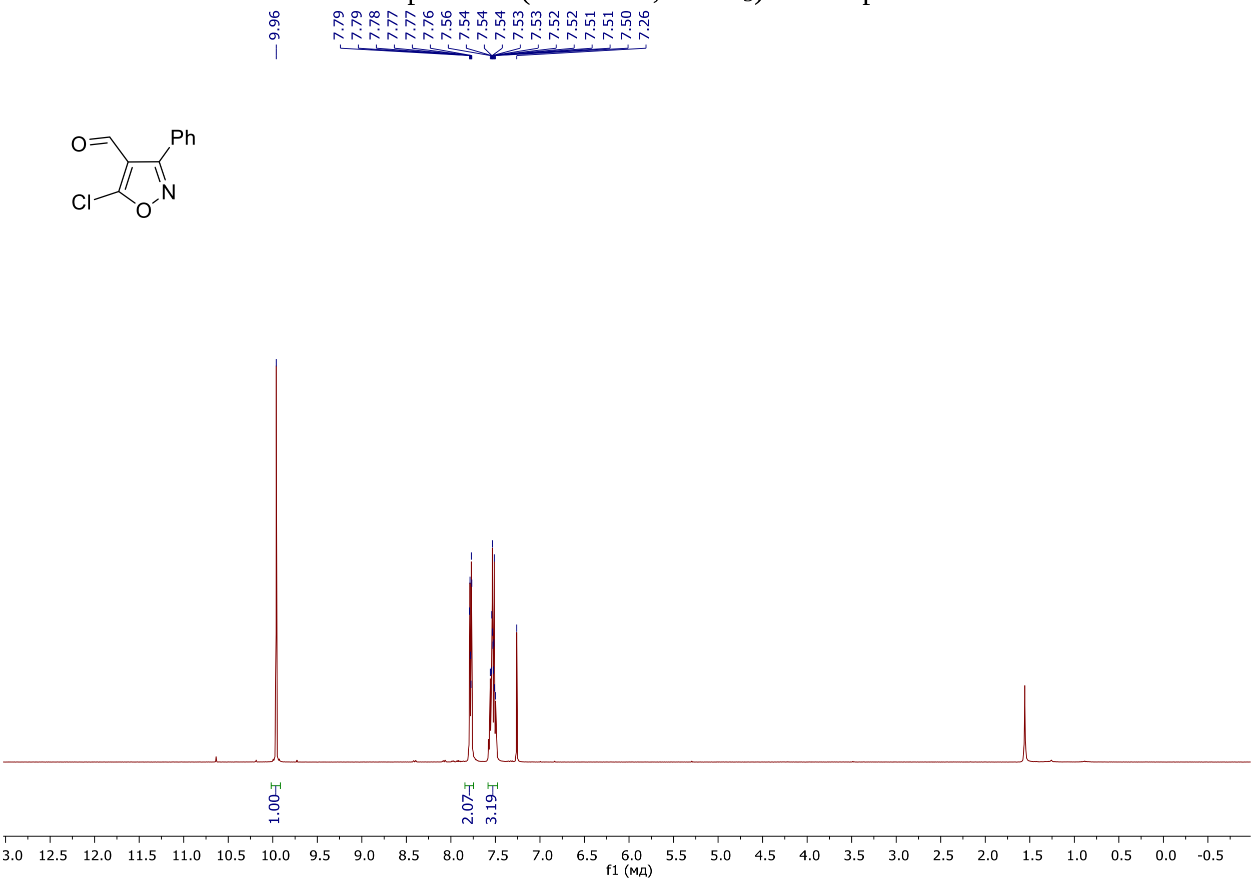

$^{13}C\{^1H\}$ NMR spectrum (100 MHz, $CDCl_3$) of compound **4a**

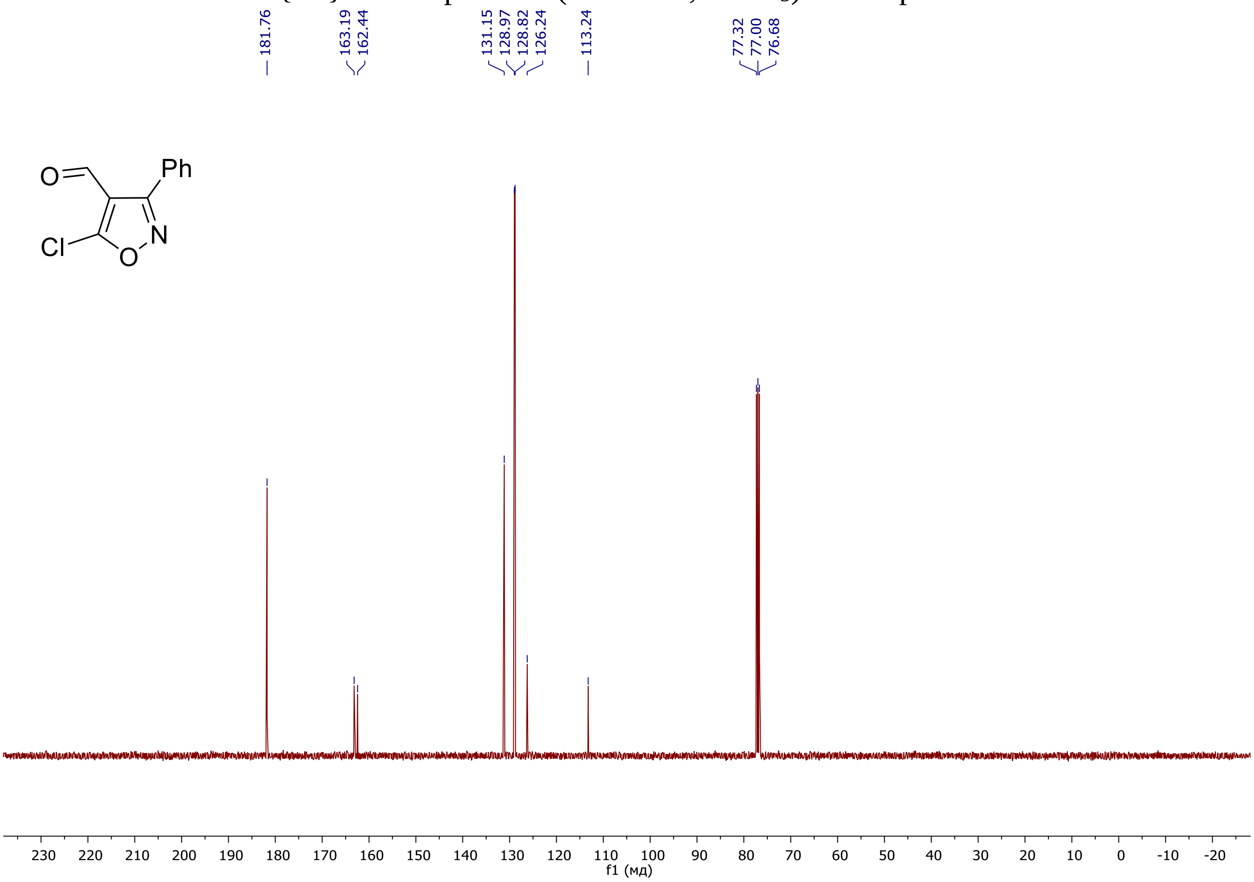

^{1}H NMR spectrum (400 MHz, $CDCl_3$) of compound **4b**

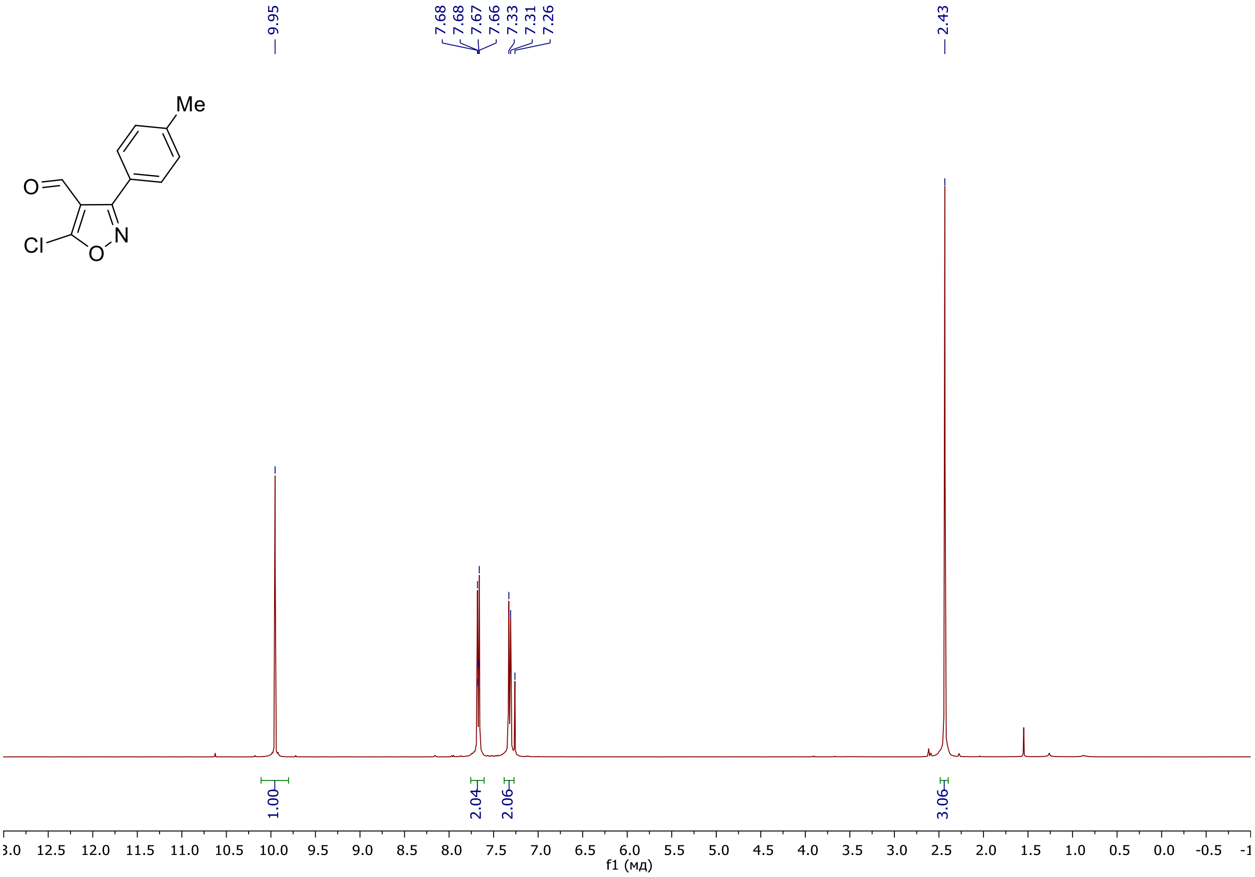

$^{13}C\{^{1}H\}$ NMR spectrum (100 MHz, $CDCl_3$) of compound **4b**

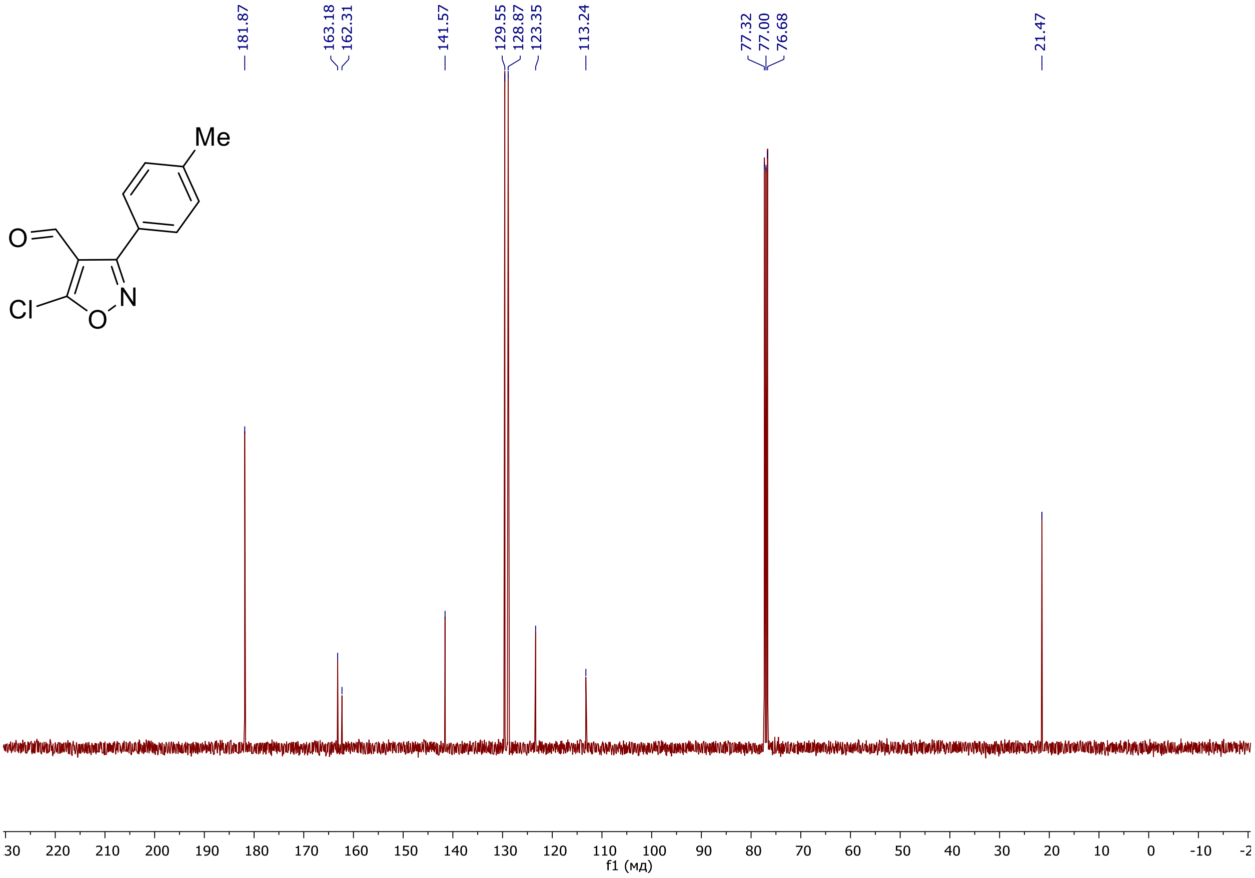

^{1}H NMR spectrum (400 MHz, $CDCl_3$) of compound **4c**

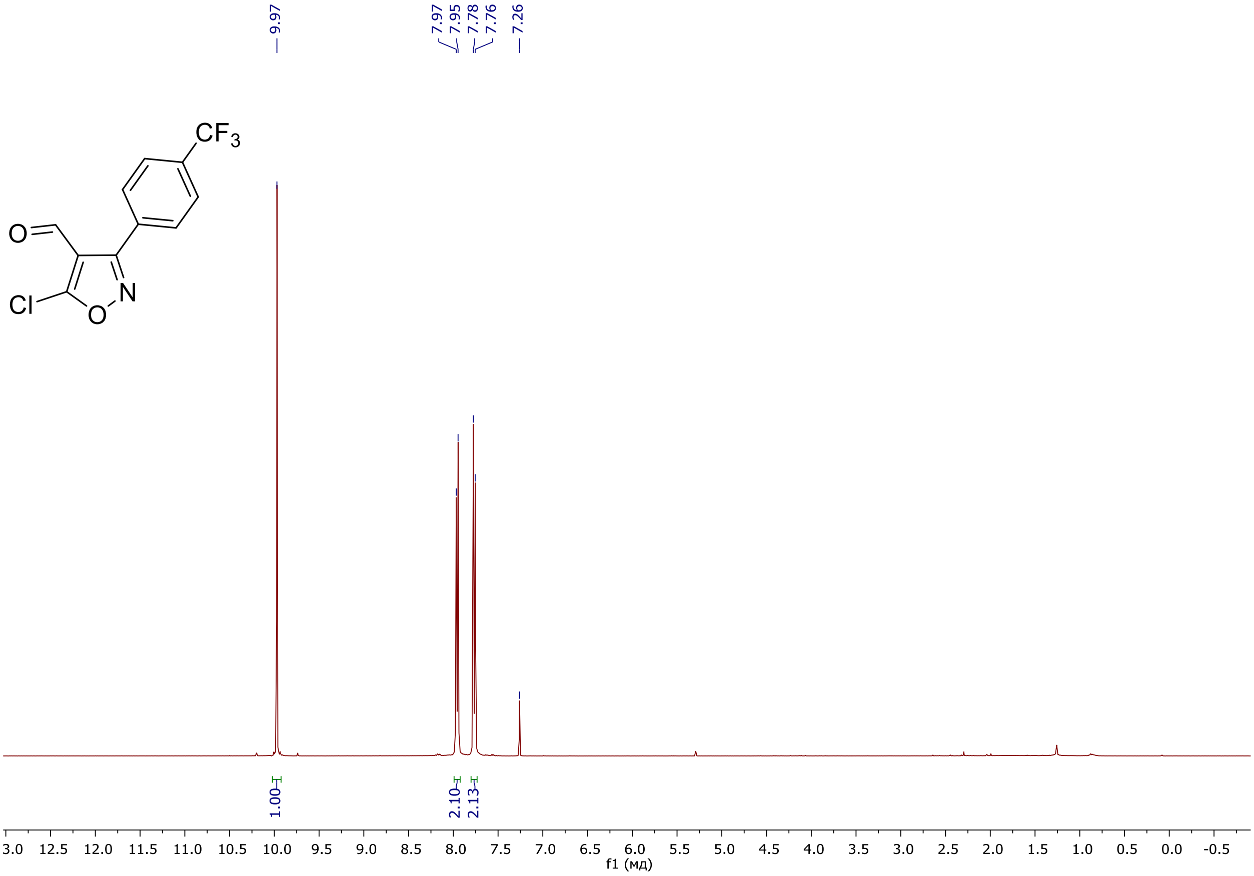

$^{13}C\{^{1}H\}$ NMR spectrum (100 MHz, $CDCl_3$) of compound **4c**

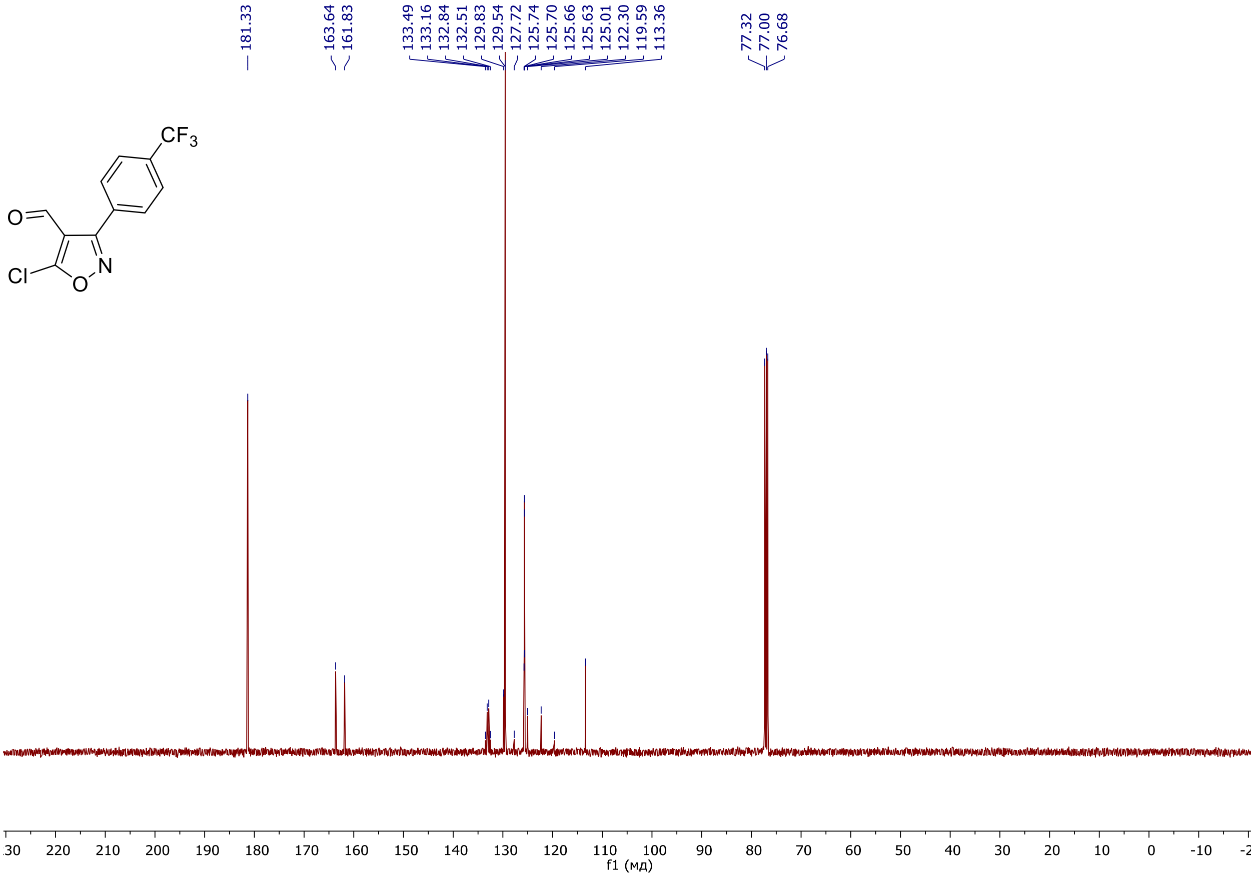

$^{19}F\{^1H\}$ NMR spectrum (376 MHz, $CDCl_3$) of compound **4c**

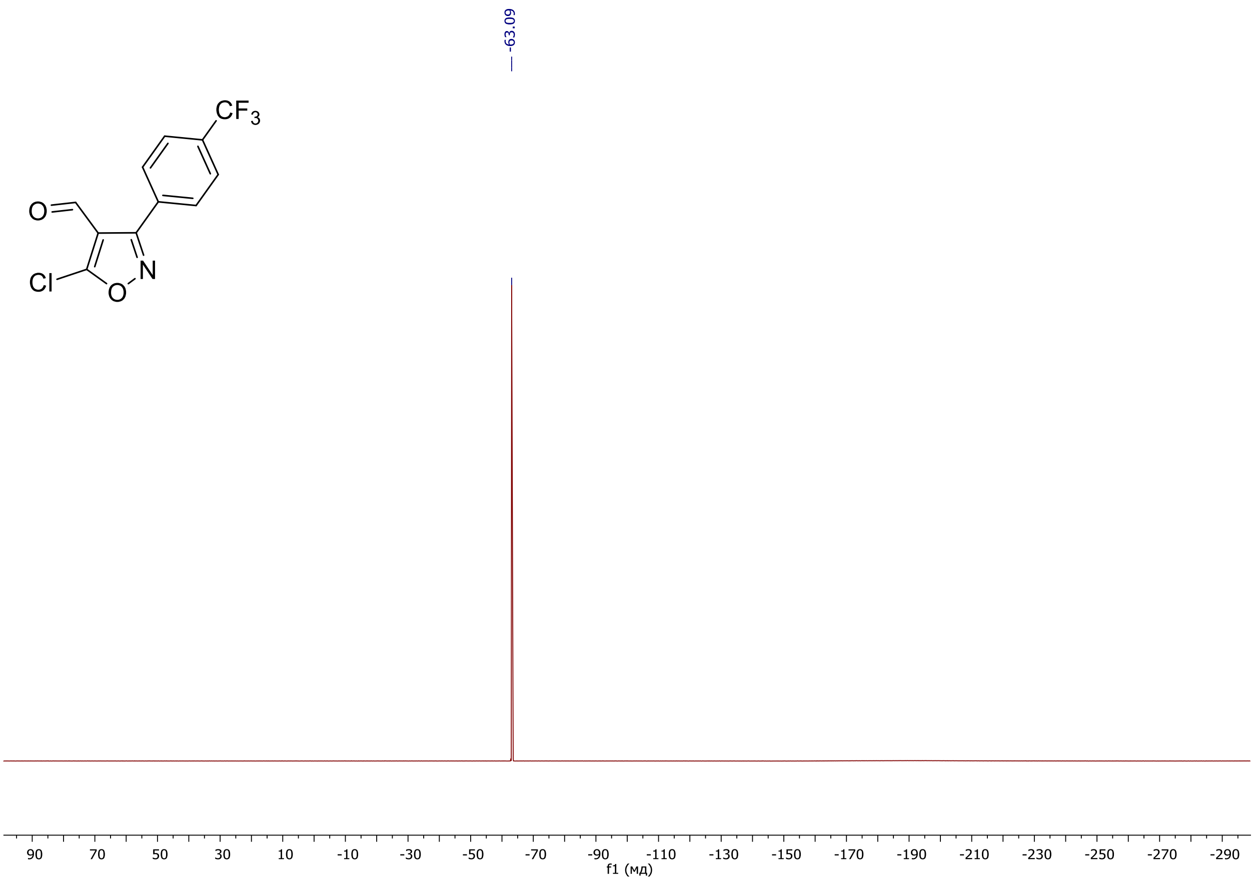

^{1}H NMR spectrum (400 MHz, $CDCl_3$) of compound **4d**

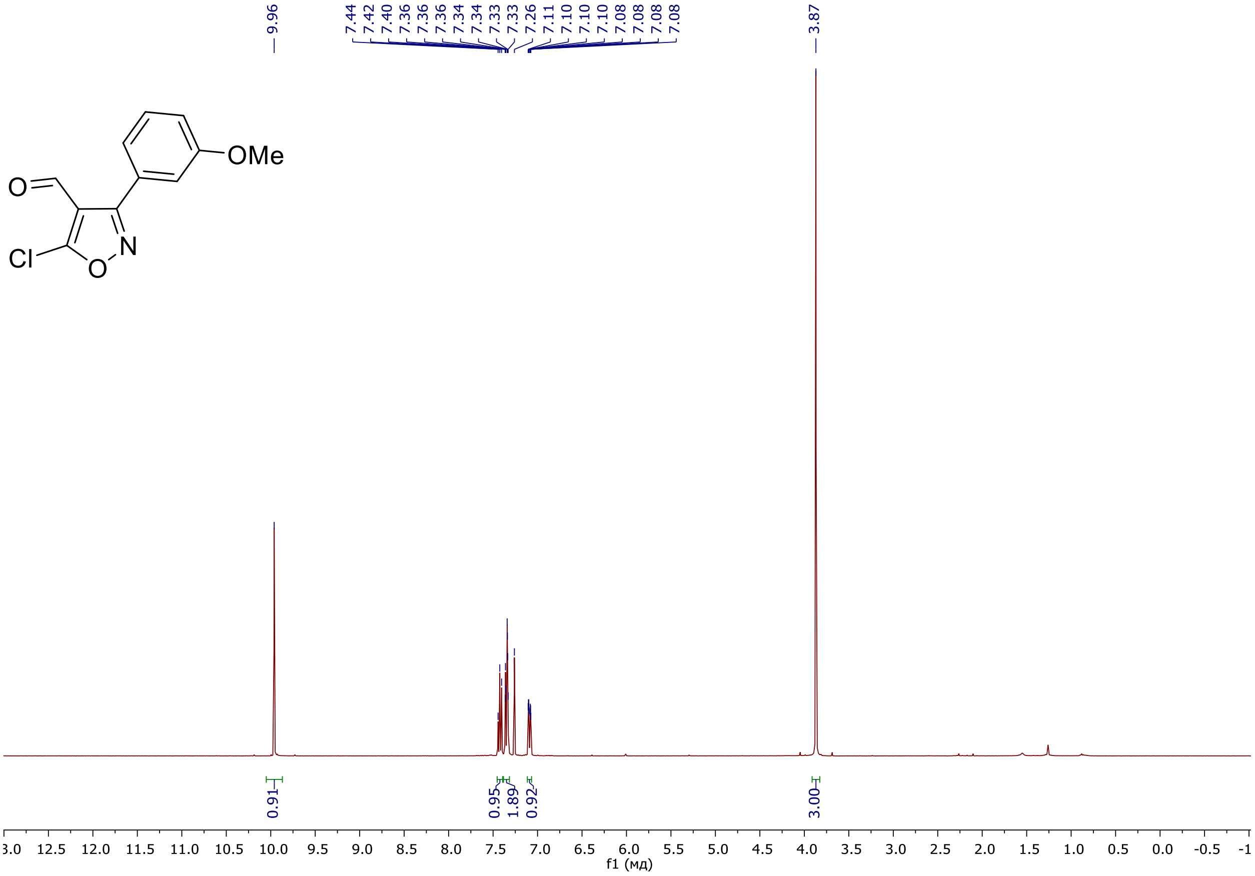

^{13}C{^{1}H} NMR spectrum (100 MHz, $CDCl_3$) of compound **4d**

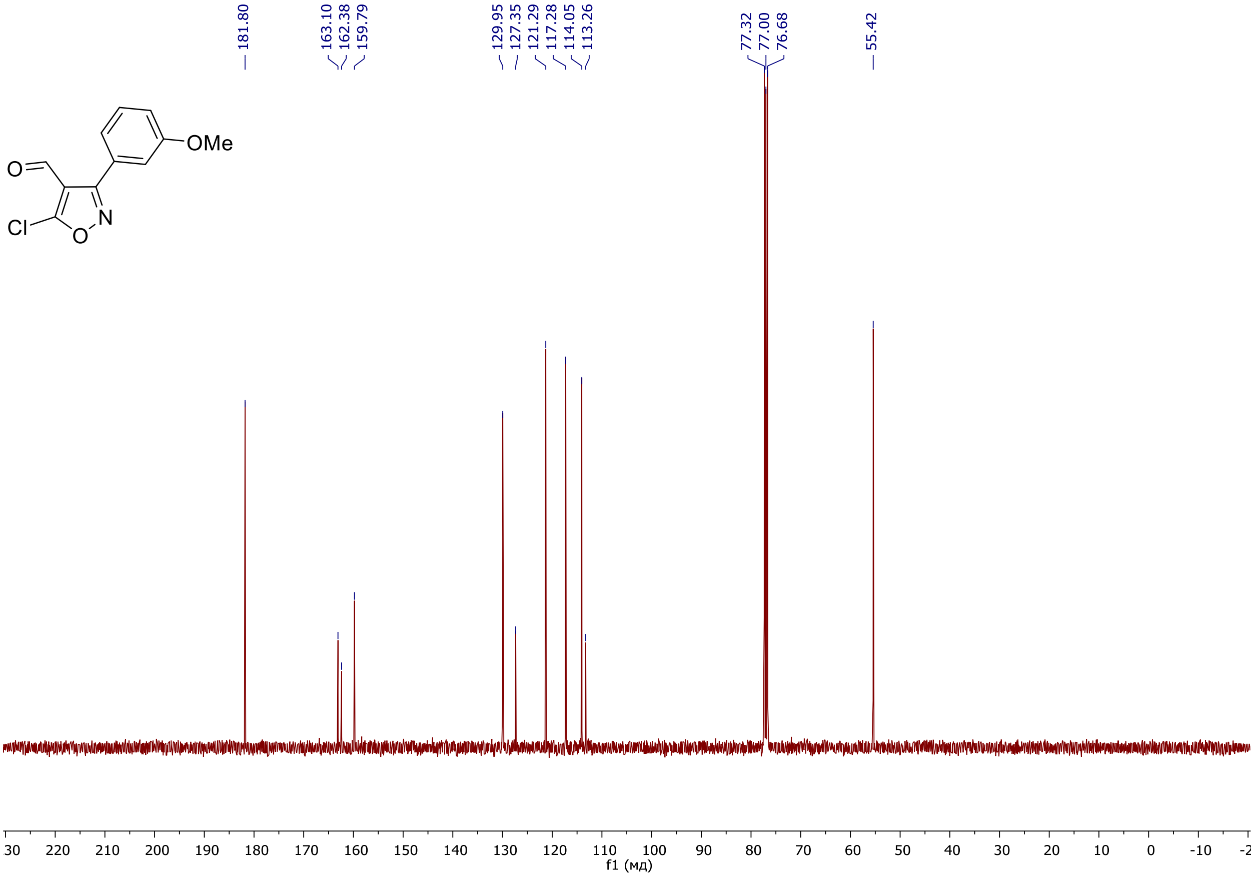

^{1}H NMR spectrum (400 MHz, $CDCl_3$) of compound **4e**

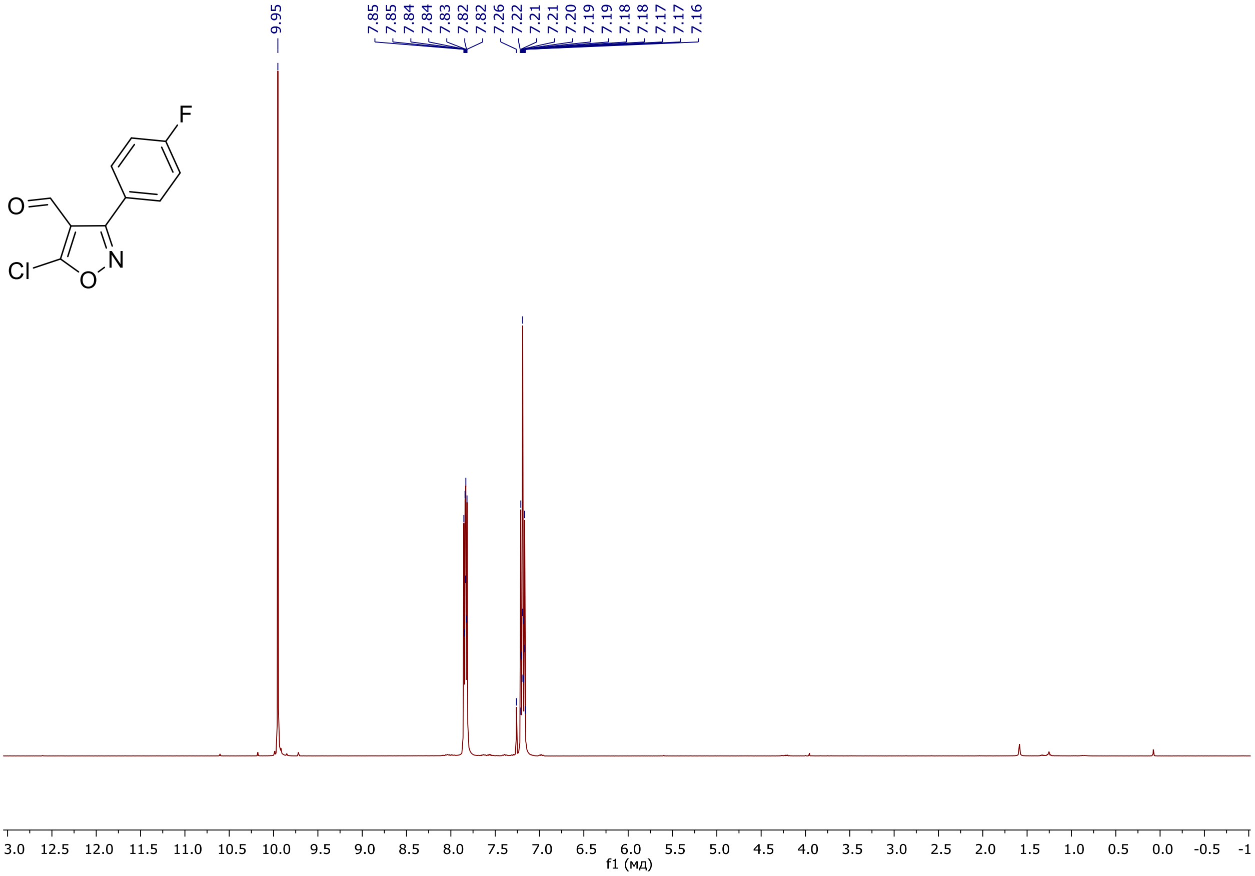

$^{13}C\{^{1}H\}$ NMR spectrum (100 MHz, $CDCl_3$) of compound **4e**

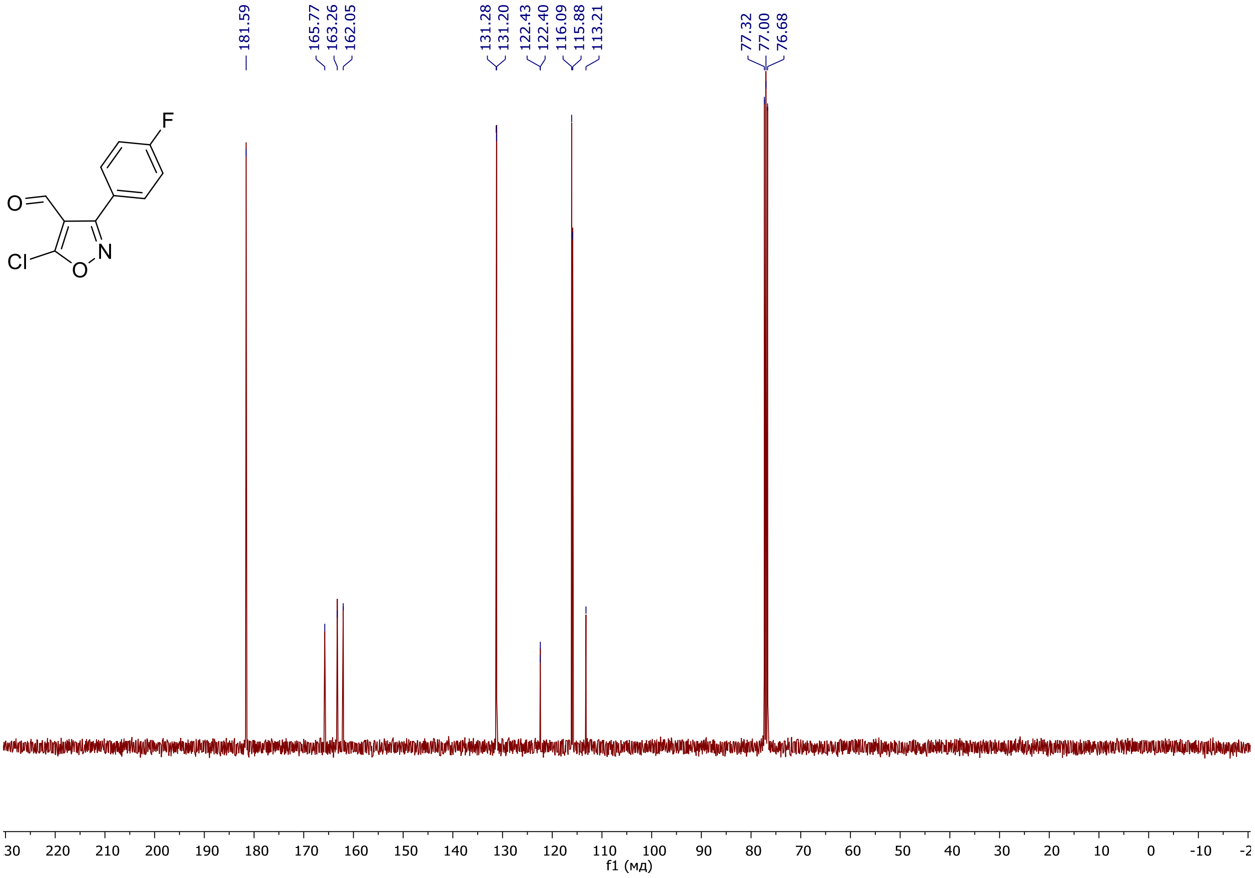

$^{19}F\{^{1}H\}$ NMR spectrum (376 MHz, $CDCl_3$) of compound **4e**

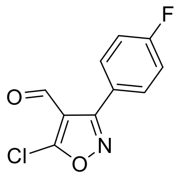

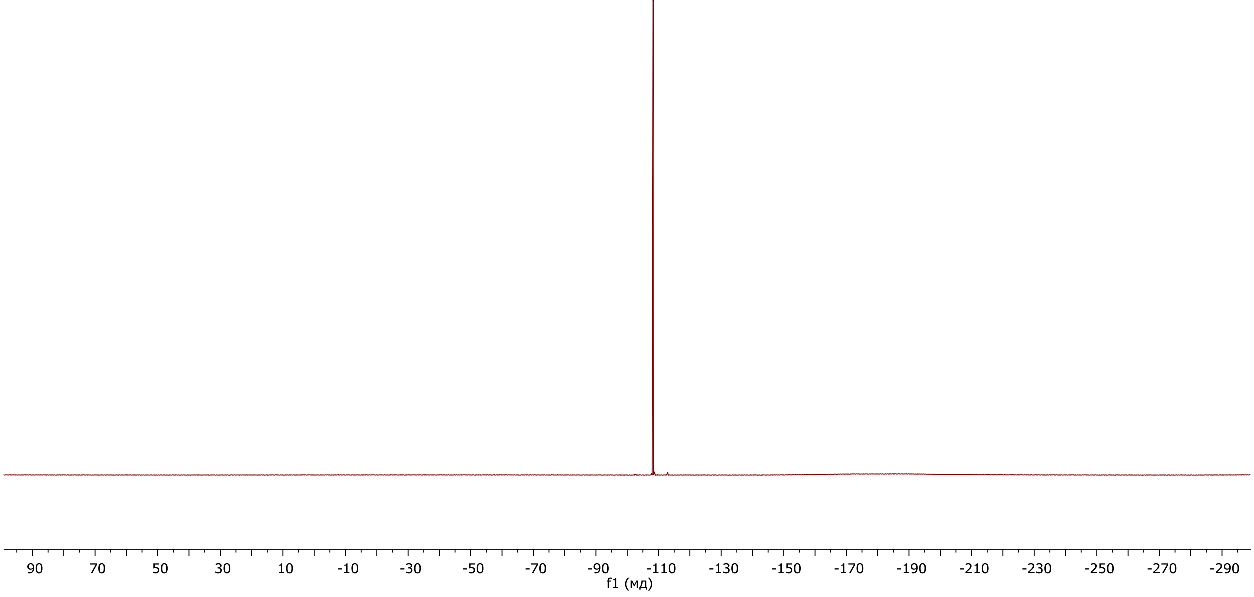

^{1}H NMR spectrum (400 MHz, $CDCl_3$) of compound **4f**

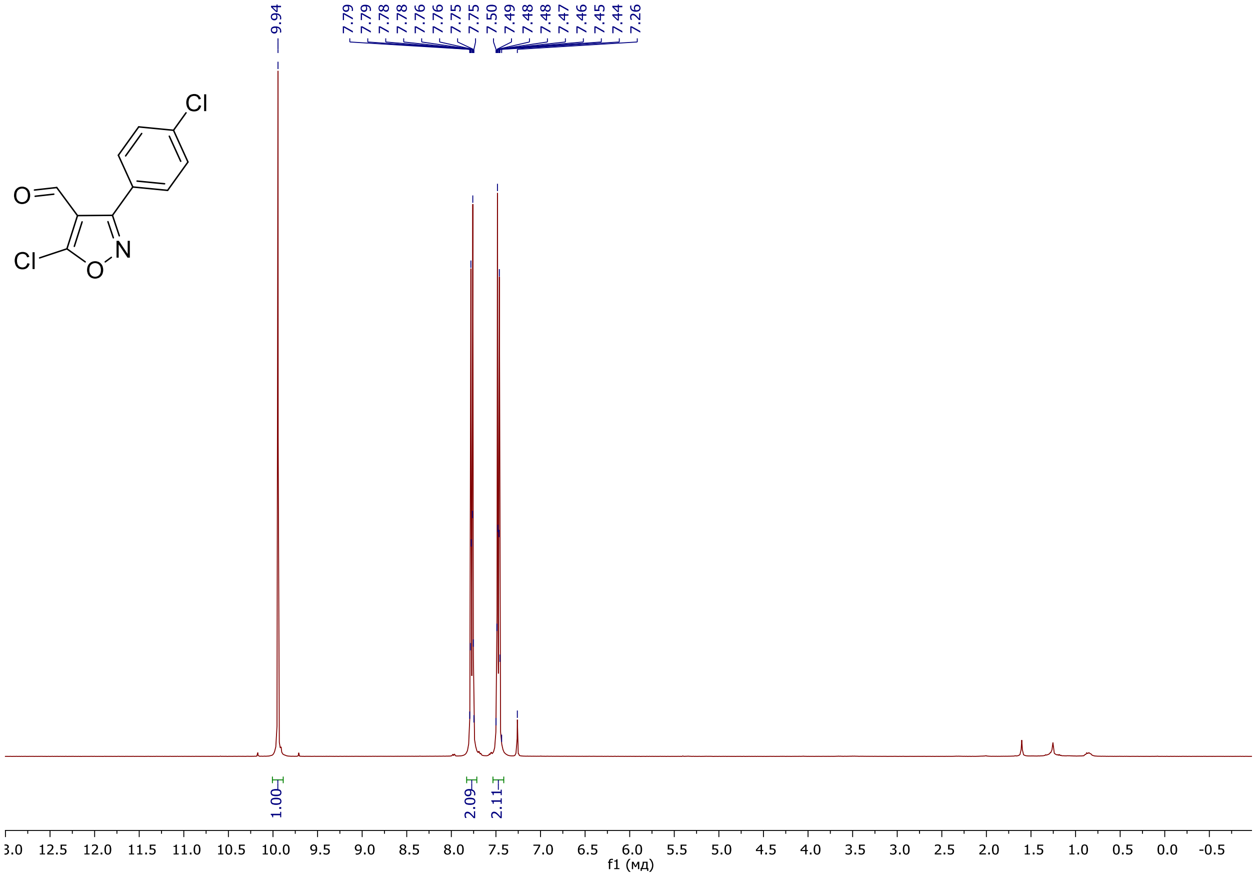

^{13}C{^{1}H} NMR spectrum (100 MHz, $CDCl_3$) of compound **4f**

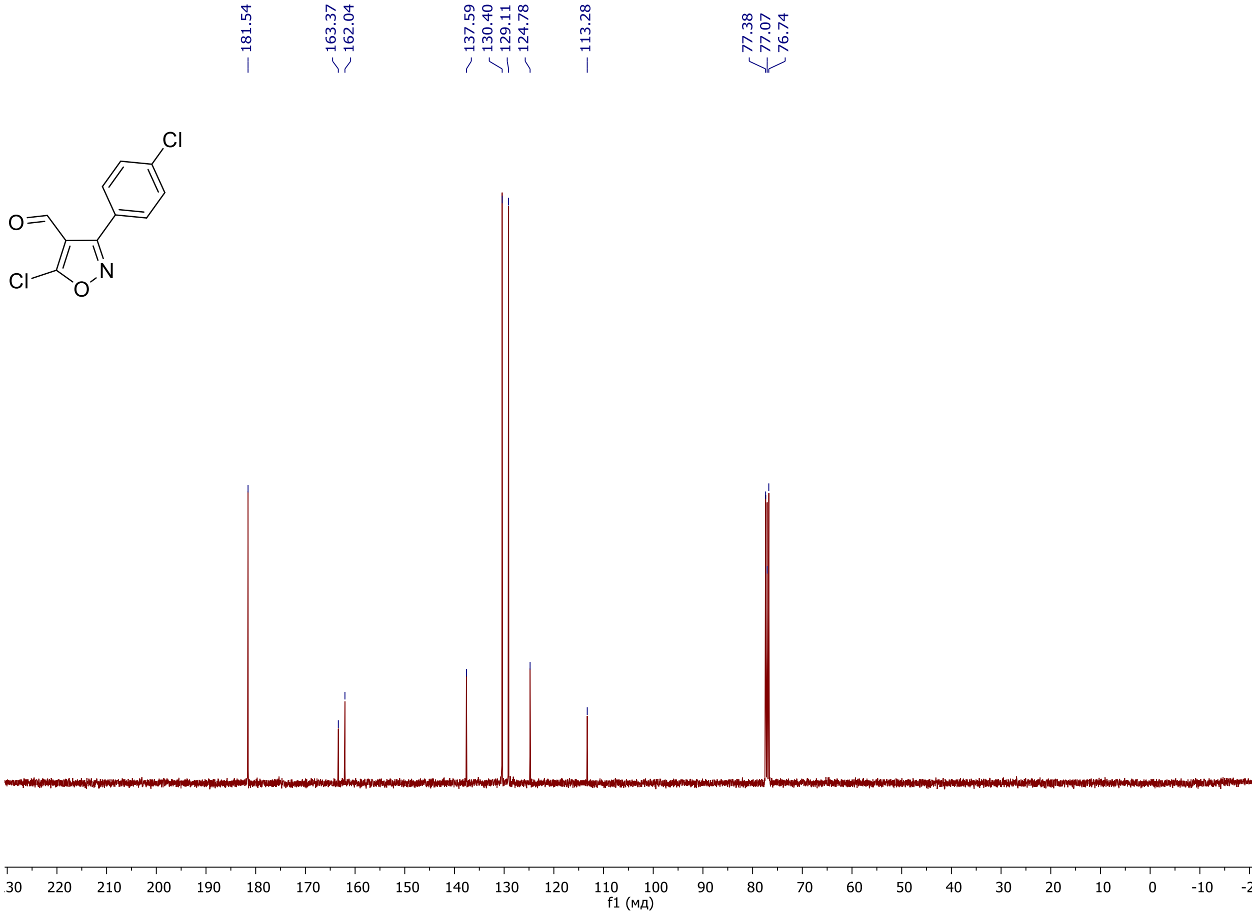

^{1}H NMR spectrum (400 MHz, $CDCl_3$) of compound **4g**

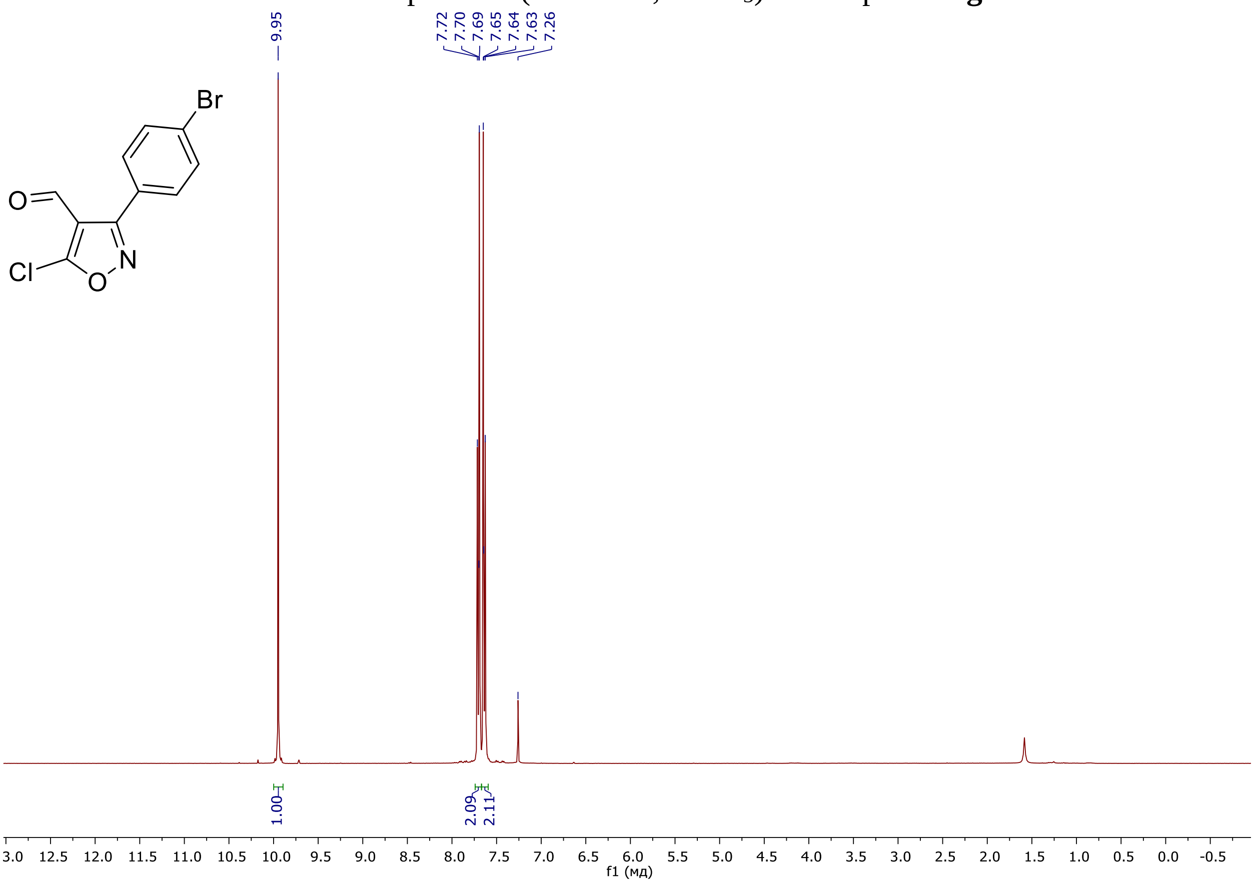

$^{13}C\{^{1}H\}$ NMR spectrum (100 MHz, $CDCl_3$) of compound **4g**

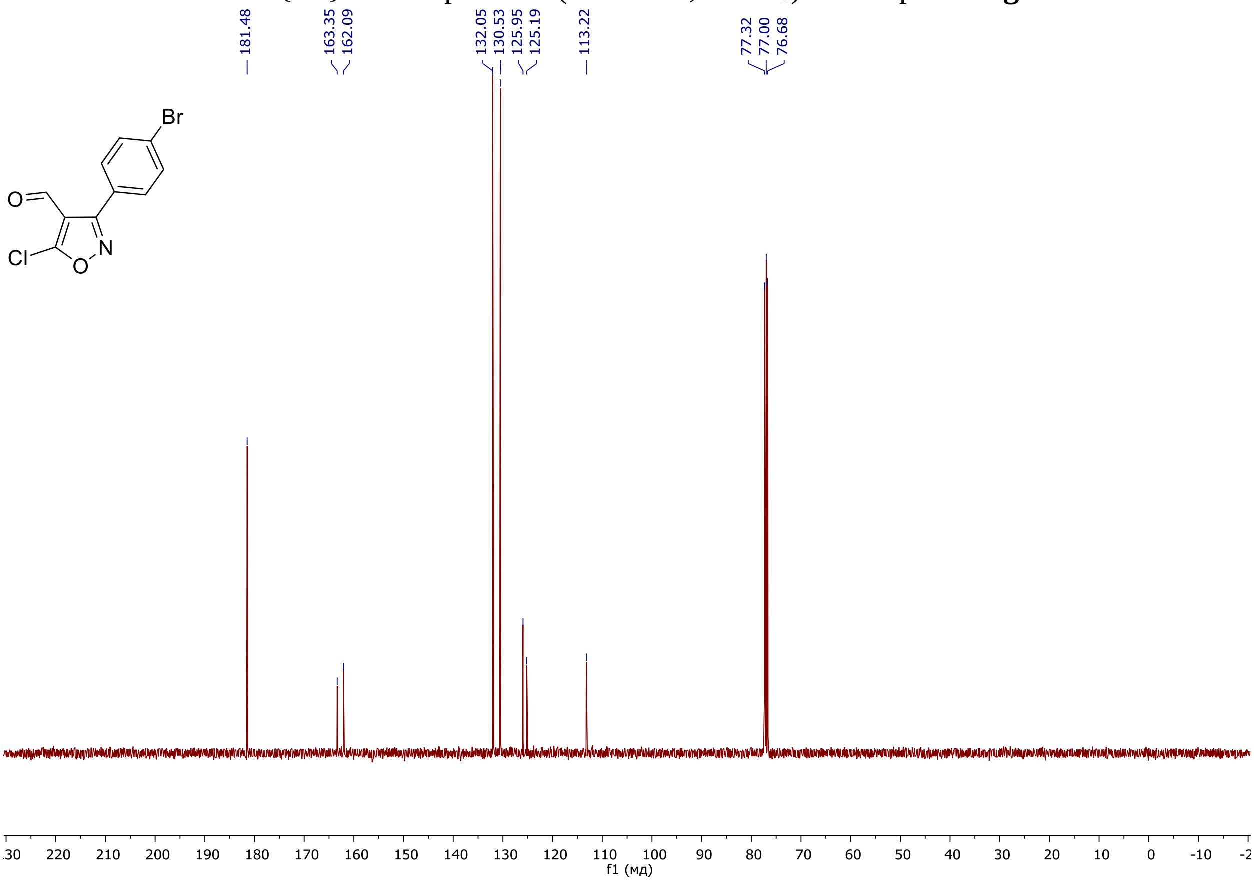

^{1}H NMR spectrum (400 MHz, $CDCl_3$) of compound **4h**

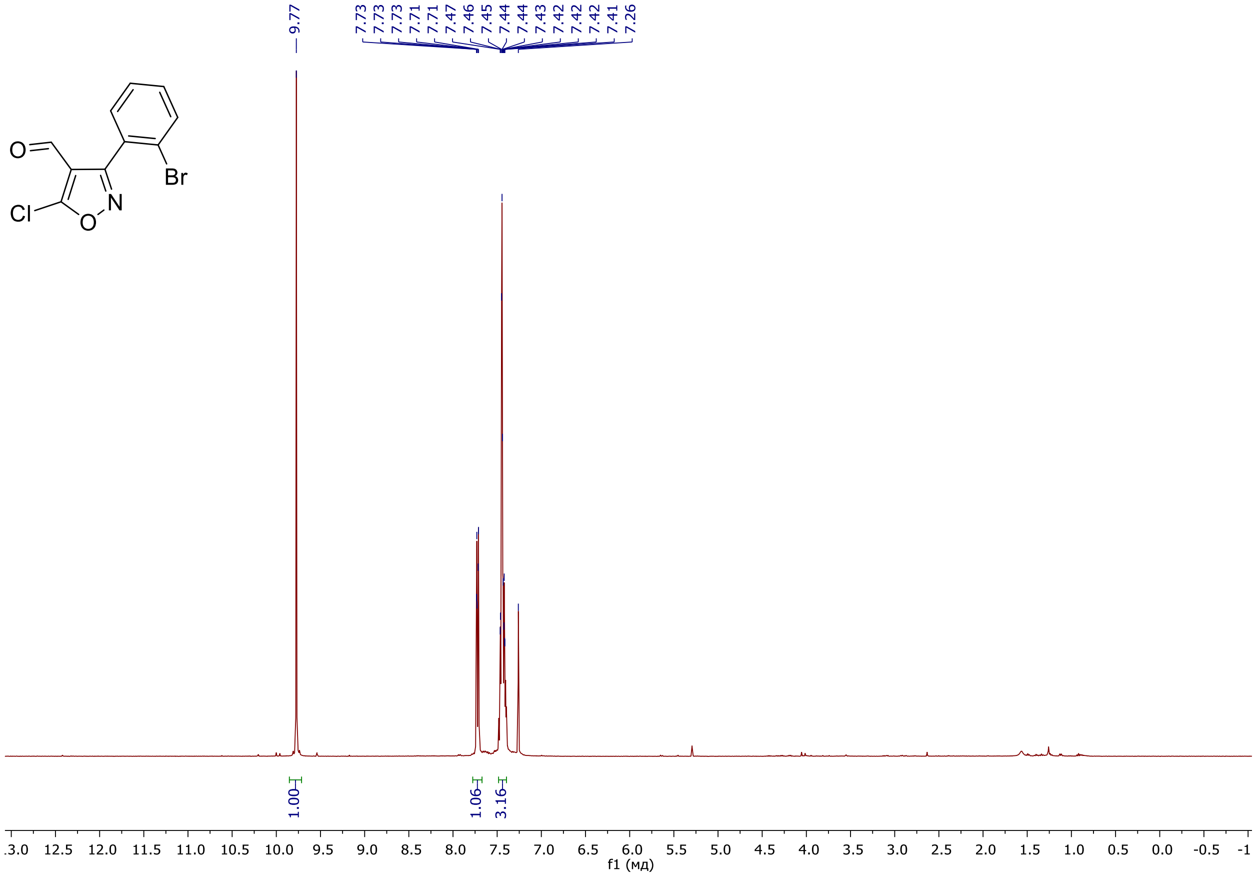

$^{13}C\{^{1}H\}$ NMR spectrum (100 MHz, $CDCl_3$) of compound **4h**

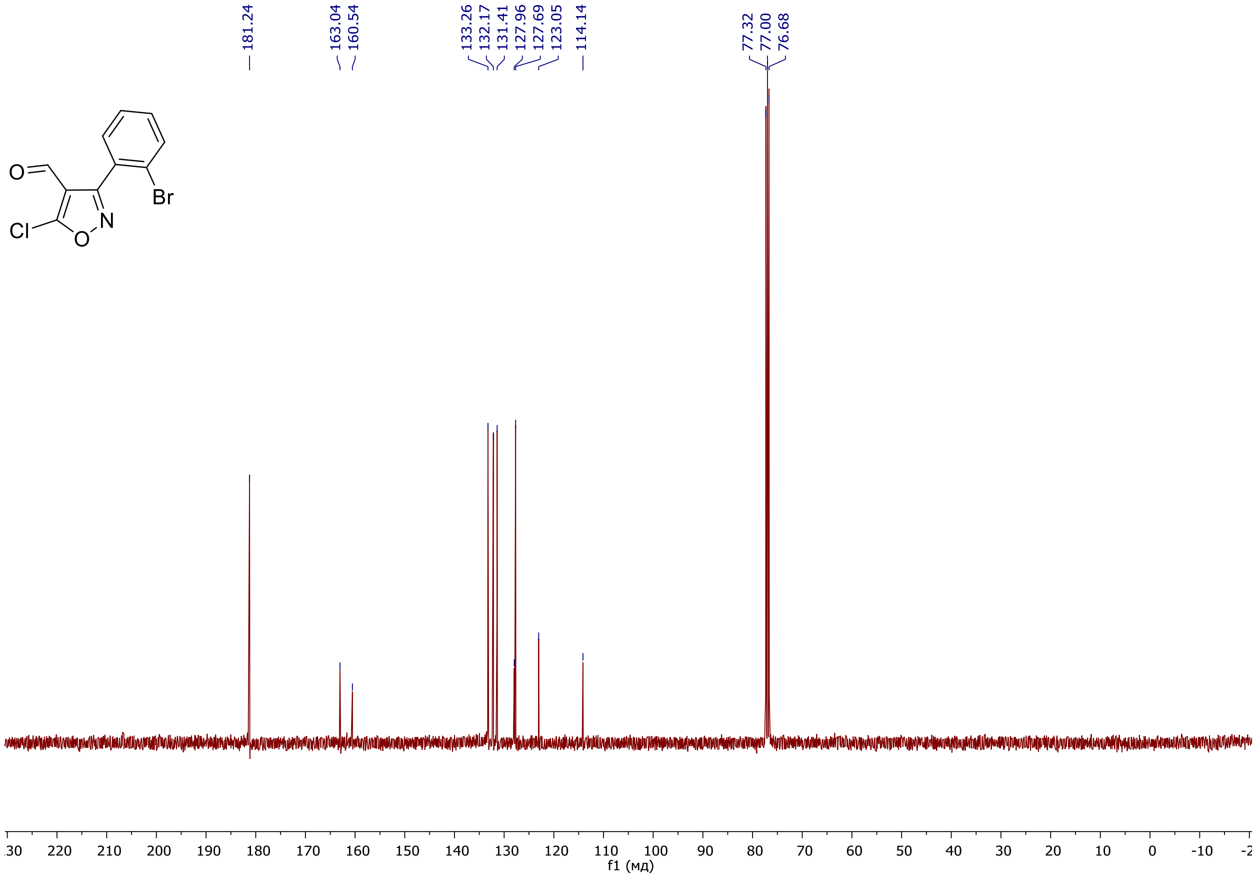

^{1}H NMR spectrum (400 MHz, $CDCl_3$) of compound **4i**

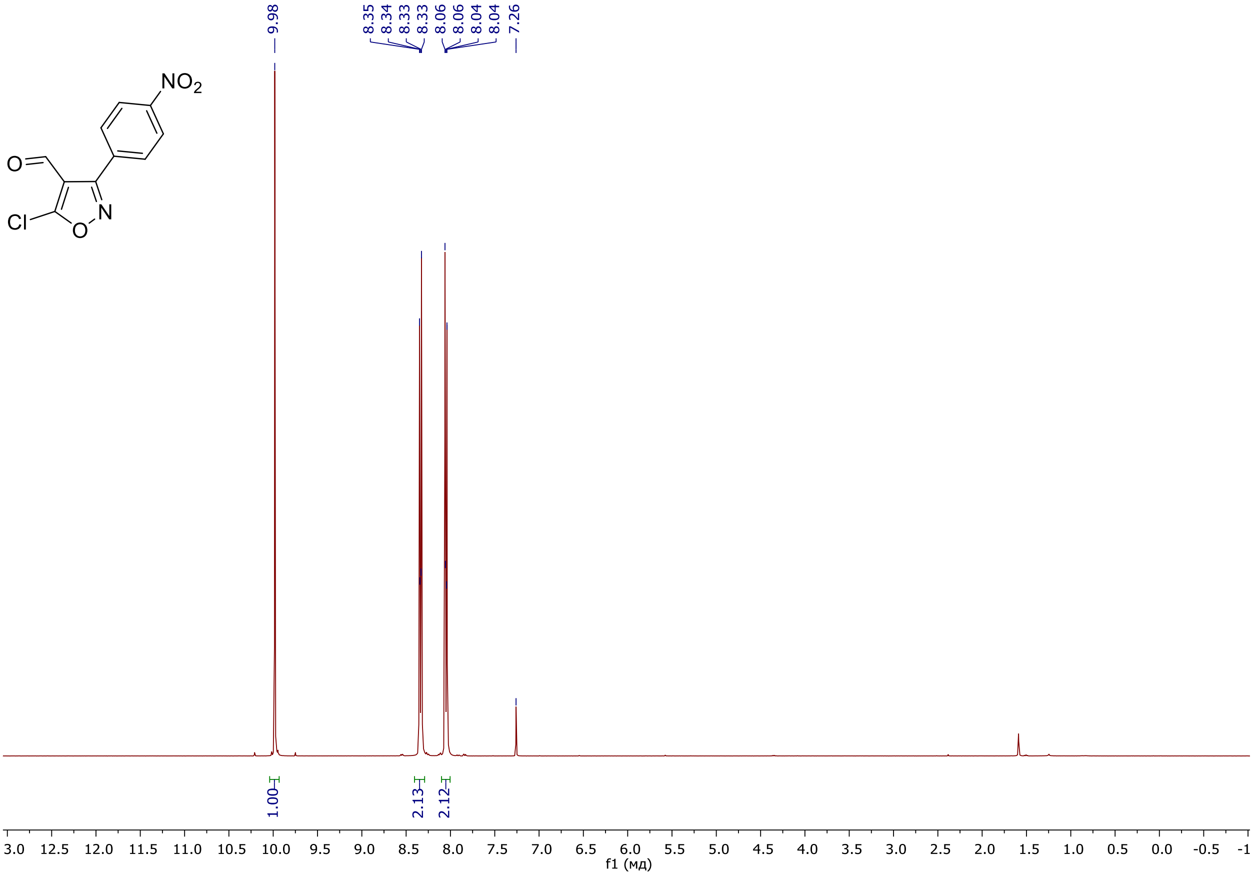

^{13}C{^{1}H} NMR spectrum (100 MHz, $CDCl_3$) of compound **4i**

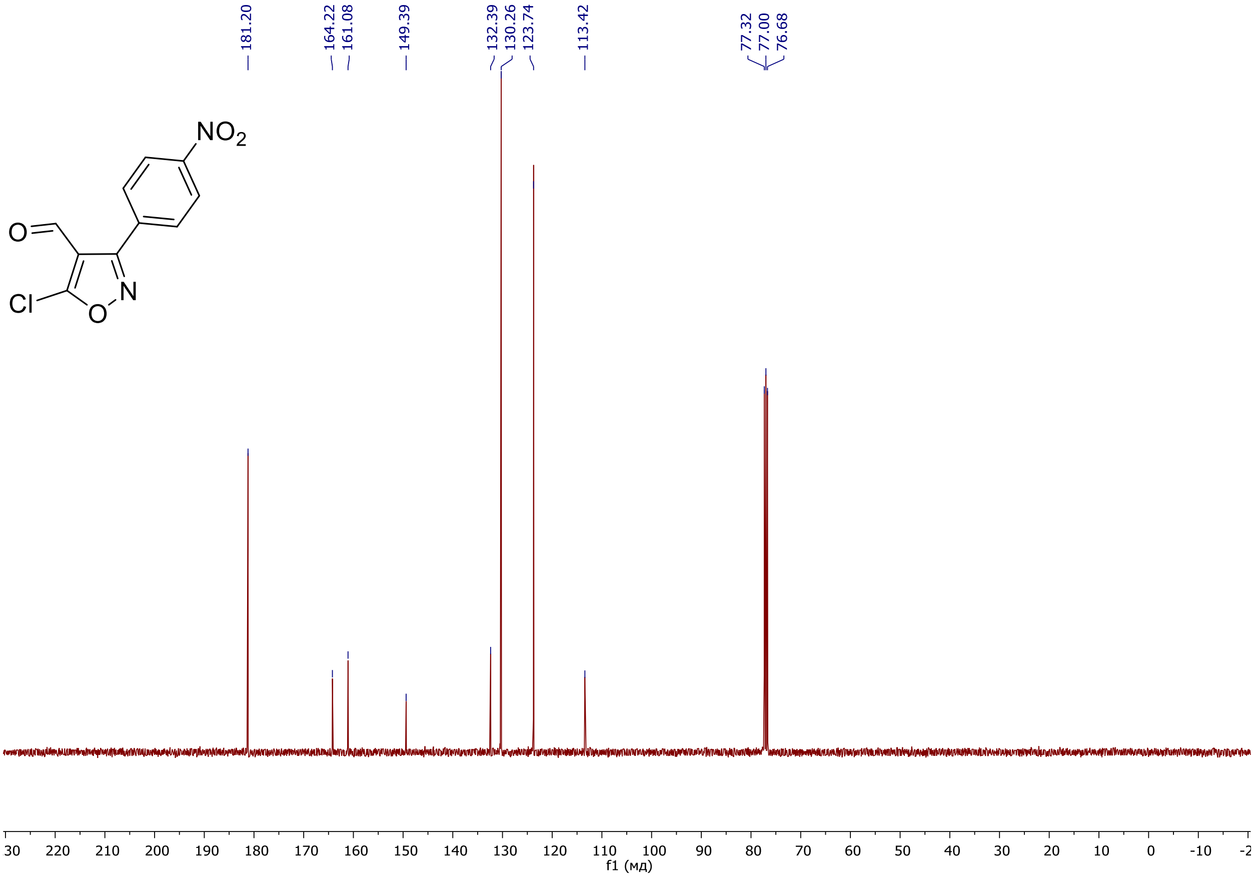

^{1}H NMR spectrum (400 MHz, $CDCl_3$) of compound **4j**

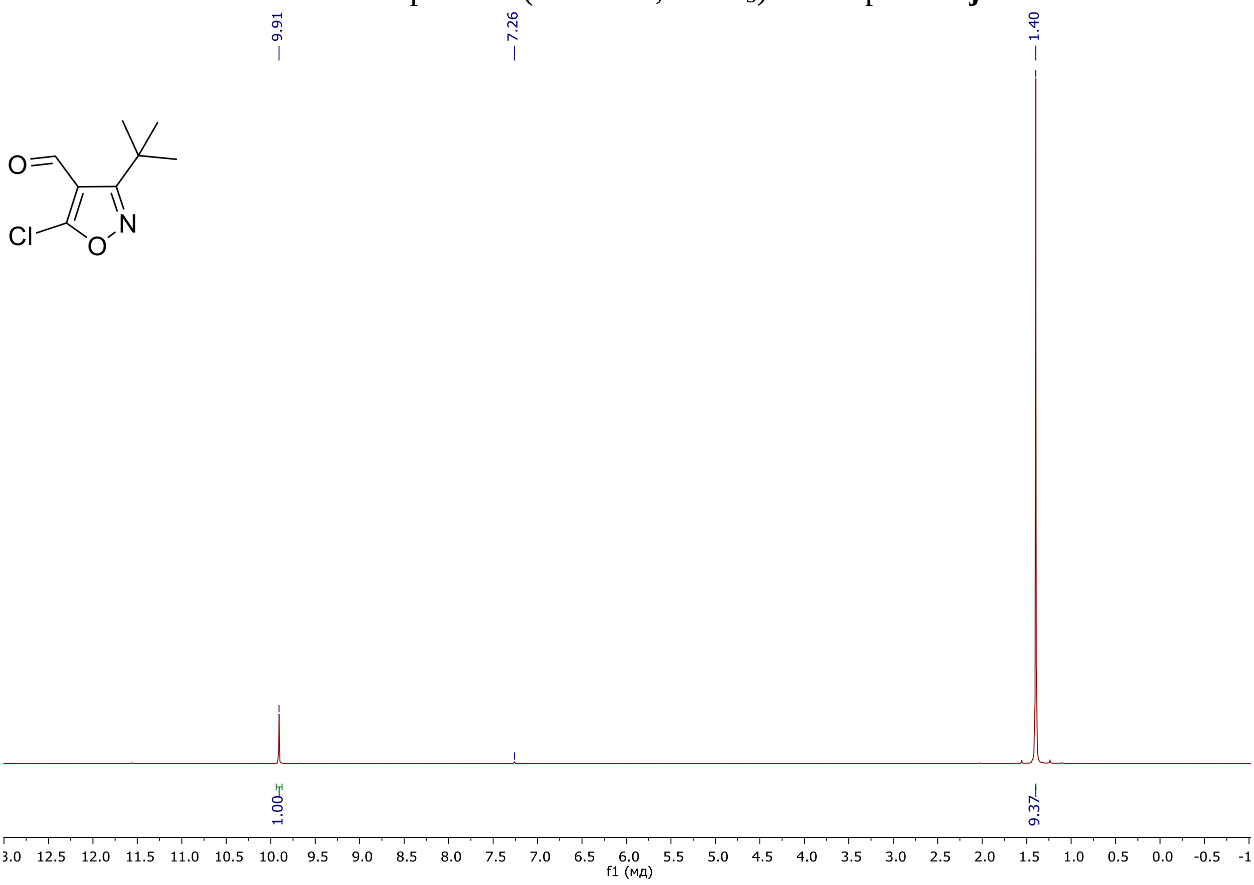

^{13}C{^{1}H} NMR spectrum (100 MHz, $CDCl_3$) of compound **4j**

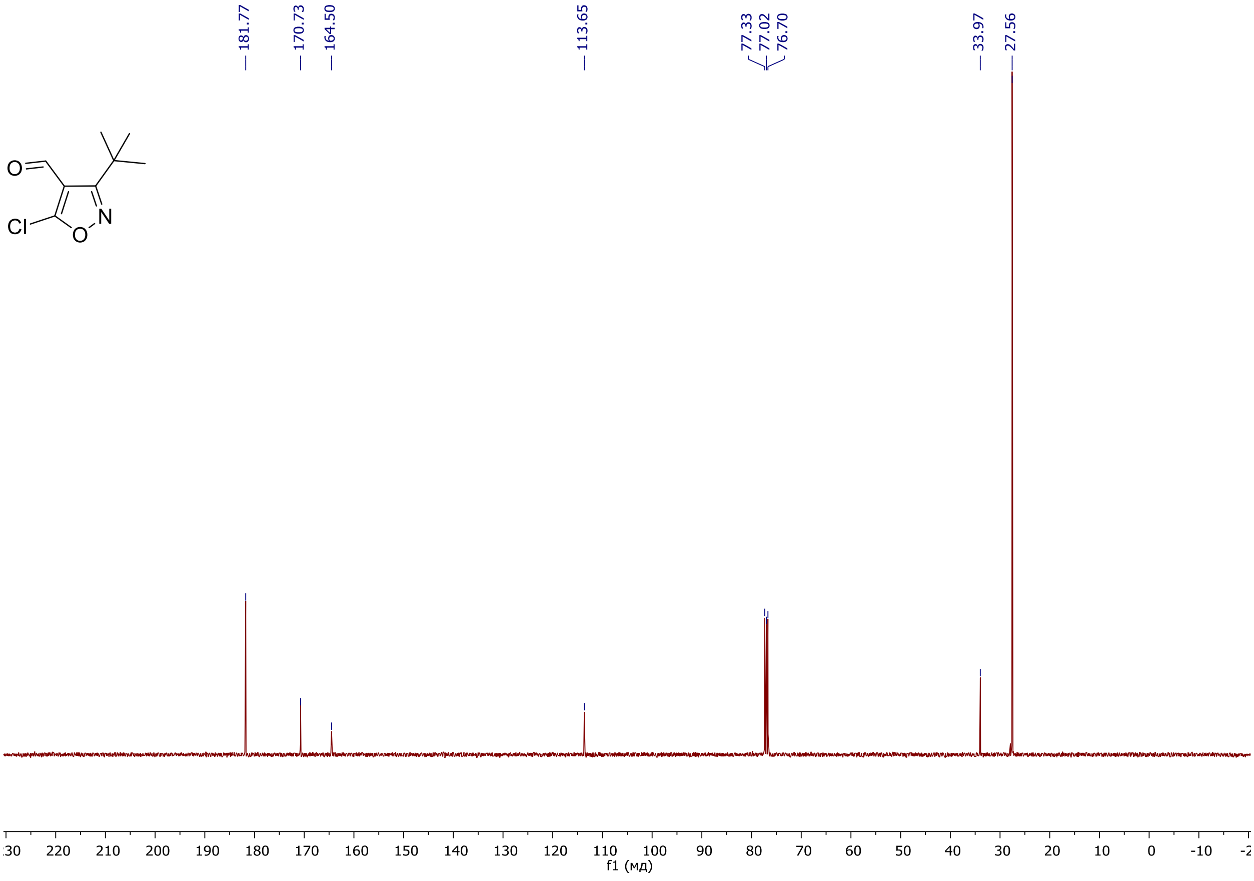

^{1}H NMR spectrum (400 MHz, $CDCl_3$) of compound **5b**

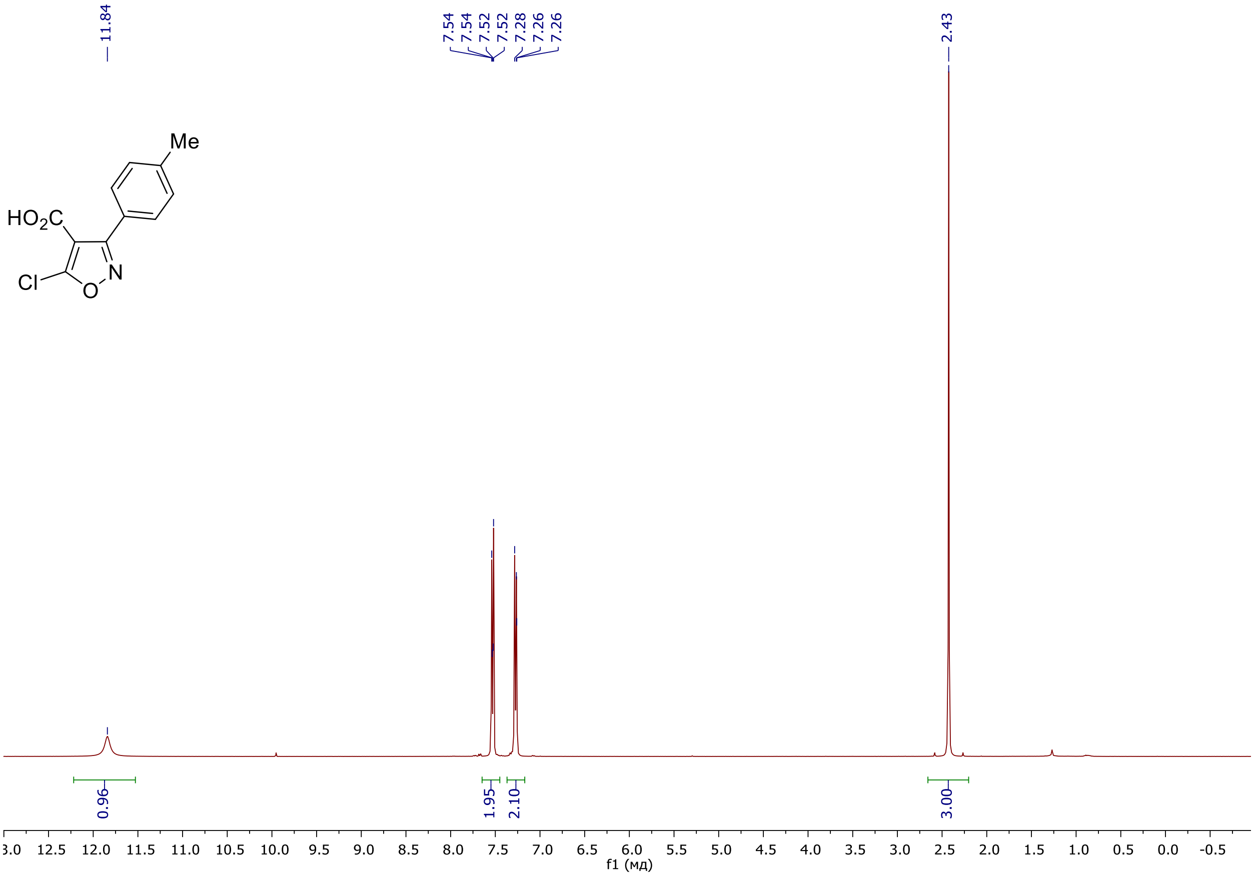

$^{13}C\{^{1}H\}$ NMR spectrum (100 MHz, $CDCl_3$) of compound **5b**

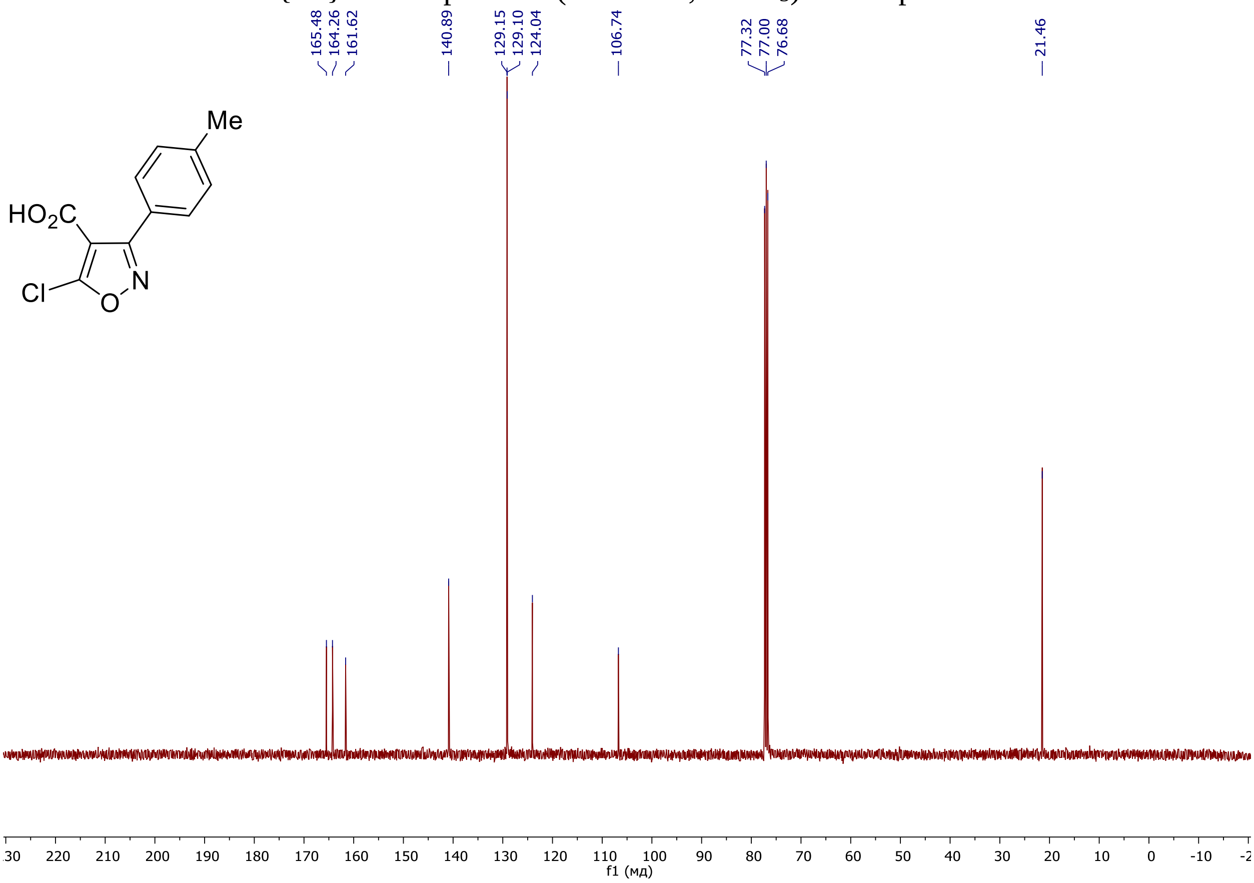

^{1}H NMR spectrum (400 MHz, DMSO-d_6) of compound **5d**

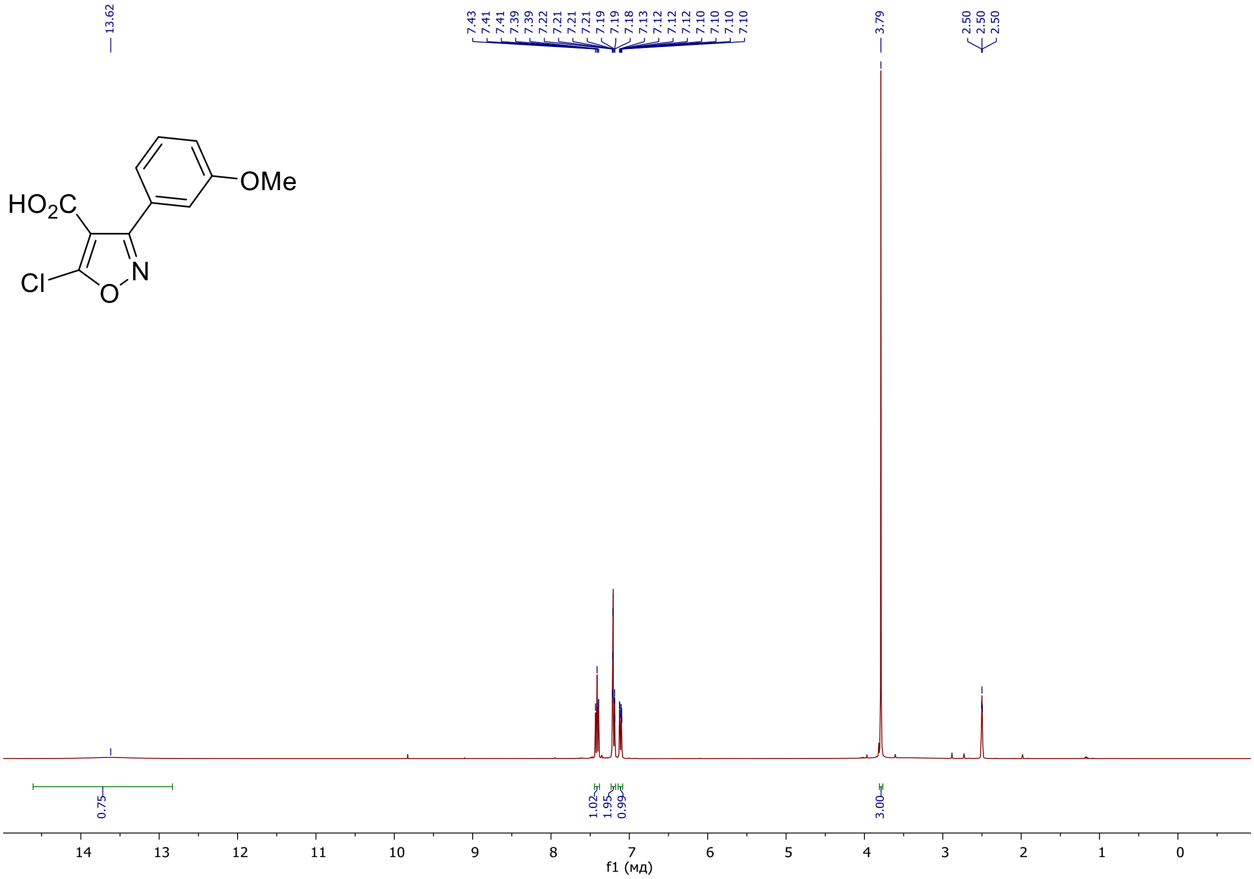

^{13}C{^{1}H} NMR spectrum (100 MHz, DMSO-d_6) of compound **5d**

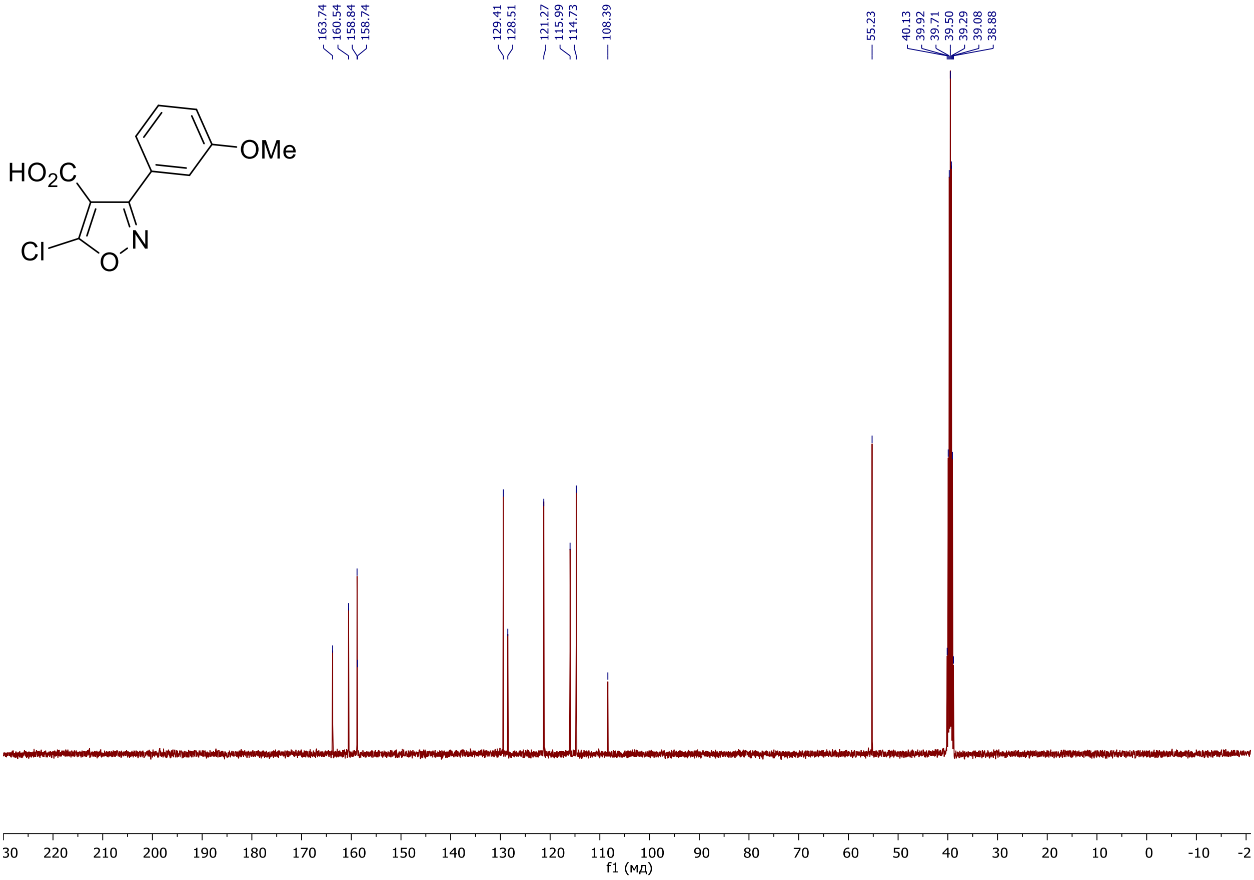

^{1}H NMR spectrum (400 MHz, DMSO-d_6) of compound **5g**

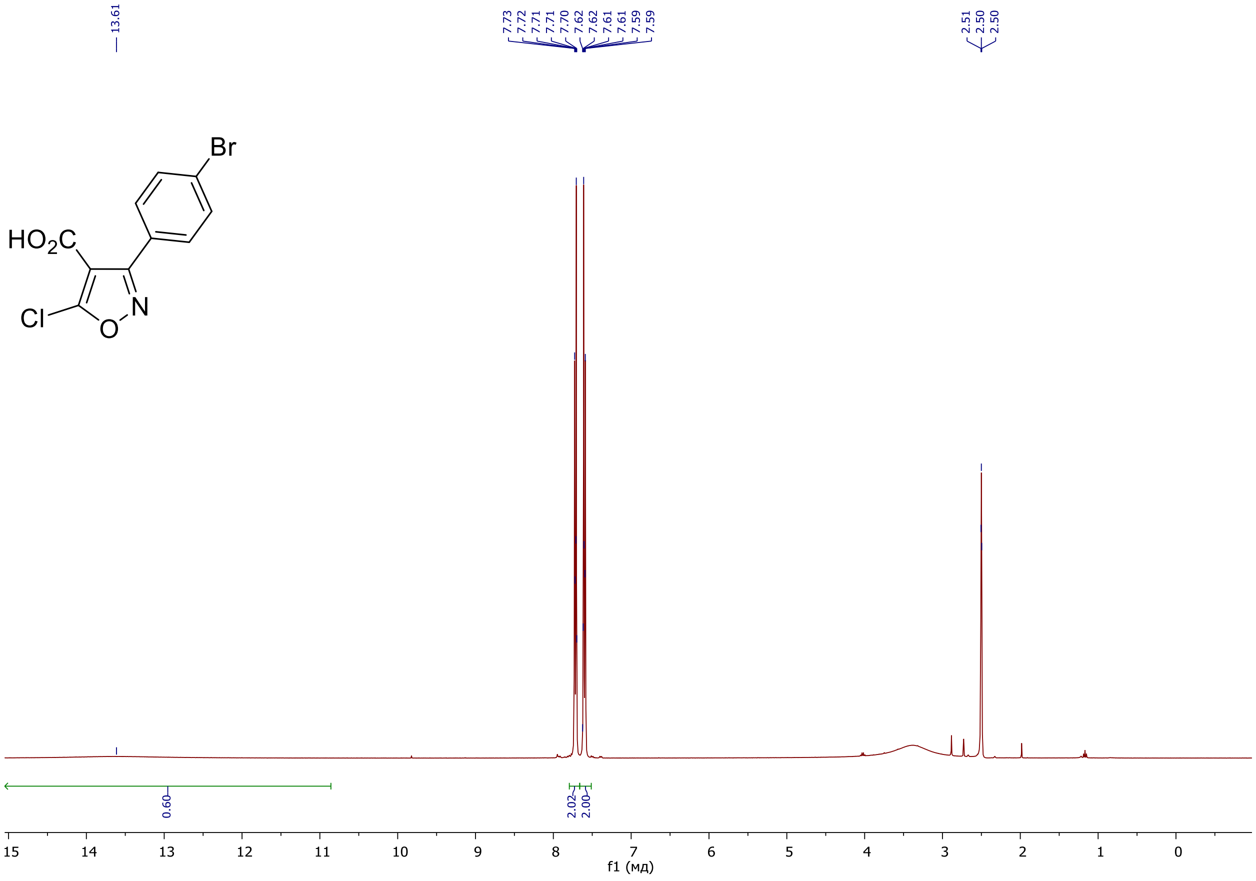

$^{13}C\{^{1}H\}$ NMR spectrum (100 MHz, DMSO-d_6) of compound **5g**

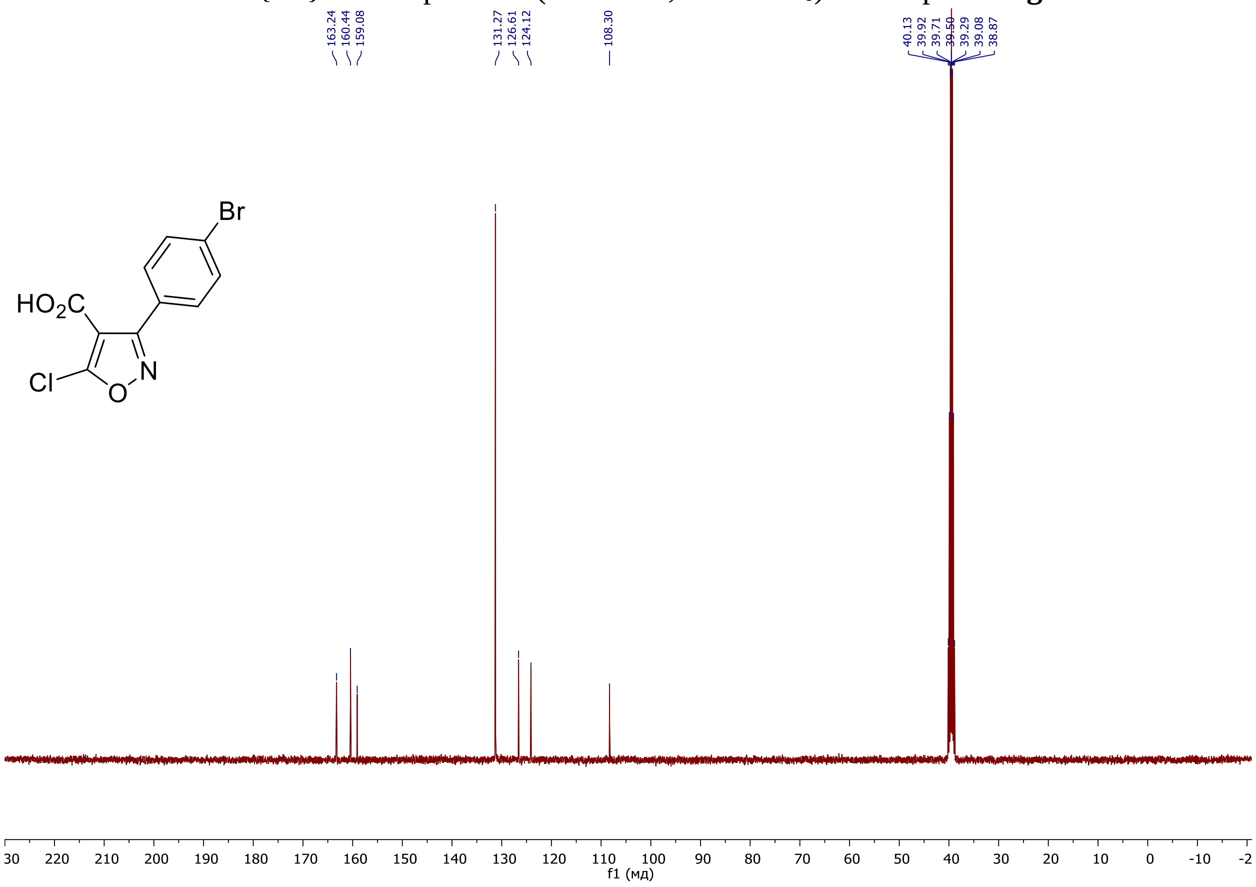

^{1}H NMR spectrum (400 MHz, DMSO-d_6) of compound **5h**

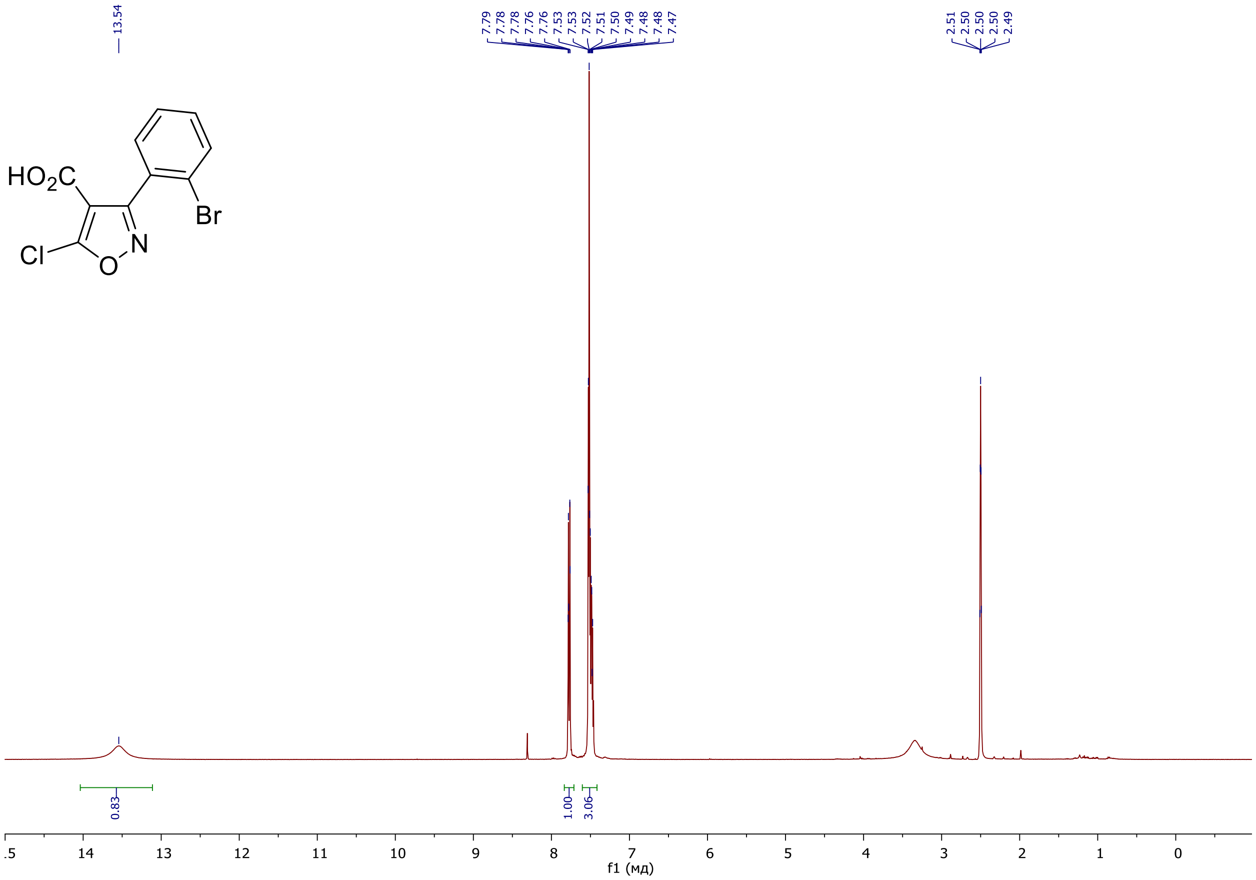

^{13}C{^{1}H} NMR spectrum (100 MHz, DMSO-d_6) of compound **5h**

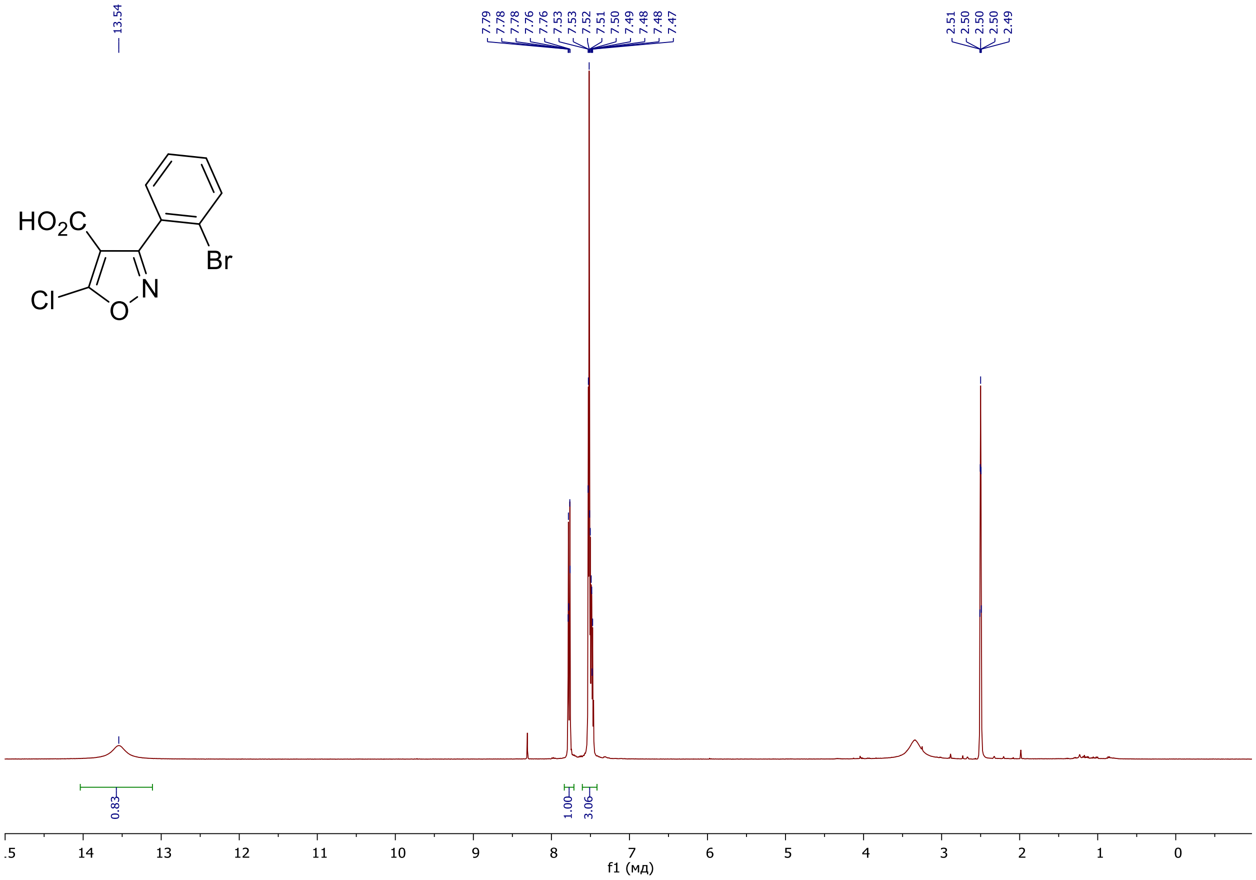

^{1}H NMR spectrum (400 MHz, DMSO-d_6) of compound **5i**

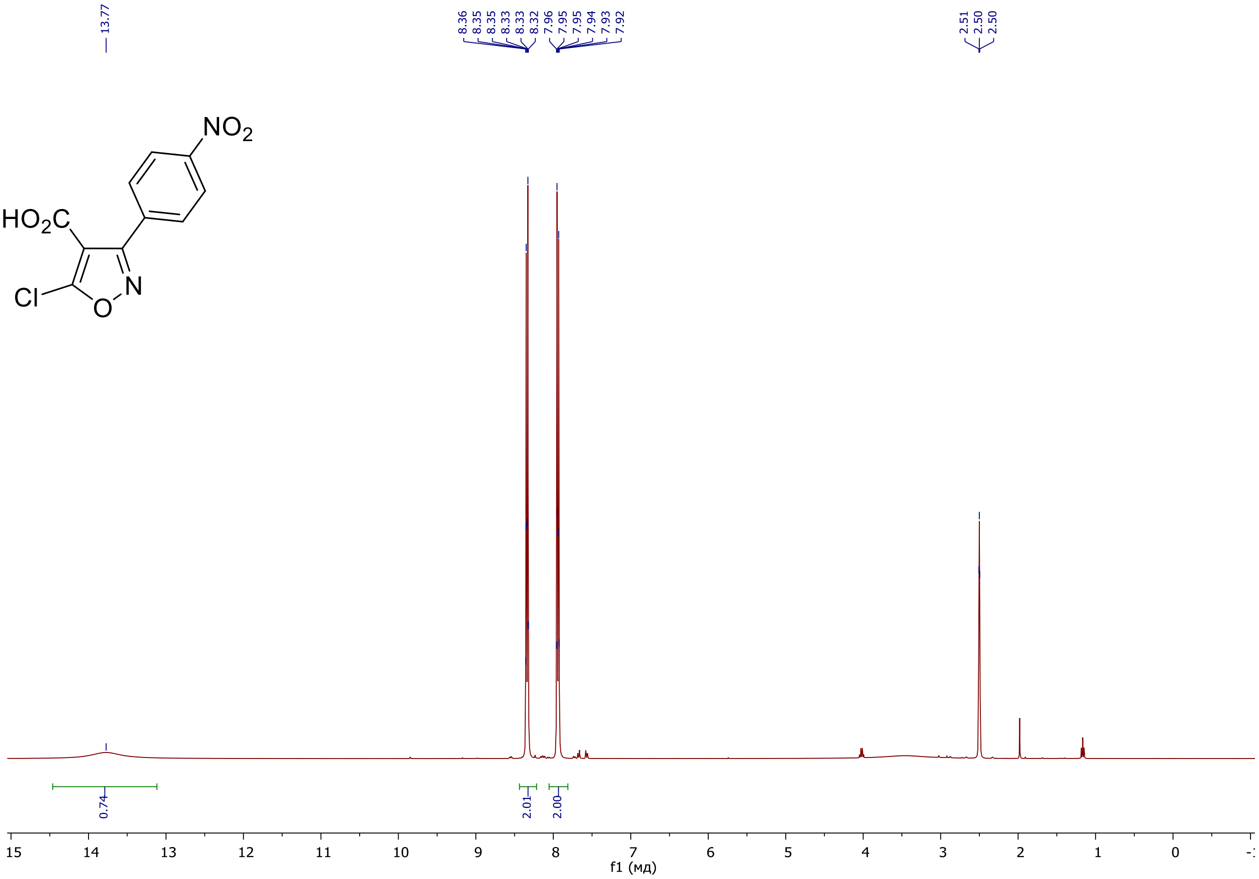

$^{13}C\{^{1}H\}$ NMR spectrum (100 MHz, DMSO-d_6) of compound **5i**

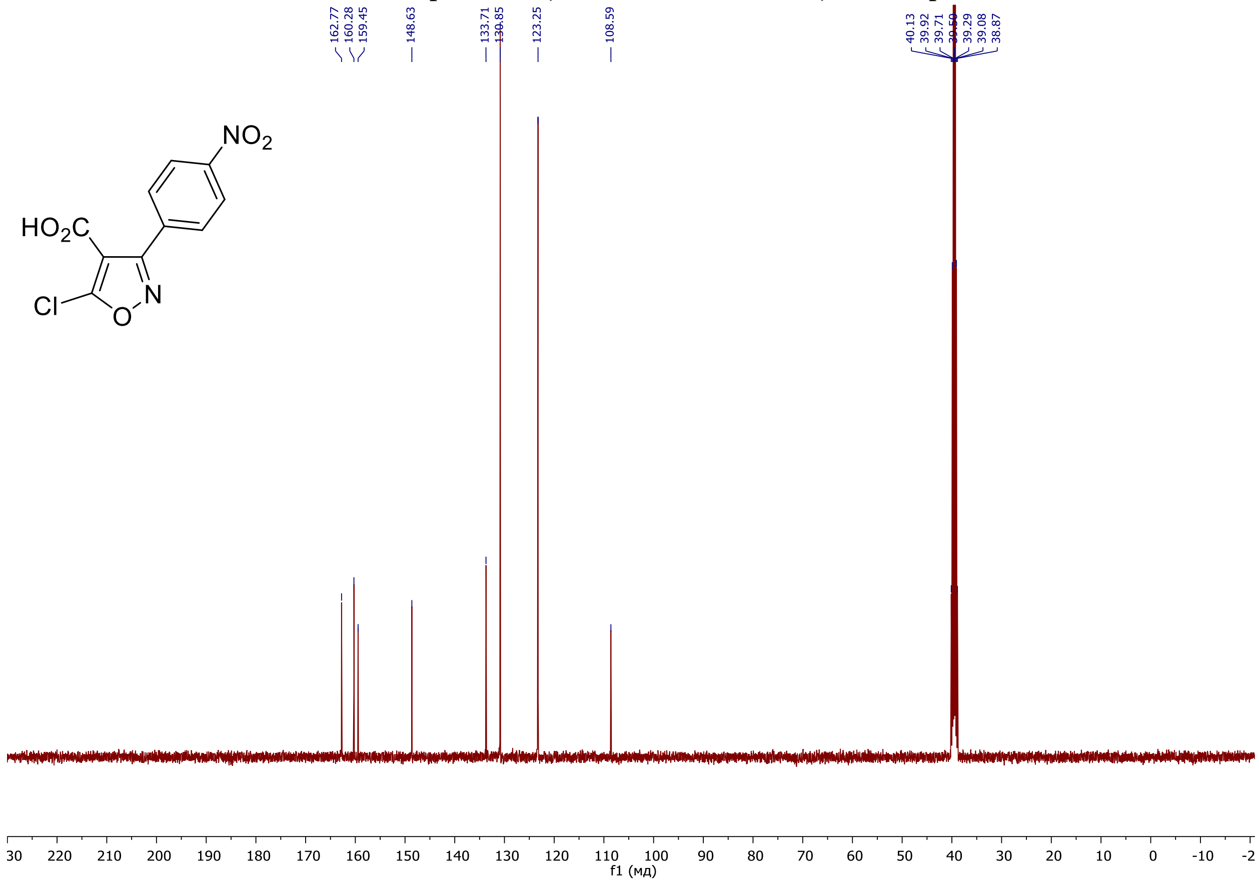

^{1}H NMR spectrum (400 MHz, $CDCl_3$) of compound **5j**

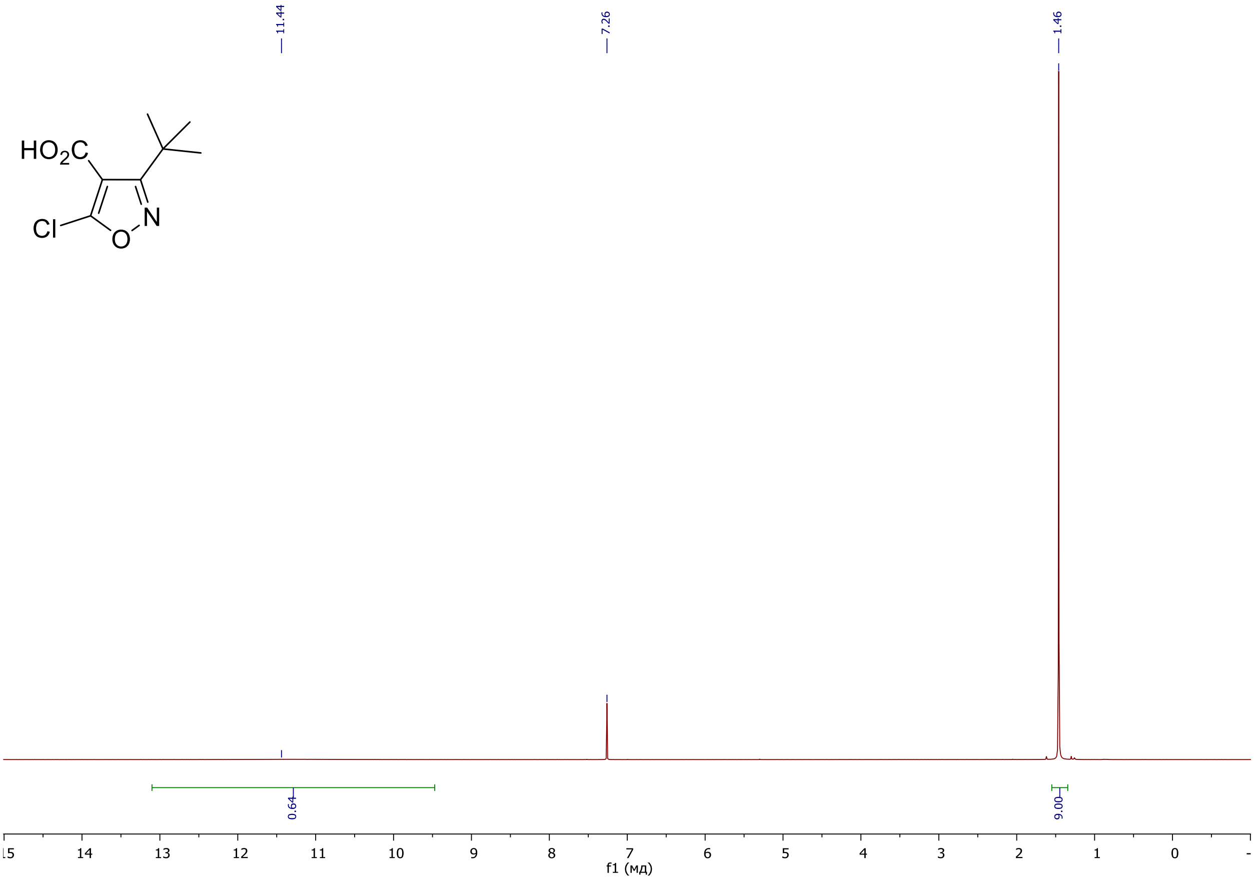

$^{13}C\{^{1}H\}$ NMR spectrum (100 MHz, $CDCl_3$) of compound **5j**

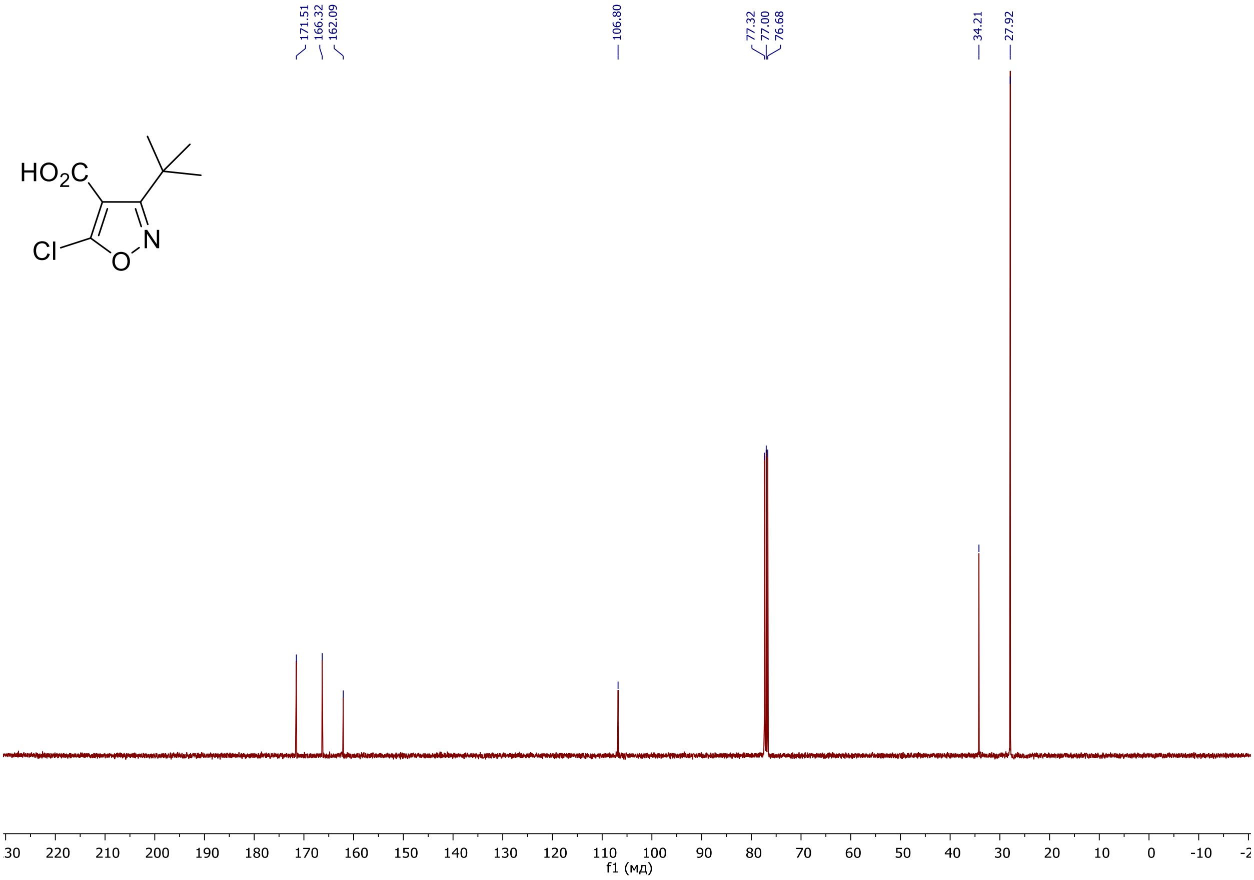

^{1}H NMR spectrum (400 MHz, $CDCl_3$) of compound **1a**

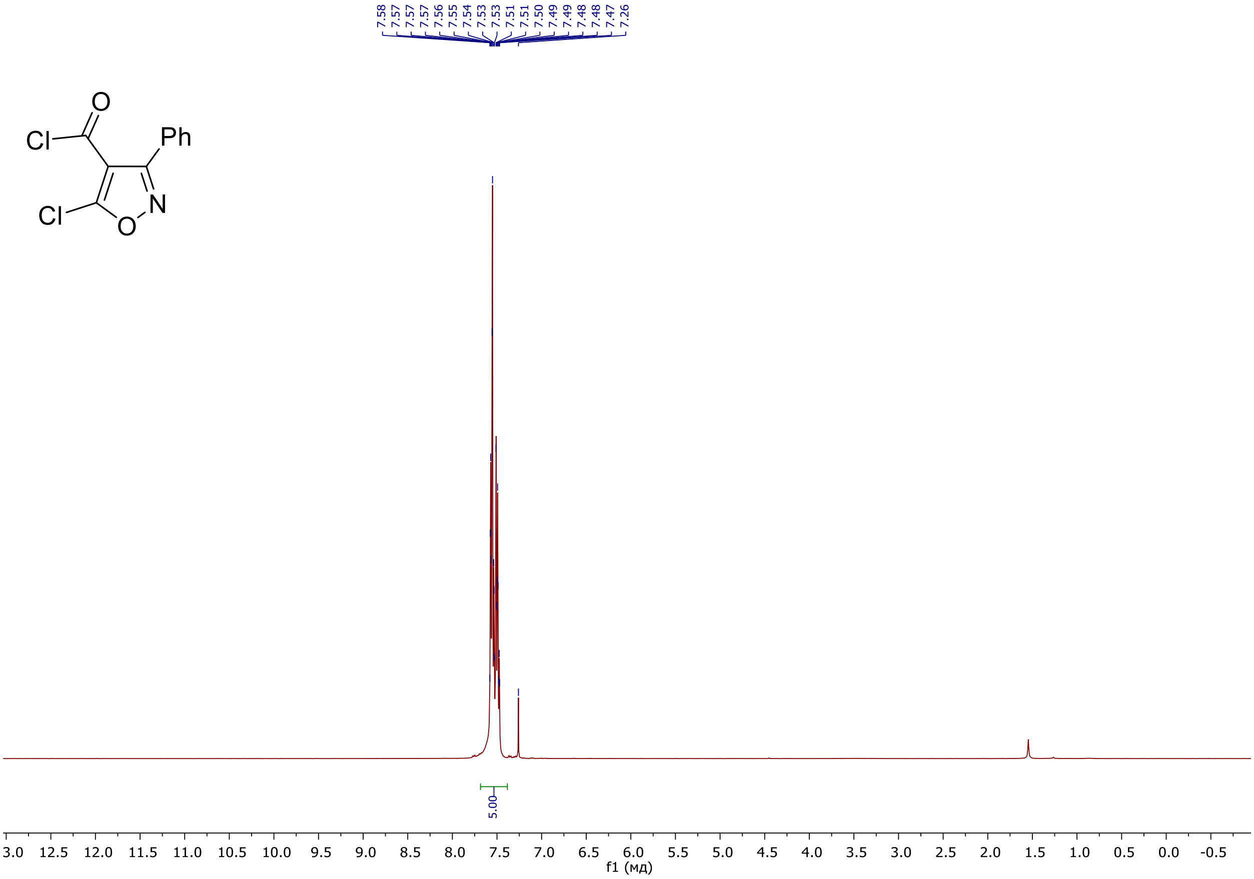

$^{13}C\{^{1}H\}$ NMR spectrum (100 MHz, $CDCl_3$) of compound **1a**

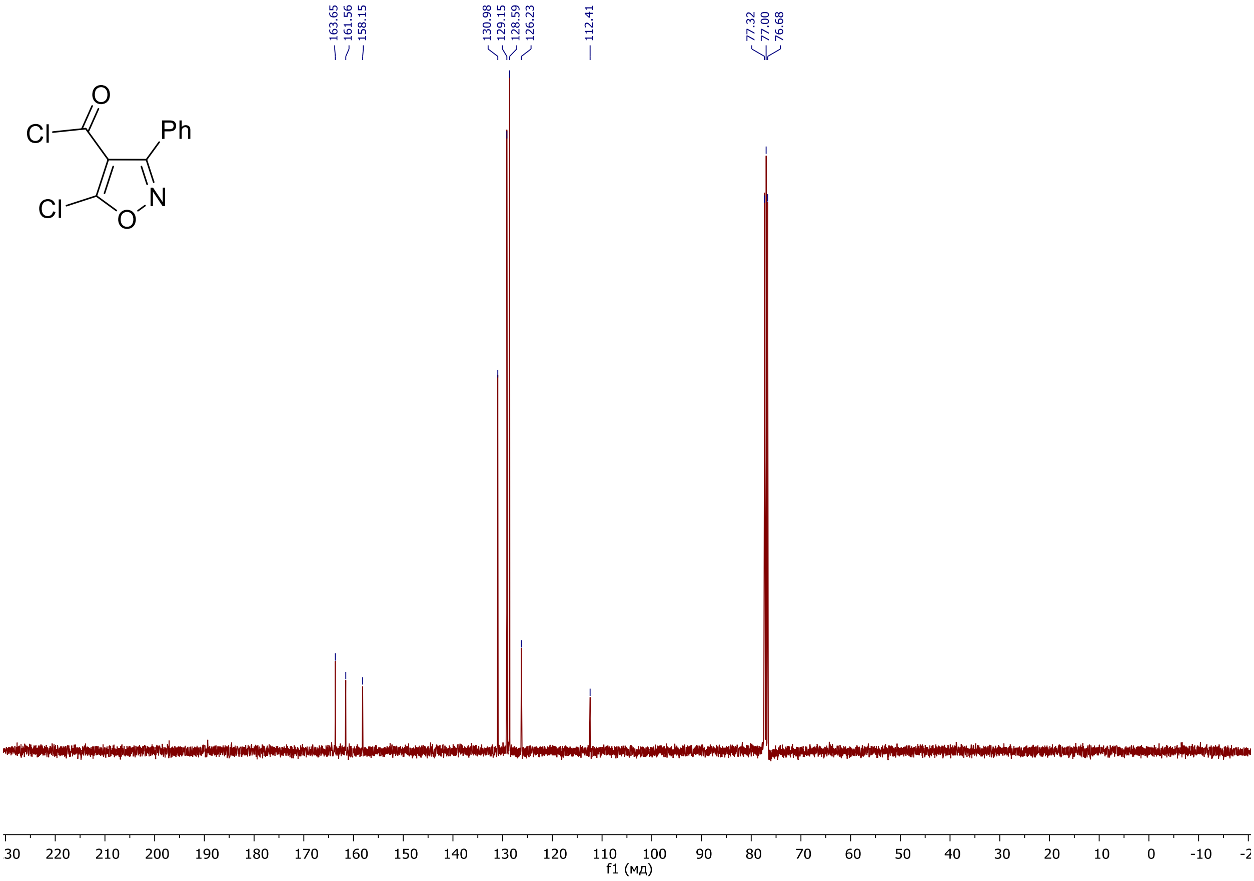

^{1}H NMR spectrum (400 MHz, $CDCl_3$) of compound **1b**

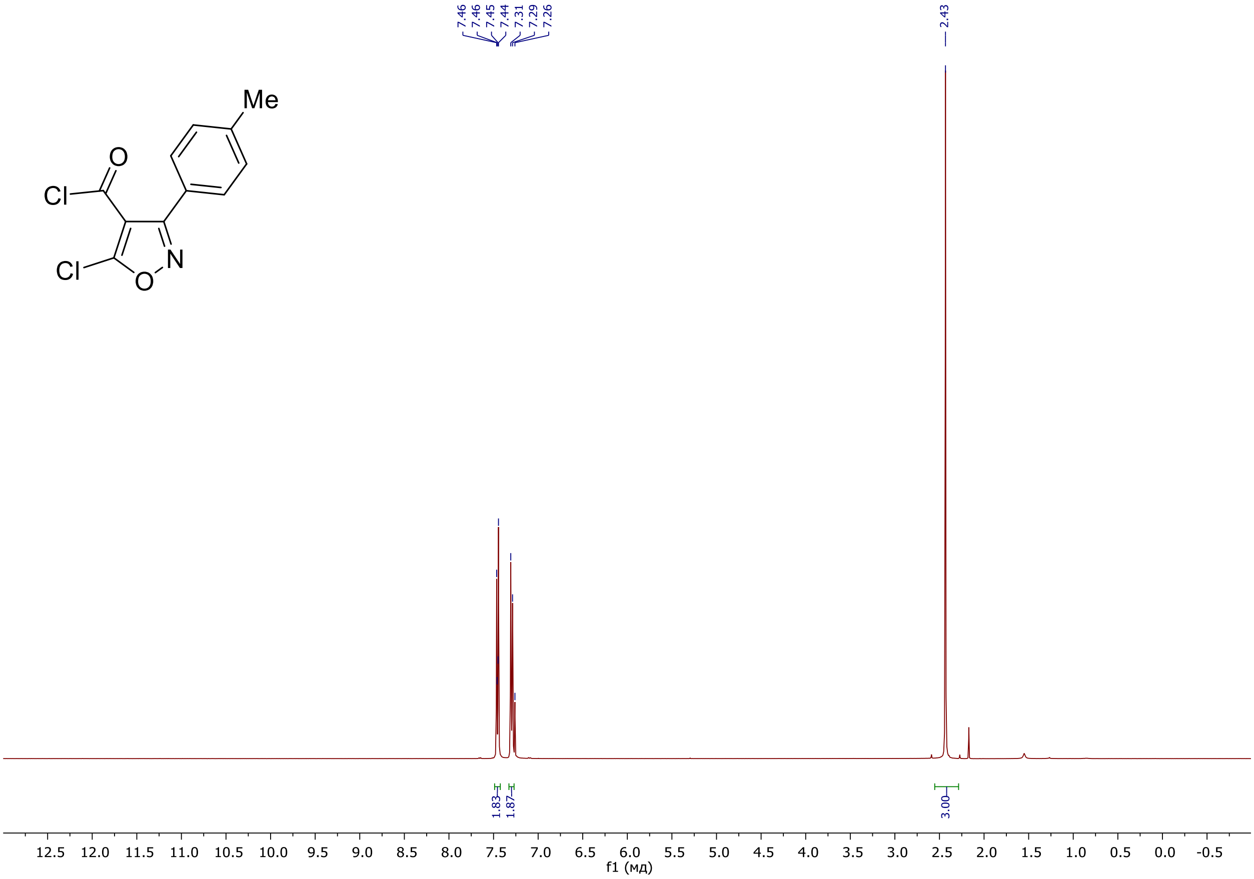

$^{13}C\{^{1}H\}$ NMR spectrum (100 MHz, $CDCl_3$) of compound **1b**

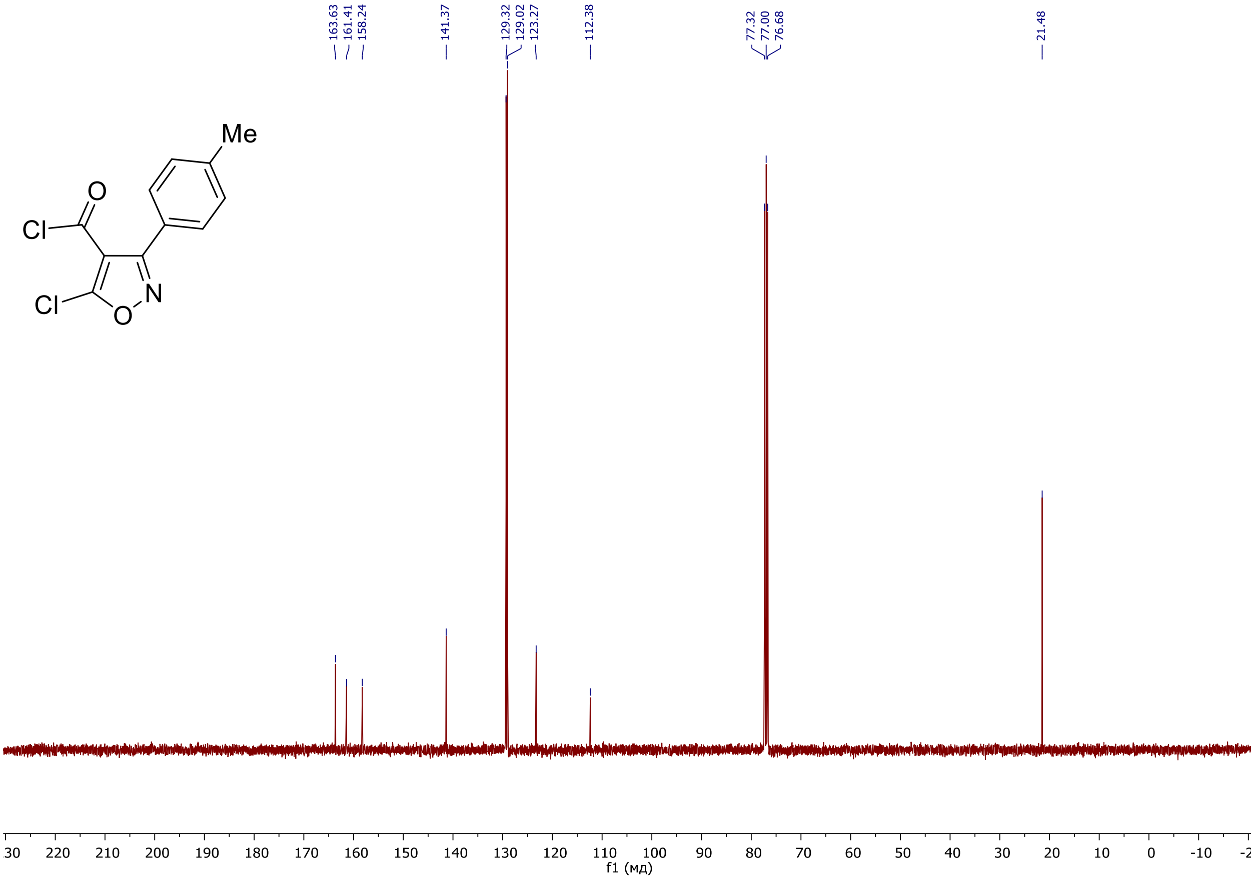

^{1}H NMR spectrum (400 MHz, $CDCl_3$) of compound **1c**

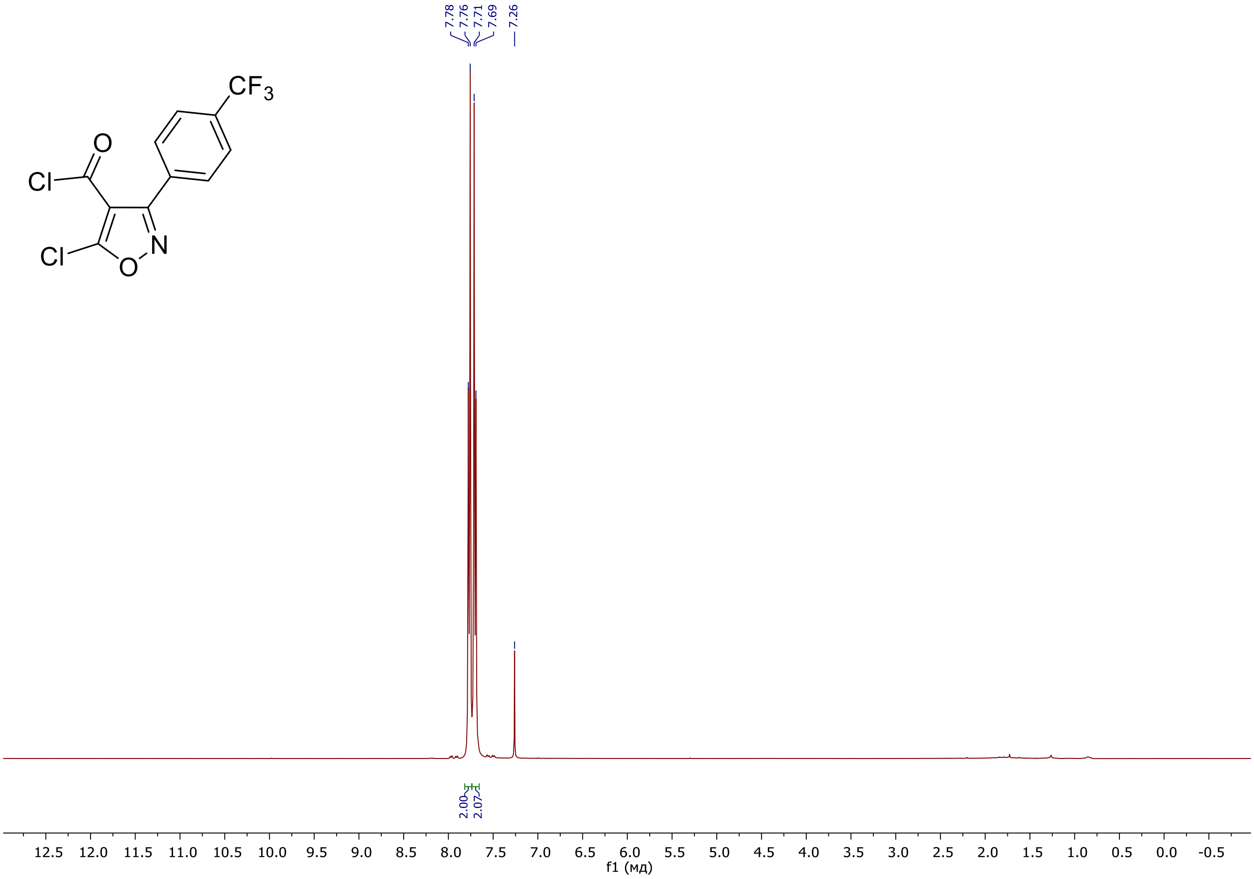

$^{13}C\{^1H\}$ NMR spectrum (100 MHz, $CDCl_3$) of compound **1c**

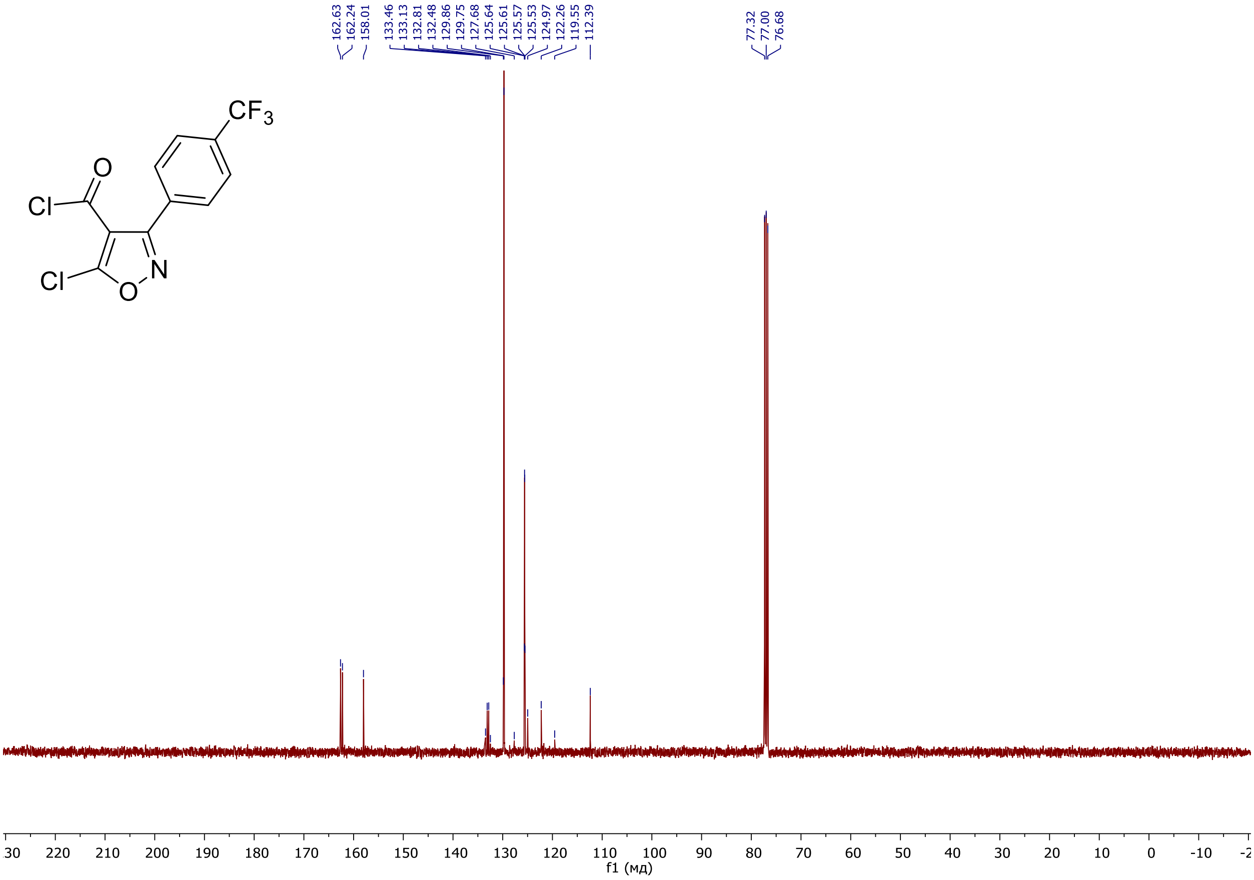

$^{19}F\{^1H\}$ NMR spectrum (376 MHz, $CDCl_3$) of compound **1c**

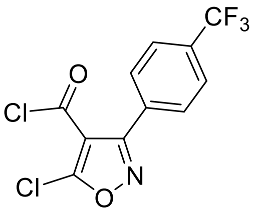

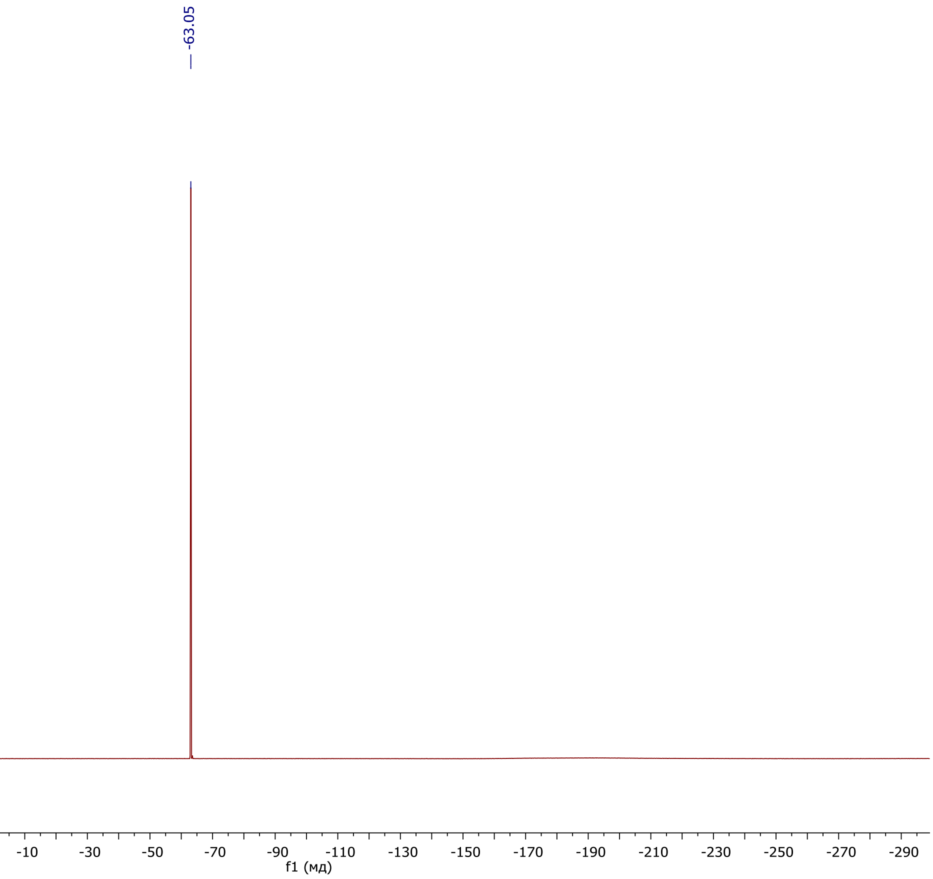

^{1}H NMR spectrum (400 MHz, $CDCl_3$) of compound **1d**

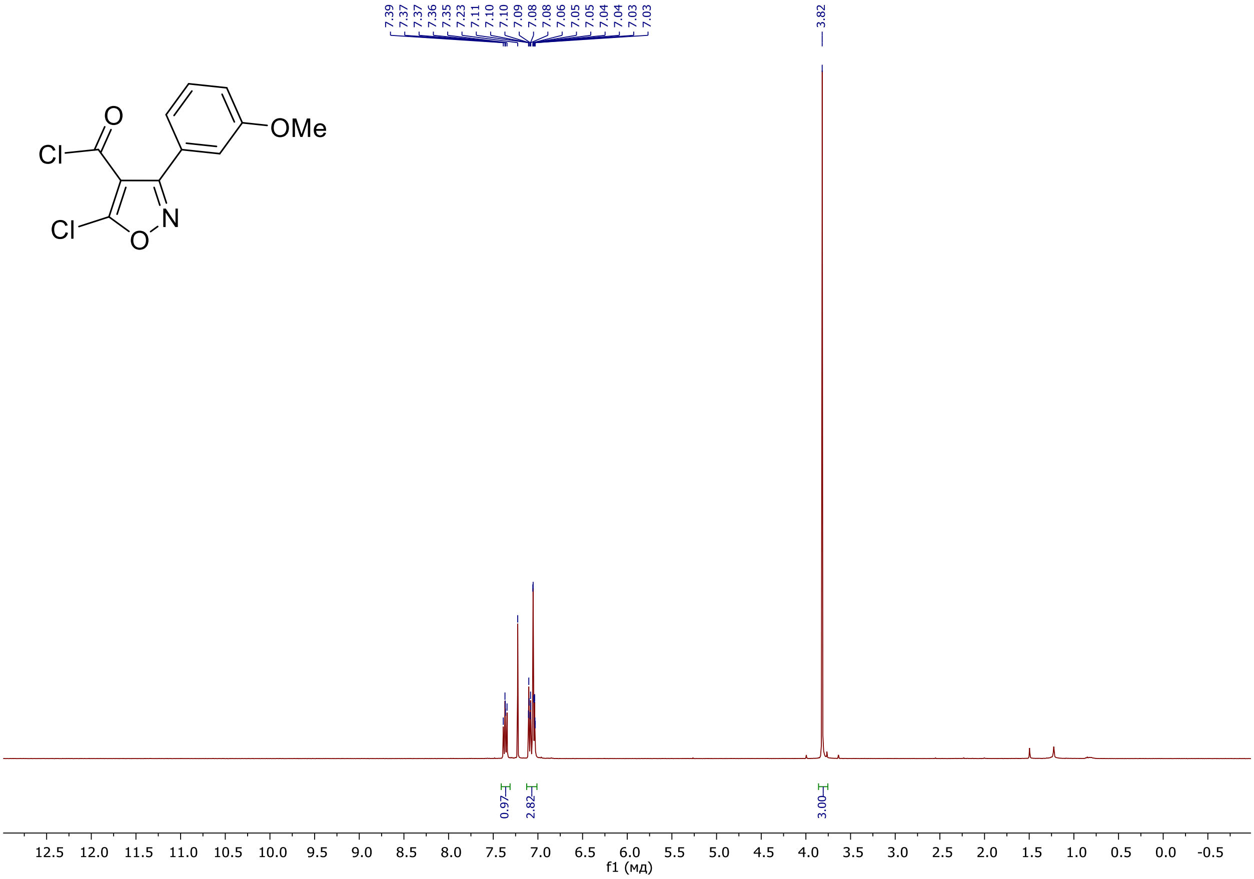

^{13}C{^{1}H} NMR spectrum (100 MHz, $CDCl_3$) of compound **1d**

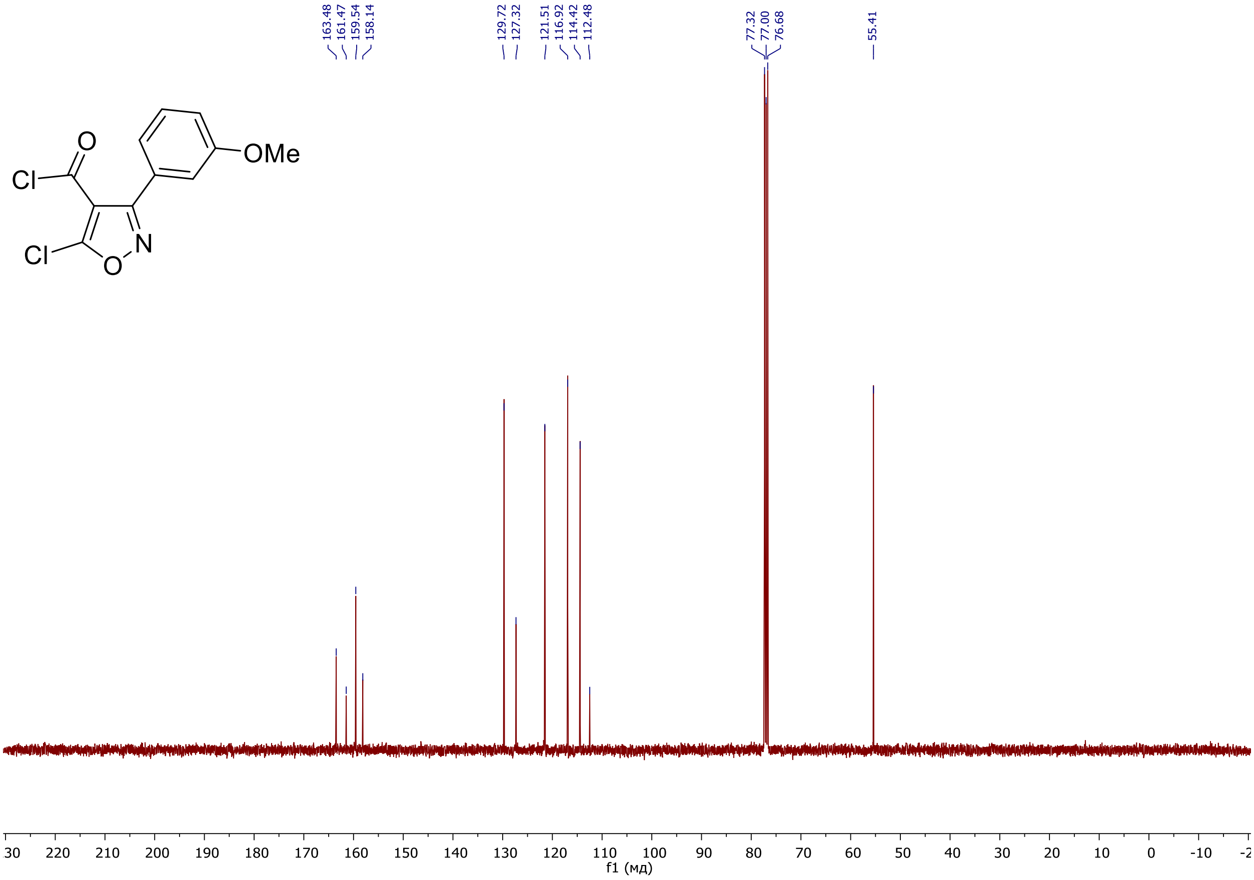

^{1}H NMR spectrum (400 MHz, $CDCl_3$) of compound **1e**

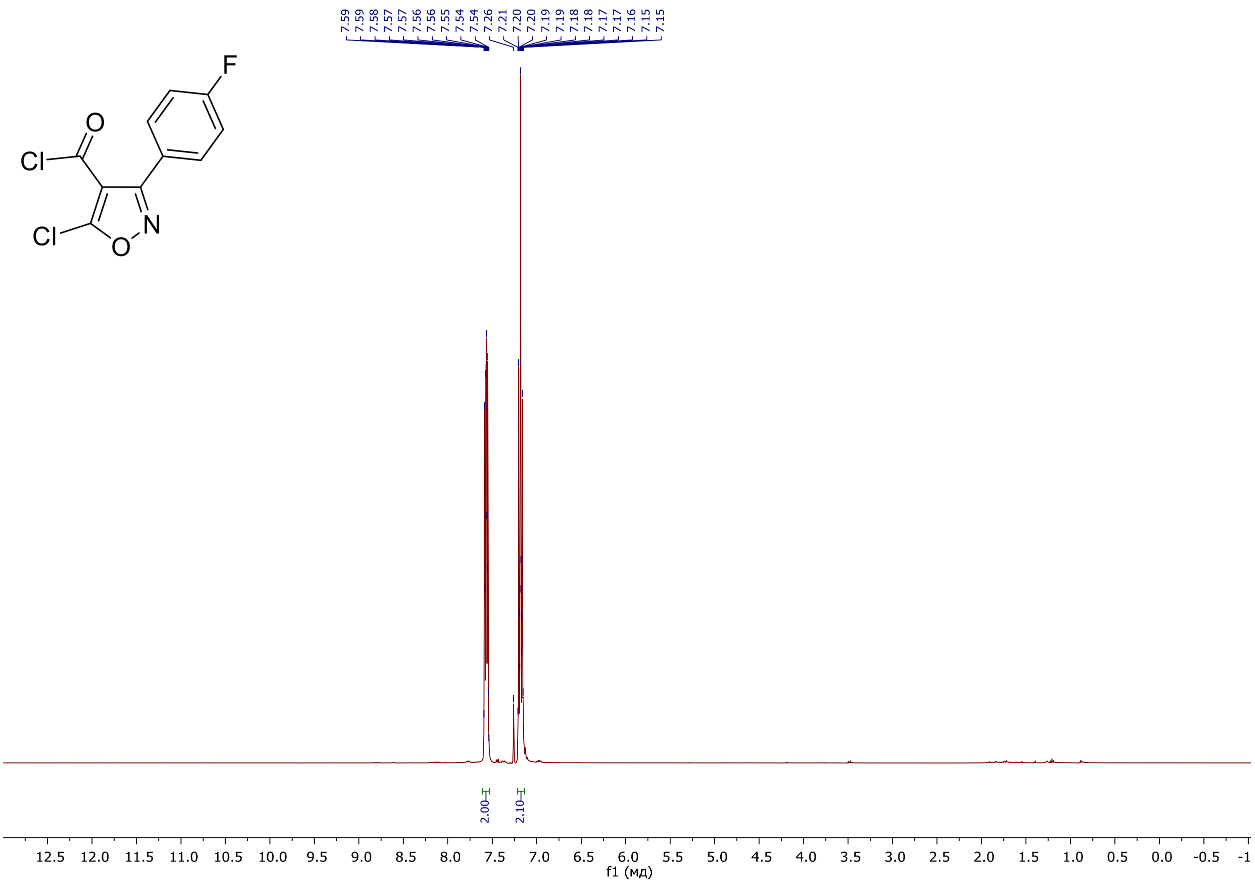

$^{13}C\{^{1}H\}$ NMR spectrum (100 MHz, $CDCl_3$) of compound **1e**

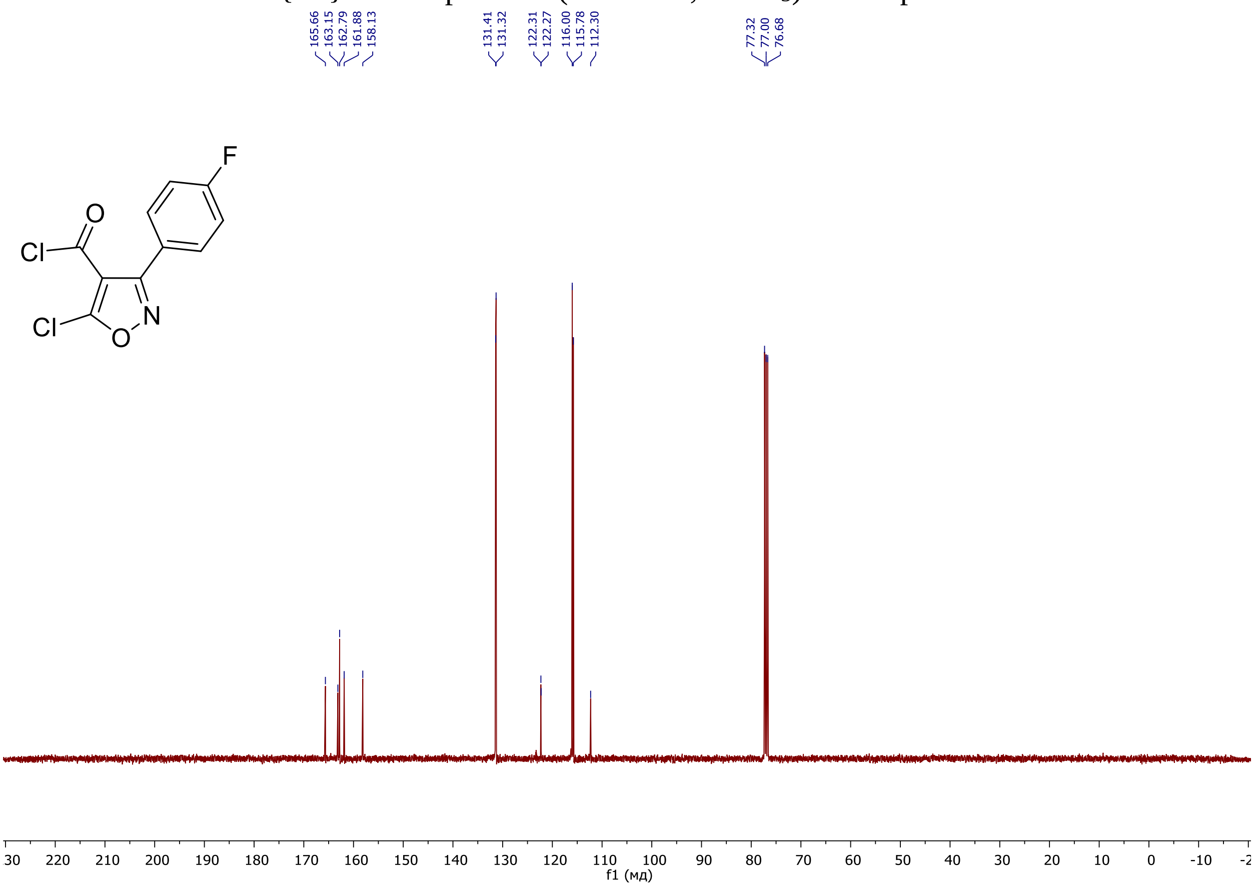

$^{19}F\{^{1}H\}$ NMR spectrum (376 MHz, $CDCl_3$) of compound **1e**

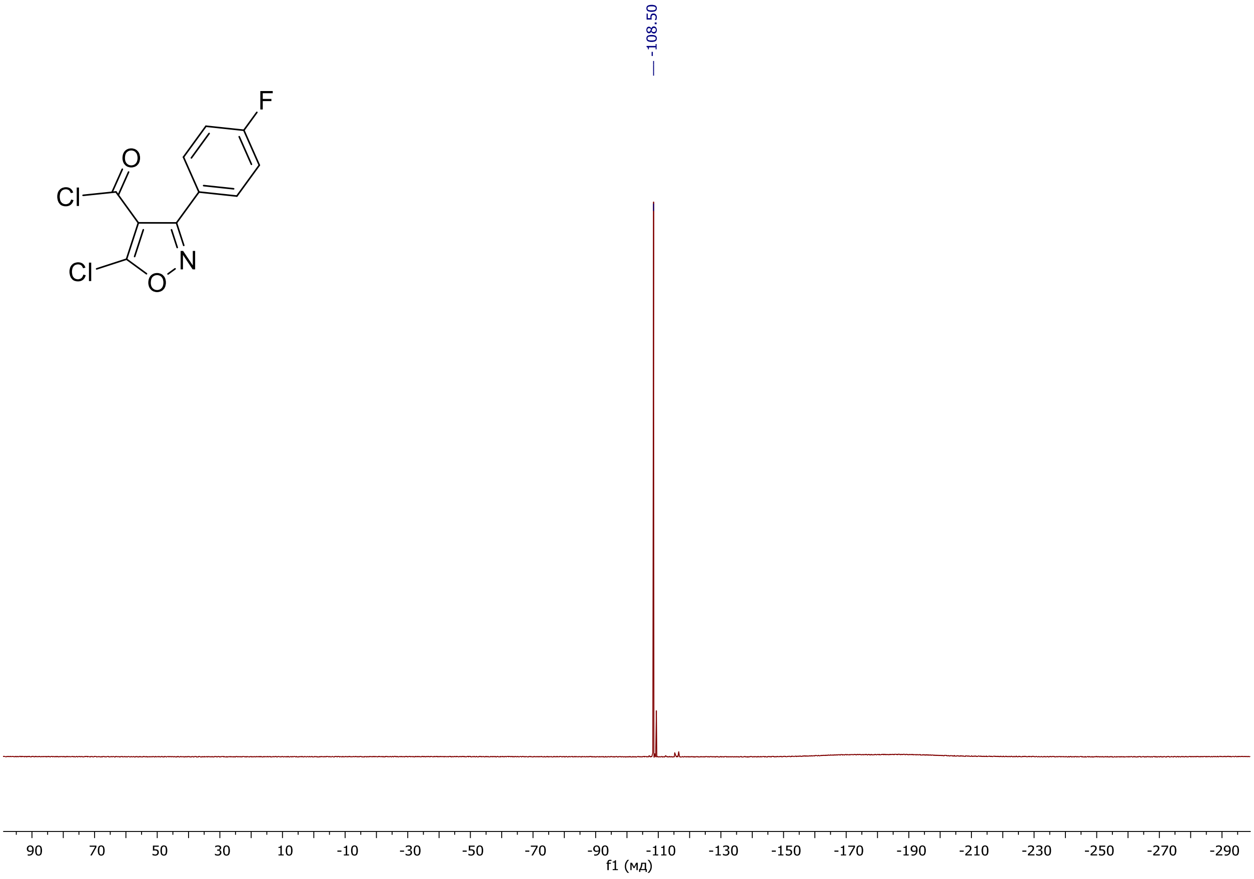

^{1}H NMR spectrum (400 MHz, $CDCl_3$) of compound **1f**

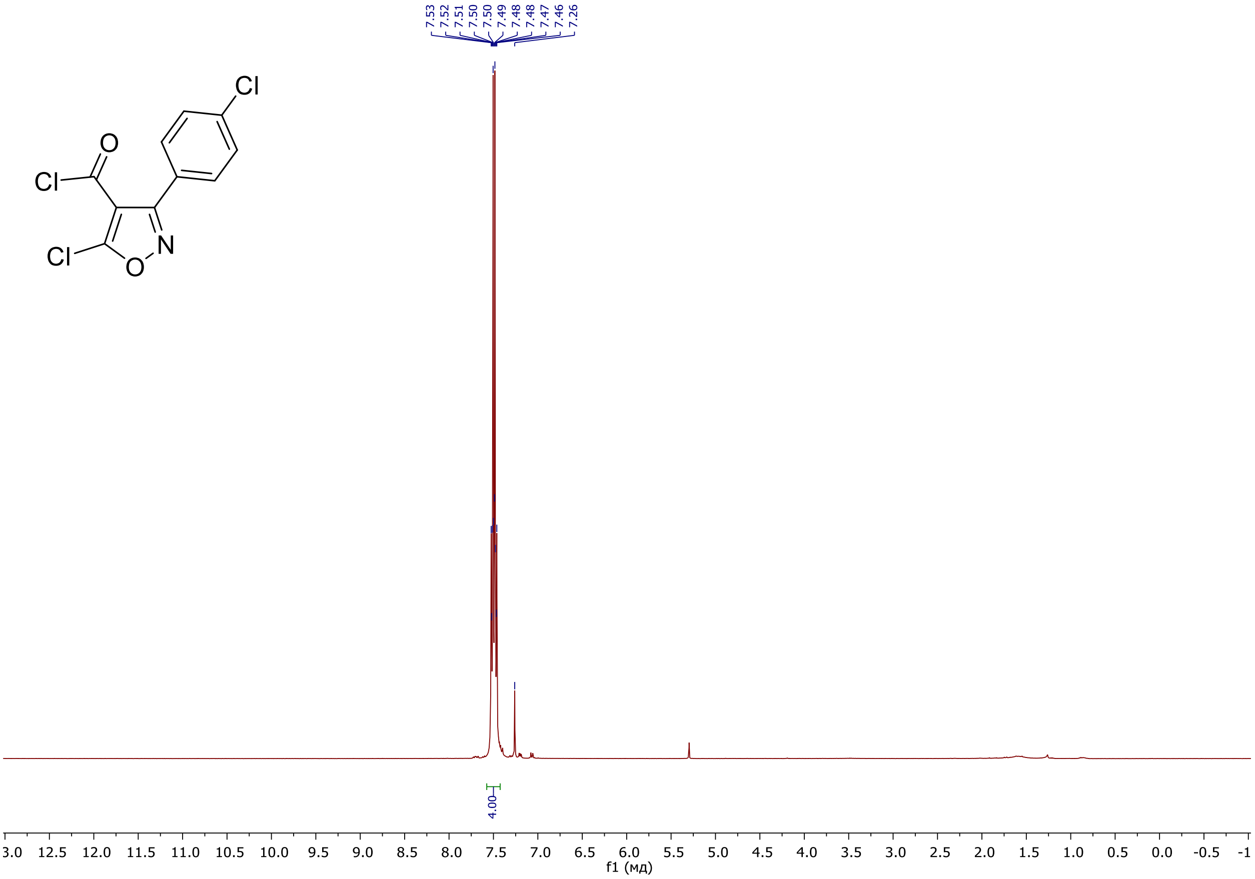

^{13}C{^{1}H} NMR spectrum (100 MHz, $CDCl_3$) of compound **1f**

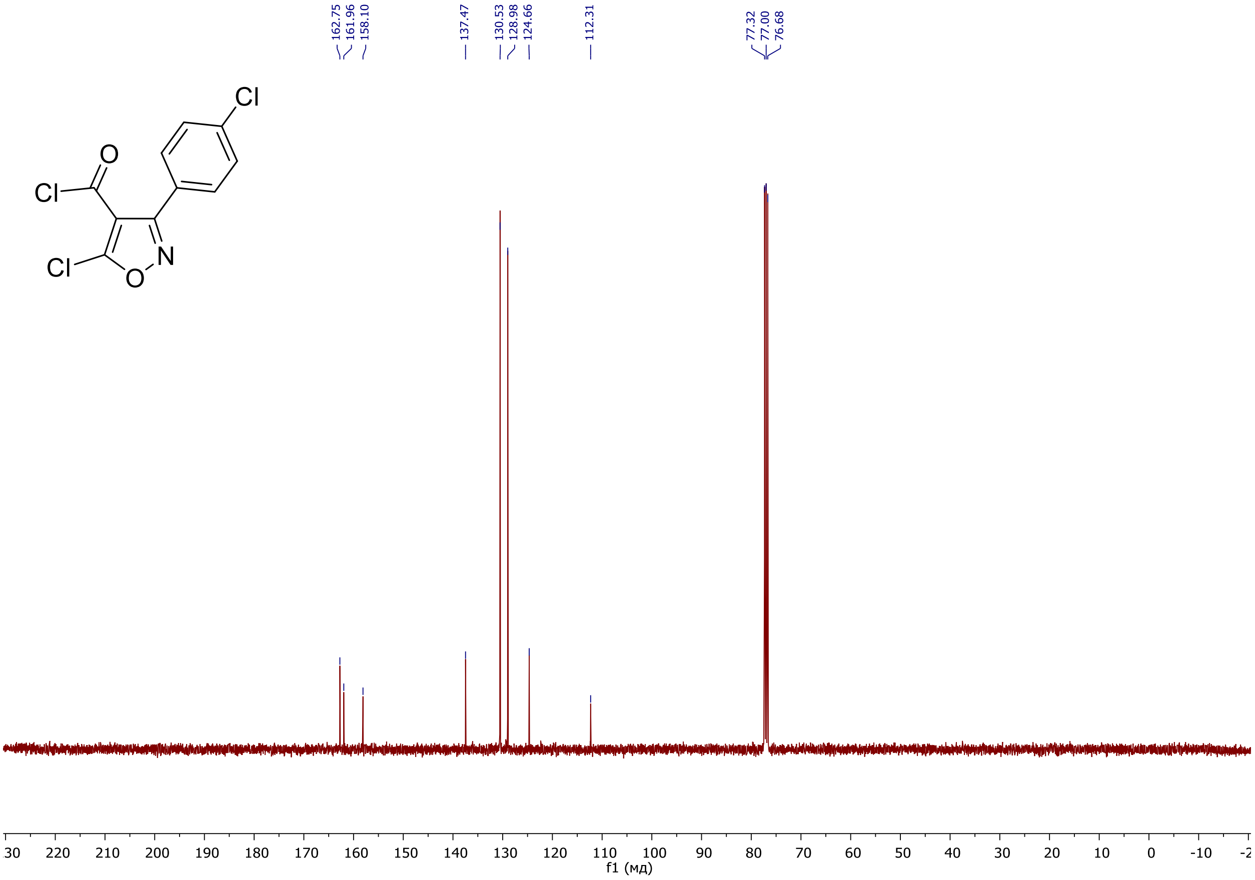

^{1}H NMR spectrum (400 MHz, $CDCl_3$) of compound **1g**

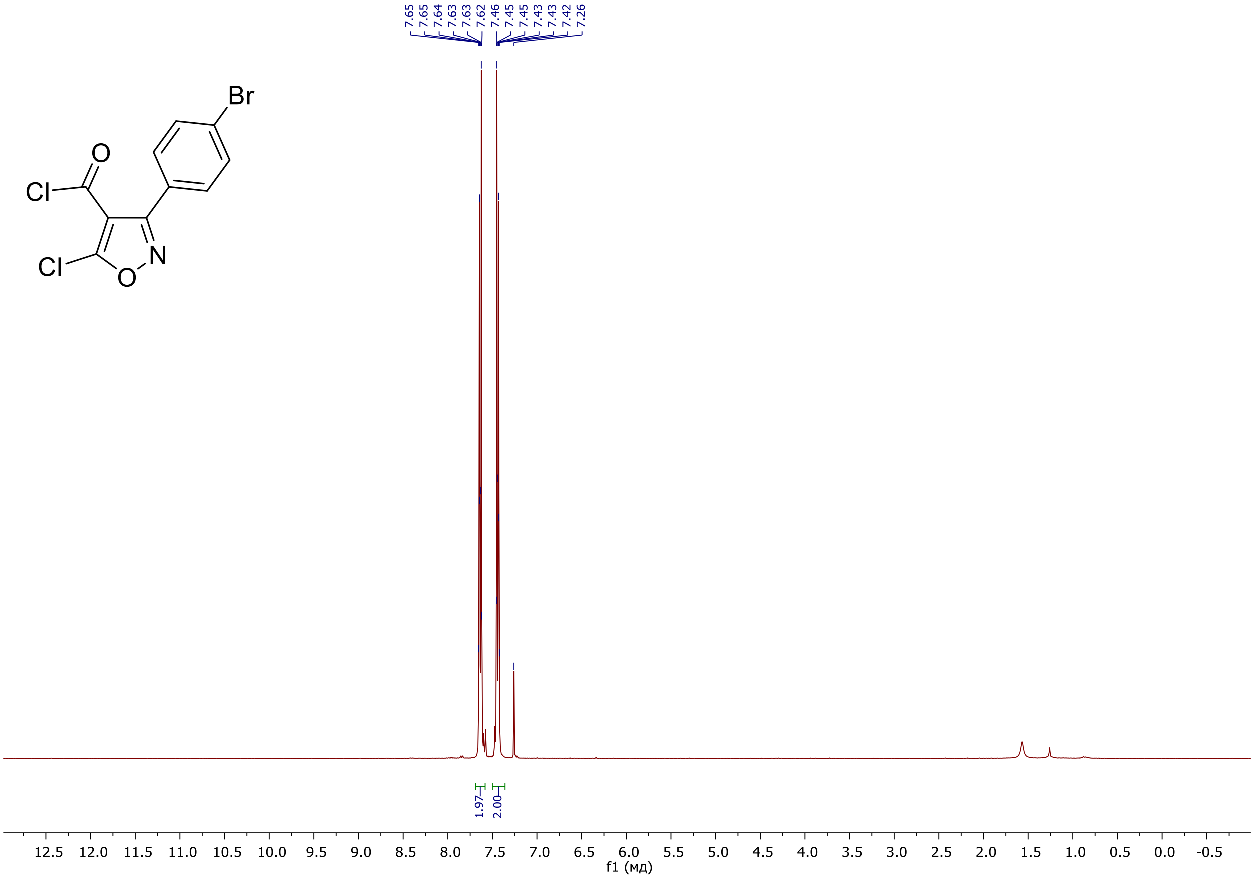

$^{13}C\{^{1}H\}$ NMR spectrum (100 MHz, $CDCl_3$) of compound **1g**

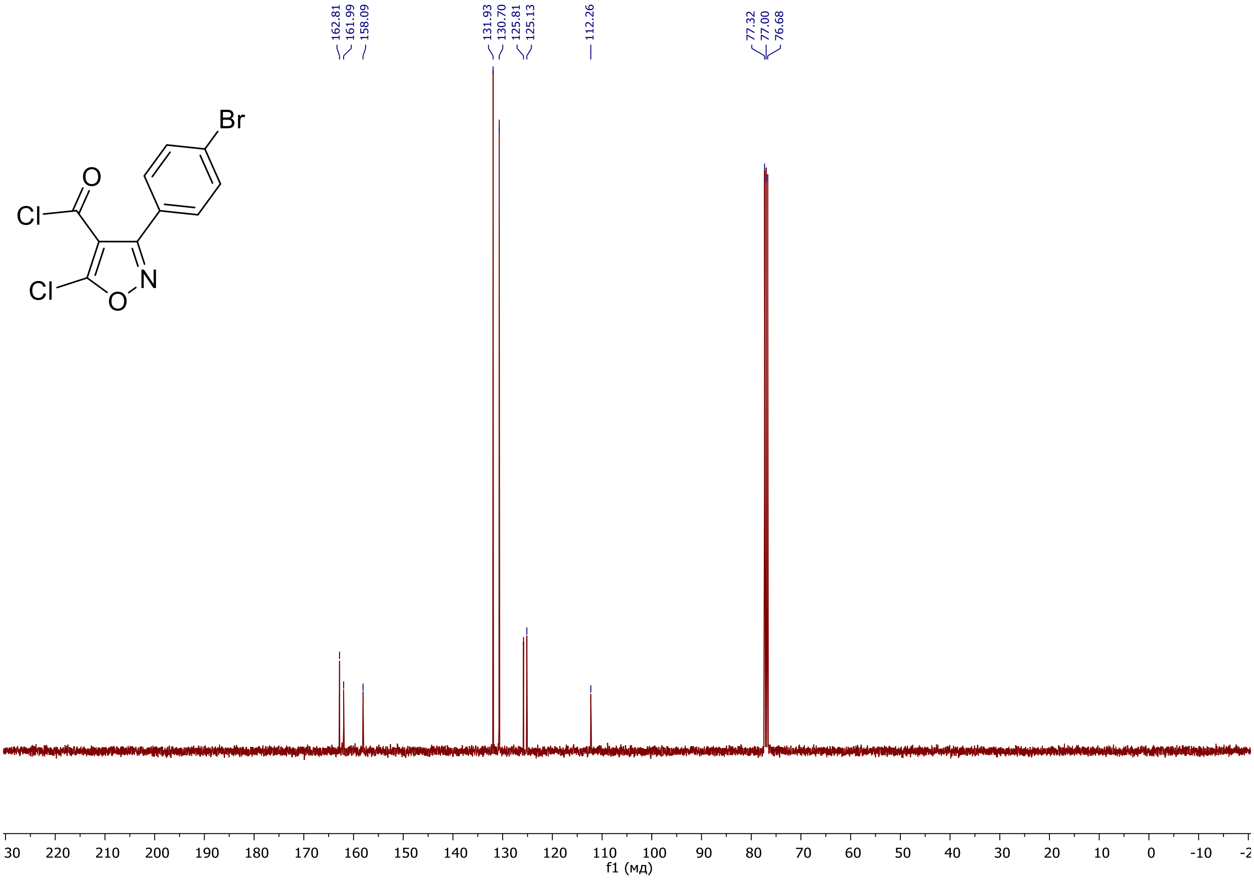

^{1}H NMR spectrum (400 MHz, $CDCl_3$) of compound **1h**

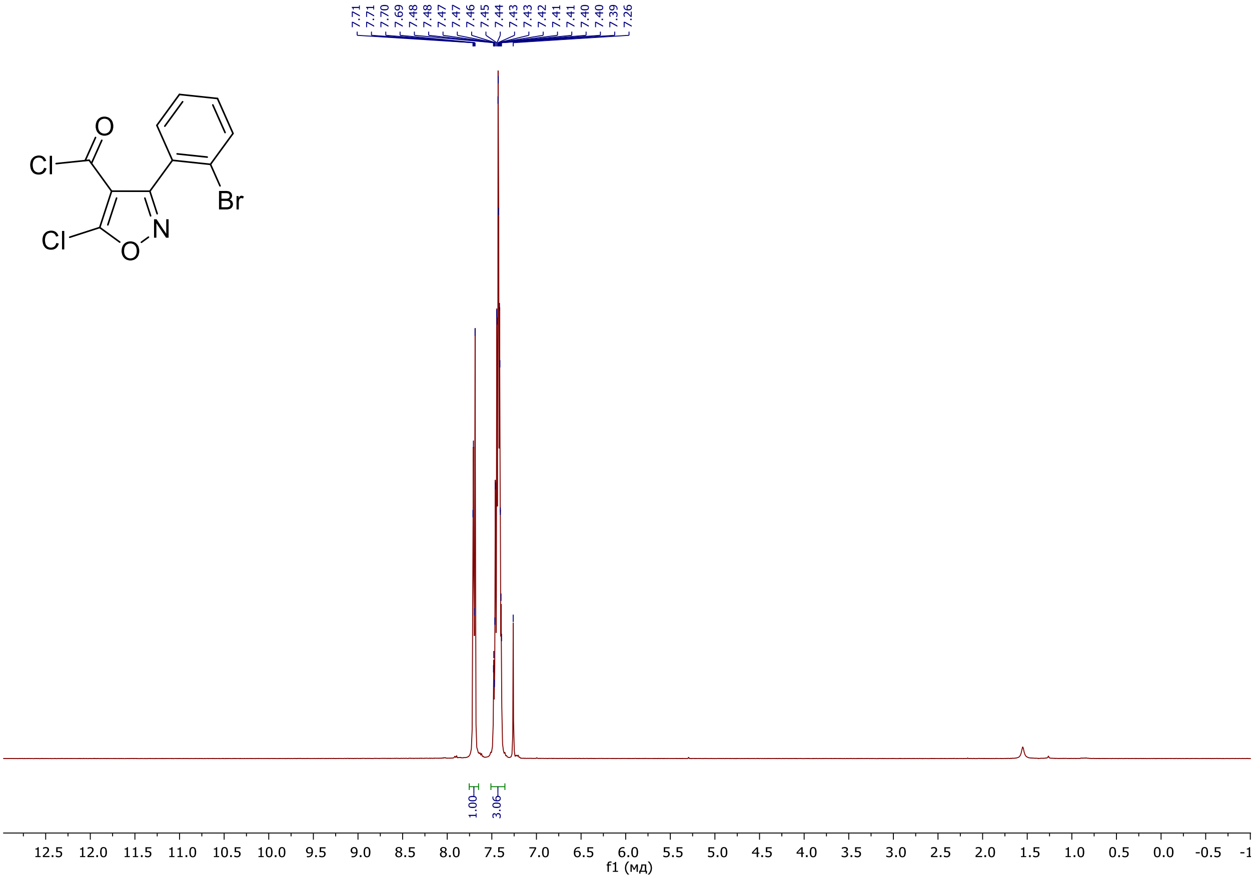

^{13}C{^{1}H} NMR spectrum (100 MHz, $CDCl_3$) of compound **1h**

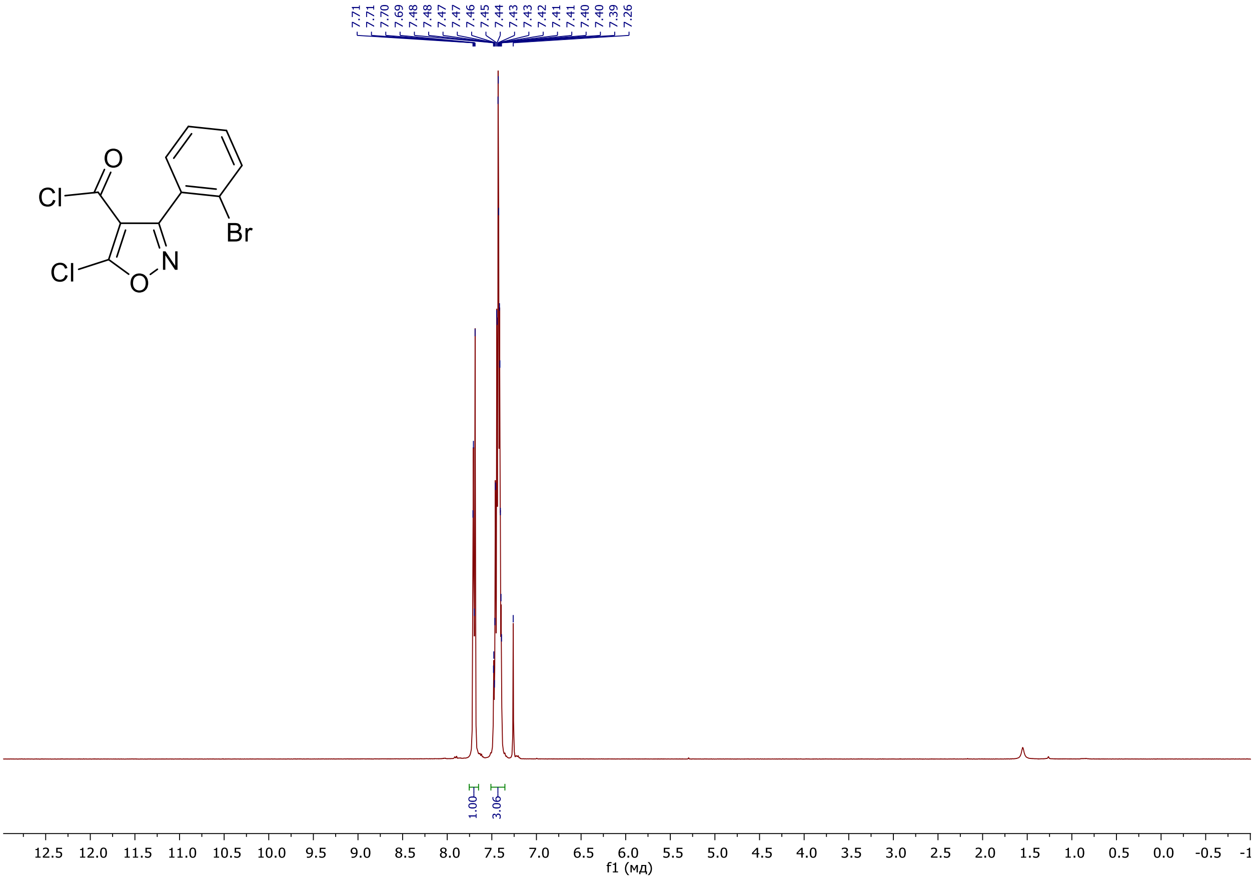

^{1}H NMR spectrum (400 MHz, $CDCl_3$) of compound **1i**

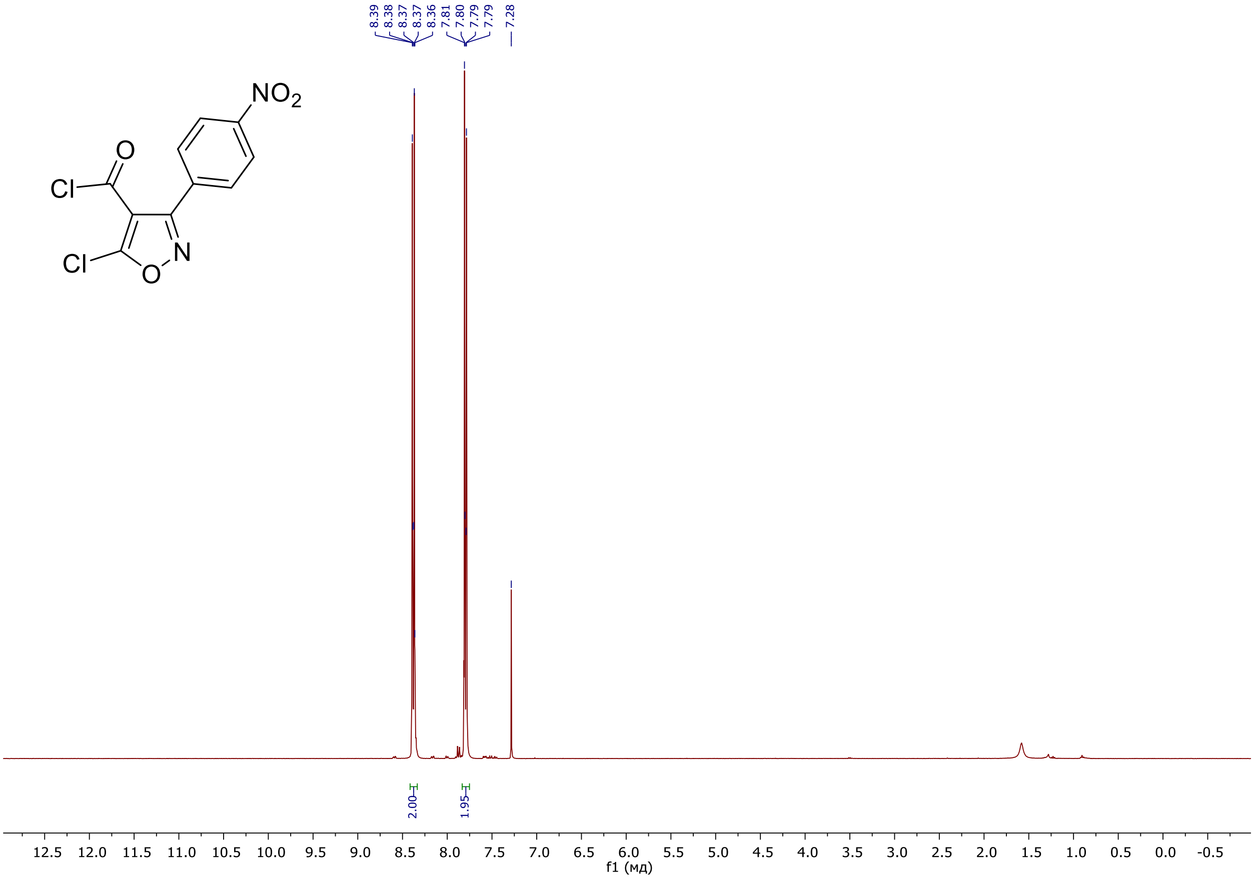

$^{13}C\{^{1}H\}$ NMR spectrum (100 MHz, $CDCl_3$) of compound **1i**

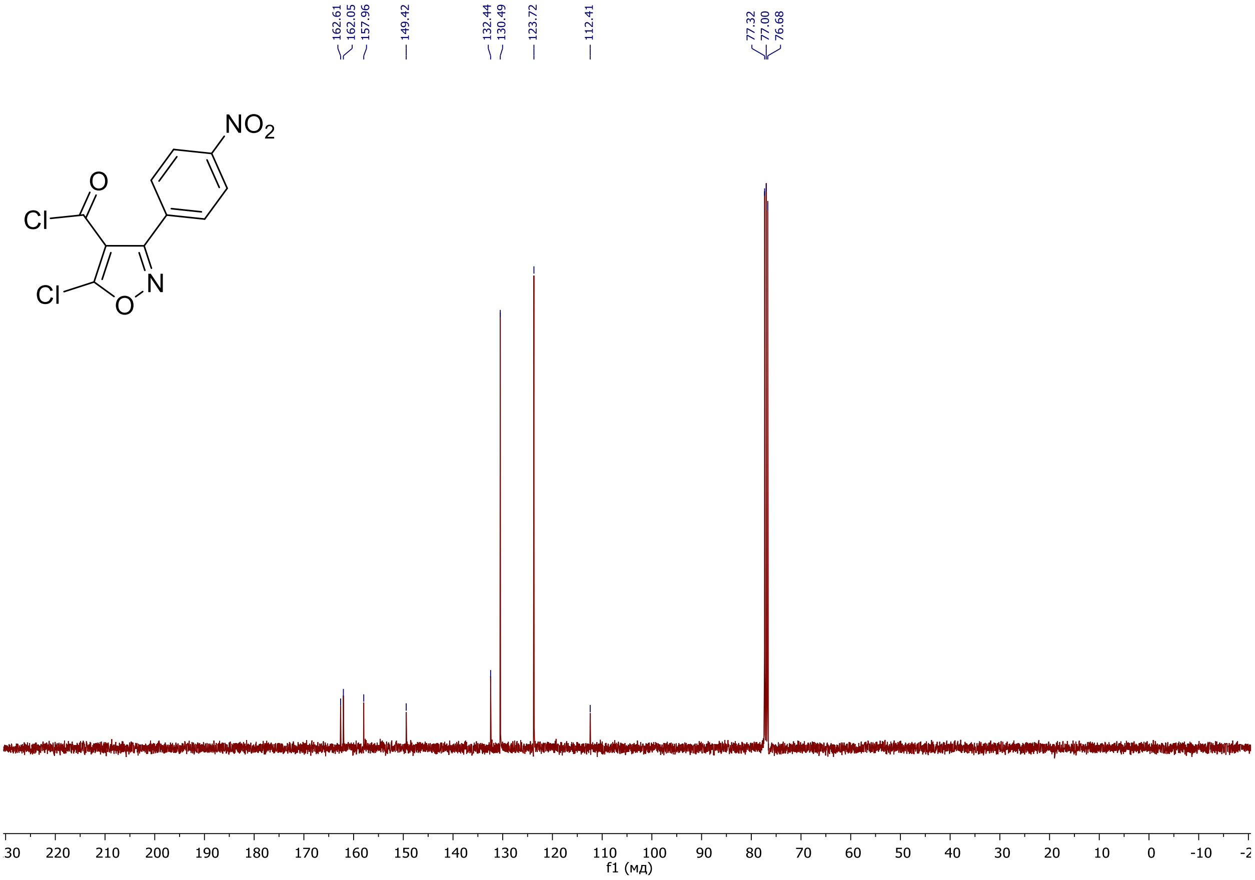

^{1}H NMR spectrum (400 MHz, $CDCl_3$) of compound **1j**

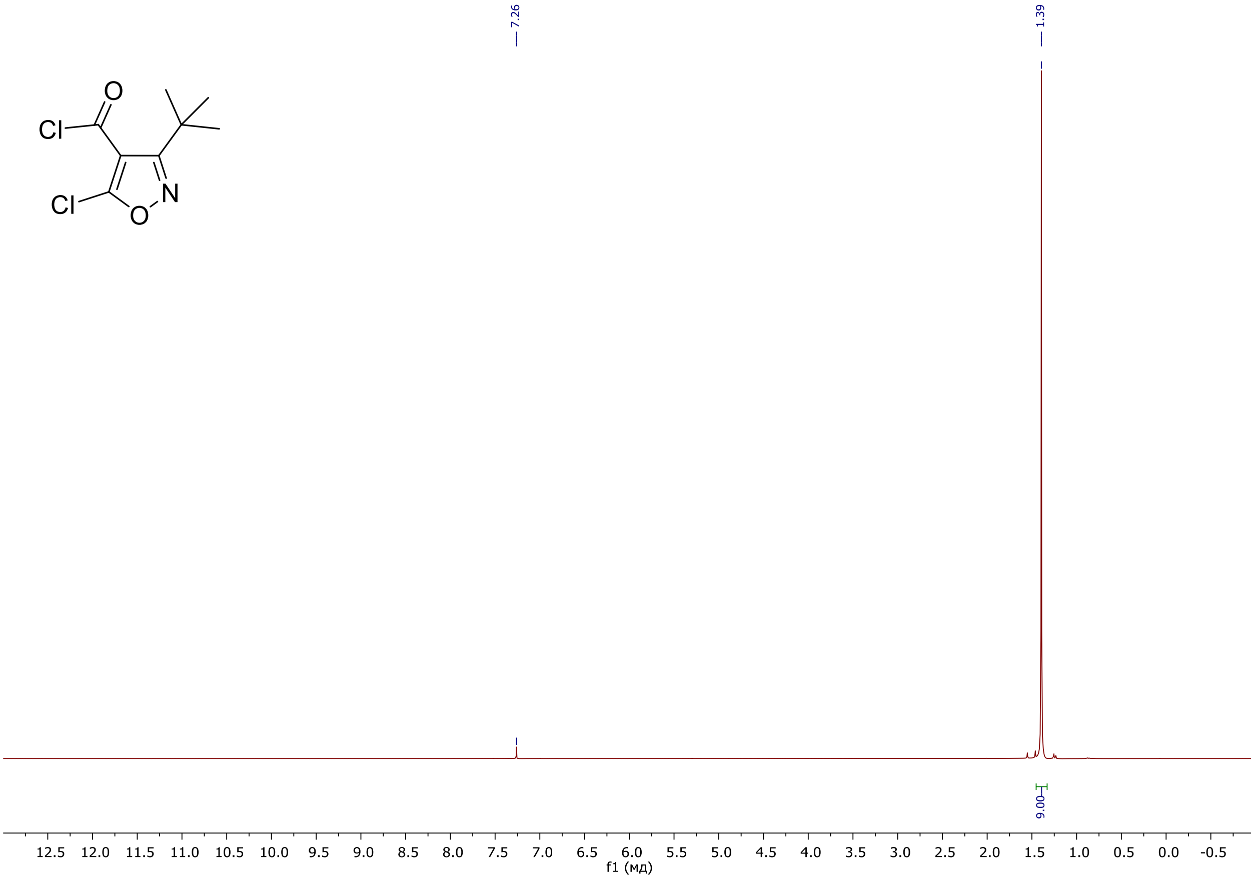

^{13}C{^{1}H} NMR spectrum (100 MHz, $CDCl_3$) of compound **1j**

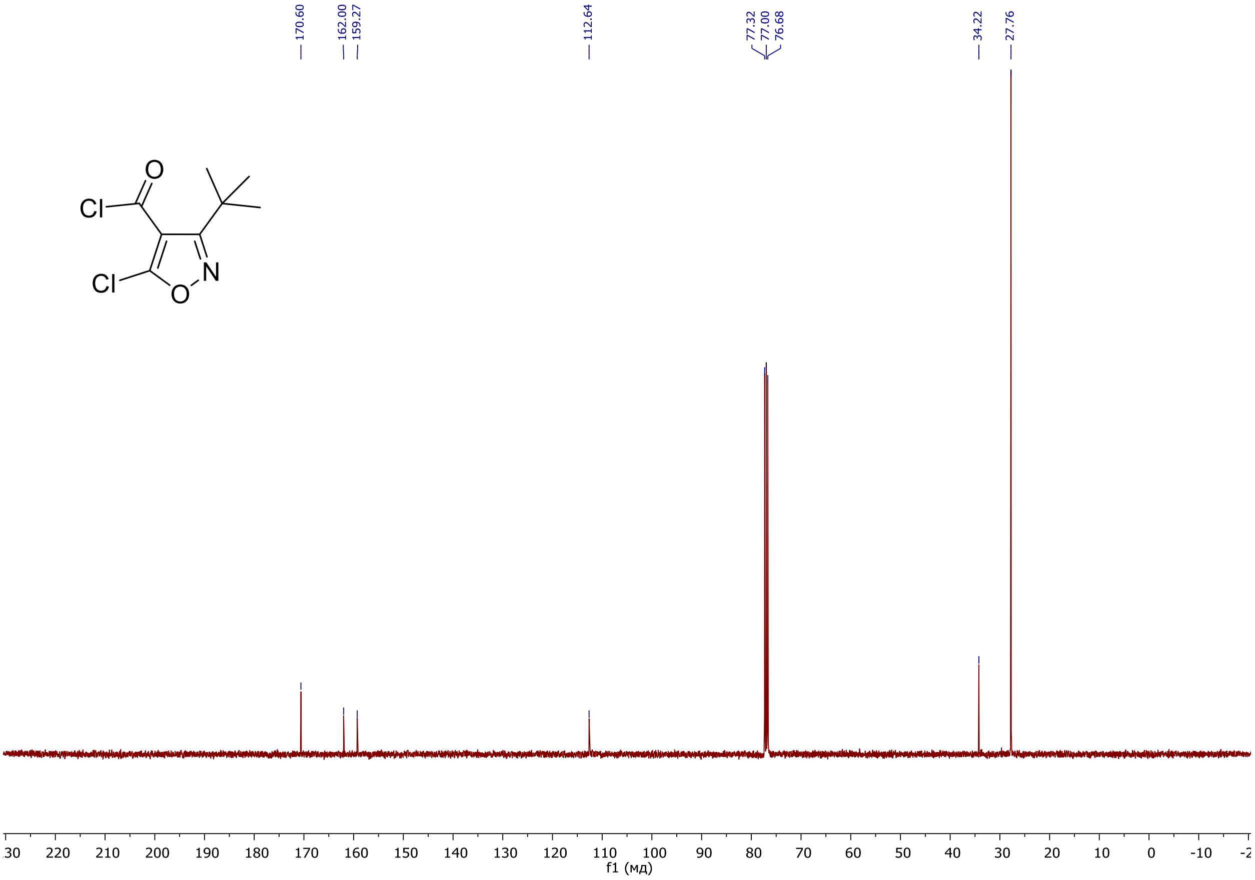

^{1}H NMR spectrum (400 MHz, DMSO-d_6) of compound **6a**

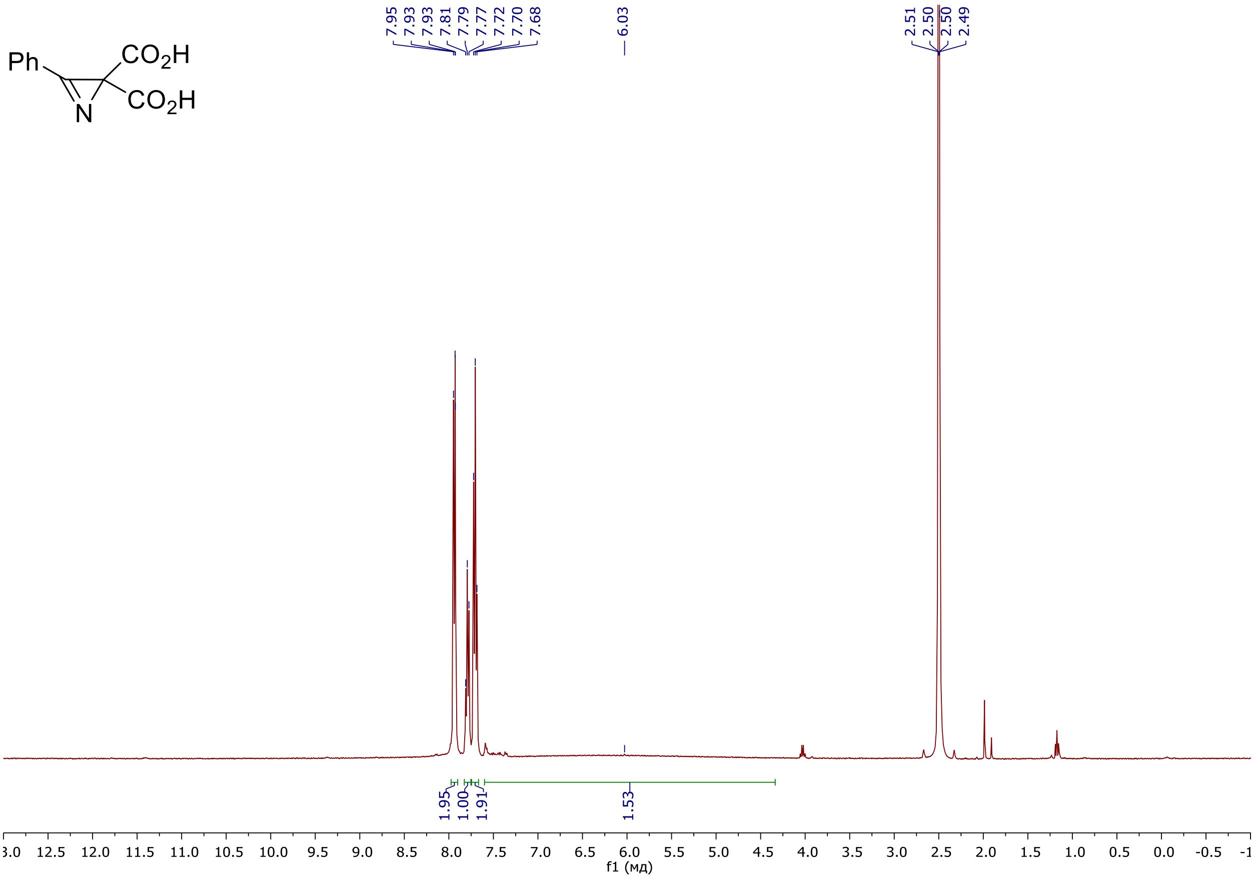

$^{13}C\{^{1}H\}$ NMR spectrum (100 MHz, DMSO-d_6) of compound **6a**

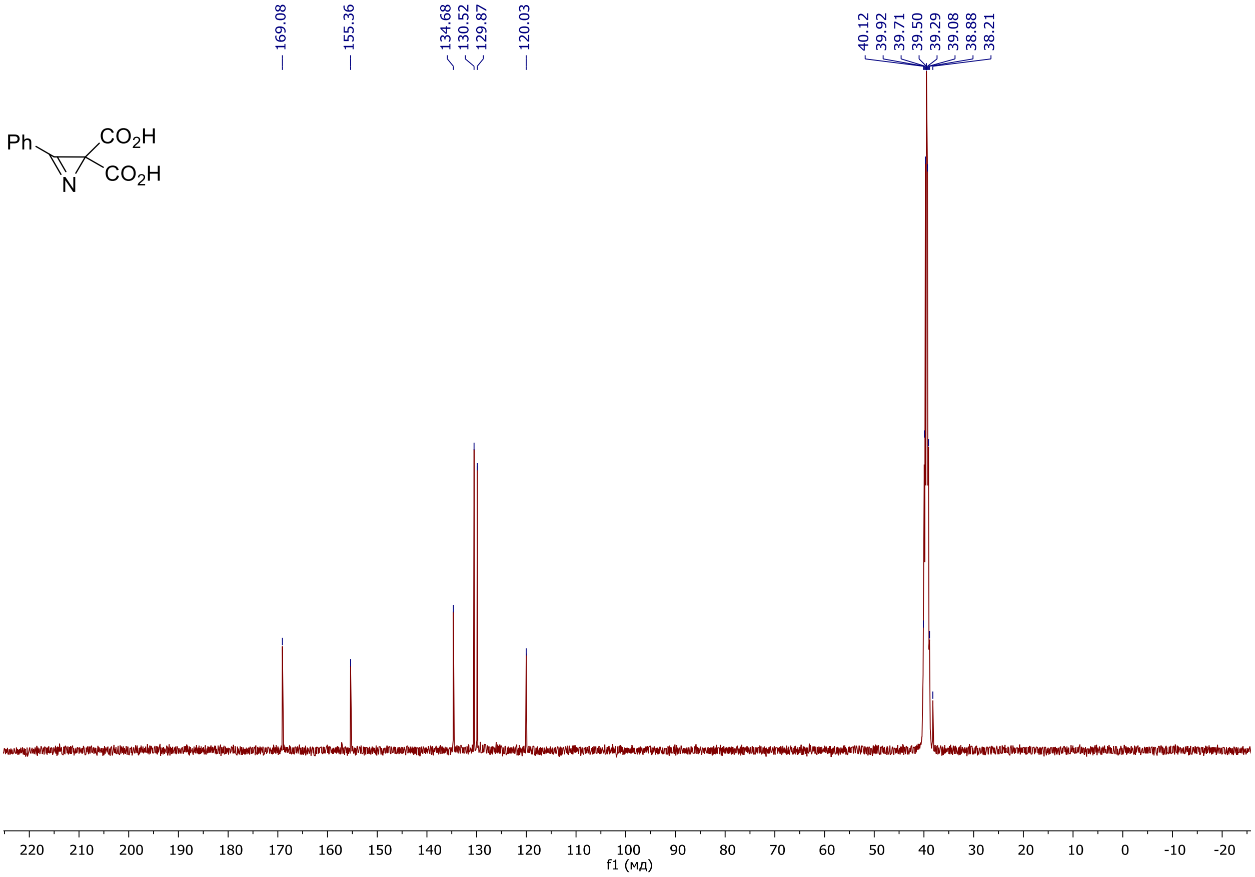

^{1}H NMR spectrum (400 MHz, DMSO-d_6) of compound **6b**

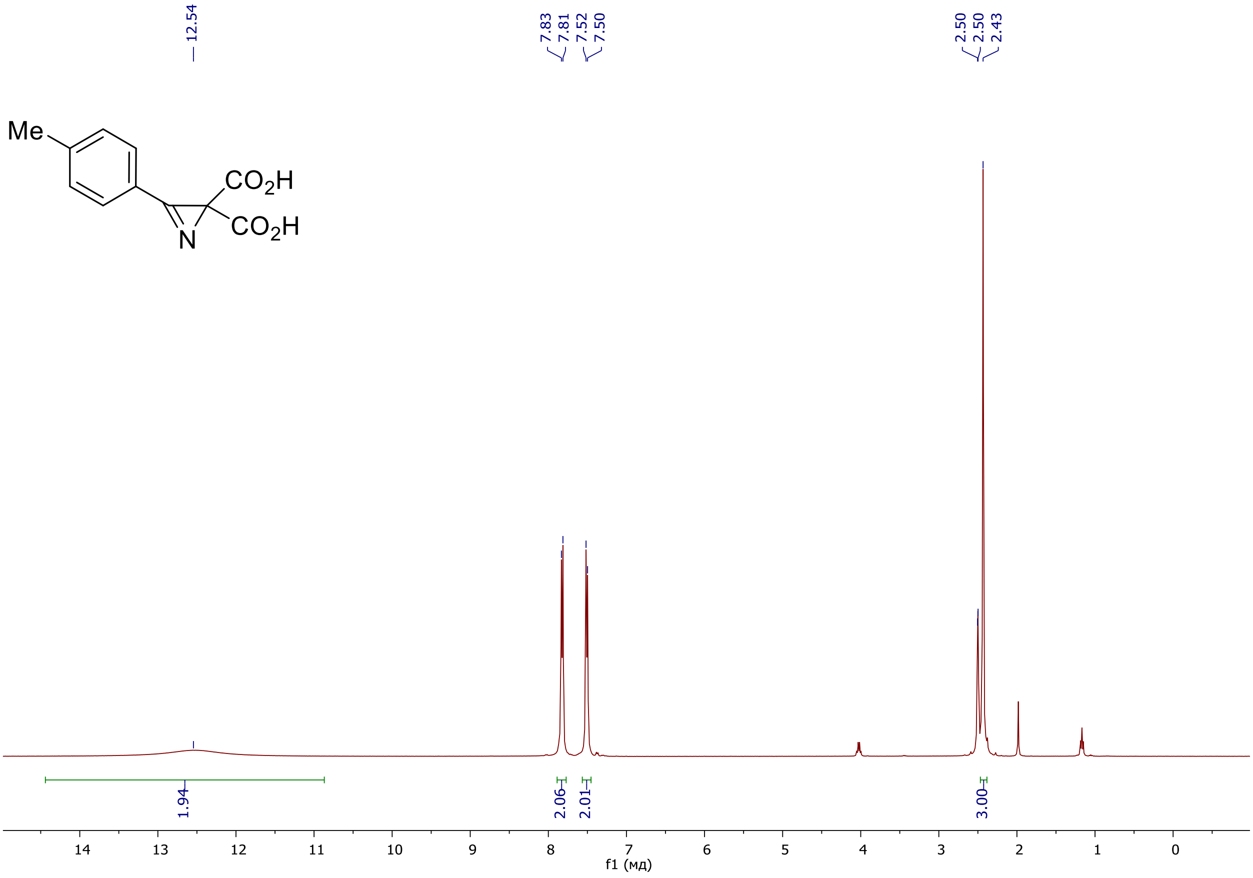

^{13}C{^{1}H} NMR spectrum (100 MHz, DMSO-d_6) of compound **6b**

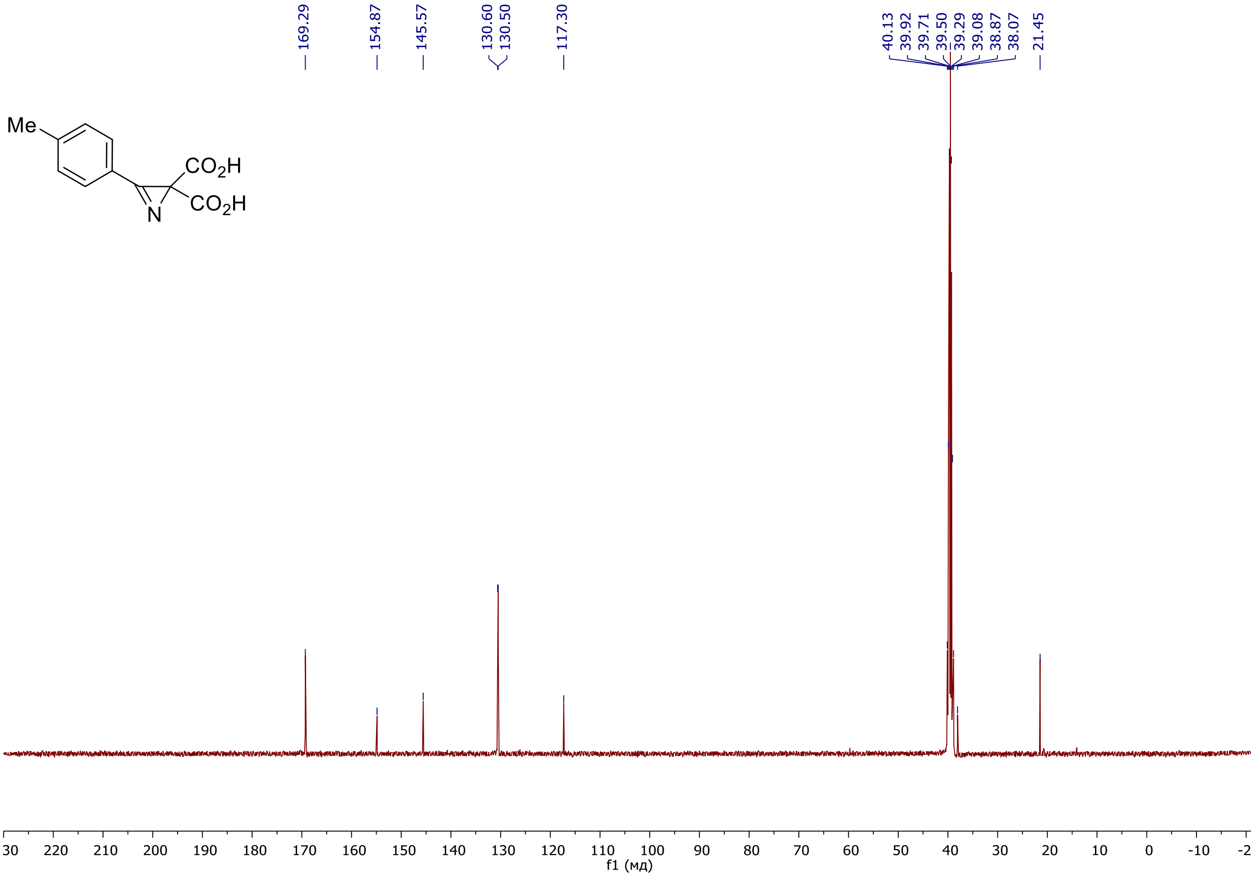

^{1}H NMR spectrum (400 MHz, DMSO-d_6) of compound **6c**

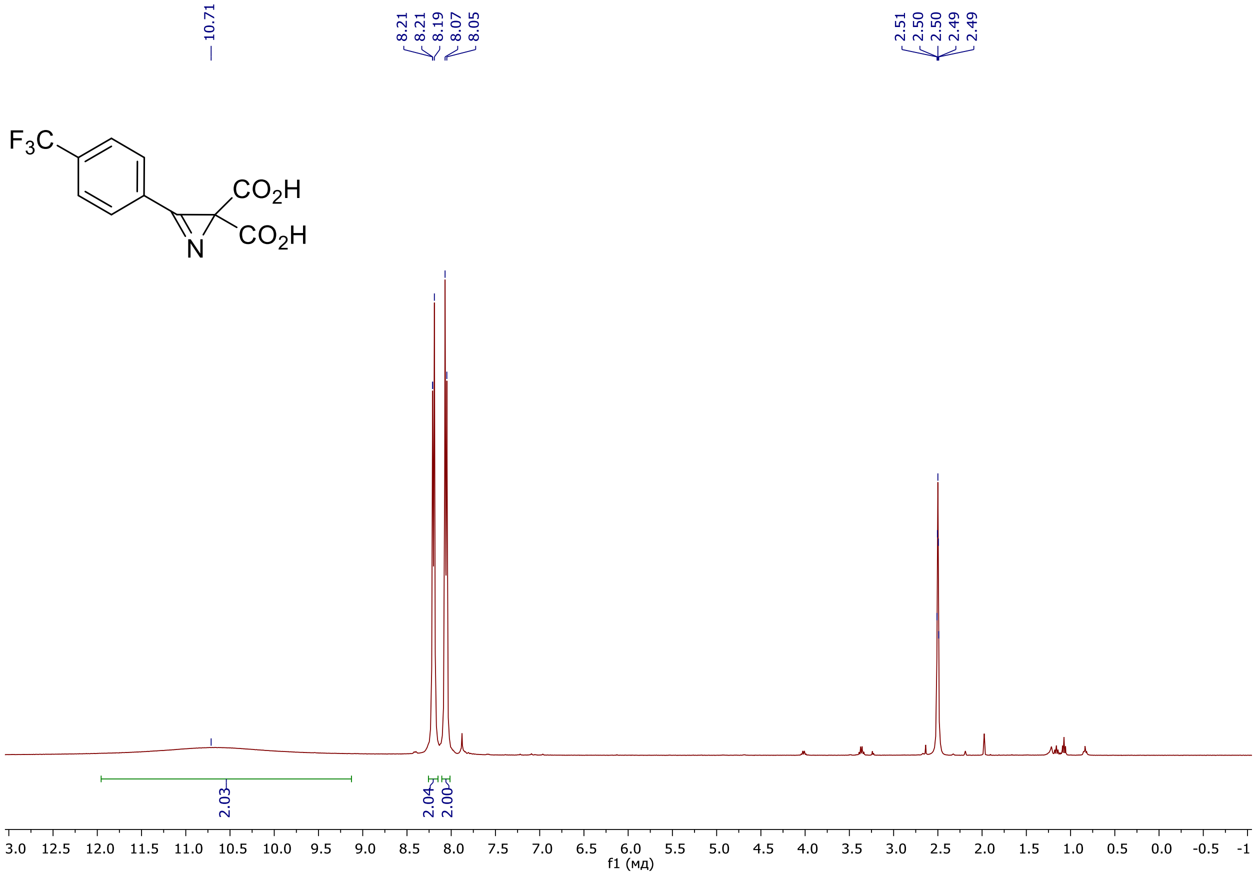

^{13}C{^{1}H} NMR spectrum (100 MHz, DMSO-d_6) of compound **6c**

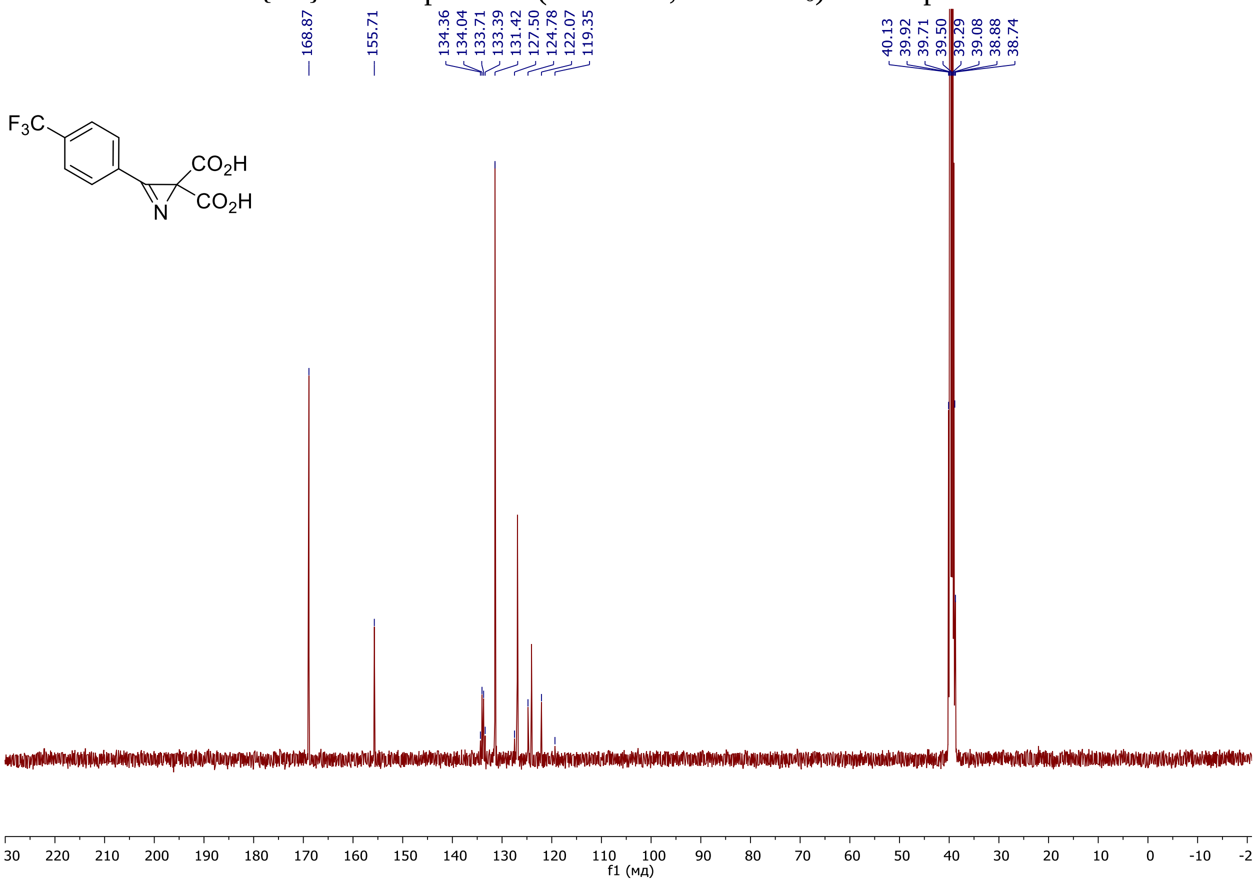

$^{19}F\{^{1}H\}$ NMR spectrum (376 MHz, DMSO-d_6) of compound **6c**

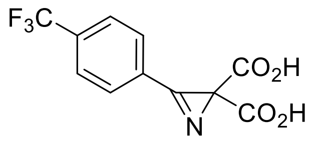

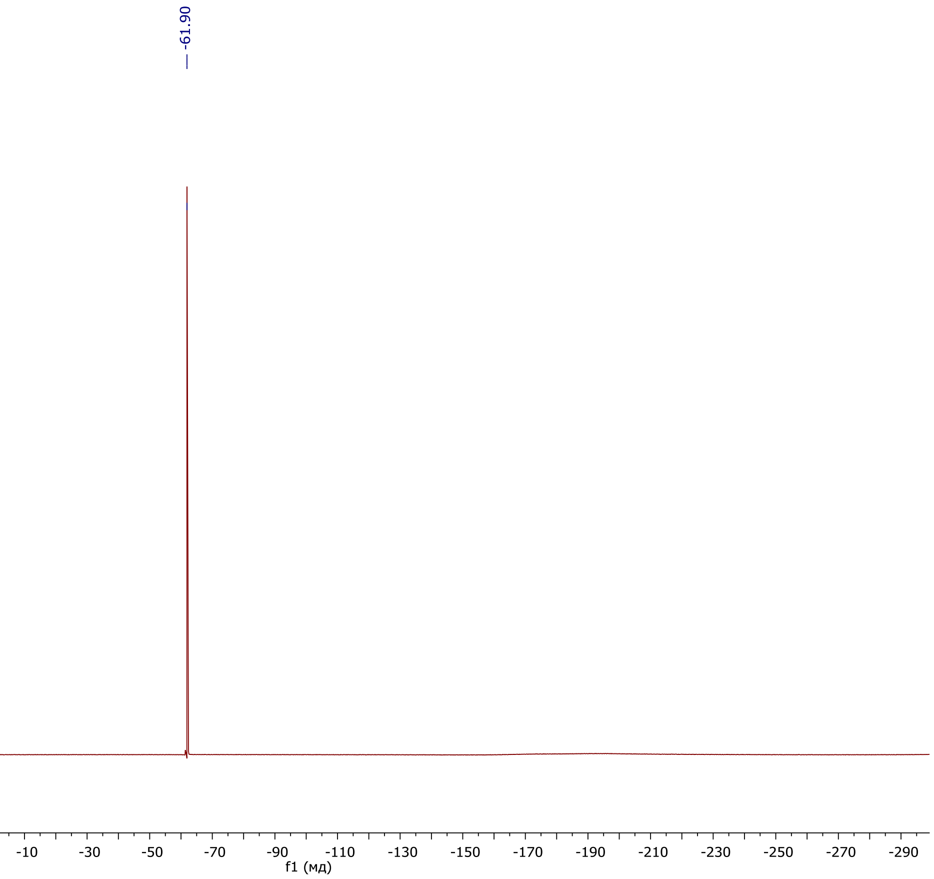

^{1}H NMR spectrum (400 MHz, DMSO-d_6) of compound **6d**

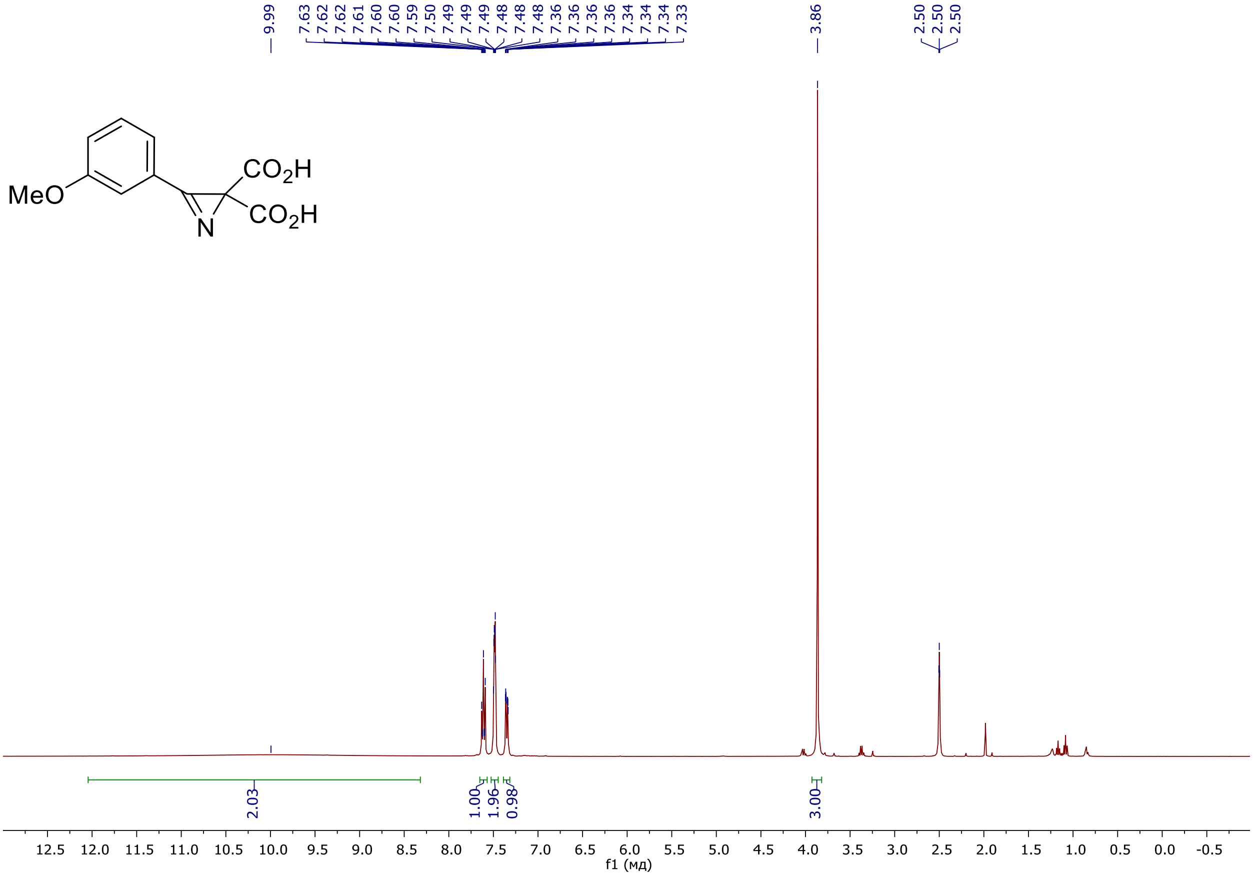

^{13}C{^{1}H} NMR spectrum (100 MHz, DMSO-d_6) of compound **6d**

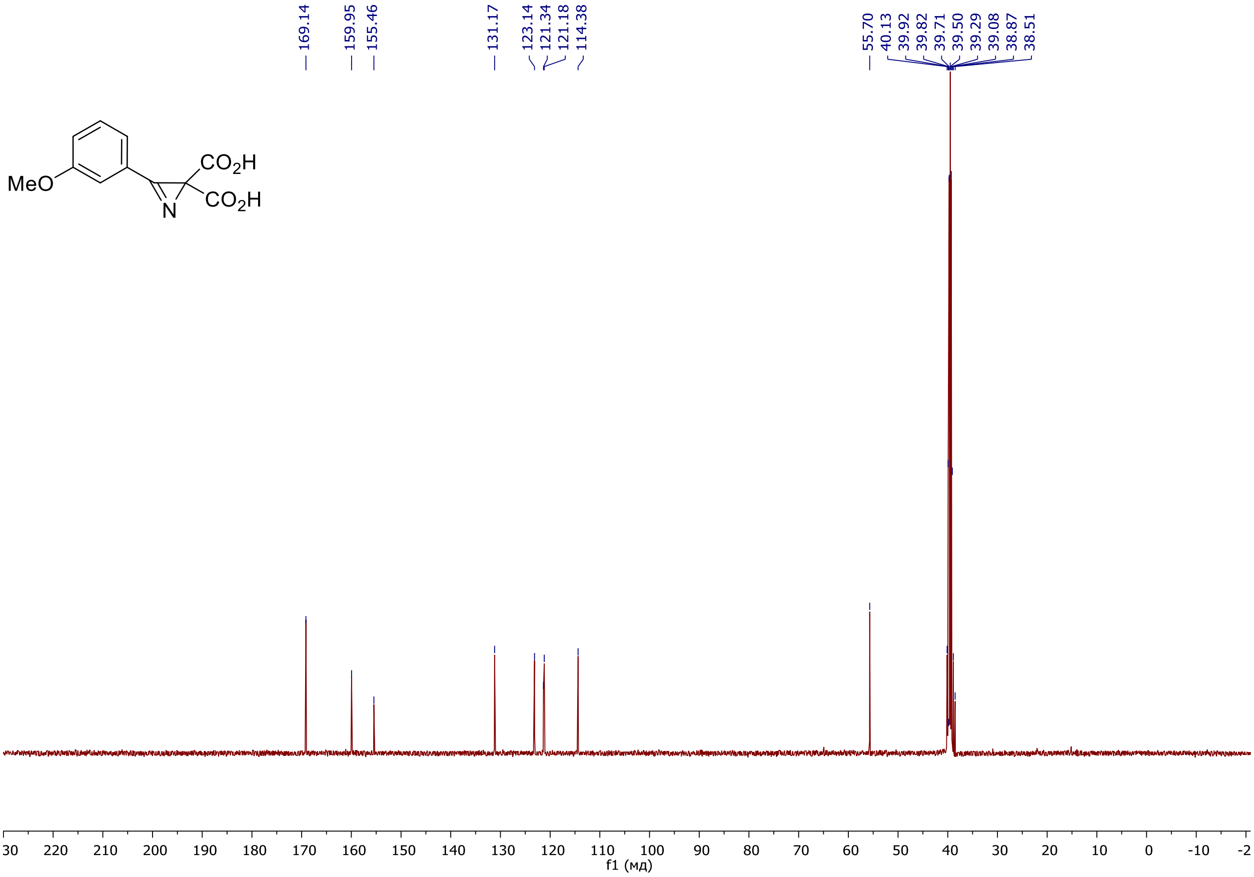

^{1}H NMR spectrum (400 MHz, DMSO-d_6) of compound **6e**

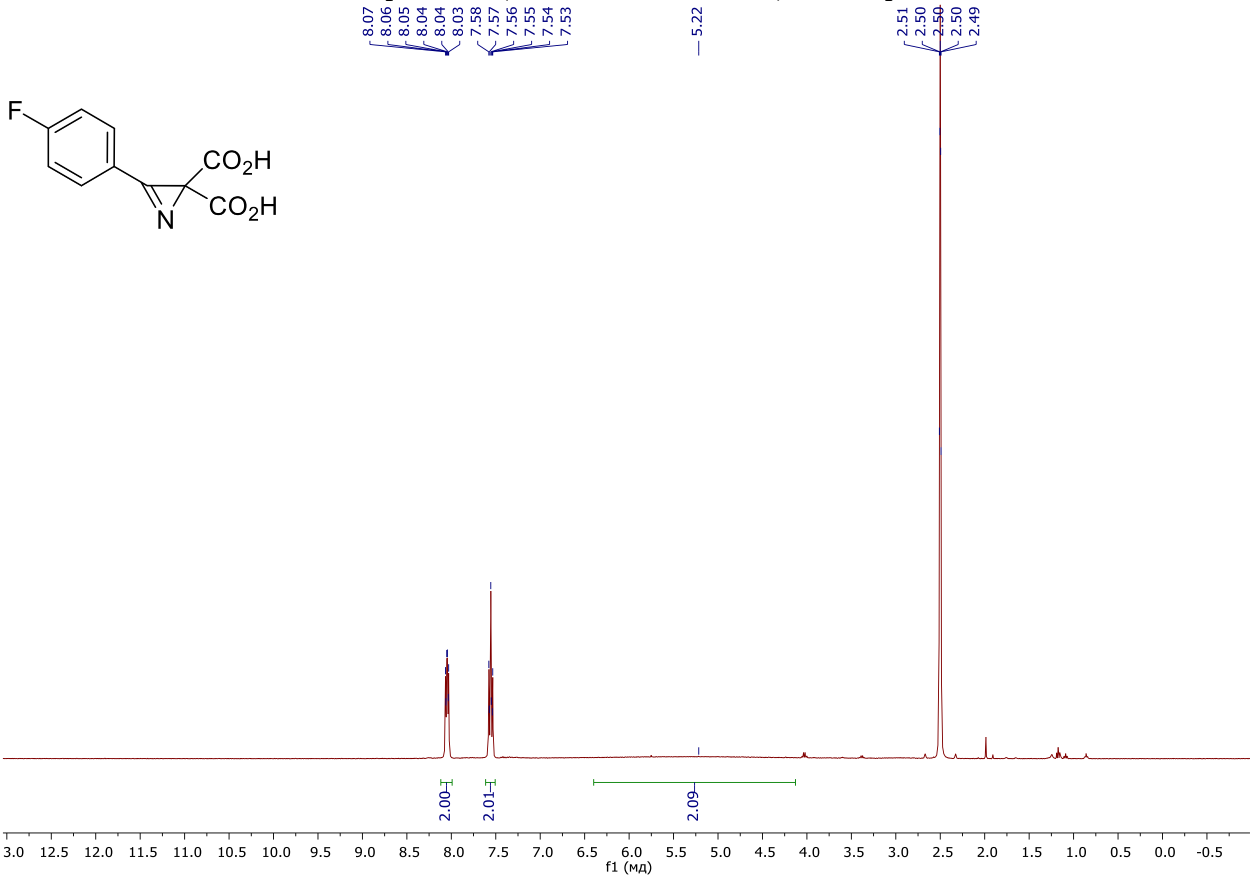

^{13}C{^{1}H} NMR spectrum (100 MHz, DMSO-d_6) of compound **6e**

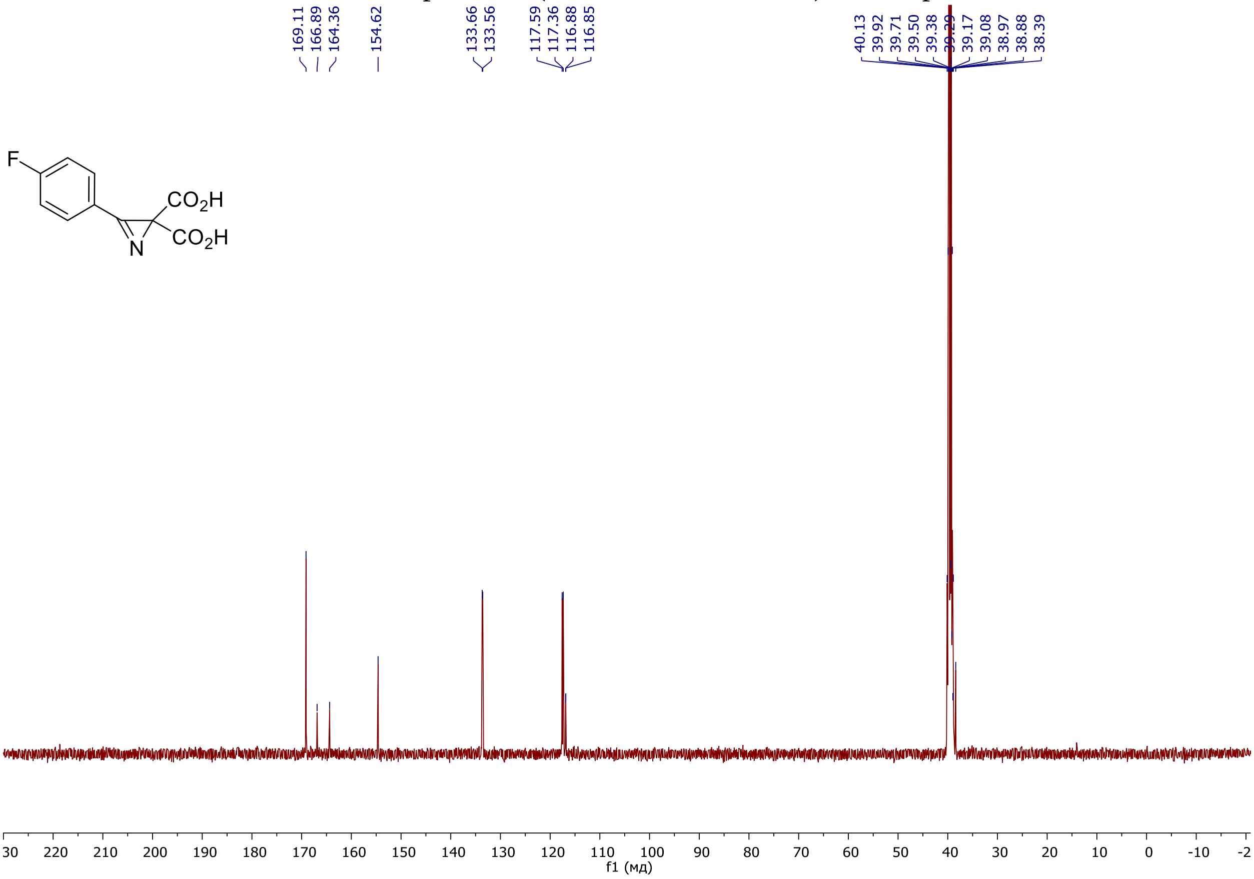

$^{19}F\{^1H\}$ NMR spectrum (376 MHz, DMSO-d_6) of compound **6e**

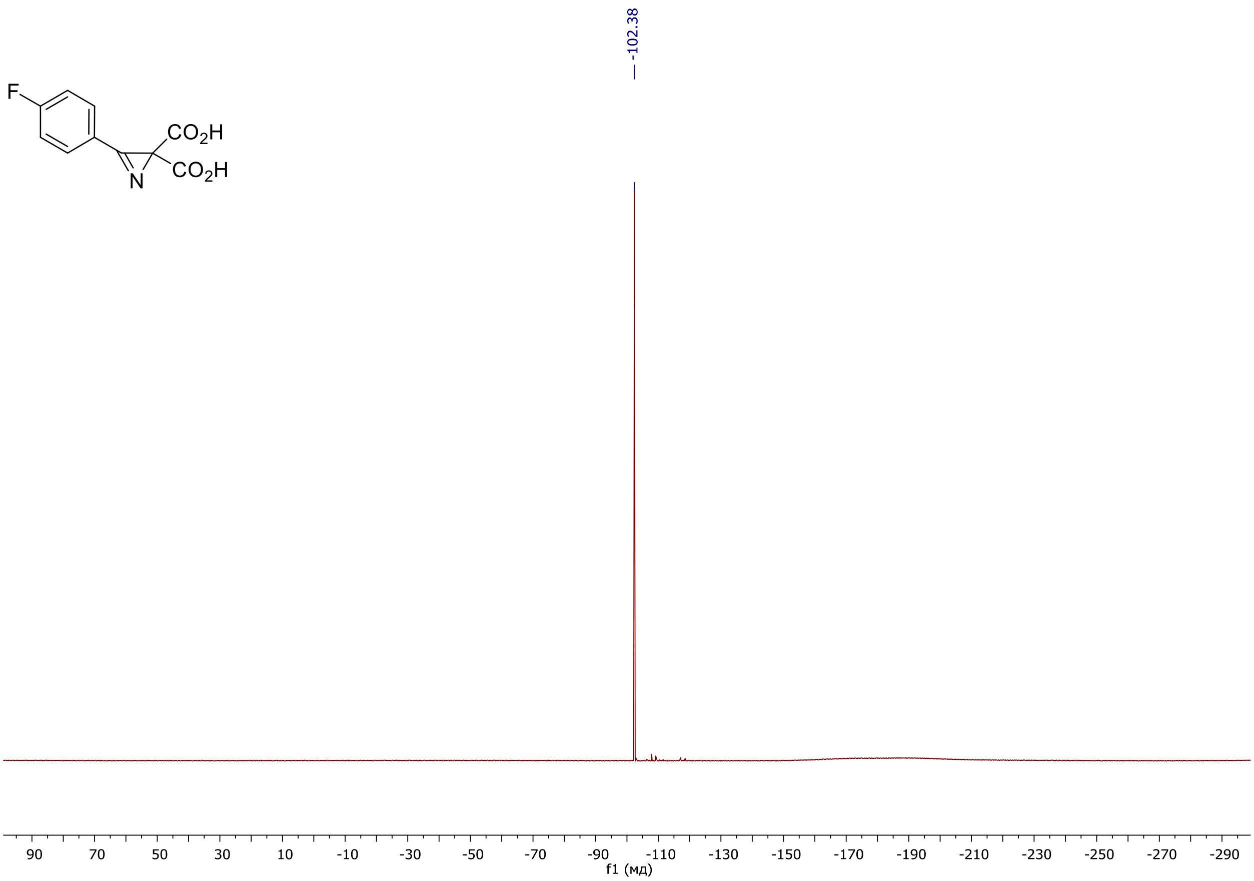

^{1}H NMR spectrum (400 MHz, DMSO-d_6) of compound **6f**

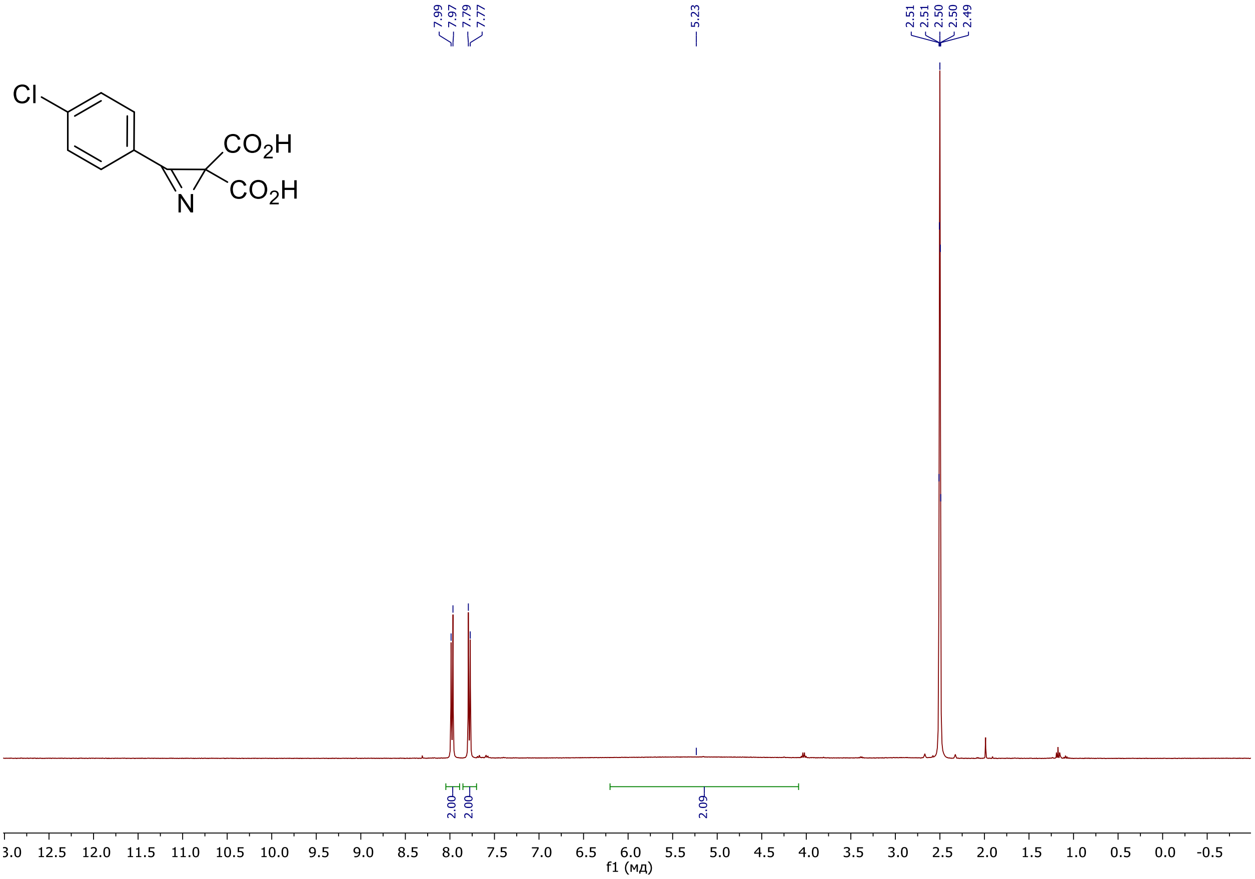

^{13}C{^{1}H} NMR spectrum (100 MHz, DMSO-d_6) of compound **6f**

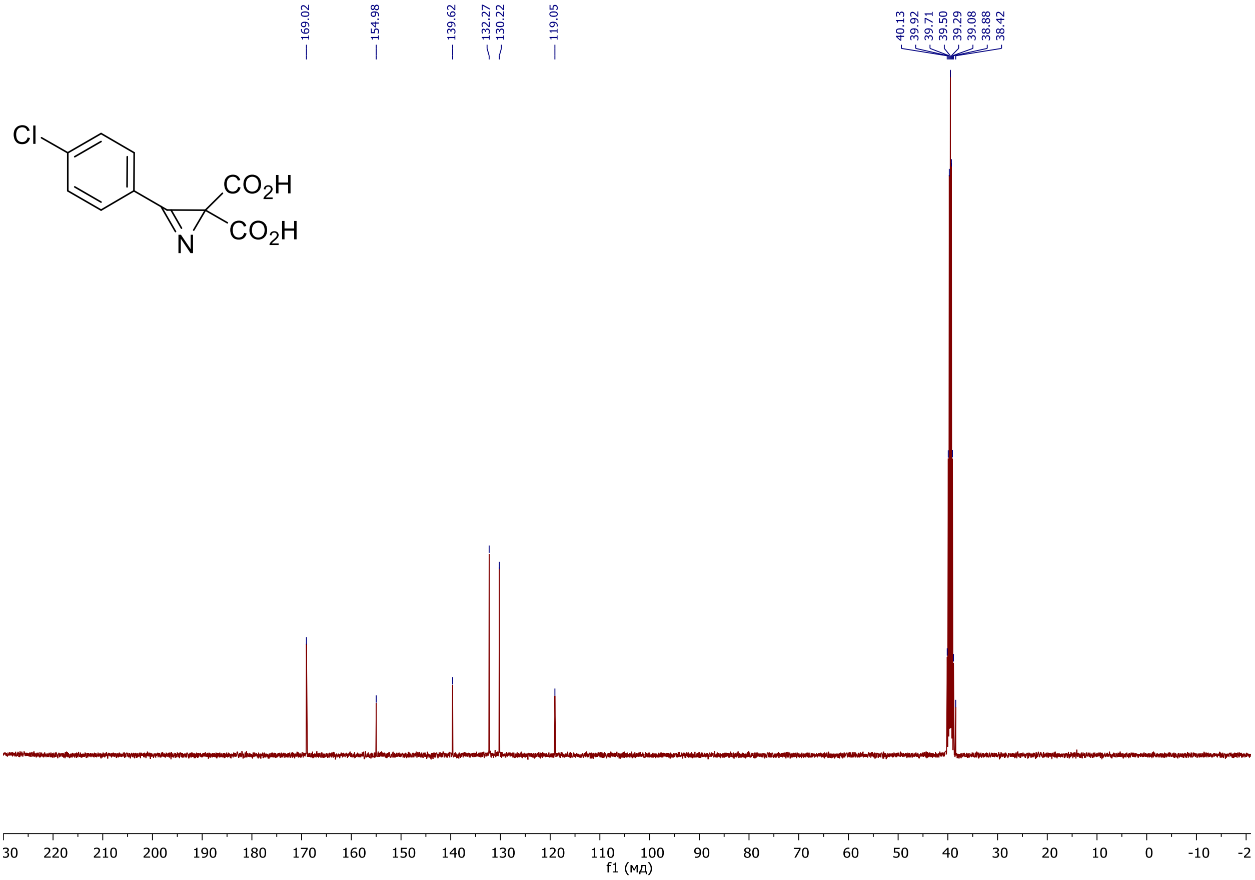

^{1}H NMR spectrum (400 MHz, DMSO-d_6) of compound **6g**

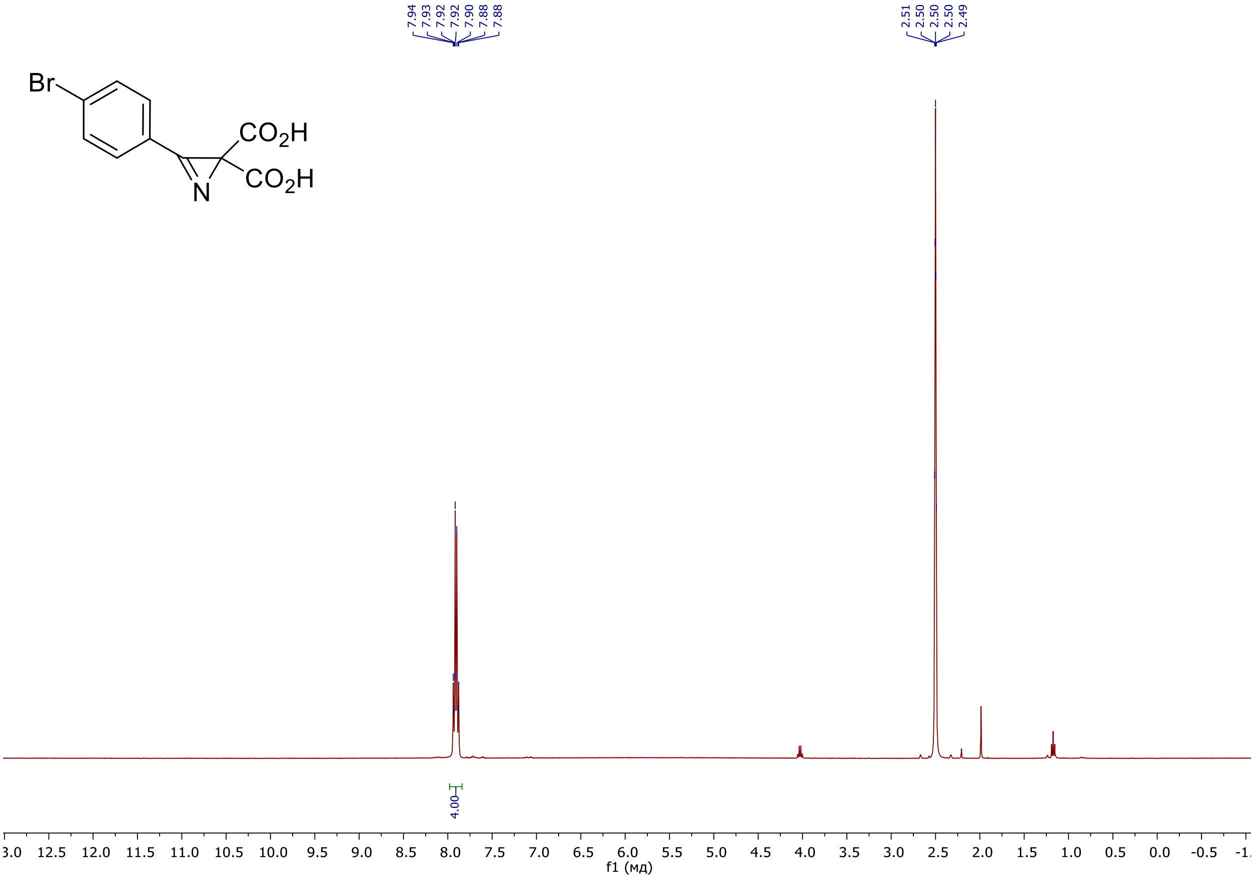

^{13}C{^{1}H} NMR spectrum (100 MHz, DMSO-d_6) of compound **6g**

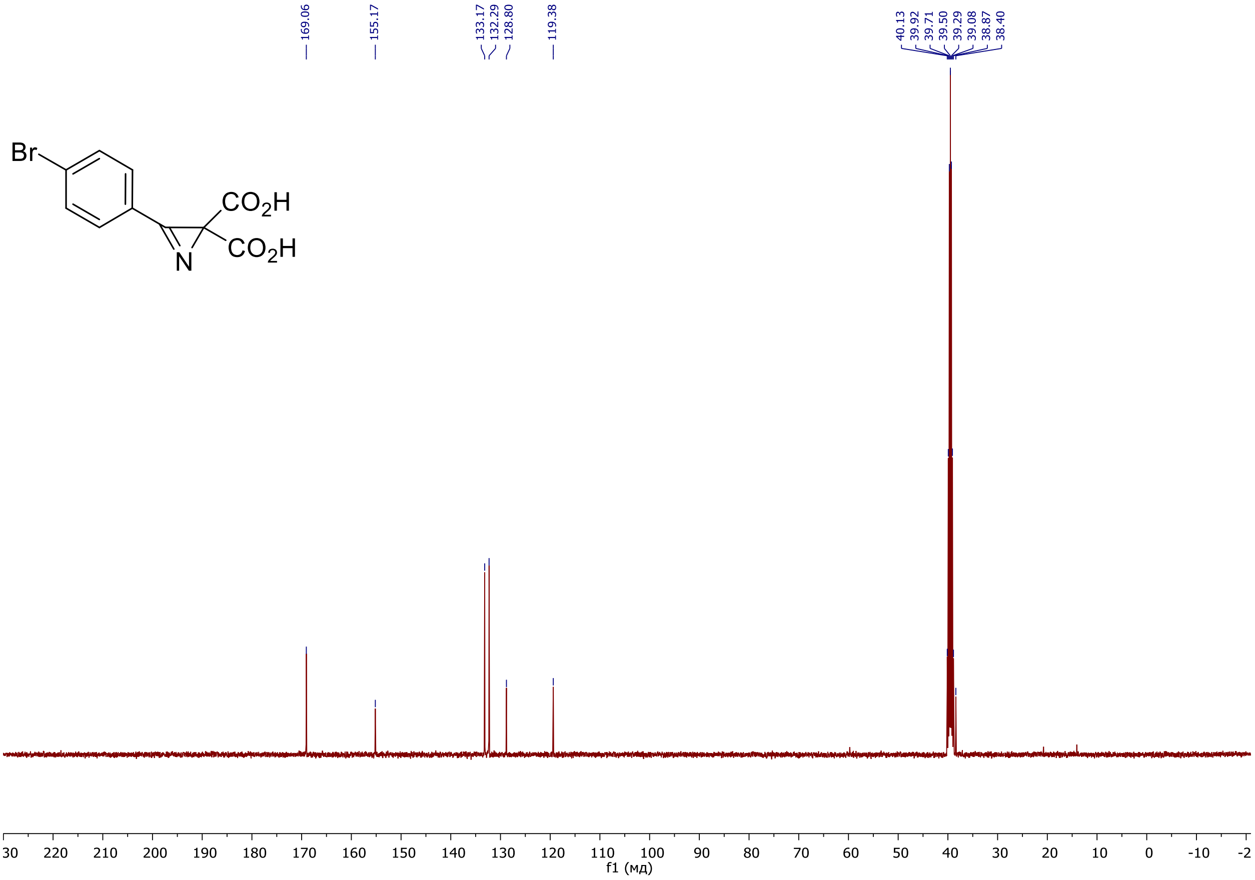

^{1}H NMR spectrum (400 MHz, DMSO-d_6) of compound **6h**

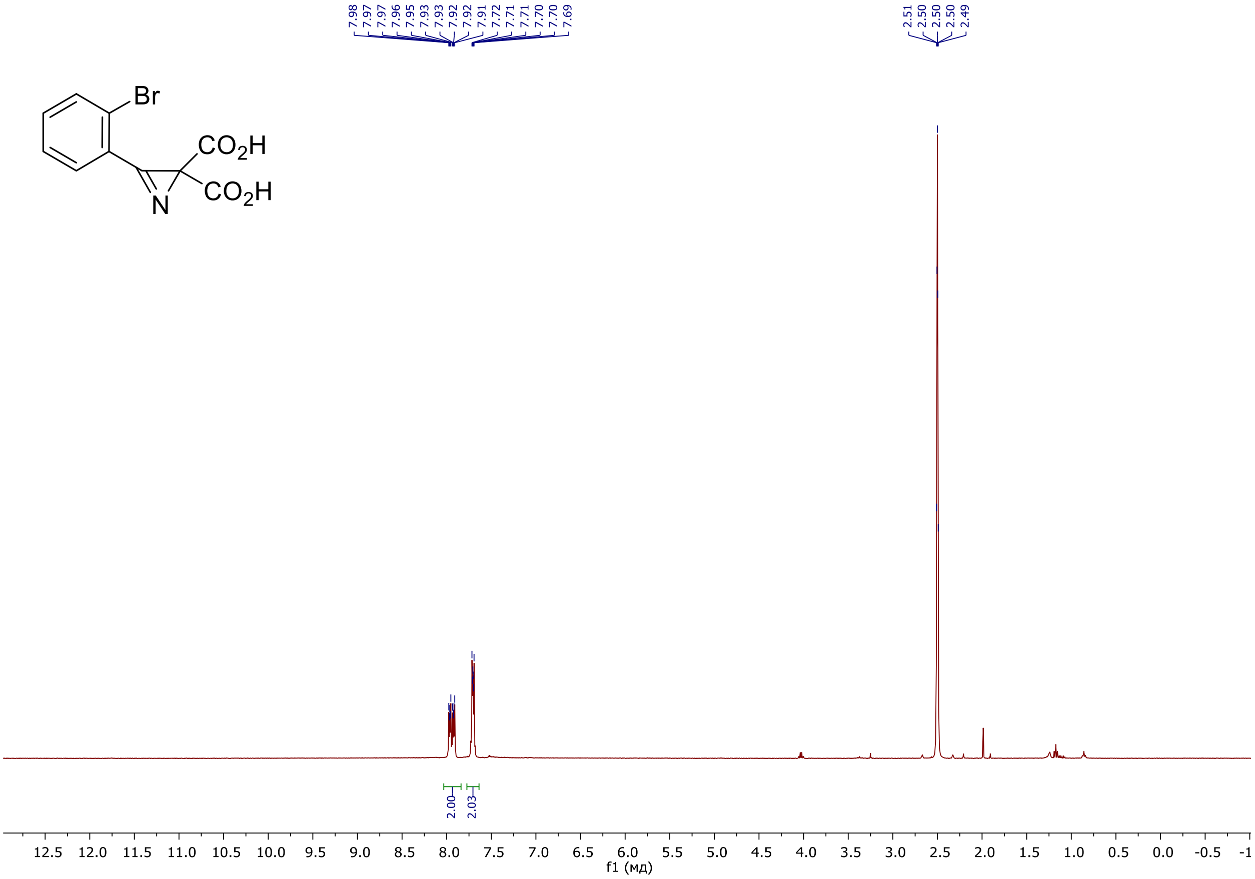

^{13}C{^{1}H} NMR spectrum (100 MHz, DMSO-d_6) of compound **6h**

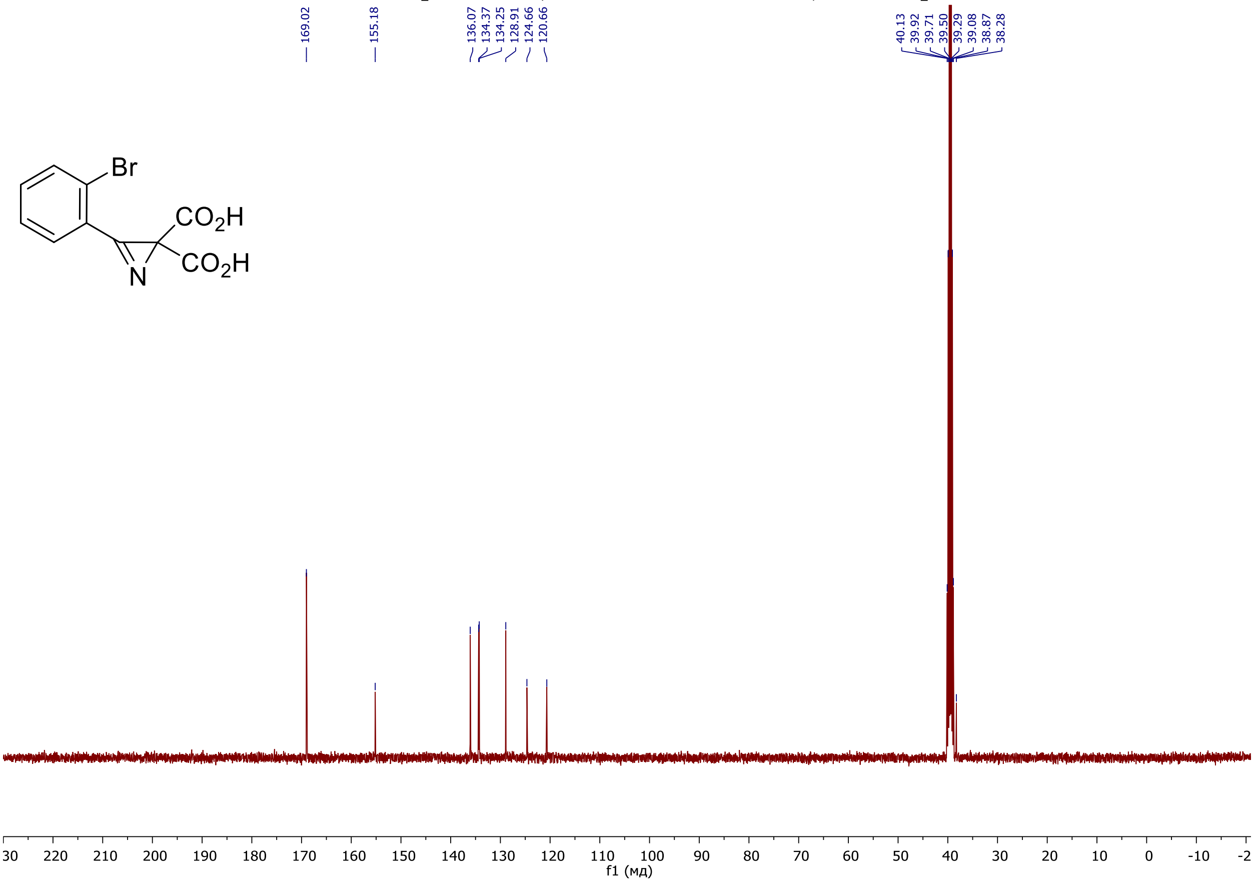

^{1}H NMR spectrum (400 MHz, DMSO-d_6) of compound **6i**

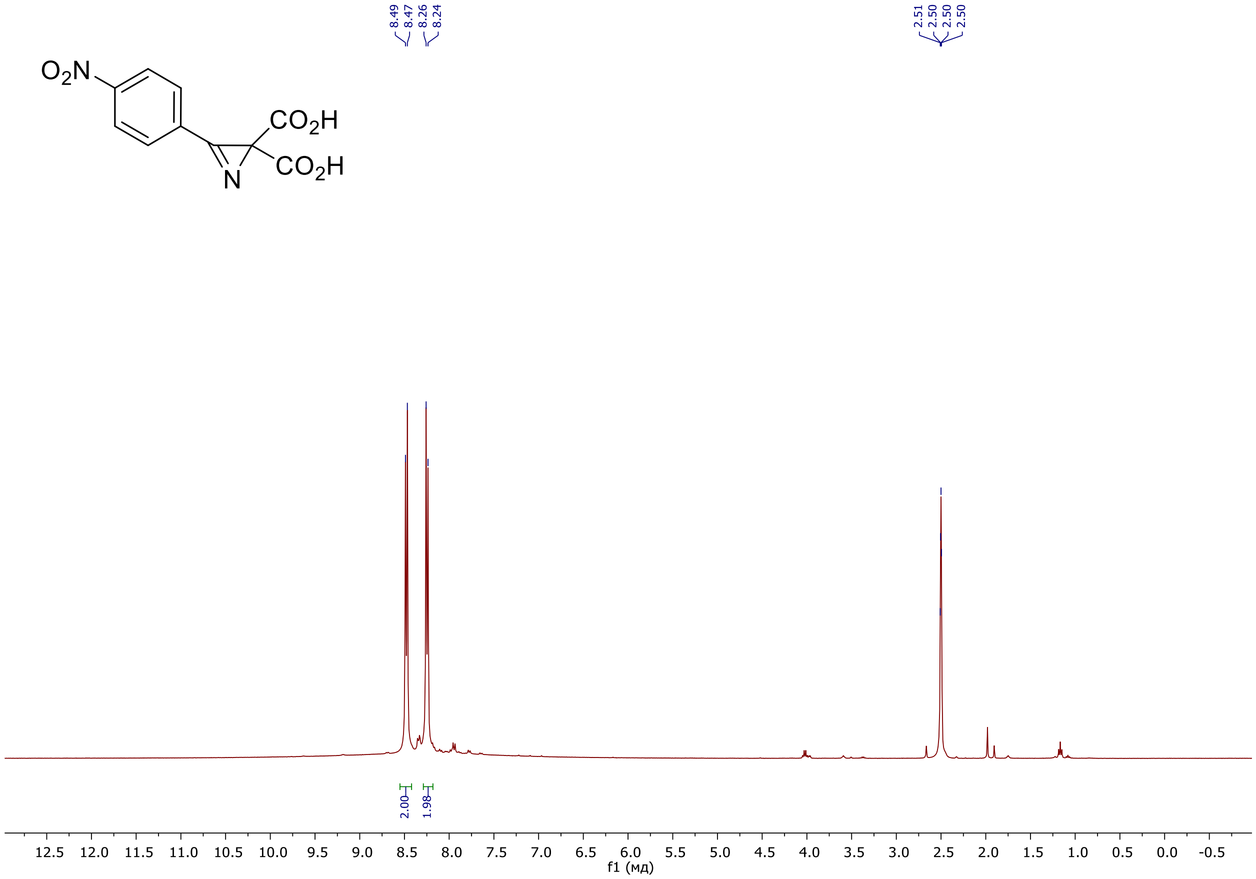

$^{13}C\{^{1}H\}$ NMR spectrum (100 MHz, DMSO-d_6) of compound **6i**

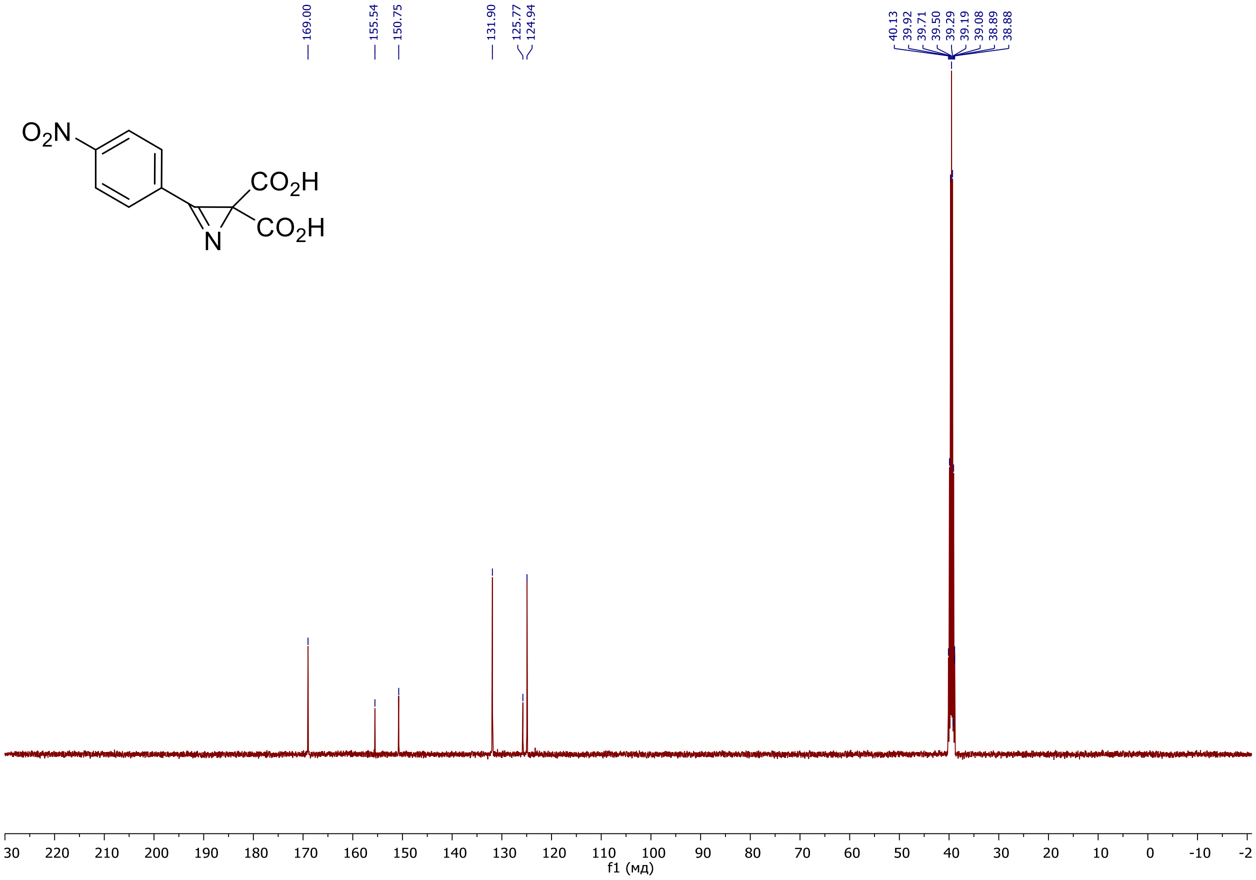

^{1}H NMR spectrum (400 MHz, $CDCl_3$) of compound **9**

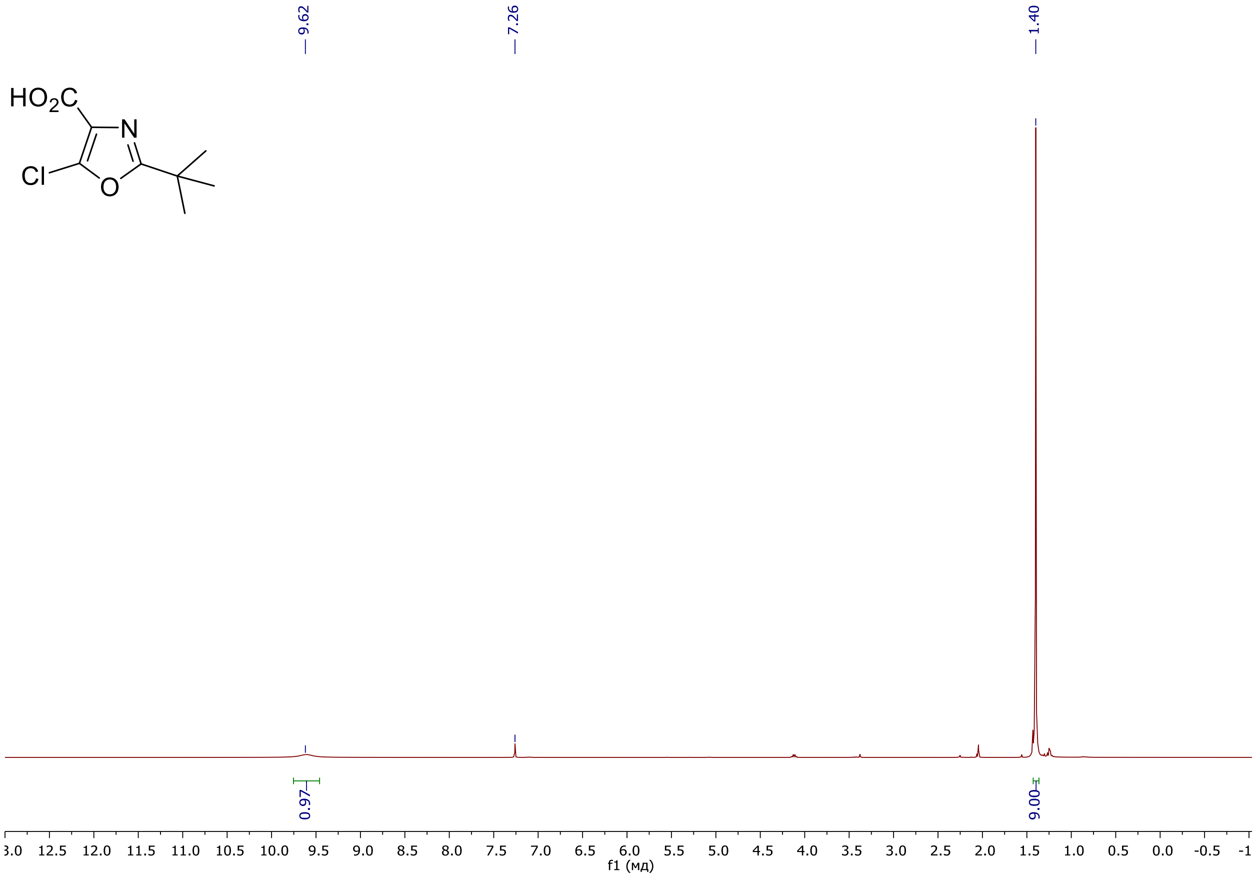

$^{13}C\{^{1}H\}$ NMR spectrum (100 MHz, $CDCl_3$) of compound **9**

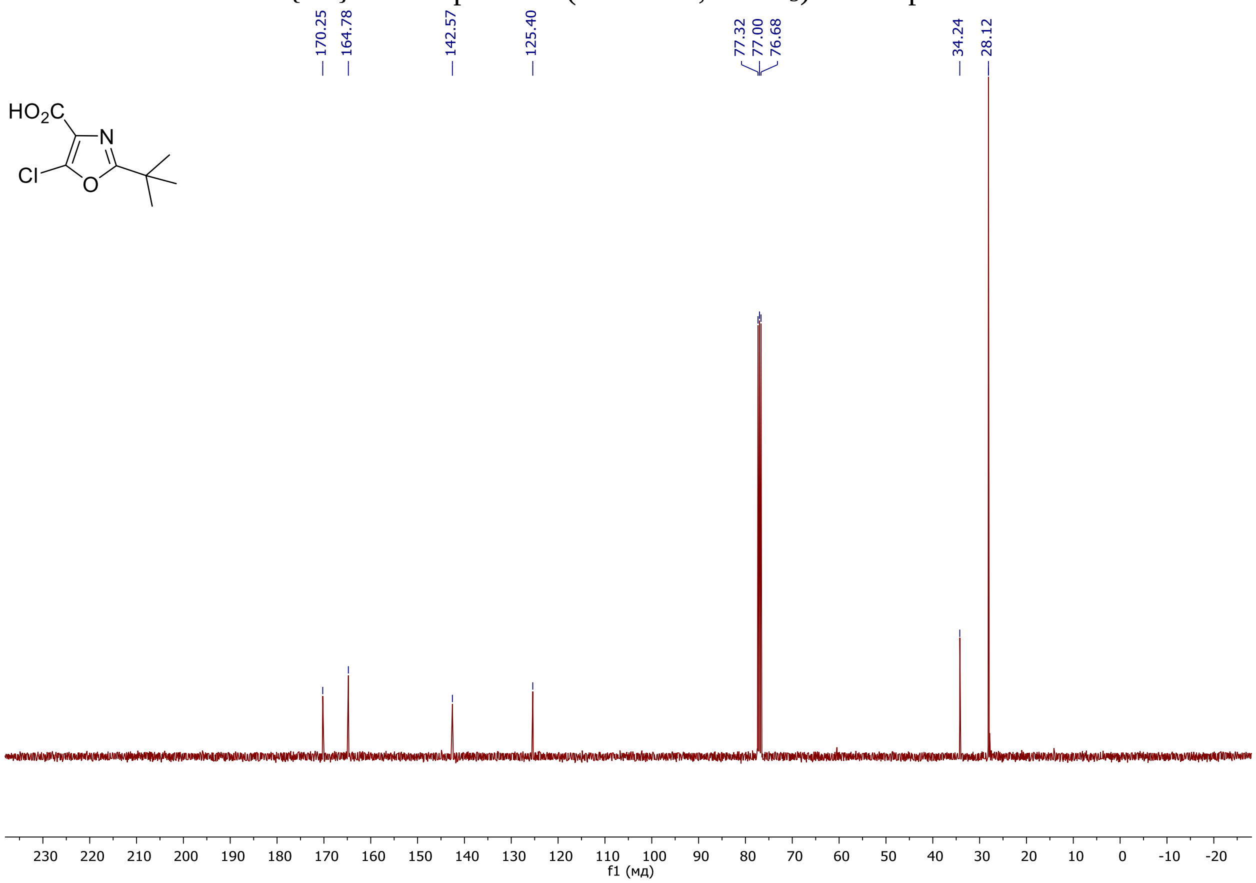

^{1}H NMR spectrum (400 MHz, $CDCl_3$) of compound **10a**

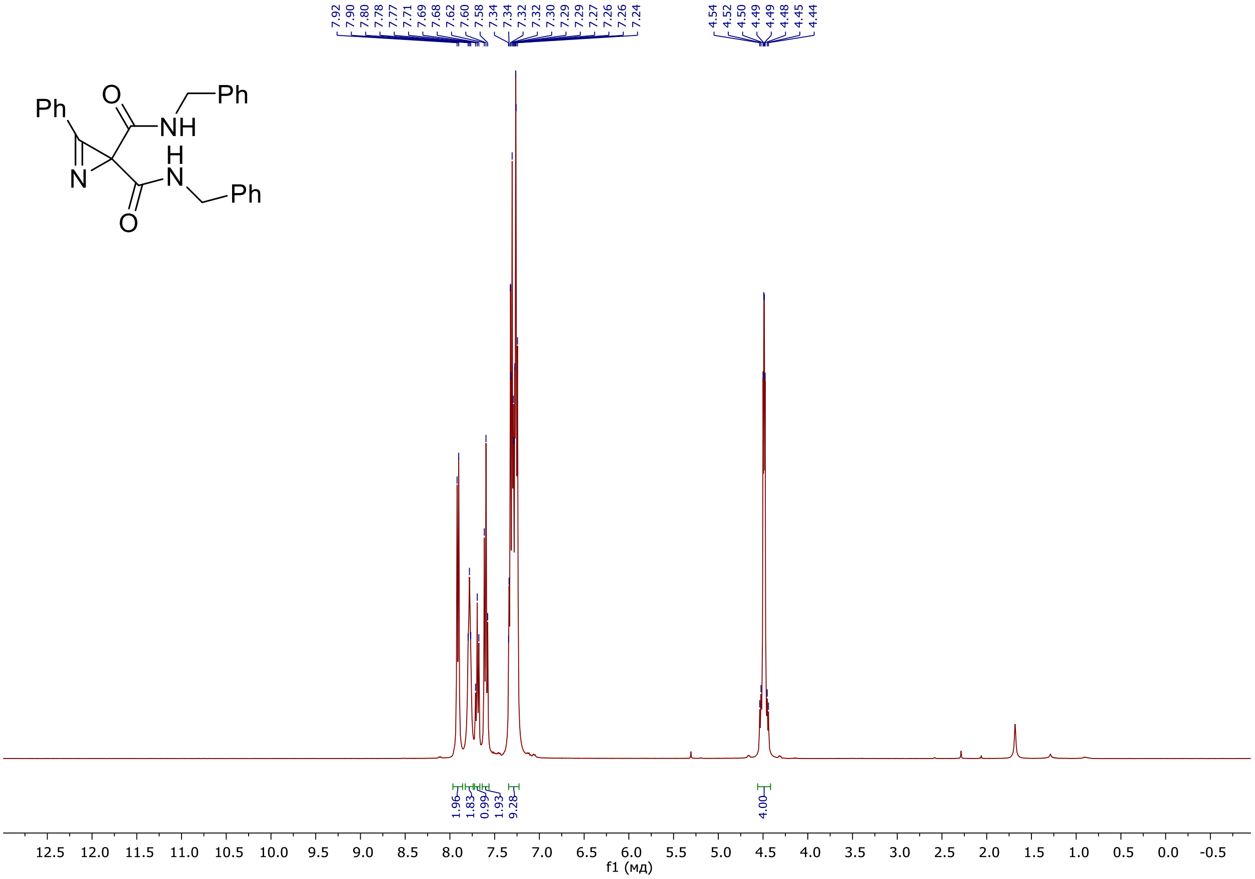

^{13}C{^{1}H} NMR spectrum (100 MHz, $CDCl_3$) of compound **10a**

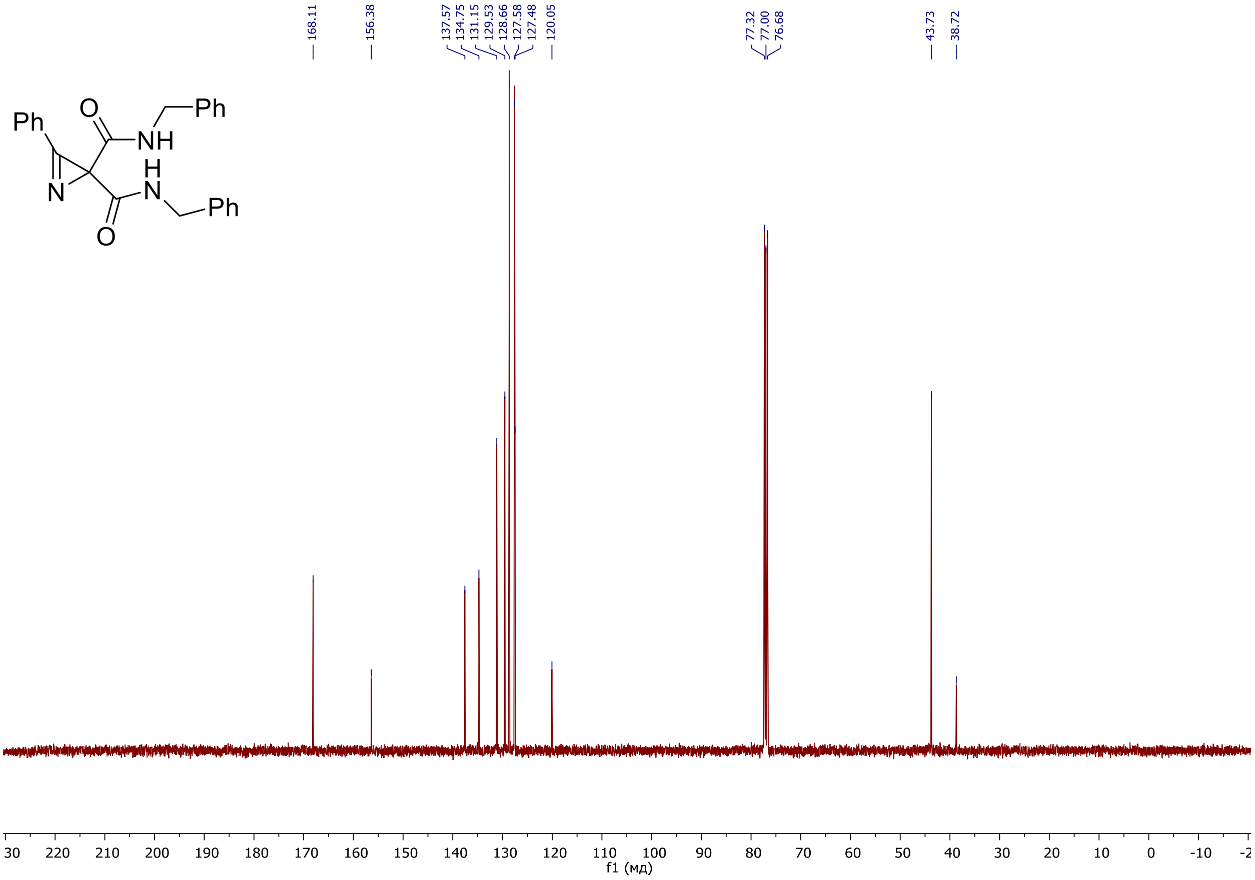

^{1}H NMR spectrum (400 MHz, $CDCl_3$) of compound **10b**

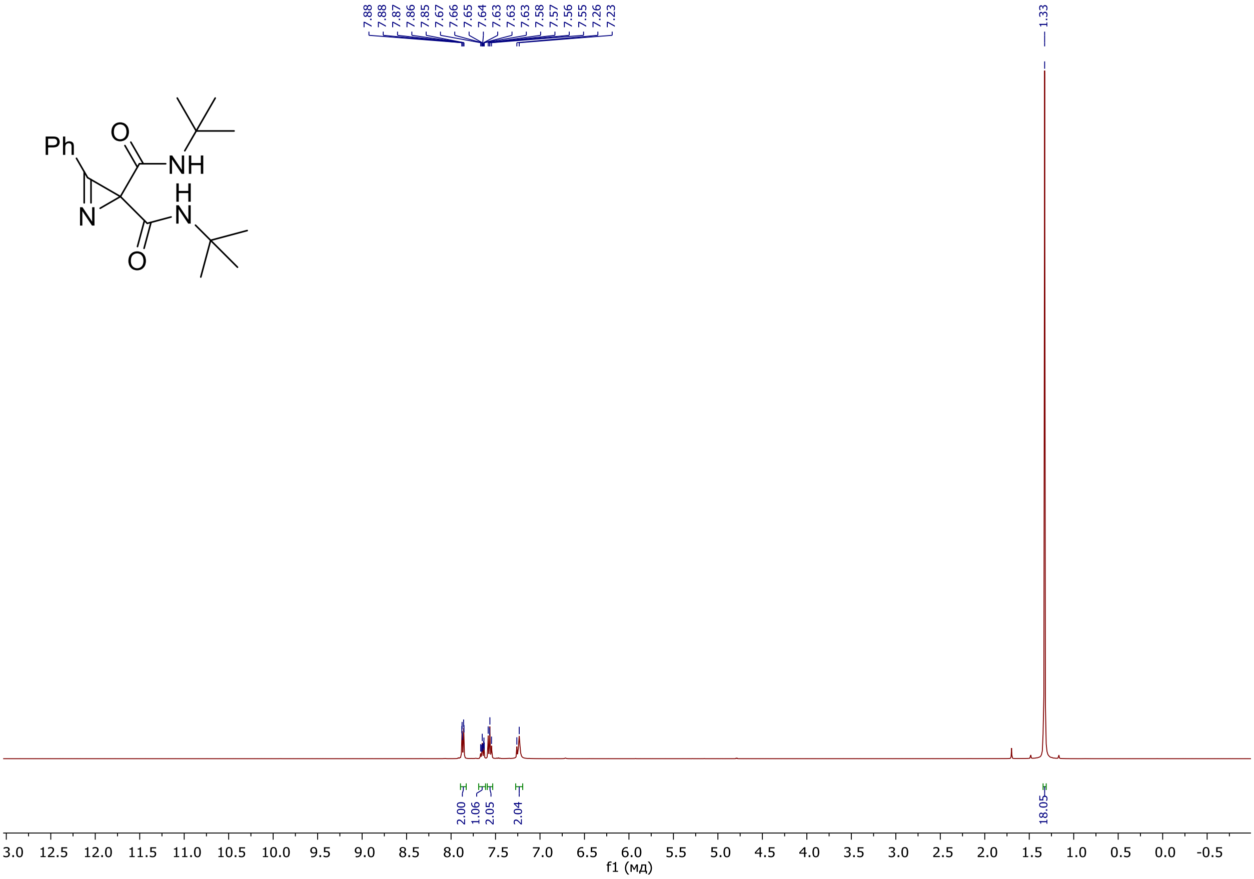

^{13}C{^{1}H} NMR spectrum (100 MHz, $CDCl_3$) of compound **10b**

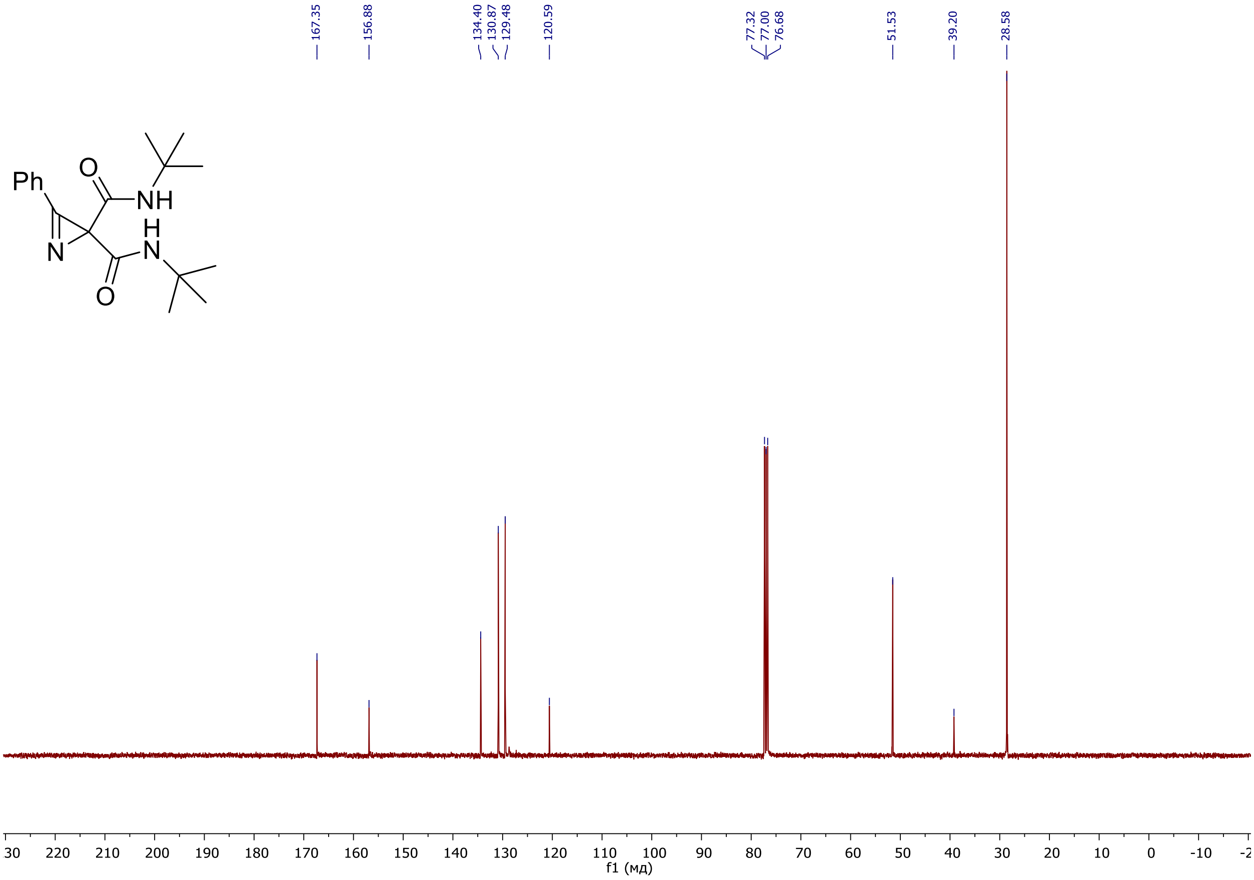

^{1}H NMR spectrum (400 MHz, DMSO-d_6) of compound **10c**

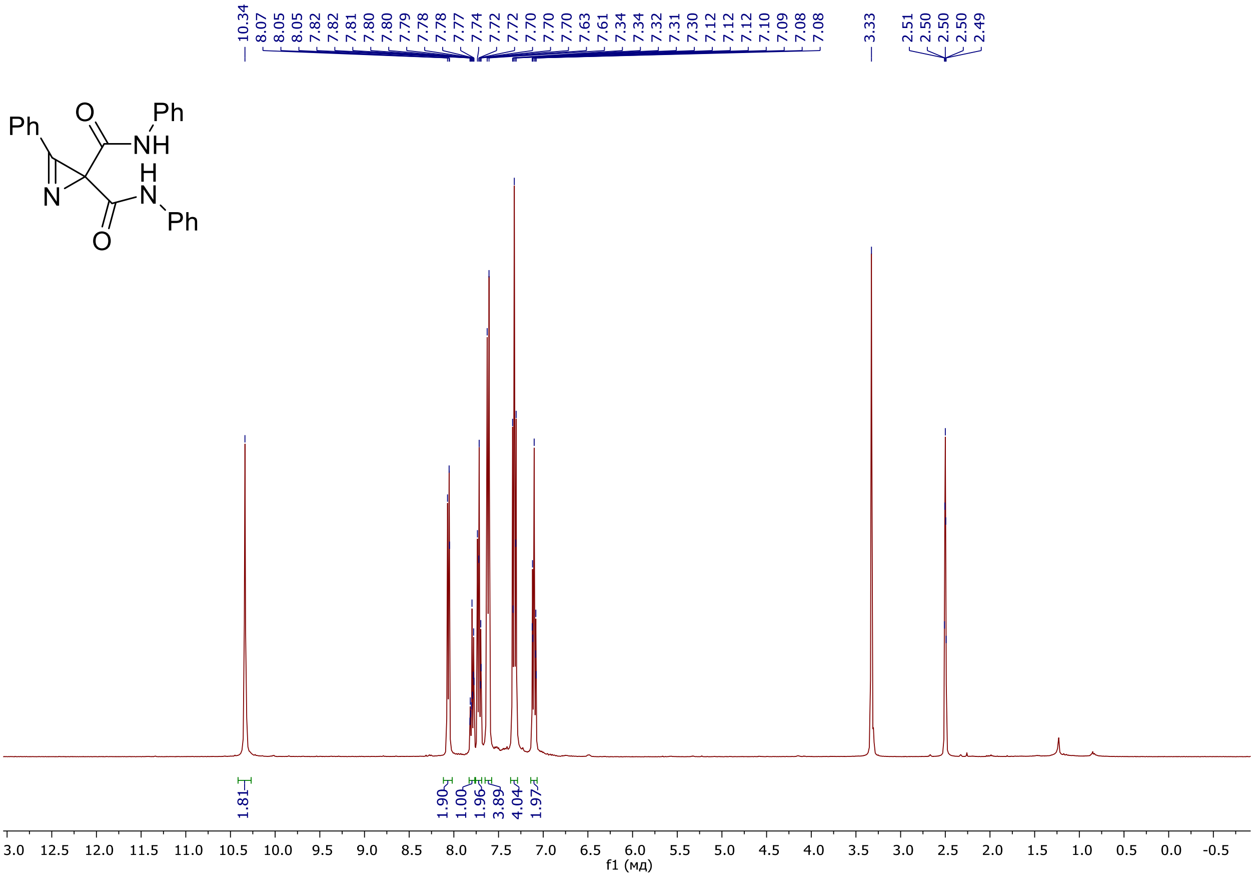

^{13}C{^{1}H} NMR spectrum (100 MHz, DMSO-d_6) of compound **10c**

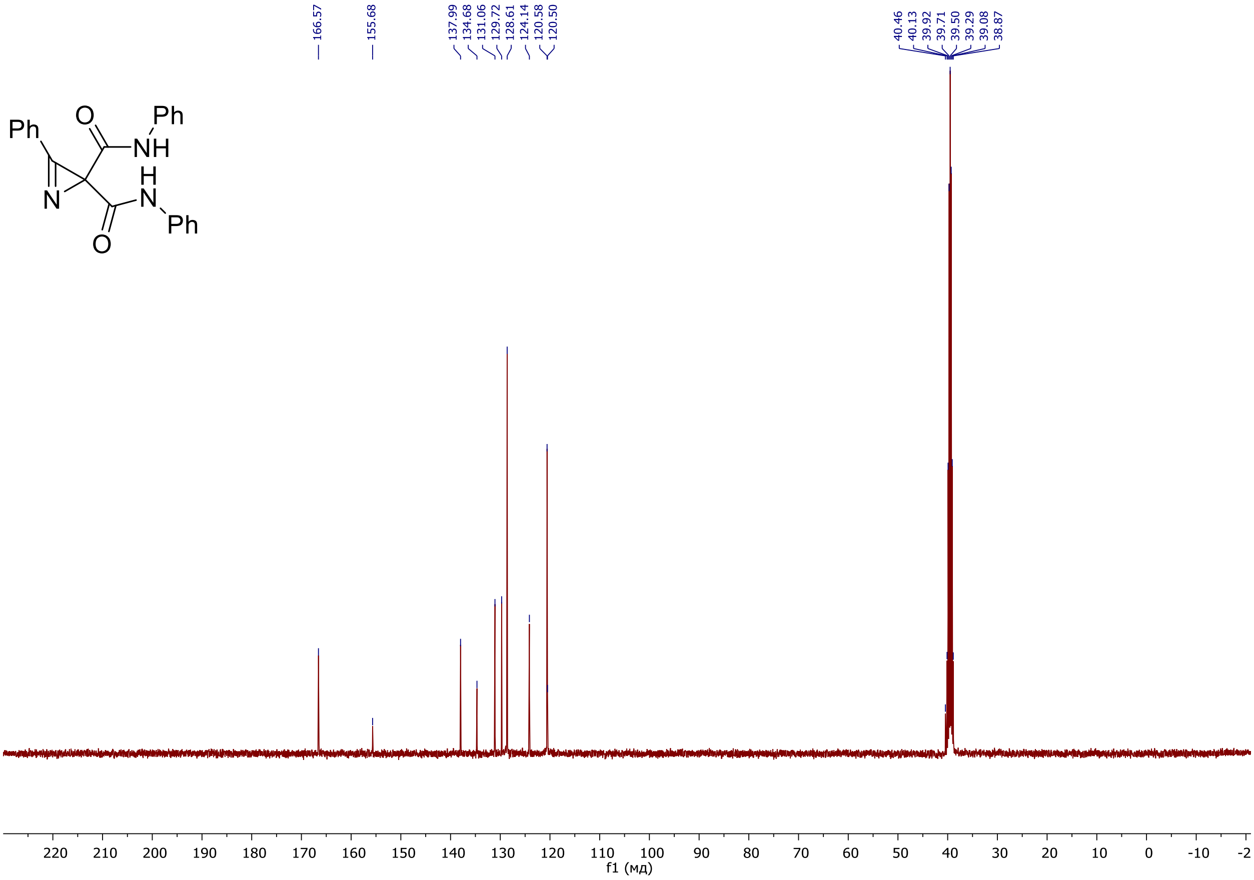

^{1}H NMR spectrum (400 MHz, $CDCl_3$) of compound **10d**

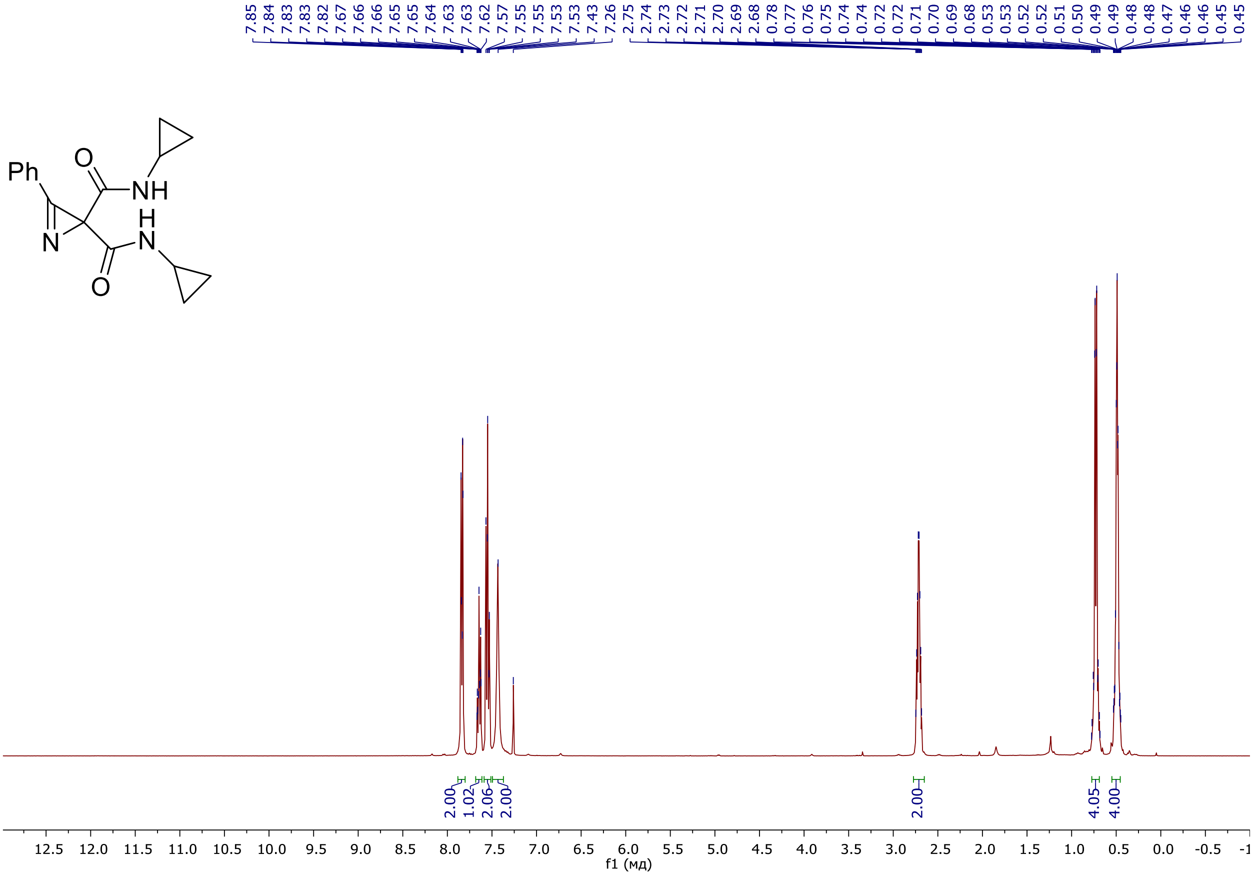

^{13}C{^{1}H} NMR spectrum (100 MHz, $CDCl_3$) of compound **10d**

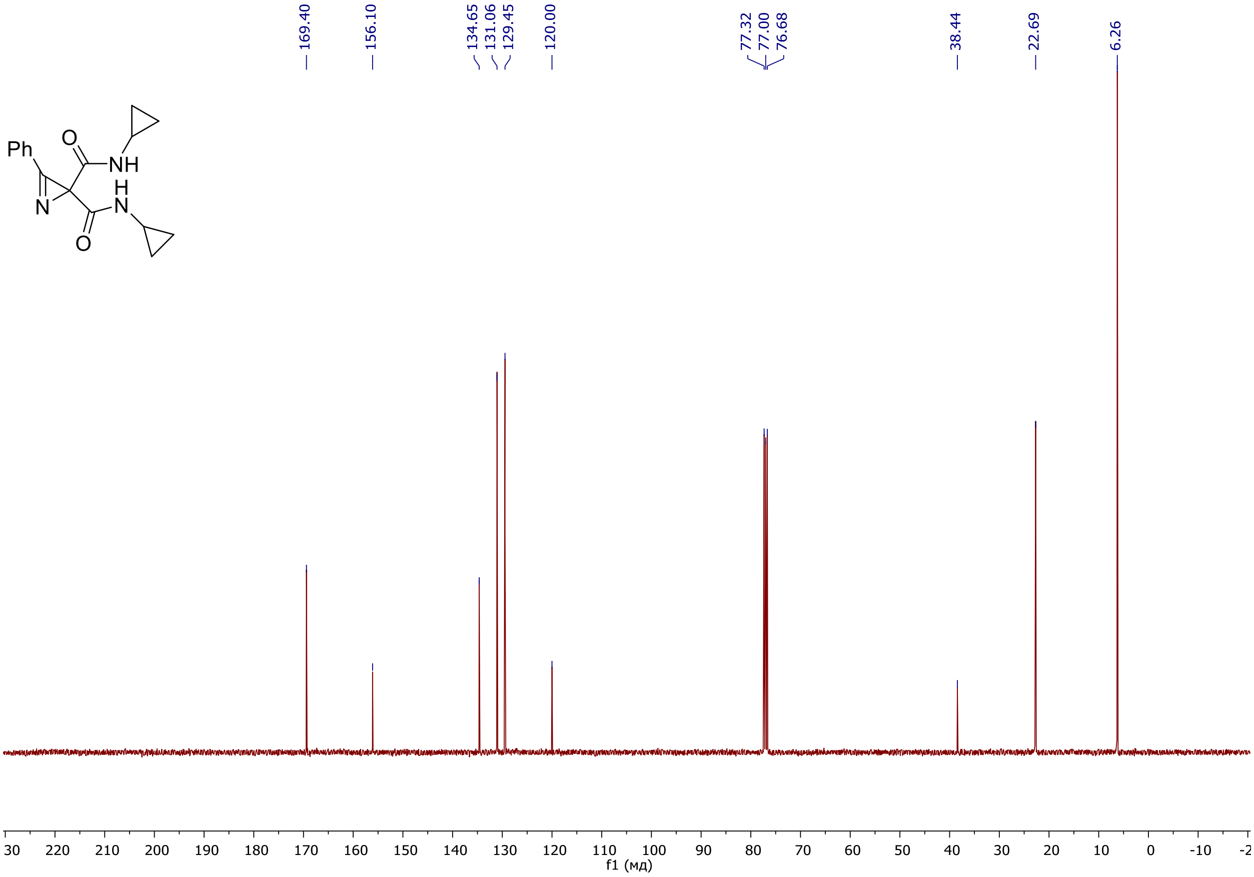

^{1}H NMR spectrum (400 MHz, $CDCl_3$) of compound **10e**

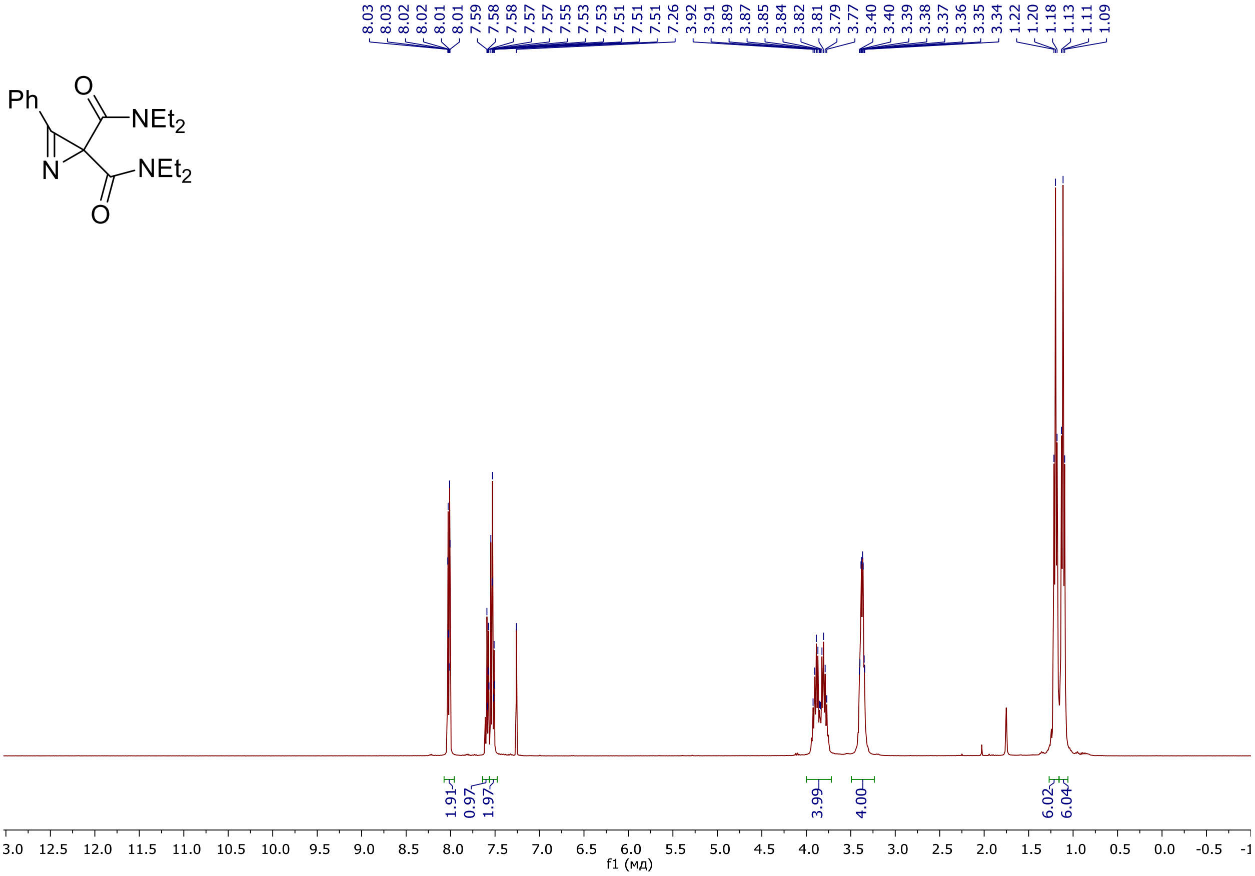

$^{13}C\{^{1}H\}$ NMR spectrum (100 MHz, $CDCl_3$) of compound **10e**

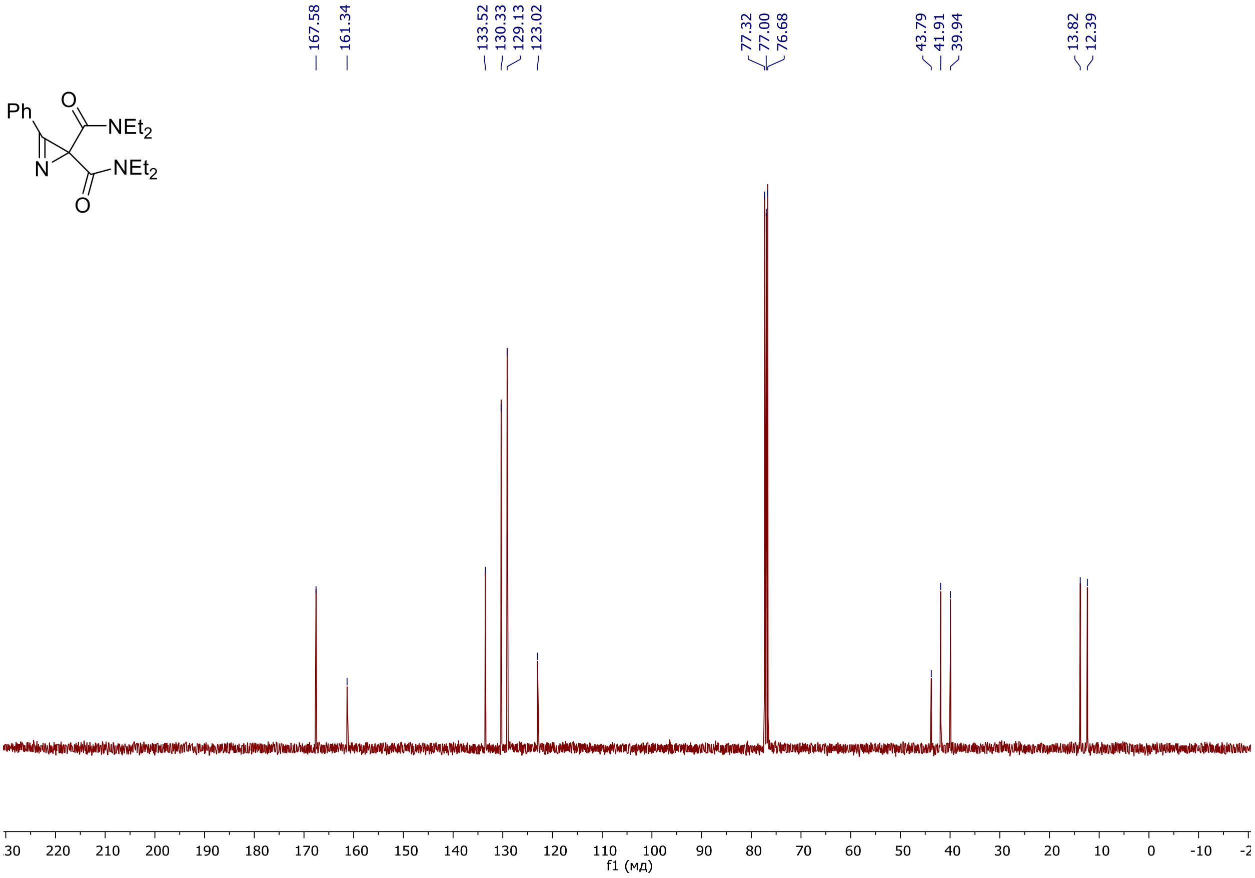

^{1}H NMR spectrum (400 MHz, $CDCl_3$) of compound **10f**

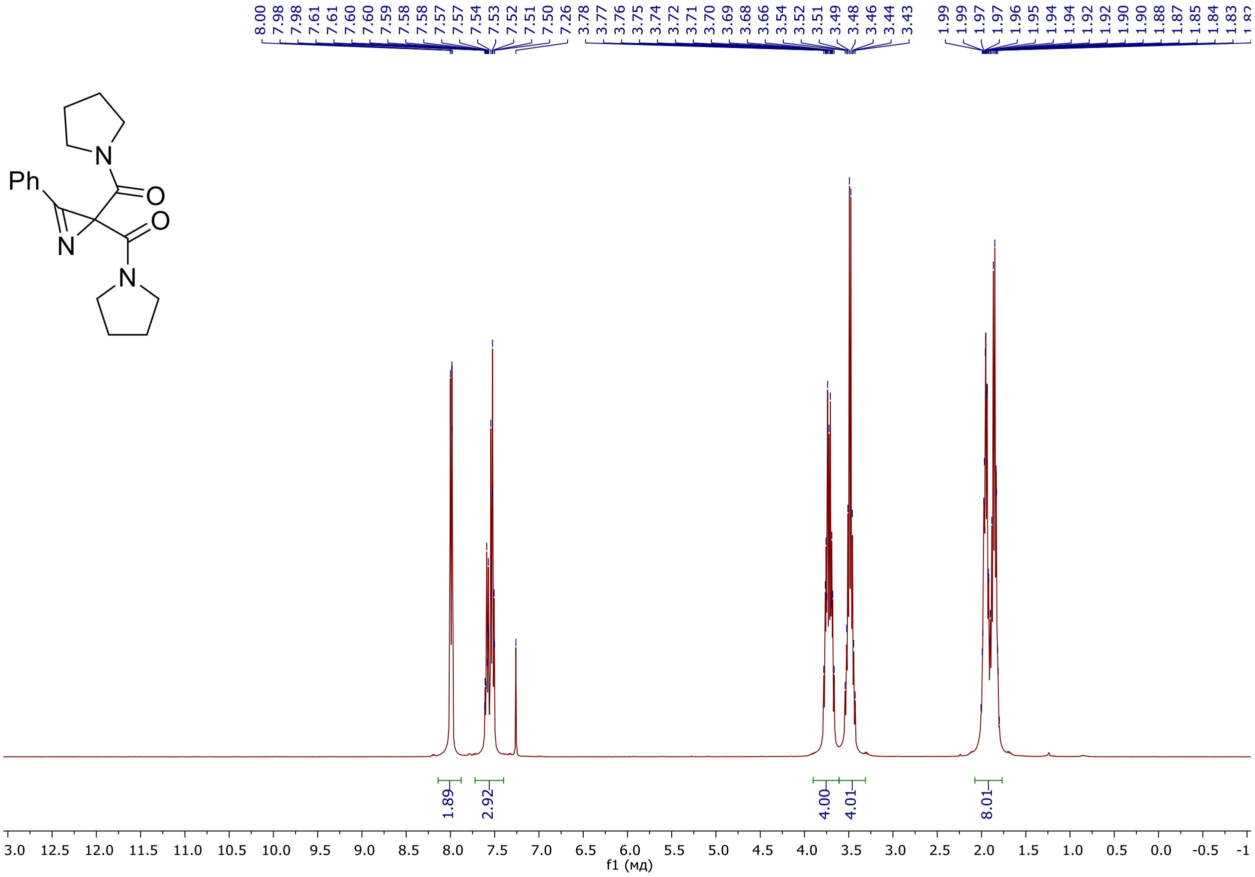

^{13}C{^{1}H} NMR spectrum (100 MHz, $CDCl_3$) of compound **10f**

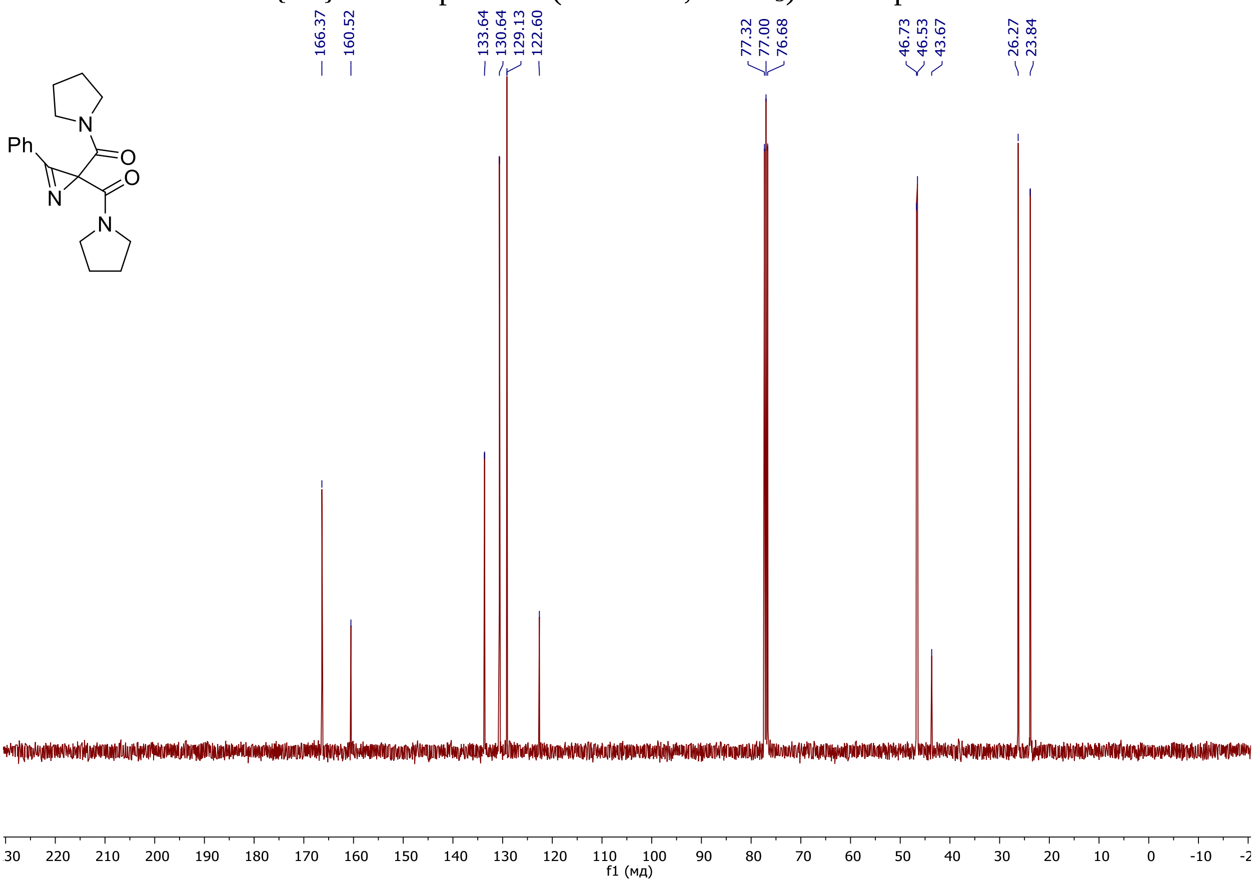

^{1}H NMR spectrum (400 MHz, $CDCl_3$) of compound **10g**

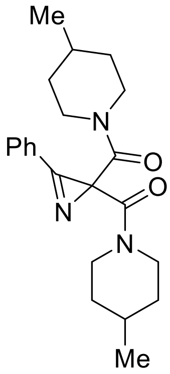

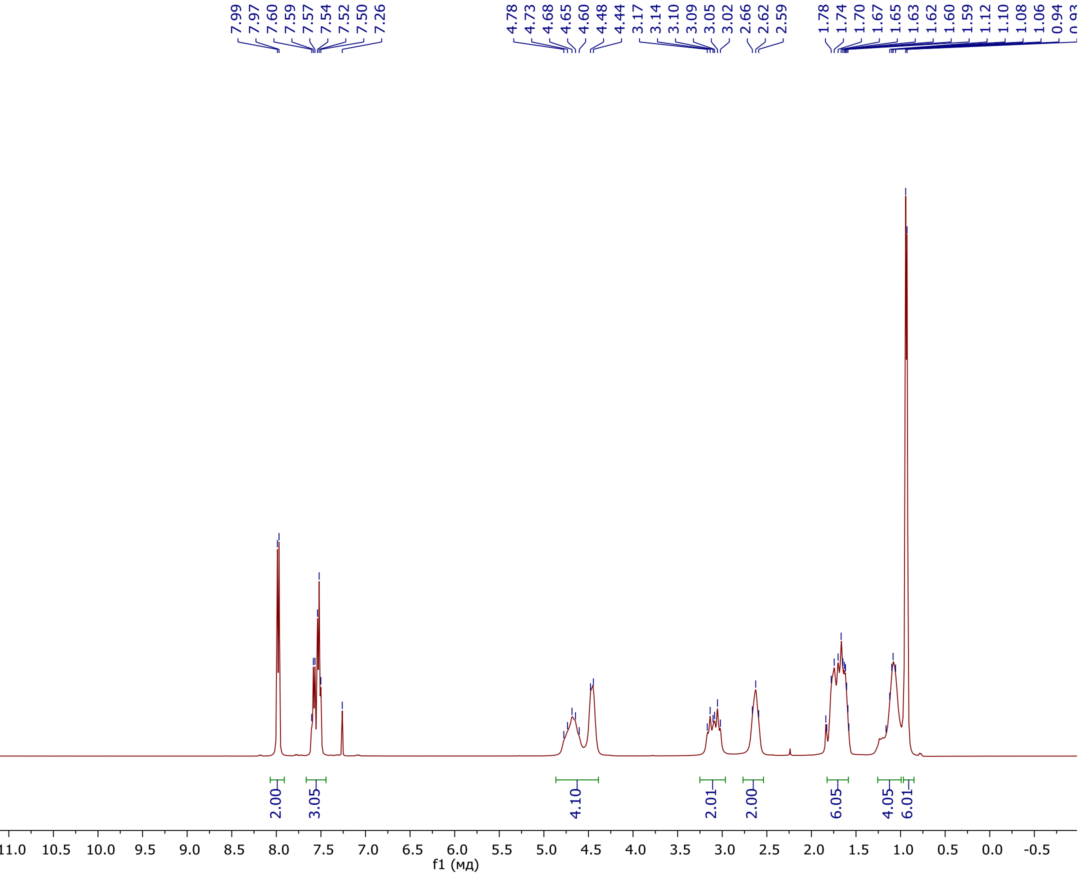

$^{13}C\{^{1}H\}$ NMR spectrum (100 MHz, $CDCl_3$) of compound **10g**

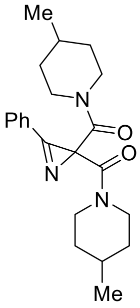

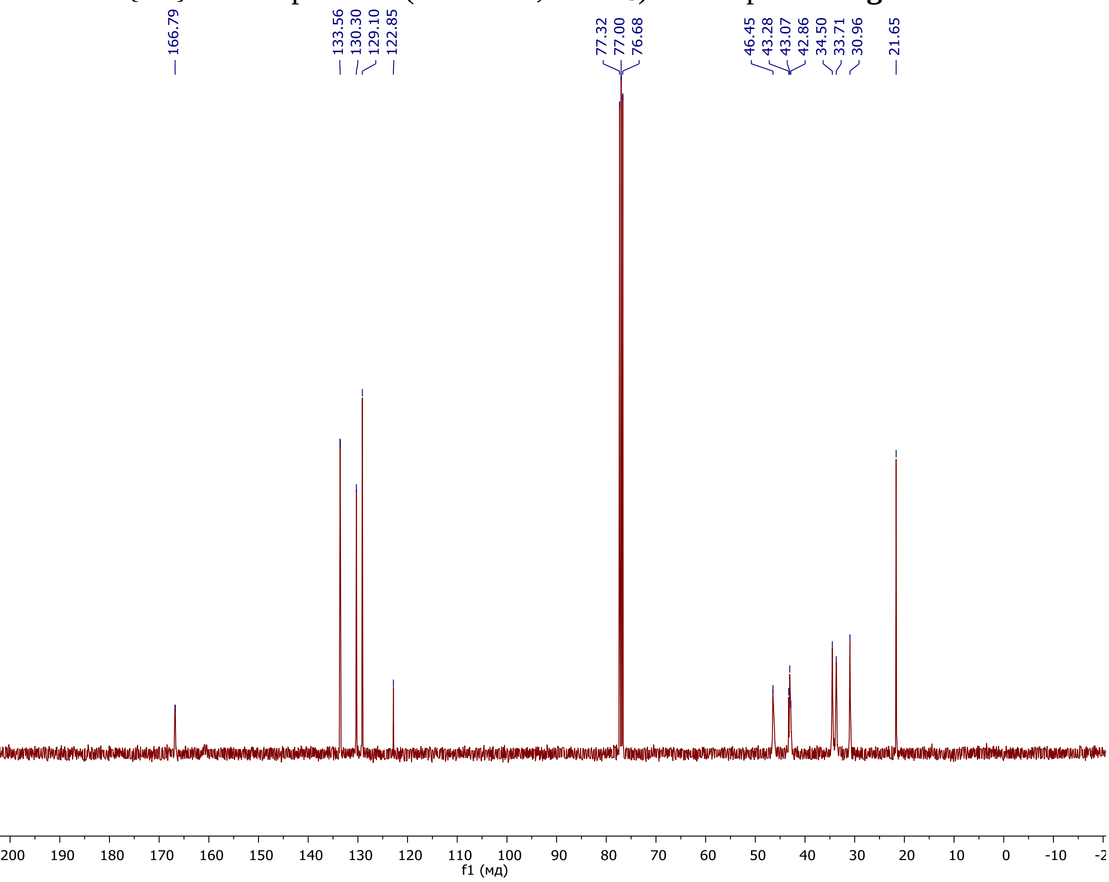

^{1}H NMR spectrum (400 MHz, $CDCl_3$) of compound **10h**

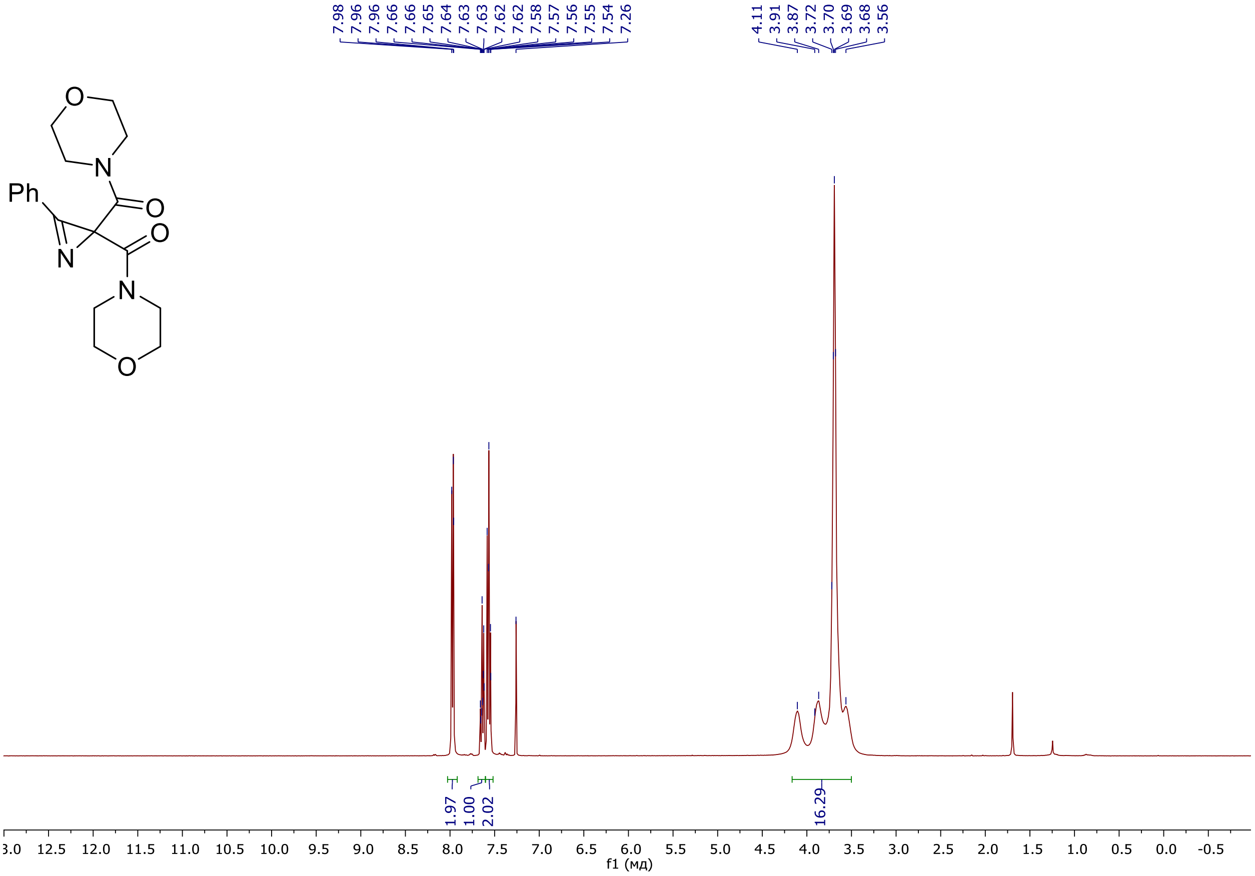

$^{13}C\{^{1}H\}$ NMR spectrum (100 MHz, $CDCl_3$) of compound **10h**

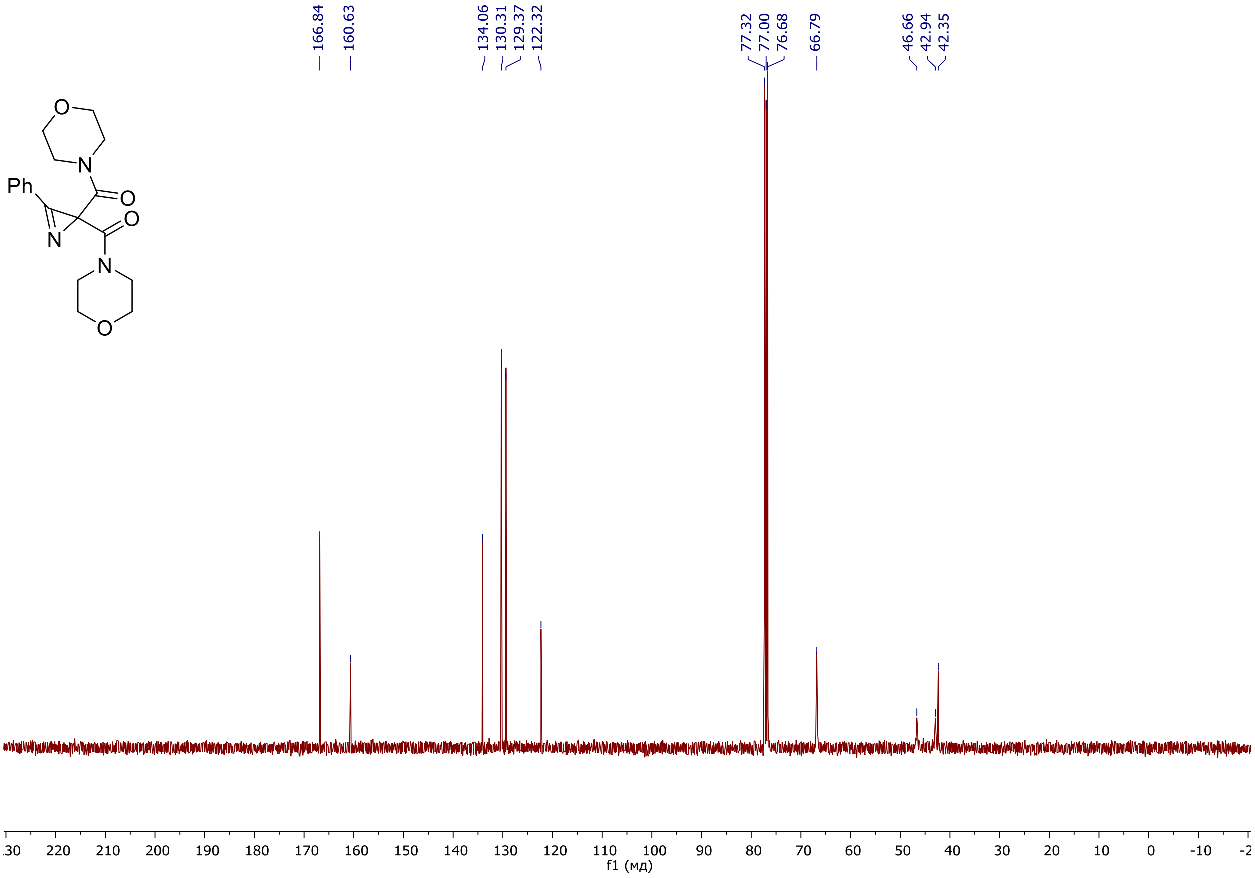

^{1}H NMR spectrum (400 MHz, $CDCl_3$) of compound **11a**

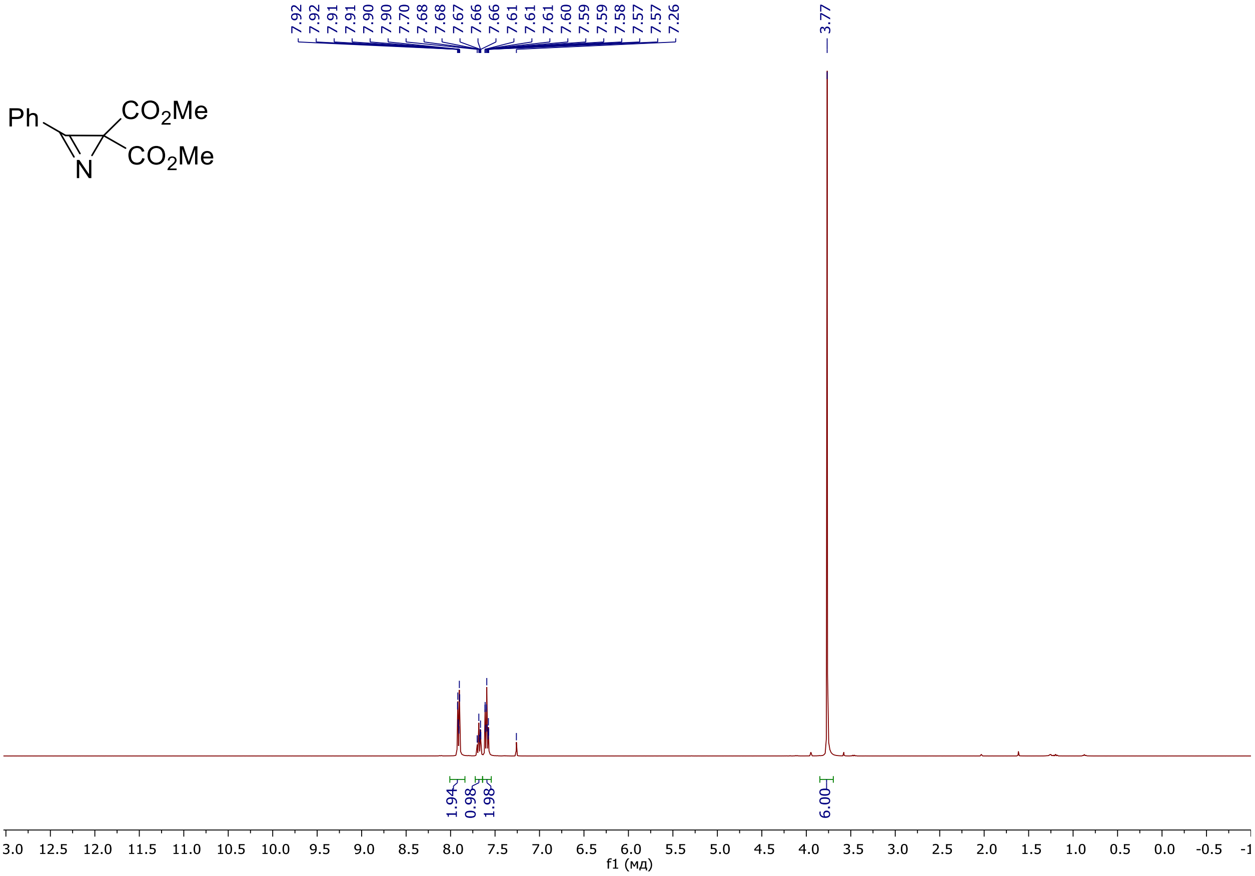

$^{13}C\{^{1}H\}$ NMR spectrum (100 MHz, $CDCl_3$) of compound **11a**

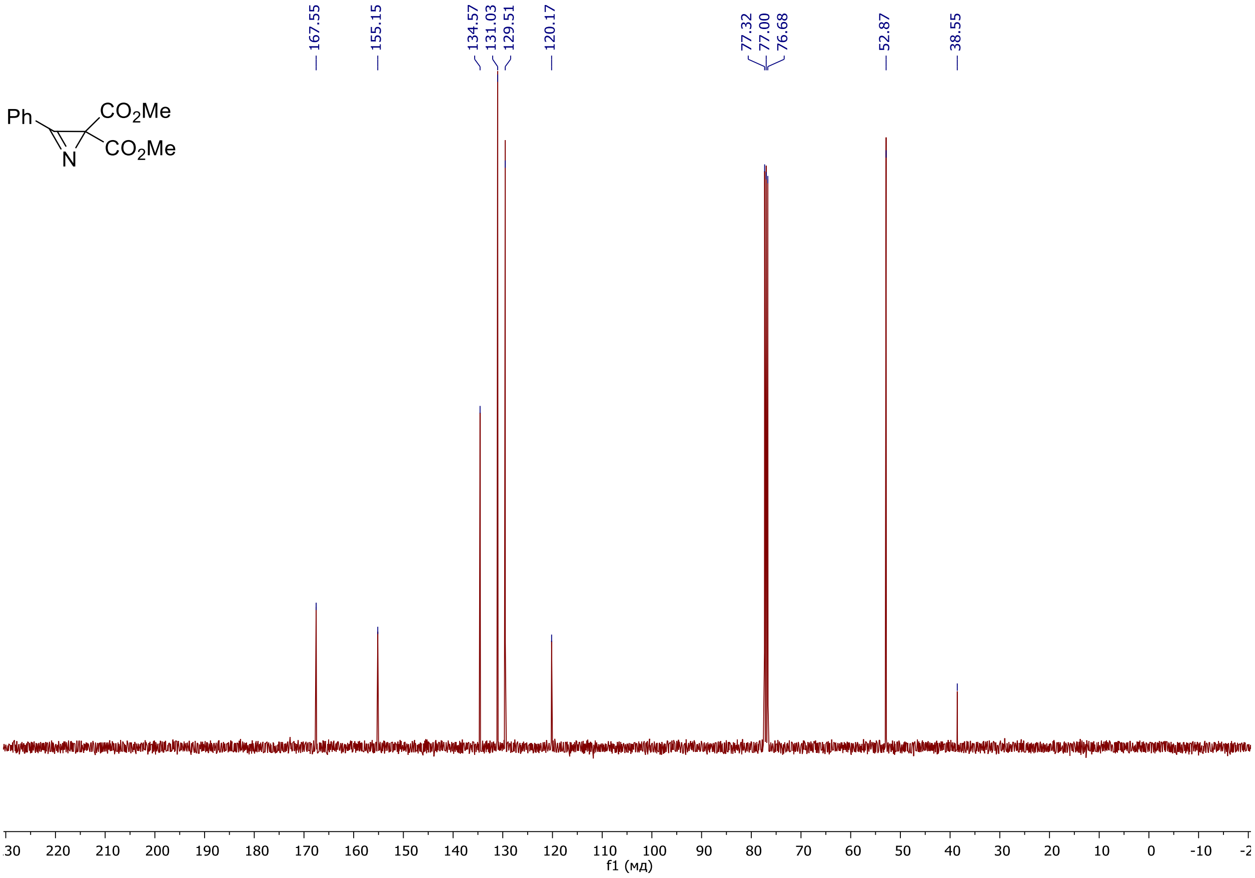

^{1}H NMR spectrum (400 MHz, $CDCl_3$) of compound **11b**

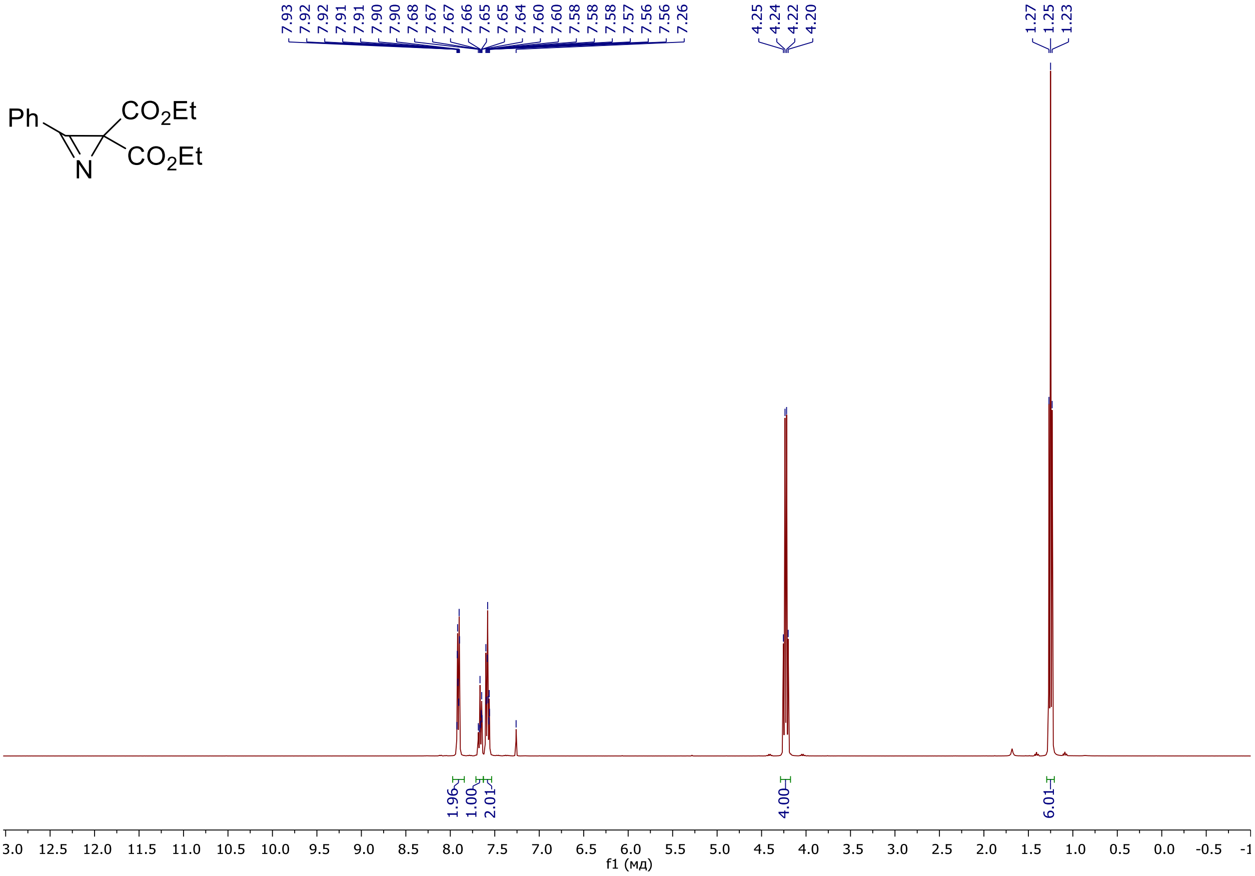

^{13}C{^{1}H} NMR spectrum (100 MHz, $CDCl_3$) of compound **11b**

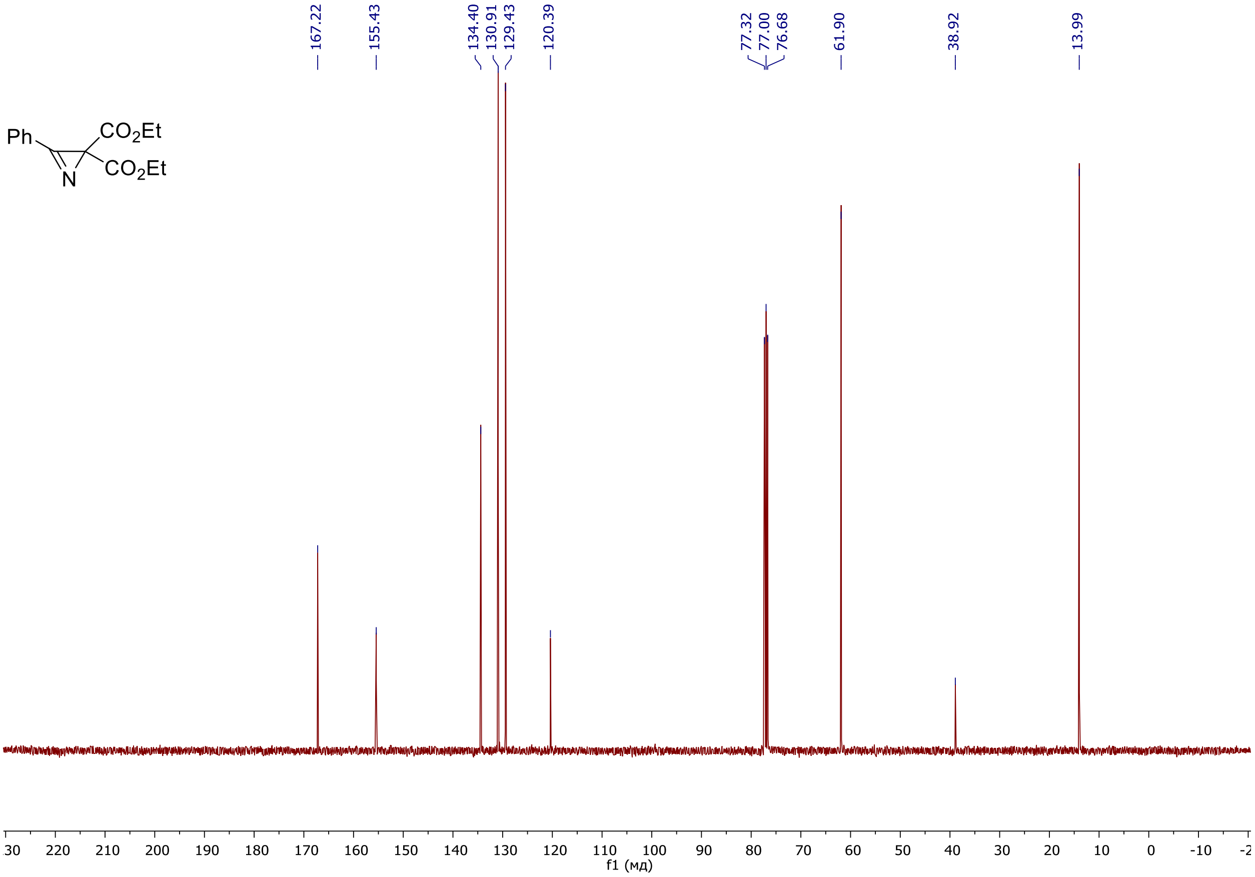

^{1}H NMR spectrum (400 MHz, $CDCl_3$) of compound **11c**

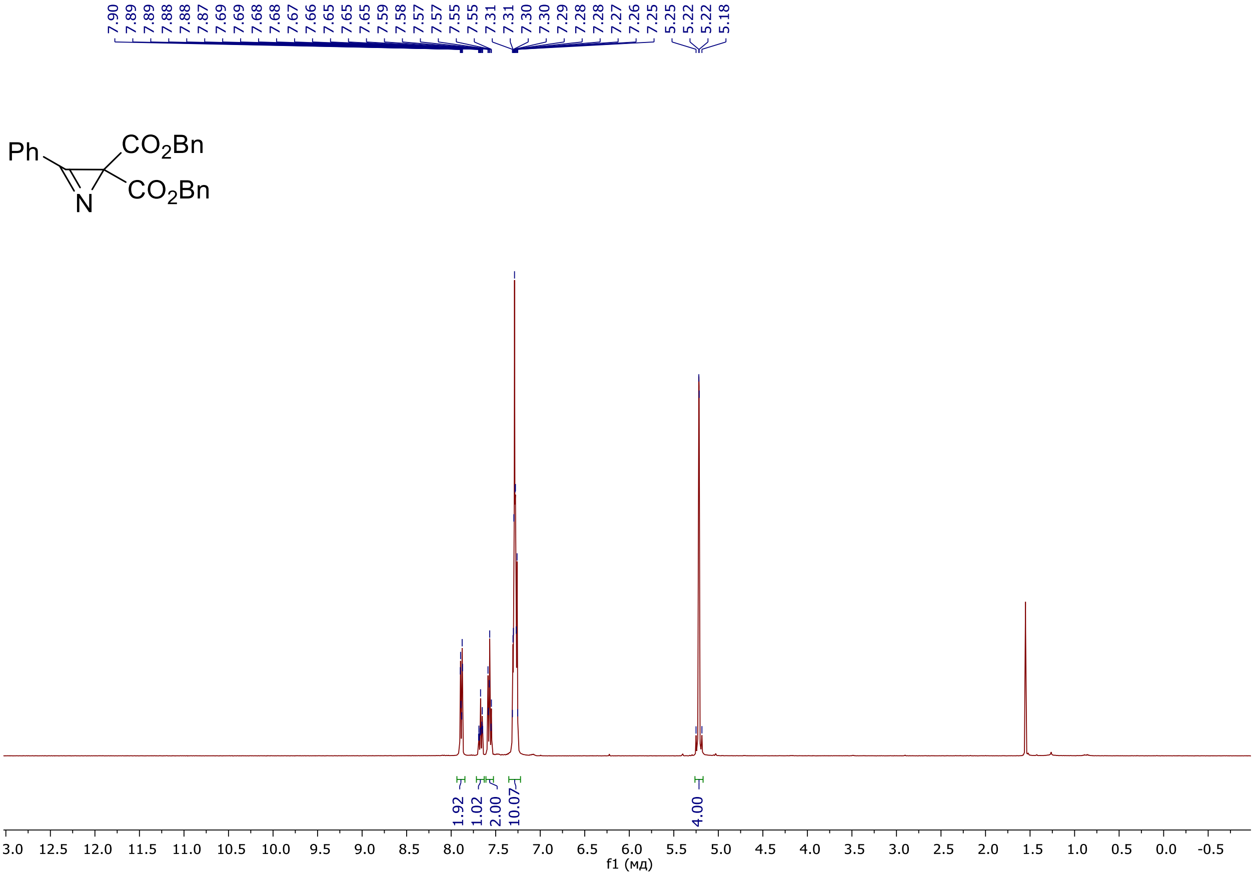

$^{13}C\{^1H\}$ NMR spectrum (100 MHz, $CDCl_3$) of compound **11c**

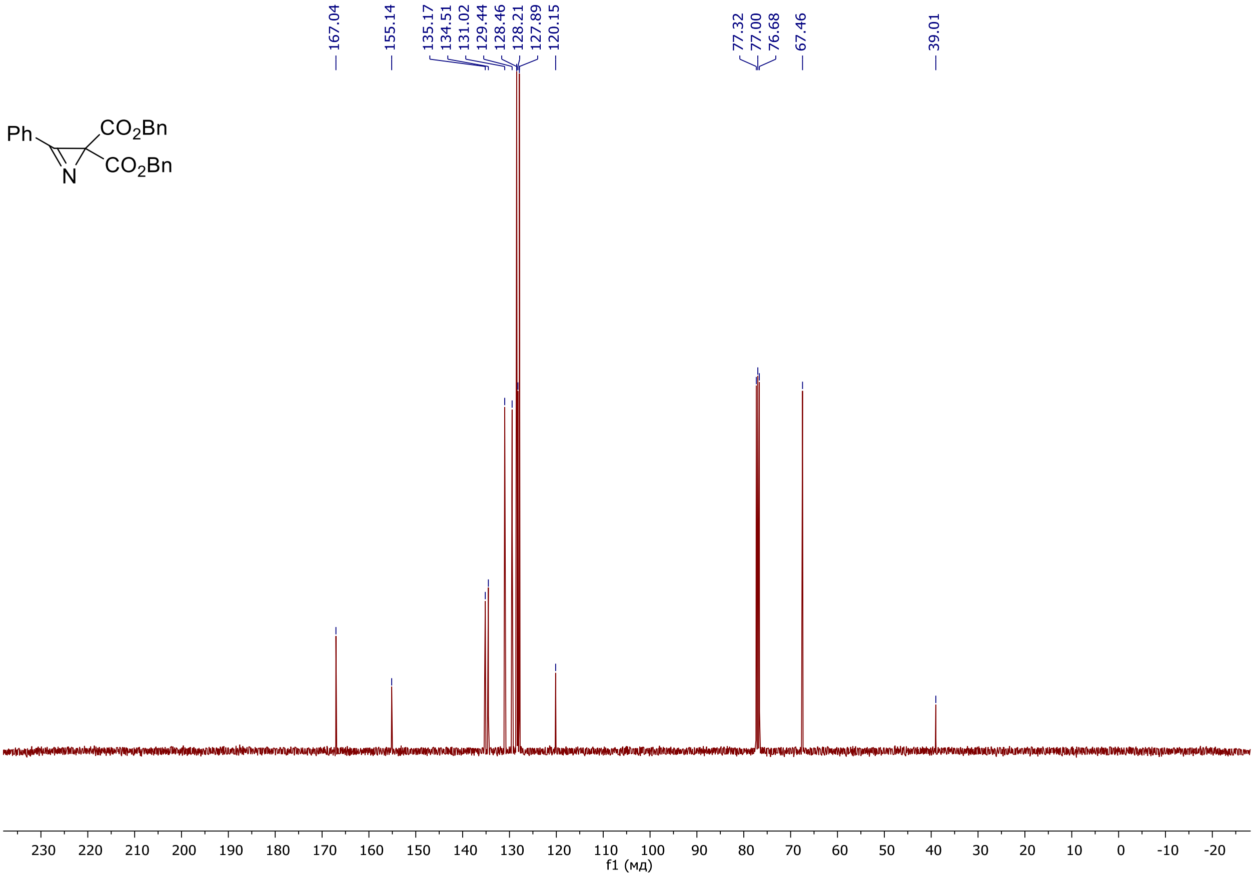

^{1}H NMR spectrum (400 MHz, $CDCl_3$) of compound **11d**

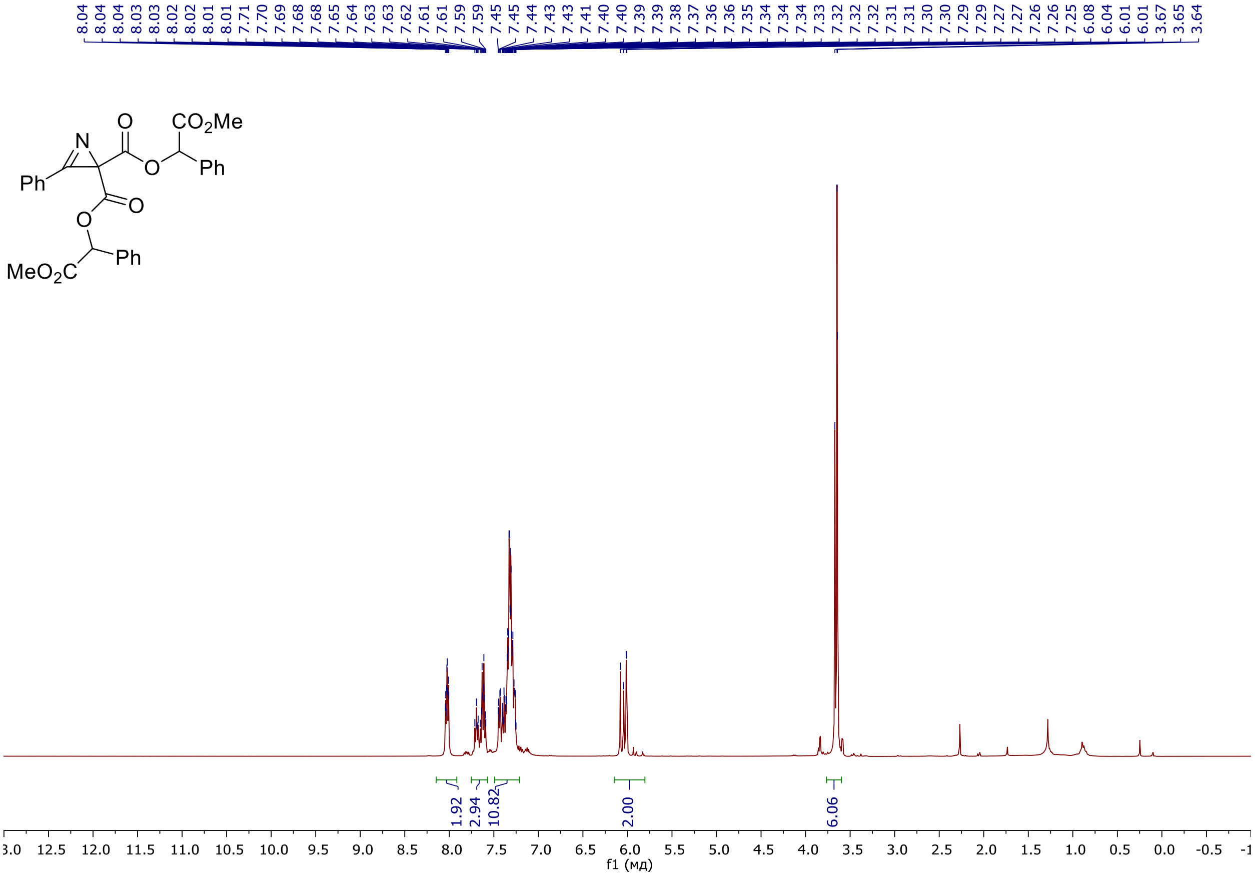

^{13}C{^{1}H} NMR spectrum (100 MHz, $CDCl_3$) of compound **11d**

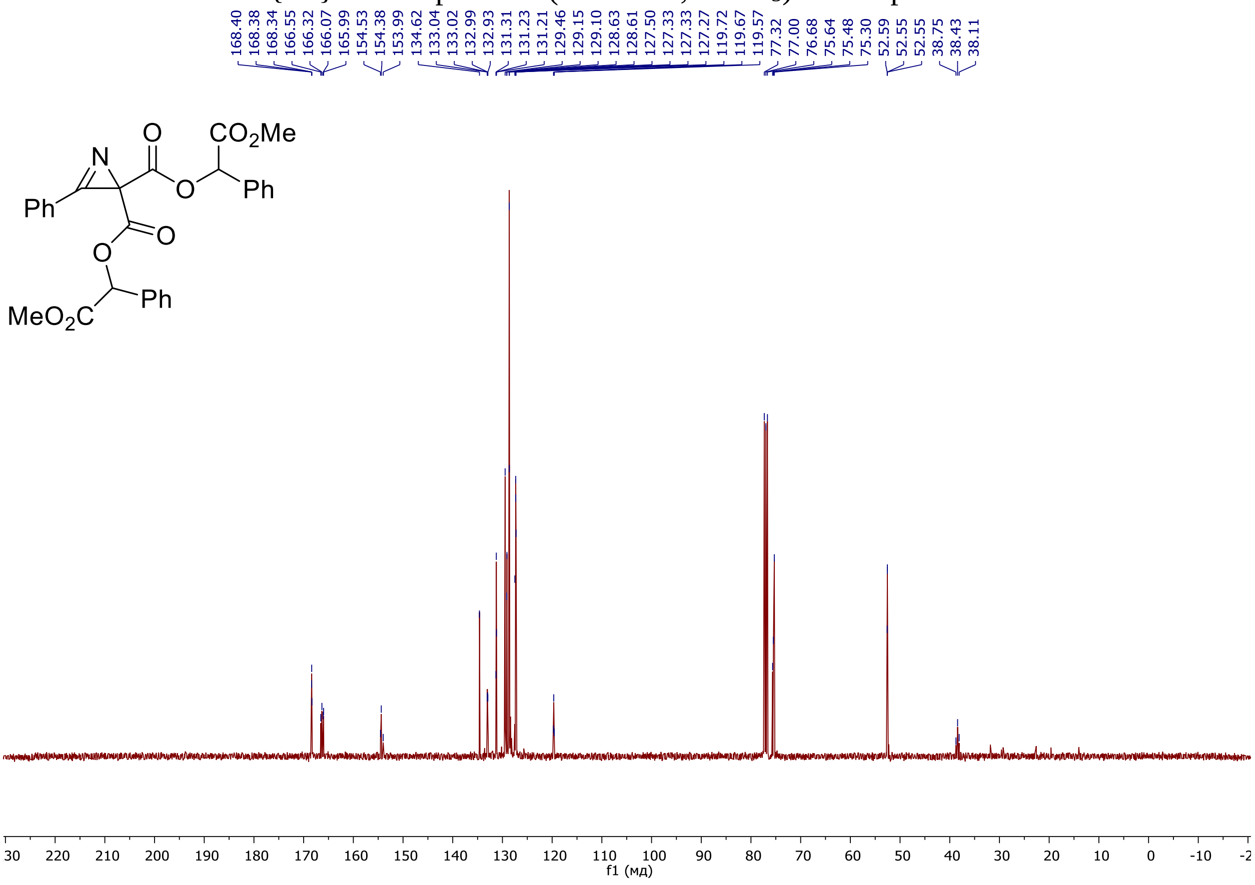

^{1}H NMR spectrum (400 MHz, $CDCl_3$) of compound **12**

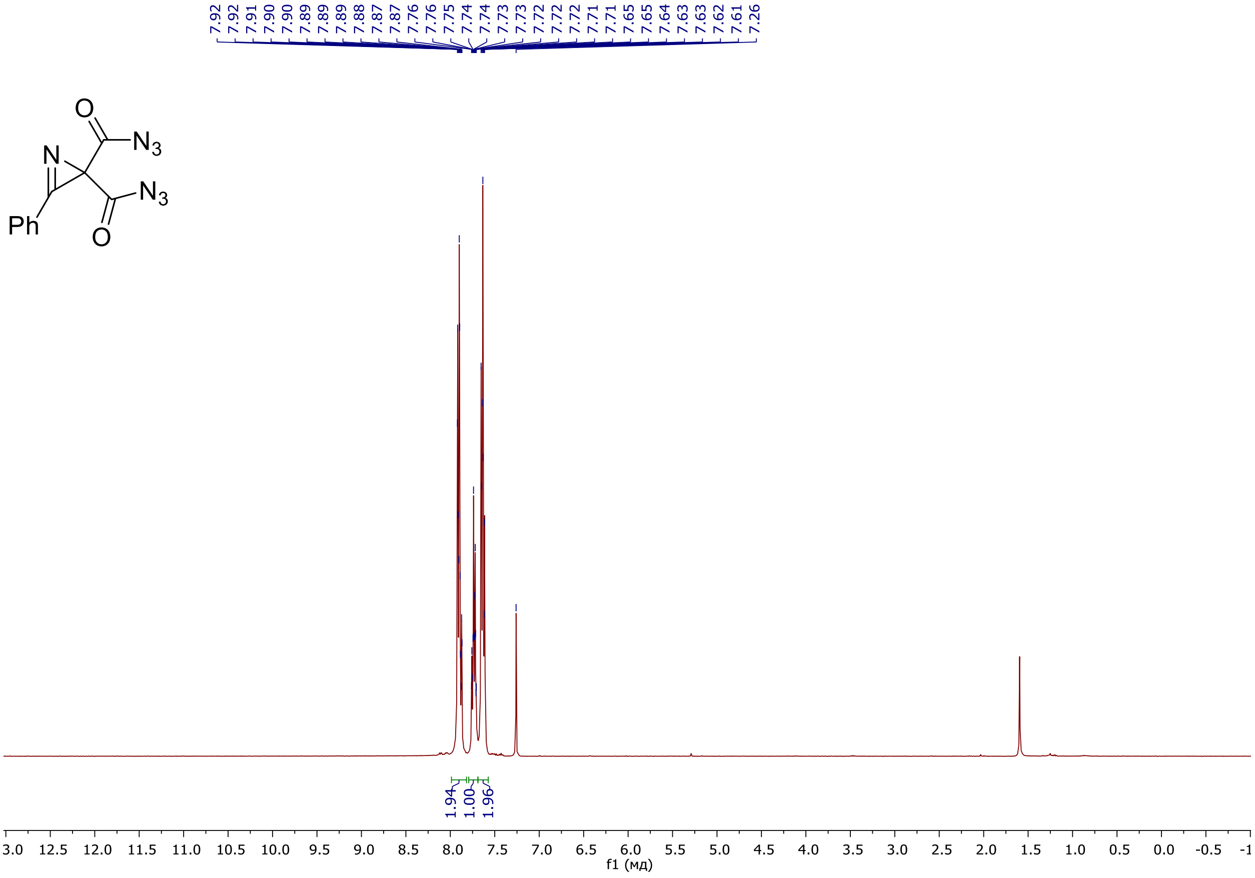

$^{13}C\{^{1}H\}$ NMR spectrum (100 MHz, C_6D_6) of compound **12**

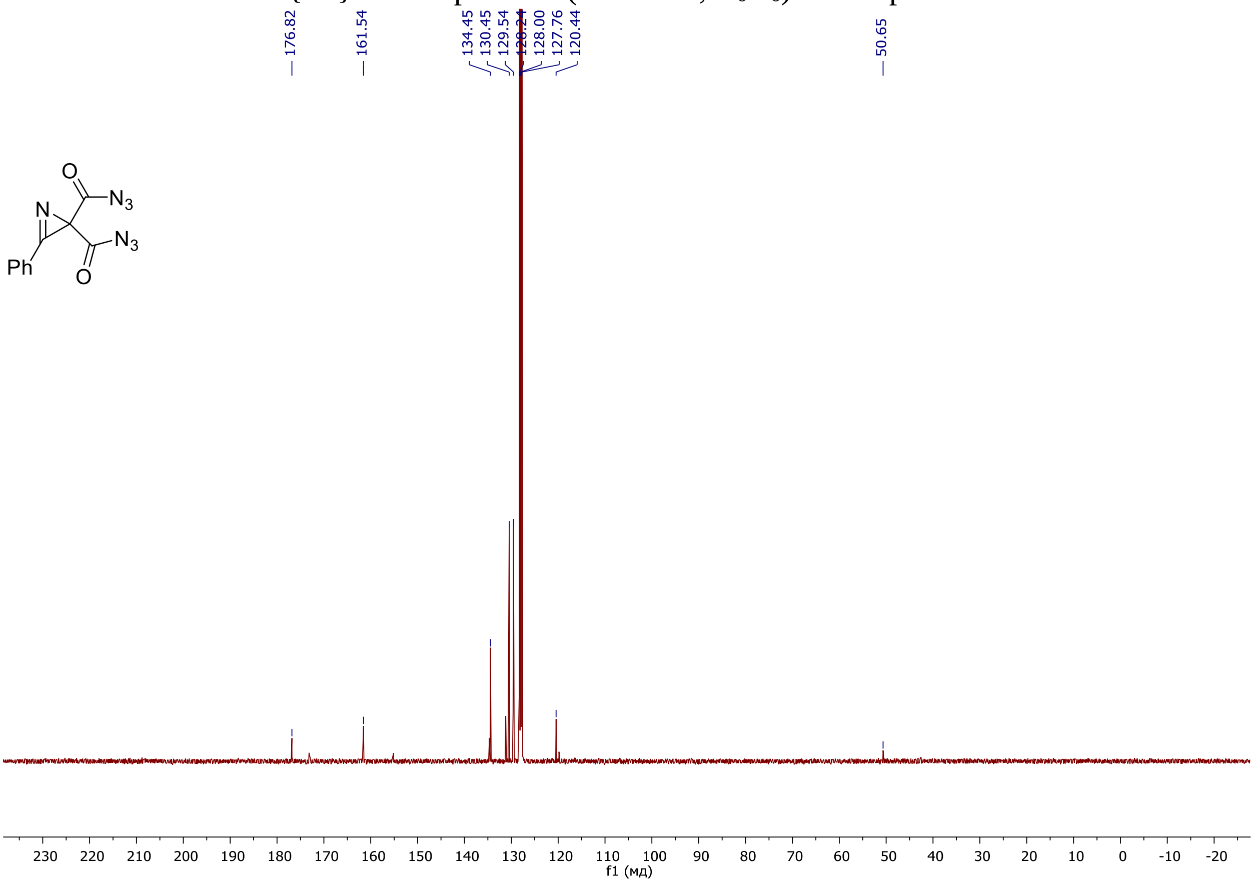

3. X-ray data

Compound 10h (CCDC 2368949)

Single crystals of **10h** were grown by slow evaporation of its solution in $CHCl_3$. A suitable crystal was selected and intensity data were collected on a SuperNova, Dual, Cu at home/near, Atlas diffractometer. The crystal was kept at 100.00(10) K during data collection. Using Olex2 [9], the structure was solved with the ShelXT [10] structure solution program using Intrinsic Phasing and refined with the olex2.refine [11] refinement package using Gauss-Newton minimisation.

Figure S1. X-ray crystal structure of compound **10h** with 50% ellipsoid probability (CCDC 2368949)

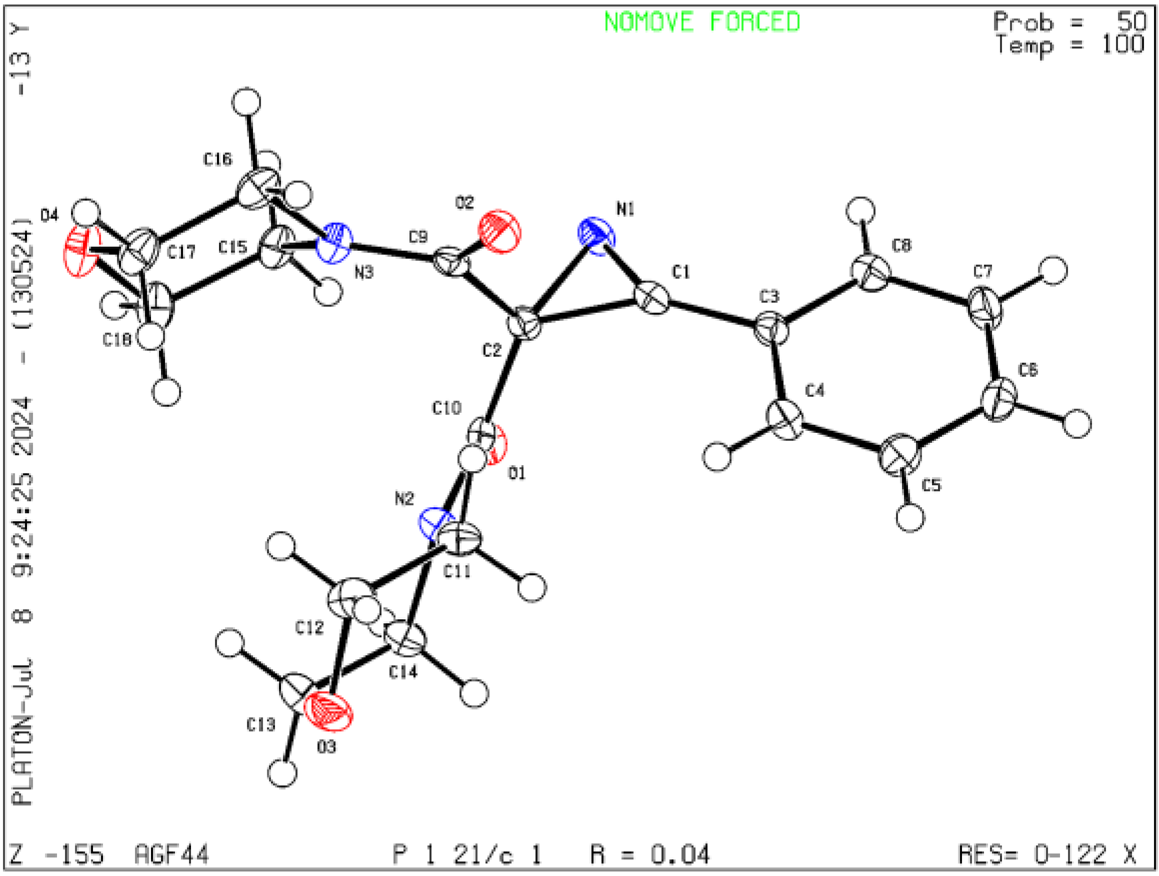

Table S1. Crystal data and structure refinement for **10h**

Empirical formula	$C_{18}H_{21}N_3O_4$
Formula weight	343.385
Temperature/K	100.00(10)
Crystal system	monoclinic
Space group	$P2_1/c$
a/Å	12.0780(3)
b/Å	13.3059(2)
c/Å	11.0413(2)
α/°	90
β/°	107.891(2)
γ/°	90
Volume/Å^3	1688.63(6)
Z	4
ρ_{calc}g/cm^3	1.351
μ/mm^{-1}	0.798
F(000)	730.6
Crystal size/mm^3	0.28 × 0.22 × 0.16
Radiation	Cu Kα (λ = 1.54184)
2Θ range for data collection/°	10.16 to 140
Index ranges	$-15 \leq h \leq 15$, $-16 \leq k \leq 16$, $-13 \leq l \leq 6$
Reflections collected	7441
Independent reflections	3200 [R_{int} = 0.0272, R_{sigma} = 0.0293]
Data/restraints/parameters	3200/0/226
Goodness-of-fit on F^2	1.042
Final R indexes [I>=2σ (I)]	R_1 = 0.0358, wR_2 = 0.0904
Final R indexes [all data]	R_1 = 0.0429, wR_2 = 0.0952
Largest diff. peak/hole / e Å^{-3}	0.37/-0.29

4. References

1. Zhu, Y.-M.; Xu, P.; Wang, S.-Y.; Ji, S.-J. *J. Org. Chem.* **2019**, *84*, 11007–11013.
2. Kafle, B.; Aher, N. G.; Khadka, D.; Park, H.; Cho, H. *Chem. Asian J.* **2011**, *6*, 2073–2079.
3. Galenko, E. E.; Galenko, A. V.; Khlebnikov, A. F.; Novikov, M. S.; Shakirova, J. R. *J. Org. Chem.* **2016**, *81*, 8495–8507.
4. Clark, A. D.; Ha, U. T.; Prager, R. H.; Smith, J. A. *Aust. J. Chem.* **1999**, *52*, 1029–1033.
5. Kay, A. J. ; Woolhouse, A. D.; Gainsford, G. J.; Haskell, T. G.; Wyss, C. P.; Giffin, S. M.; McKinnieb, I. T.; Barnes, T. H. *J. Mater. Chem.* **2001**, *11*, 2271–2281.
6. Anderson, D. J. A. *J. Org. Chem.* **1986**, *51*, 945–947.
7. Serebryannikova, A. V.; Galenko, E. E.; Novikov, M. S.; Khlebnikov, A. F. *J. Org. Chem.* **2019**, *84*, 15567–15577.
8. Beccalli, E. M.; Marchesini, A. *J. Org. Chem.* **1987**, *52*, 3426–3434.
9. Rostovskii, N. V.; Agafonova, A. V.; Smetanin, I. A.; Novikov, M. S.; Khlebnikov, A. F.; Ruvinskaya, J. O.; Starova, G. L. *Synthesis* 2017, 4478-4488**.**
10. Dolomanov, O. V.; Bourhis, L. J.; Gildea, R. J; Howard, J. A. K.; Puschmann, H. *J. Appl. Cryst*. **2009**, *42*, 339– 341.
11. Sheldrick, G. M. *Acta Cryst*. **2015**, *A71*, 3–8.
12. Sheldrick, G. M. *Acta Cryst.* **2015**, *C71*, 3–8.
